# Multiresidue Pesticides Analysis of Vegetables in Vietnam by Ultrahigh-Performance Liquid Chromatography in Combination with High-Resolution Mass Spectrometry (UPLC-Orbitrap MS)

**DOI:** 10.1155/2019/3489634

**Published:** 2019-05-08

**Authors:** Nam Vu-Duc, Trung Nguyen-Quang, Thuy Le-Minh, Xuyen Nguyen-Thi, Tri Manh Tran, Hai Anh Vu, Lan-Anh Nguyen, Tien Doan-Duy, Bui Van Hoi, Cam-Tu Vu, Dung Le-Van, Lan-Anh Phung-Thi, Hong-An Vu-Thi, Dinh Binh Chu

**Affiliations:** ^1^Center for Research and Technology Transfer, Vietnam Academy of Science and Technology (VAST), 18 Hoang Quoc Viet, Hanoi 100000, Vietnam; ^2^Faculty of Chemistry, VNU University of Science, Vietnam National University-Hanoi, 19 Le Thanh Tong, Hanoi 100000, Vietnam; ^3^Institute of Chemistry, Vietnam Academy of Science and Technology, 18 Hoang Quoc Viet, Hanoi 100000, Vietnam; ^4^Department of Water-Environment-Oceanography, University of Science and Technology of Hanoi (USTH), Vietnam Academy of Science and Technology (VAST), 18 Hoang Quoc Viet, Hanoi 100000, Vietnam; ^5^Department of Chemistry, Vietnam Military Medical University, 160 Phung Hung, Hadong, Hanoi 100000, Vietnam; ^6^School of Environmental Science and Technology, Hanoi University of Science and Technology, 1 Dai Co Viet, Hanoi 100000, Vietnam; ^7^Department of Analytical Chemistry, School of Chemical Engineering, Hanoi University of Science and Technology, 1 Dai Co Viet, Hanoi 100000, Vietnam

## Abstract

An ultrahigh-performance liquid chromatography in combination with high-resolution mass spectrometry Thermo Q-Extractive Focus Orbitrap MS has been introduced for analysis of multiclass pesticides in vegetable samples collected in Hanoi, Vietnam. Multiclass pesticides were separated on the Thermo Hypersil Gold PFP column utilizing a gradient of the mobile phase consisting of 5 mM ammonium formate, 0.1% formic acid in deionized water, and methanol. The target analytes were detected in the full-scan mode on Thermo Scientific Q-Exactive Focus Orbitrap MS for quantitation at the optimum operating conditions. These conditions included, but not limit to, the resolution of 70000 at the full width at half maximum in both positive and negative mode, mass range from 80 to 1000 *m/z*, and optimized parameters for the heated electrospray ionization source. The identification of the analytes in real samples was based on retention times, mass to charge ratios, mass accuracies, and MS/MS spectra at the confirmation mode with the inclusion list of target analytes. The mass accuracies of target analytes were from −4.14 ppm (dinotefuran) to 1.42 ppm (cinosulfuron) in the neat solvent and from −3.91 ppm (spinosad D) to 1.29 ppm (cinosulfuron) in the matrix-matched solution. Target analytes in the vegetable-based matrix were extracted by the QuEChERS method. Some critical parameters of the analytical method such as linearity, repeatability, limit of detection, and limit of quantitation have been evaluated and implemented. Excellent LOD and LOQ of the developed method were achieved at the range of 0.04–0.85 and 0.13–2.9 *μ*g·kg^−1^, respectively. Intraday and interday repeatability of the analytical signal (peak area, *n*=6) of the developed method were below 3% and 10%, correspondingly. The matrix effect, extraction recovery, and overall recovery were fully investigated by spiking experiments. Experimental results demonstrated that the ionization suppression or enhancement was the main contribution on the overall recoveries of target analytes. Finally, the in-house validated method was applied to pesticides screening in vegetables samples in local villages in Hanoi, Vietnam. The concentrations of all target analytes were below limit of quantitation and lower than US-FDA or EU maximum residue levels.

## 1. Introduction

The residual of pesticides in the foods is global, concerning in the context of food safety. Pesticide is used in the agriculture in order to protect crops. Therefore, pesticide presents as residue in food, especially in vegetables. The maximum residue level of pesticide in vegetables is lower than 0.01 mg·kg^−1^ according to EU regulation [1] and 0.01 mg·kg^−1^ according to US-FDA regulation as well [2]. Other regions have their own standards, regulations, requirements, and maximum residual levels of pesticides in their food production and consumption [3, 4]. In Vietnam, the pesticide residue level in food in general and in vegetables was also monitored. According to Regulation TT50/2016/TT-BYT of the Ministry of Health, Vietnam, the maximum residue level of pesticides ranged from 0.01 to 100 mg·kg^−1^, depending on each kind of pesticides or vegetables [5]. Ministry of Agriculture and Rural Development, Vietnam (MARD), also regulated maximum levels used of more than 1700 different pesticides. Besides, more than 30 different pesticides were banned in agriculture according to Regulation TT03/2018/TT-BNNPTNT of MARD [6].

Recently, many mass spectrometry techniques have been proposed for analysis of pesticides in environmental samples such as GC-EI-MS, GC-EI-MS/MS, GC-CI-MS, LC-ESI-MS, and LC-ESI-MS/MS [7–13]. Among these methods, LC-MS/MS has been the most popular for pesticide analysis, especially for analysis of multiclass pesticides [14, 15]. In addition, the high-resolution mass spectrometry-based methods with several advantages, namely, excellent accurate mass and high sensitivity, have been introduced for screening of residual pesticides in food matrices [9]. Several sample preparation procedures have been reported, for example, liquid-liquid extraction and solid-phase extraction for such kind of analytes [16]. Besides, QuEChERS (Quick, Easy, Cheap, Effective, Rugged, and Safe) sample preparation method has been known as the most powerful for removing isobaric and nonisobaric interference compounds in the food matrices. This method could be proposed as a “golden standard” for the sample preparation, especially in multiclass residue analysis. In the recent reports, a QuEChERS has been in combination with high-resolution mass spectrometry for screening of pesticides, their metabolites, or multiclass compounds in various food matrices [17–19], for instance, in baby food [20], in fruits, in vegetables [21], and in surface water [22]. Therefore, a fast, sensitivity, and reliable analytical method is necessary for quality control of vegetable-based food in domestic, imported, or exported food matrices (vegetable, fruit, and meat).

In this work, a QuEChERS sample preparation and reversed-phase liquid chromatography in combination with high-resolution Orbitrap MS have been introduced for analysis of multiclass pesticides in vegetable samples. The ionization suppression or enhancement has been also investigated by spiking experiments. The in-house validation of the developed method including limit of detection (LOD), limit of quantitation (LOQ), linearity, short- and long-term stability, matrix effect, and overall recovery has been performed via spiking experiments, according to the guidelines of the USA-FDA, EU [23, 24]. Finally, the developed method was applied to screen multiresidues in vegetable samples, which were collected from several villages in Hanoi, Vietnam.

## 2. Materials and Methods

### 2.1. Chemicals and Reagents

Fifty-three pesticide standards (high purity grade, >90%) were purchased from Dr. Ehrenstorfer GmbH. Acetonitrile (ACN, LC-MS grade), methanol (MeOH, LC-MS grade) for HPLC with 99.80% of purity, and formic acid (FA, ACS reagent) were from Merck (Merck, Singapore). Ammonium formate (LC-MS grade, Sigma-Aldrich, Singapore) was also used for preparation of the mobile phase. Standard compounds were classified into 30 groups and used to prepare individual stock solutions around 1000 *μ*g·mL^−1^ in appropriate solvents such as acetone (GC-MS grade), methanol, *n*-hexane (GC-MS grade), acetonitrile, and ethanol (ACS reagent) in amber vials. The mixed standard solutions of all target analytes (10 *μ*g·mL^−1^) were prepared and diluted with acetonitrile. Stock standards were stored in the amber LC vial at 4°C. The working standard solutions were daily prepared by diluting the mixed standard solution in the mobile phase. The mobile phase was daily prepared by dissolving appropriate amount of ammonium formate in methanol/deionized water (Milli-Q Integral 3, Merck Millipore, France) containing 0.1% formic acid. The mobile phase was degassed in the ultrasonic bath (S 100H, Elma, Germany) to eliminate dissolved gas.

### 2.2. Instrumentation

An ultrahigh-performance liquid chromatography (UPLC) system including the column oven and thermostat autosampler (Ultimate 3000, Thermo Fisher Scientific, Bremen, Germany) in combination with the Thermo Scientific Q-Exactive Focus Orbitrap MS (Thermo Fisher Scientific, Bremen, Germany) was used for data acquisition.

For liquid chromatographic separation, Hypersil GOLD PFP column (150 × 2.1 mm, 3 *μ*m, Thermo Fisher Scientific, USA) was used for separation of target analytes at the temperature of 40°C. The binary mobile phases were 0.1% FA + 5 mM HCOONH_4_ in H_2_O (A) and 0.1% FA + 5 mM HCOONH_4_ in MeOH (B). The gradient elution started at 2% B in 0.25 minutes, raised to 30% B in 0.75 minutes, and linearly increased to 100% B in 24 minutes (held for 5 minutes). In the end, the eluent was restored to initial conditions in 0.5 minutes and held for 7.0 minutes to reequilibrate the column for the next injection. Total time for chromatographic separation was 37.5 minutes. The flow rate was constantly kept at 0.3 mL·min^−1^ during the whole chromatographic analysis process. Both samples and standard solutions were kept at 10°C in the sample tray. A 5 *µ*L of standard or samples was injected into LC-Q-Exactive Focus Orbitrap MS system via an autosampler. The needle and the sample loop in the autosampler were washed triplicate, using the mixture of methanol and deionized water (1 : 1, v : v).

A high-resolution mass spectrometer Thermo Scientific Q-Exactive Focus Orbitrap MS equipped with heated electrospray ionization (HESI) and working at 70000 full width at half maximum (FWHM) resolution (at 200 Da) was operated in both positive and negative electrospray ionization modes. The mass spectrometer was calibrated before each batch of measurement by using Pierce positive/negative ion mass calibration solution (Thermo Fisher, USA). This system was optimized by the direct infusion experiment, using the mixture of pesticides standard solutions in the mobile phase. Optimum operating conditions were achieved with following parameters: sheath gas pressure at 32 psi; auxiliary gas flow rate at 7 L·min^−1^; sweep gas flow rate at 1 L/min; spray voltage +2800 V; and −2500 V for positive and negative ionization mode, respectively; capillary temperature at 320°C; vaporizer temperature at 295°C; and S-lens RF level at 50 V. The HRMS was acquired under full MS mode (resolution 70000-FWHM at 200 Da) over the mass range *m/z* of 80–1000 for both positive and negative ionization mode, and it was conducted to measure the target ions of precursors. The full MS/dd-MS^2^ (full-scan and data-dependent MS/MS mode) could simultaneously record the MS/MS (fragmentation) spectra for the precursors. Besides, the full MS/confirmation mode (with an inclusion list of target analytes) was also used to confirm fragments of the selected precursors. The dd-MS^2^ with confirmation mode conditions was set up with the following parameters: resolution 17500 FWHM; mass isolation window 1.0 Da; maximum and minimum automatic gain control (AGC) target 8 × 10^3^ and 5 × 10^3^, respectively; normalized collision energy (NCE) 30%; spectrum data format for confirmation: centroid. All the parameters of the UHPLC-HRMS system were controlled through Thermo Scientific Xcalibur software version 4.0 (Thermo Scientific, Bremen, Germany).

### 2.3. Sample Preparation

**Figure 1:**
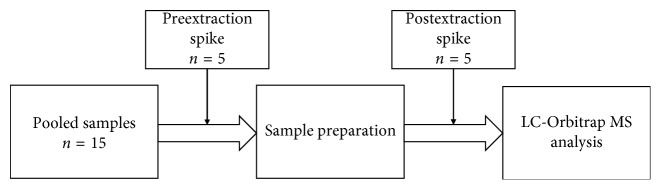
Experimental setup for assessment of recovery, matrix effect, and entire sample preparation procedure.

Vegetable samples were collected from local villages in Hanoi, Vietnam, and stored at 4°C until analysis. In brief, 10 g of each homogenized sample was weighed into a 50 mL QuEChERS centrifuge tube (Thermo Scientific, USA), which contained 4 g MgSO_4_, 1 g NaCl, 1 g Na_3_ citrate, and 0.5 g Na_2_ citrate. After that, 10 mL of ACN was added into the QuEChERS tube. The sample was placed in an ultrasonic bath for 5 minutes before centrifuging at 4450 × g for 15 minutes (Z 326 k, Hermle, Germany). The supernatant was filtrated through 0.45 *μ*m Titan 3, PTFE membrane (Thermo Scientific, USA). The clear aqueous solution was collected into the 2 mL amber LC vial (Thermo Scientific, USA) and then injected into UPLC-Q-Exactive Focus Orbitrap MS system via the autosampler at the optimum experimental conditions. For recovery testing, the experiment was conducted with three groups of pooled samples, which were the mixtures of six types of homogenized vegetables (containing the same amount of cabbage, white mustard, Chinese spinach, green mustard, water morning spinach, and edible chrysanthemum in mixture) to control the effect of matrix. The individual matrix was also investigated in the same manner. The first set of pooled samples was performed following the above procedure. The second set and the last set of pooled samples were spiked preextraction and postextraction, respectively, at the concentration of 50 ng·mL^−1^ for all target analytes in the final solution, as shown in Figure 1. In addition, the same experiments were conducted with three vegetables: cabbage, white mustard, and edible chrysanthemum, which are planted most popularly in the winter time in Vietnam. Besides, real vegetable samples containing cabbage, white mustard, Chinese spinach, green mustard, water morning spinach, and star gooseberry were prepared as the same manner as above for quantitation of pesticides. The concentrations of target analytes in real samples were calculated by external calibration curves.

### 2.4. Matrix Effect in LC-Orbitrap MS

In liquid chromatography tandem mass spectrometry, the matrix is the most important factor that affects the reproductivity of the analytical method. For the assessment of the matrix effect, several designs of experiments were proposed, for example, postextraction spiking experiments, matrix-match calibration curves, and isotopic labelled internal standard spiking experiments [25, 26]. In this study, the matrix effect was investigated by postextraction spiking experiments. Pesticide standards were spiked pre- and postextraction during the sample preparation procedure. A set of 15 pooled samples was used for such experiments as in Figure 1.

Matrix effect (ME), in terms of signal suppression/enhancement, was investigated and assessed by comparing analytical signals of analytes in samples postextraction spike with analytes to those in the “neat” solvent. The matrix effect was calculated by the following equation:

(1) $$\begin{array}{c} \text{ME }\left(\text{\%}\right)=100\;*\;\frac{\text{peak area of target }{\text{analyte}}_{\text{postextraction spike}}}{\text{peak area of target }{\text{analyte}}_{\text{in standard solution}}}. \end{array}$$

Ideally, a value of 100% means the absence of the matrix effect on the MS measurement. ME lower than 100% and higher than 100% are indicated to be ionization suppression and enhancement, respectively. ME is acceptable when its absolute value ranged from 80% to 120%. Any value of ME that is outside that range denotes matrix effect occurs in the MS measurement [10, 27]. In addition, the losing of analytes or contamination in the sample preparation step was also addressed in this study. In order to figure out the contribution of each step in the entire sample preparation procedure, spiking experiments were carried out before and after sample preparation. Therefore, the recovery of the extraction step (RE) (*R*) is calculated as follows:

(2) $$\begin{array}{c} \text{RE }\left(\text{\%}\right)=100\;*\frac{\text{peak area of target }{\text{analyte}}_{\text{preextraction spike}}}{\text{peak area of target }{\text{analyte}}_{\text{postextraction spike}}}. \end{array}$$

The recovery of the entire of sample preparation is calculated as follows:

(3) $$\begin{array}{c} \text{R }\left(\text{\%}\right)=100\;*\frac{\text{peak area of target }{\text{analyte}}_{\text{preextraction spike}}}{\text{peak area of target }{\text{analyte}}_{\text{in standard solution}}}. \end{array}$$

By using three above equations, the recovery of each step could be investigated and assessed. The recovery of each step is very noteworthy and important for development and validation of a new analytical method, in terms of overall extraction efficiency. The LOD, LOQ, MQLs, and MDLs were assessed by spiking standards in the real matrix in pooled samples and individual matrix as well. Therefore, the matrix effect, extraction efficiency, and overall recovery were taken into account for calculation of LOD and LOQ.

### 2.5. Fragmentation Pathway and Mass Accuracy

The mass accuracy of all target analytes in standard solution and matrix-match solution was assessed by injecting independently five times of 50 ng·mL^−1^ of all analytes in mobile phase/matrix-match solution into the LC-Q-Exactive Orbitrap MS. The mass of the protonated molecular ion (in the positive ionization mode) or deprotonated molecular ion (in the negative ionization mode) was extracted by Thermo qualitative software version 4.0. The theoretical mass was calculated by online enviPat Web 2.2 from Eawag aquatic research (https://www.envipat.eawag.ch/index.php). The mass accuracy was calculated by subtracting the theoretical mass from experimental one and then dividing the result by the theoretical mass and presented in part per million mass accuracy. The fragmentation pathway of all target analyte was performed at 30% NCE (normalized collision energy) at resolution 17500 FWHM and crosschecked using Mass Frontier software version 7.0 (Thermo Scientific, USA). Mass fragmentation pathway is also an interesting topic, especially in the context of the nontargeted analysis. The fragmentation pathway of pesticides has been reviewed on the work of Niessen [28]. The product ions with associate mass accuracy of all target analytes are listed in Table S5 in Supplemental Materials. In addition, identification points (IPs) for confirmation of target analytes presenting in the samples were calculated according to European Council Directive 96/23/EC for MS-based methods [29]. The identification of the analytes was based on the protonated/deprotonated molecular mass to charge ratio, at least two daughter ions in MS/MS confirmation modes, isotopic pattern, and mass accuracy of these ions. Therefore, the minimum IPs number achieved 5 in this work.

### 2.6. Quality Control

The Thermo Orbitrap Q-Exactive Focus MS was calibrated by using Pierce positive/negative ion mass calibration solution (Thermo Fisher, USA) before each batch of samples. Before each batch, the quality control (QC) sample was injected five-time repeatability for checking intensity and retention time. After each 10 samples, three blank samples (mobile phase solution) were injected in triplicate for assessment of the carry-over effect. In total, the number of method blank and quality control samples was approximately 20% of the total number of injections on LC-Orbitrap MS, as a proposal from Peters et al. [30].

### 2.7. Data Evaluation

Quantification of pesticides in vegetables was performed by Thermo Trace Finder version 3.3 (Thermo Scientific, Bremen, Germany) using external calibration curves. The mass accuracy of quantitative ions was set at 5 ppm for all pesticides in both negative and positive ionization modes. Weighted and nonweighted linearity and quaternary of the peak areas plotted as a function of concentration of the target analytes were used for quantification. The confirmation of analytes presenting in the real samples was based on the retention times, mass accuracies, isotopic pattern, and MS/MS spectrum at 30% of the normalized collision energy. The peak area was extracted and integrated without a smooth factor in 5 ppm mass resolution window for confirmation. Other important parameters of the developed method are LOD and LOQ. The LOD and LOQ of the developed method were assessed by spiking experiments at low concentrations (at level 10 ng·g^−1^) and then injecting into the LC-Orbitrap MS at the optimized operating conditions. The signal to noise ratio, which was determined according to European Pharmacopoeia guideline, was taken into account for the calculation of LOD (3 ∗ S/N) and LOQ (10 ∗ S/N) [30–33].

## 3. Results and Discussion

### 3.1. Chromatographic Separation of All Target Analytes

**Figure 2:**
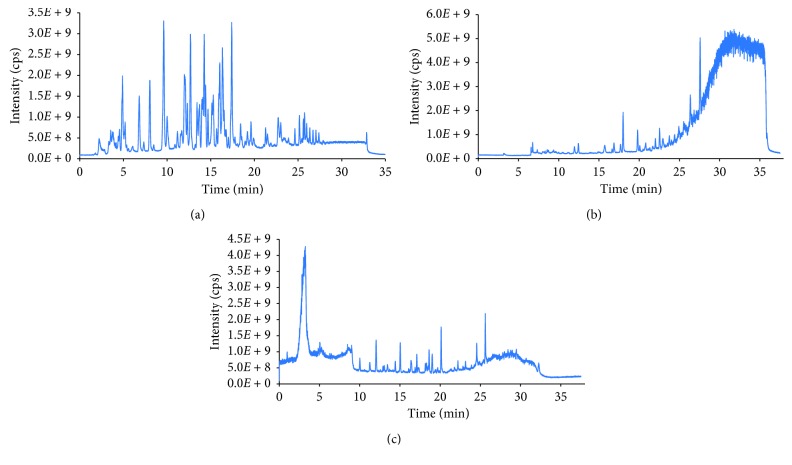
Total chromatogram of multiclass pesticides on the different stationary phases: (a) PFP column; (b) Hypersil ODS; (c) HyperClone™ 5 *μ*m ODS 120 Å.

Because of the nature of multiclass pesticides in the standard solutions, reversed-phase column was the best choice for separation. Many reversed-phase columns with different kinds of stationary phases have been introduced for pesticides analysis. In this work, the Thermo Hypersil GOLD PFP column (silica-based pentafluorophenyl endcapped stationary phase) has been used for chromatographic separation of multiclasses pesticides. The advantages of the PFP stationary phase for retaining of polar compounds were assessed by Si-Hung et al. [34]. Separation mechanism of the PFP column is multi-interactions, such as hydrogen bonding, dipole-dipole interactions, and hydrophobic between functional groups on the surface of stationary phase and analytes, especially the *π*-*π* interaction of the aromatic-like compounds with the stationary phase [35, 36]. For testing, several available columns in our lab were used such as Hypersil ODS (C18, 125 × 2.1 mm, 5 *μ*m, Thermo Fisher Scientific, USA) and HyperClone™ 5 *μ*m ODS 120 Å (C18, 125 × 4.0 mm, 5 *μ*m, Phenomenex, USA). Total ion chromatograms in the positive ionization mode of the mixture standard (500 ng·mL^−1^) on three LC columns: Thermo Hypersil GOLD PFP, Hypersil ODS, and HyperClone™ were depicted in Figures 2(a)–2(c), respectively. It is clearly shown that the Hypersil GOLD PFP LC column was the best separation for multiclass pesticides in terms of separation efficiency and peak shape. Therefore, this column has been chosen for the further experiments.

In addition, components of mobile phase, organic modifiers, and other factors also have influenced the chromatographic separation efficiency. In this work, the mobile phase containing methanol, ammonium formate, and formic acid was used as the suggestion from Lee et al. with some modifications [37]. For optimization of chromatographic conditions, the effects of various method parameters such as mobile phase, flow rate, and solvent ratio were evaluated; the chromatographic parameters such as peak asymmetric factor, resolution, and column efficiency were calculated. The best results were obtained with a gradient mobile phase composition of 5 mM ammonium formate, 0.1% formic acid, and methanol, at a flow rate of 0.3 mL·min^−1^ as mentioned above. Adding 5 mM ammonium formate in the mobile phase improved the chromatographic peak shape of the target analyte, especially the compounds that were eluted in the end of chromatogram [38].

Six independent standard solutions were prepared and injected into the LC-Orbitrap MS in triplicate at the optimum operating conditions as above. The short-term (in 3 hours of continuous measurement) and long-term (in 24 hours of continuous measurement) stabilities of retention time in the mobile phase and in matrix-match solution were assessed and presented by relative standard deviation (RSD). The relative standard deviation of short- and long-term stabilities of retention time of these compounds are calculated and presented in Table 1. Stability of all target analytes in terms of retention time was calculated and is listed in Table S1 of Supplemental Materials.

**Figure 3:**
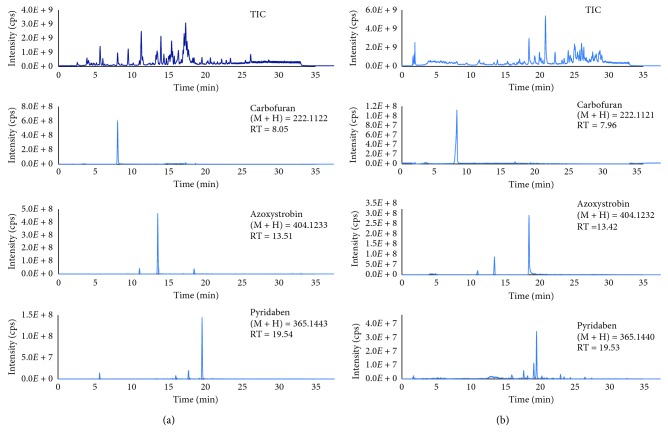
The total ion and extracted ion chromatogram of the target analytes in the standard solution (a) and in the matrix-match solution (b) on the Thermo Hypersil GOLD PFP column, and other operating conditions are mentioned in Section 2.2. The first and second numbers on the right upper are mass-to-charge ratio and retention time, respectively.

As can be seen from Table 1, the excellent repeatability of retention time of three selected analytes was achieved, and relative standard deviation of short- and long-term stabilities in standard solution were below 0.18 and 0.53%, respectively. The repeatability of retention time of the target analytes was also investigated in the real sample by postextraction spiking experiments. In the matrix-match solution, relative standard deviations of short- and long-term stabilities of retention time of these compounds were below 0.27 and 0.36%, respectively. The total ion chromatogram (TIC) and extracted ion chromatogram (EIC) of three representative pesticides in the standard solution and the matrix-match solution are shown in Figures 3(a) and 3(b), respectively.

It is clear from Figure 3 that the retention times of three selected analytes were observed a little earlier in the matrix-match solution. However, relative standard deviations of three selected analytes were below 0.3% for both short- and long-term stabilities in terms of retention time. It should be concluded that the developed method was excellent and stable for chromatographic separation of the multiclass pesticides. In addition, sample matrix was not affected on the chromatographic separation. The short- and long-term stabilities of retention time of all target analytes on the Thermo HyperGold PFP column are listed in Table S1 in Supplemental Materials. Relative standard deviations of short- and long-term stabilities of retention time of all target analytes were in the range of 0.07–0.2% and 0.13–0.29%, respectively. The total ion chromatogram and extracted ion chromatograms of all target analytes in standard solution and matrix-match solution with both ionization modes are depicted in Figures S1–S4 in Supplemental Materials, respectively.

### 3.2. Fragmentation Pathway and Mass Accuracy

For assessment of mass accuracy, five independent mixtures of target analytes in the mobile phase and matrix-match solutions were injected on UPLC-Orbitrap MS at the optimum operating conditions. The mass accuracies of target analytes are calculated and presented as parts per million (ppm) in Table 2.

As clearly demonstrated in Table 2, the mass accuracy of three target analytes was achieved below 2.5 ppm. It should be noted that the excellent mass accuracy was achieved in both pure solvent and matrix-match solutions. The mass accuracies of all target analytes are calculated and presented in Table S1 in Supplemental Materials. Mass accuracies of all target analytes were below ±3 ppm for both matrices: pure solvent and matrix-match solution. In addition, the tandem mass spectra of all target analytes in standard solution and in the matrix-match solution have been performed at 17500 FWHM resolution, 1 Da isolated mass window, and 30% normalized collision energy. Mass accuracy of all product ions is listed in Table S5 in Supplemental Materials. It was clearly shown that mass accuracy of all product ions was achieved below 5 ppm, except for dinotefuran (−11.5 ppm). The lower mass accuracy of product ions in comparison with the precursor ion should be attributed by lower resolution setting in the confirmation MS/MS mode (17500 FWHM at 200 Da).

### 3.3. Validation of the Developed Method

#### 3.3.1. Stability of the Analytical Signal

Short term and long term of the analytical signal play as a critical role in terms of measurement uncertainty of the developed analytical method. For assessment of the analytical signal, two sets of five solutions containing all target analytes at a concentration of 50 ng·mL^−1^ were prepared in solvent and in matrix-match solution, respectively. These solutions were injected on the LC-LC Q-Exactive Focus Orbitrap MS at the optimum conditions. The short-term and long-term stabilities were performed in three and twenty-four hours of continuous measurement, respectively. The peak area was integrated on TraceFinder version 3.3 (Thermo Scientific, Bremen, Germany) with 5 ppm mass accuracy window. The peak area and relative standard deviation of peak are shown in Table 3.

As can be seen from Table 3, excellent repeatability of the analytical signal was achieved for both short term and long term. RSD of the peak area of short term and long term was below 3.68% and 5.78% for both standard and matrix-match solutions, respectively. It was worthy to note that the good repairability of the analytical signal was achieved in this study. The relative standard deviation of the analytical signal of all target analytes is listed in Table S6 in Supplemental Materials. All RSD values of the analytical signal were lower than the acceptable value according to Horwitz [39].

#### 3.3.2. Linearity, LOD, and LOQ

Seven independent standard solutions (with concentrations of 10, 25, 50, 100, 250, 500, and 1000 *μ*g·L^−1^ for all target analytes) of all targeted analytes were prepared by dilution stock solution in the mobile phase and injected triplicate into the LC-Orbitrap MS at the optimum operating conditions. The peak area of the target analyte was taken into account for quantitation. The average peak area was fitted either linearity or as a quadratic function of concentration of target analyte. The nonweighted linearity/quadratic or weighted linearity/quadratic of peak area (*Y*) as a function of concentration (*X*) was performed as a proposal from Gu et al. [40].

As shown in Table 4, the good correlation between analytical signal (peak area) and concentration of analyte (*R* ^2^ > 0.998 for three representative analytes) was achieved. The calibration equations and correlation coefficients of all target analytes are listed in Table S2 in Supplemental Materials.

As can be clearly seen from Table 5, LOD and LOQ of the developed method were high enough for directly analyzing multiclass pesticides in vegetable samples according to US-FDA and European Commission [23, 24]. The LOD and LOQ of all analytes are listed in Table S3 in Supplemental Materials. It should be noted that the developed LOD and LOQ of the method were also comparable with recent publications [41–45].

#### 3.3.3. Overall Recovery and Matrix Effect

Matrix effect (ionization suppression/enhancement) is the most important factor in liquid chromatography in combination with tandem mass spectrometry, especially in electrospray ionization (ESI) mass spectrometry, because it influences the robustness and ruggedness of the analytical method. Ideally, a value of 100% means the absence of the matrix effect on the MS measurement. ME is acceptable when it is in range of 80% to 120%. Any value of ME that is outside that range denotes that the matrix effect occurs in the MS measurement [46]. Due to the fact that there is no vegetable-based matrix-certified reference materials for multiclass pesticides commercially available at the moment, the assessment of the matrix effect and recovery of the sample preparation were performed by spiking experiments in this study as the proposal from B.K. Matuszewski with some modifications [47]. The matrix effect during validation of the developed method was investigated by comparison of the analytical signal of a target analyte (peak area or peak height normally) in the postextraction spiked solution and that of the same target analyte in the mobile phase.

The matrix effect, recovery of sample preparation, and overall recovery were calculated by equations (1)–(3). ME, RE, and *R* of three representative analytes are listed in Table 6.

As can be seen from Table 6, the main factors affected on the overall recoveries of azoxystrobin and pyridaben were both the matrix effect and extraction recovery. However, the ionization enhancement was observed in both cases. ME and RE in case of carbofuran were in an acceptable range (from −20% to 20%). In case of azoxystrobin, a strong enhancement of the analytical signal was observed (40.1% higher than the peak area of this compound in the neat solvent). In addition, low extraction recovery of such compounds was also observed. Interestingly, the overall recovery of this compound was still in an acceptable range (from 80 to 120%), according to SANTE Guideline of European Commission [23, 48]. The experimental results indicated that the matrix effect (ionization suppression/enhancement) had a major contribution to overall recovery of three representative analytes. The matrix effect, extraction recovery, and overall recovery of all target analytes are listed in Table S4a in Supplemental Materials. ME, RE, and *R* of all target analytes were in the range of 50–293%, 52–94%, and 38–209%, respectively. It should be concluded that the major contribution to the overall recoveries of almost all analytes was ionization suppression/enhancement. In addition, extraction recovery, matrix effect, and overall recovery of all target analytes in three individual vegetable matrices (cabbage, white mustard, and edible chrysanthemum) that are mostly planted in the winter in Vietnam were investigated in the same manner as the pooled sample. The results of ME, RE, and *R* are listed in Table S4b in Supplemental Materials. The experimental results indicated that ME, RE, and *R* in an individual matrix sample obtained the same characteristic as in the pooled sample. Therefore, the pooled sample of the individual matrix in the same commodity group could be proposed for assessment of ME, RE, and *R*.

**Figure 4:**
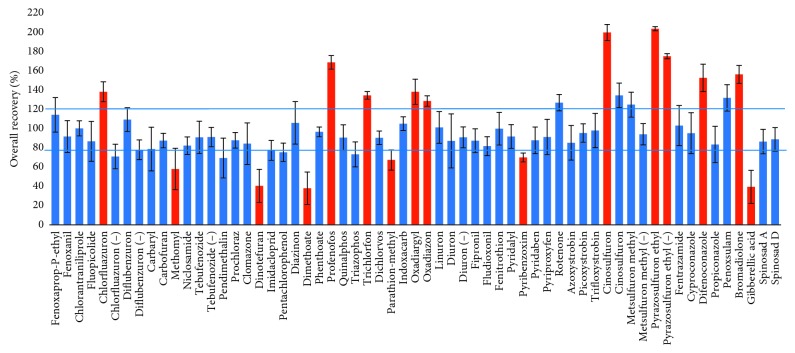
Overall recovery of all targeted analytes in pooled samples analyzed by UPLC-Orbitrap MS. Columns and error bars present the overall recovery and relative standard deviation (*n*=3), respectively. Red color indicates the recovery of the analyte out of an acceptable range (from 80 to 120%). (−) denotes an analyte that is measured in the negative electrospray ionization mode.

Overall recovery of all target analytes in the pooled sample is shown in Figure 4. As clearly shown that an overall recovery of 70% of target analytes was in an acceptable range (from 80 to 120% overall recovery). Overall recovery of 18% and 12% of total number of target analytes was higher and lower than 120% and 80%, respectively.

### 3.4. Analysis of Real Samples

**Figure 5:**
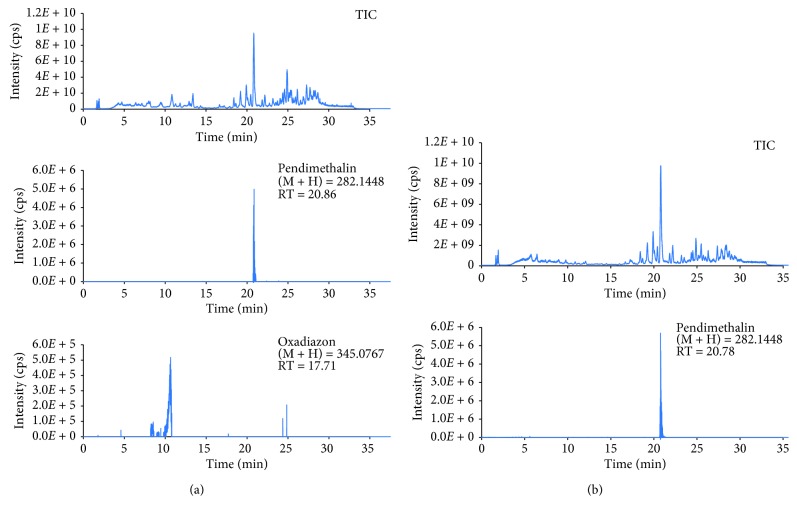
TIC and EICs of pesticides found in edible chrysanthemum (a) and green salad (b).

For application, the in-house validated method was used for analysis of pesticides in vegetable samples collected from local villages in Hanoi, Vietnam. The QuEChERS sample preparation procedure was used for vegetable samples. Total ion chromatogram and extracted ion chromatograms of some pesticides found in the real samples are shown in Figure 5.

As can be seen from Figure 5, only a few analytes were detected in the real samples. For instant, concentration of oxadiazon and pendimethalin in edible chrysanthemum and spinosad D in green salad was found to be 3.8 ng·g^−1^, 9.4 ng·g^−1^, and 12.5 ng·g^−1^, respectively. In addition, some pesticides were detected at below the limit of quantitation. However, the concentration of all detected analytes in the vegetable samples was below the maximum residual level according to USA-FDA, EU, and Vietnamese national regulation as well. Total ion and extracted ion chromatograms of detected analytes in some real samples are also shown in Figures S5 and S6 in Supplemental Materials.

## 4. Conclusion

An UHPLC-Q-Exactive Focus Orbitrap MS analytical method was successfully developed for multiclass pesticides (53 compounds and 30 classes) analysis in vegetable samples using a PFP column. The critical parameters of the analytical method such as linearity, correlation coefficients, LOD, and LOQ have been investigated and implemented. The excellent LOQ of the developed method was achieved. The ionization suppression or enhancement effect was also addressed. The experimental results indicated that the main contribution to overall recovery was the ionization effect on heated electrospray ionization sources. The developed method was applied to screen pesticides in some kinds of vegetables that were collected in local villages in Hanoi, Vietnam. Only few pesticides were found in these samples. Screening of pesticides and their metabolites in the vegetable samples will be focused by using in-house high-resolution mass spectra and MS/MS spectra in the next steps. The further sample clean-up steps will also be taken into account in order to minimize the matrix effect on the liquid chromatography high-resolution mass spectrometry.

## Figures and Tables

**Table 1:** Short-term and long-term stabilities of retention time of three target analytes in the standard solution and in the real matrix.

No	Analytes	Retention time (min)	Standard solution		Matrix-match solution	
			RSD (short term, *n*=6) (%)	RSD (long term, *n*=6) (%)	RSD (short term, *n*=6) (%)	RSD (long term, *n*=6) (%)
1	Carbofuran	8.03	0.18	0.53	0.19	0.31
2	Azoxystrobin	13.49	0.06	0.25	0.27	0.36
3	Pyridaben	19.54	0.09	0.18	0.10	0.22

**Table 2:** Mass and mass accuracy of three representative pesticides in the standard solution and in the matrix-match solution.

No	Analytes	Chemical formula	Theoretical mass^*∗*^ (Da, *m/z*)	Standard solution		Matrix-match solution	
				Experimental mass (Da), mean value, *n*=5	Mass accuracy (ppm)	Experimental mass (Da), mean value, *n*=5	Mass accuracy (ppm)
1	Carbofuran	C_12_H_14_NO_3_	222.1125	222.1122	−1.4	222.1120	−1.8
2	Azoxystrobin	C_22_H_17_N_3_O_5_	404.1241	404.1234	−2.0	404.1231	−2.2
3	Pyridaben	C_19_H_25_ClN_2_OS	365.1449	365.1443	−1.6	365.1441	−2.5

^*∗*^Theoretical mass was calculated through online enviPAT software (https://www.envipat.eawag.ch/).

**Table 3:** Short-term and long-term repeatability of the analytical signal (peak area) of target analytes in UPLC-Orbitrap MS.

No	Analytes	Standard solution, RSD of peak area (%)		Matrix-match solution, RSD of peak area (%)	
		Short term, *n*=5	Long term, *n*=5	Short term, *n*=5	Long term, *n*=5
1	Carbofuran	0.70	4.22	0.77	0.99
2	Azoxystrobin	0.96	5.78	1.17	6.31
3	Pyridaben	1.71	5.54	3.68	4.40

**Table 4:** Calibration curves and correlation of three representative analytes on the LC-Q-Exactive Focus Orbitrap MS.

No	Analytes	*t*_R_ (min)	Polarity	Regression equation	Correlation coefficient (*R* ^2^)
1	Carbofuran	8.03	+	*Y* = 1.557e7 ∗ *X* + 8.567e7	0.9989
2	Azoxystrobin	13.49	+	*Y* = 1.503e7 ∗ *X* + 8.688e7	0.9980
3	Pyridaben	19.54	+	*Y* = 2.855e6 ∗ *X* + 6.367e6	0.9993

**Table 5:** Analytical figures of merit of the LC-Q-Exactive Orbitrap MS method for three representative pesticides.

No	Analytes	*t*_R_ (min)	LOD (ng·mL^−1^)	LOQ (ng·mL^−1^)	MDL^*∗*^ (pg)	MQL^*∗*^ (pg)
1	Carbofuran	8.03	0.09	0.30	0.5	1.5
2	Azoxystrobin	13.48	0.02	0.07	0.1	0.3
3	Pyridaben	19.54	0.09	0.30	0.5	1.5

^*∗*^Absolute method of detection (MDL) and method of quantitation (MQL) are defined as the absolute amount of analyte injected into the LC column.

**Table 6:** Matrix effect, recovery of extraction, and overall recovery of three representative pesticides in pooled samples.

No	Analytes	Class	*t*_R_ (min)	ME ± RSD (%) (*n*=5)	RE ± RSD (%) (*n*=5)	*R* ± RSD (%) (*n*=5)
1	Azoxystrobin	Strobilurin	13.42	140.1 ± 13.8	60.7 ± 11.4	85 ± 17.9
2	Carbofuran	Carbamate	7.96	97.1 ± 1.6	89.9 ± 7.4	87.3 ± 7.5
3	Pyridaben	Pyridazinone	19.47	121 ± 8.7	72.3 ± 10.8	87.5 ± 13.8

## Data Availability

The LC-Orbitrap MS and MS/MS spectra data used to support the findings of this study are available from the corresponding authors upon request.

## Supplementary Materials

Supplementary Materials Table S1: mass accuracy and retention time of target analytes in neat solvent and matrix-matched solutions. Table S2: calibration curves and correlation of analytes on the UPLC-Orbitrap Q-Exactive MS. Table S3: analytical figures of merit of the UPLC-Q-Exactive Orbitrap MS method for pesticide analysis. Table S4a: matrix effect, recovery of extraction, and overall recovery of pesticides in pooled samples. Table S4b: matrix effect, recovery of extraction, and overall recovery of pesticides in three vegetable matrices (cabbage, white mustard, and edible chrysanthemum). Table S5: mass and mass accuracy of analytical product ions. Table S6: short- and long-term stabilities of the analytical signal in standard solution and in matrix-match solution. Figure S1: the total ion chromatogram of the target analytes in the standard solution (positive mode). Figure S2: the total ion chromatogram of the target analytes in the standard solution (negative mode). Figure S3: the total ion chromatogram and extracted ion chromatogram of the target analytes in the matrix-match solution (positive mode). Figure S4: the total ion chromatogram and extracted ion chromatogram of the target analytes in the matrix-match solution (negative mode). Figure S5: the total ion chromatogram and extract ion chromatogram of the target analytes in several real samples (positive mode). Figure S6: the total ion chromatogram and extracted ion chromatogram of the target analytes in several real samples (negative mode).
