# Supplementary material for: Multiresidue Pesticides Analysis of Vegetables in Vietnam by Ultrahigh-Performance Liquid Chromatography in Combination with High-Resolution Mass Spectrometry (UPLC-Orbitrap MS)
Source: J Anal Methods Chem. 2019 May 8;2019:3489634. doi: 10.1155/2019/3489634 (PMC6530207; doi:10.1155/2019/3489634)
Supplement: Supplementary Materials — Table S1: mass accuracy and retention time of target analytes in neat solvent and matrix-matched solutions. Table S2: calibration curves and correlation of analytes on the UPLC-Orbitrap Q-Exactive MS. Table S3: analytical figures of merit of the UPLC-Q-Exactive Orbitrap MS method for pesticide analysis. Table S4a: matrix effect, recovery of extraction, and overall recovery of pesticides in pooled samples. Table S4b: matrix effect, recovery of extraction, and overall recovery of pesticides in three vegetable matrices (cabbage, white mustard, and edible chrysanthemum). Table S5: mass and mass accuracy of analytical product ions. Table S6: short- and long-term stabilities of the analytical signal in standard solution and in matrix-match solution. Figure S1: the total ion chromatogram of the target analytes in the standard solution (positive mode). Figure S2: the total ion chromatogram of the target analytes in the standard solution (negative mode). Figure S3: the total ion chromatogram and extracted ion chromatogram of the target analytes in the matrix-match solution (positive mode). Figure S4: the total ion chromatogram and extracted ion chromatogram of the target analytes in the matrix-match solution (negative mode). Figure S5: the total ion chromatogram and extract ion chromatogram of the target analytes in several real samples (positive mode). Figure S6: the total ion chromatogram and extracted ion chromatogram of the target analytes in several real samples (negative mode). [file 3489634.f1.pdf]

Multiresidue pesticides analysis in vegetables in Vietnam by ultrahigh performance liquid chromatography in combination with high resolution mass spectrometry (UPLC-Orbitrap MS)

Nam Vu-Duc[1], Trung Nguyen-Quang[1], Thuy Le-Minh[1,2], Xuyen Nguyen-Thi[1,2], Tri Manh Tran[2], Hai Anh Vu[2], Lan-Anh Nguyen[2], Tien Doan-Duy[3], Hoi Bui -Van[4], Cam-Tu Vu[4], Dung Le-Van[5], Lan-Anh Phung-Thi[6], Hong-An Vu-Thi[7], Dinh Binh Chu[7]

[1]Center for Research and Technology Transfer, Vietnam Academy of Science and Technology (VAST), 18 Hoang Quoc Viet, Hanoi 100000, Vietnam

[2]Faculty of Chemistry, VNU University of Science, Vietnam National University-Hanoi, 19 Le Thanh Tong, Hanoi 100000, Vietnam

[3]Institute of Chemistry, Vietnam Academy of Science and Technology, 18 Hoang Quoc Viet, Hanoi, 100000, Vietnam

[4]Department of Water - Environment - Oceanography, University of Science and Technology of Hanoi (USTH), Vietnam Academy of Science and Technology (VAST), 18 Hoang Quoc Viet, Hanoi 100000, Vietnam

[5]Department of Chemistry, Vietnam Military Medical University, 160 Phung Hung, Hadong, Hanoi 100000, Vietnam

[6]School of Environmental Science and Technology, Hanoi University of Science and Technology, 1 Dai Co Viet, Hanoi 100000, Vietnam

[7]Department of Analytical Chemistry, School of Chemical Engineering, Hanoi University of Science and Technology, 1 Dai Co Viet, Hanoi 100000, Vietnam

Corresponding author:

1. Dr. Dinh Binh Chu
Department of Analytical Chemistry, School of Chemical Engineering, Hanoi University of Science and Technology, 1 Dai Co Viet, Hanoi 100000, Vietnam
Email: binh.chudinh@hust.edu.vn
Fax: + 84 2438680070
Phone: + 84 2438692206

2. Dr. Nam Vu-Duc

Center for Research and Technology Transfer, Vietnam Academy of Science and Technology, 18 Hoang Quoc Viet, Hanoi 100000, Vietnam
Email: namvd@yahoo.com
Fax: +84 2437568422
Phone: + 84 2437568422

Contents

Table S1: Mass accuracy and retention time of target analytes in neat solvent and matrix matched solutions 3

Table S2: Calibration curves and correlation of analytes on the UPLC-Orbitrap Q Exactive MS 6

Table S3. Analytical figures of merit of the UPLC- Q Exactive Orbitrap MS method for pesticide analysis 8

Table S4a: Matrix effect, recovery of extraction and overall recovery of pesticides in pooled samples 10

Table S4b: Matrix effect, recovery of extraction and overall recovery of pesticides in three vegetable matrices (cabbage, white mustard and edible chrysanthemum) 12

Table S5: Mass and mass accuracy of analytical product ions 14

Table S6. Short- and long-term stability of the analytical signal in standard solution and in matrix-match solution 16

Fig. S1. The total ion chromatogram of the target analytes in the standard solution (positive mode) 18

Fig. S2. The total ion chromatogram of the target analytes in the standard solution (negative mode) 34

Fig. S3. The total ion chromatogram and extracted ion chromatogram of the target analytes in the matrix match solution (positive mode) 40

Fig. S4. The total ion chromatogram and extracted ion chromatogram of the target analytes in the matrix match solution (negative mode) 56

Fig. S5: The total ion chromatogram and extract ion chromatogram of the target analytes in several real samples (positive mode) 62

Fig. S6. The total ion chromatogram and extracted ion chromatogram of the target analytes in several real samples (negative mode) 66

Table S1: Mass accuracy and retention time of target analytes in neat solvent and matrix matched solutions

No	Compound name	Chemical formula	Category	Precursor theorical (polarity)	RT ± rsd, min (n=6)		[M+H] or [M-H] experiment			
					solvent	Match matrix	solvent		Match matrix	
							Mass accuracy	m/z ± SD (n=6)	Mass accuracy	m/z ± SD (n=6)
1	Fenoxaprop-P-Ethyl	$C_{18}H_{16}ClNO_5$	aryloxyphenoxypropionate	362.0790 (+)	17.70 ± 0.27	17.66 ± 0.09	-1.93	362.0783 ± 0.0001	-2.67	362.0780 ± 0.0001
2	Fenoxanil	$C_{15}H_{18}Cl_2N_2O_2$	Amide	329.0818 (+)	15.12 ± 0.09	15.06 ± 0.15	-1.92	329.0812 ± 0.0001	-2.53	329.0810 ± 0.0002
3	Chlorantraniliprole	$C_{18}H_{14}BrCl_2N_5O_2$	anthranilic diamide	481.9781 (+)	11.5 ± 0.18	11.47 ± 0.15	-1.59	481.9773 ± 0.0002	-2.42	481.9769 ± 0.0001
4	Fluopicolide	$C_{14}H_8Cl_3F_3N_2O$	benzamide pyridine	382.9727 (+)	13.40 ± 0.05	13.35 ± 0.21	-1.44	382.9722 ± 0.0001	-2.13	382.9719 ± 0.0001
5	Chlorfluazuron	$C_{20}H_9Cl_3F_5N_3O_3$	Benzoylurea	539.9702 (+)	20.34 ± 0.09	20.34 ± 0.09	-1.73	539.9693 ± 0.0002	-2.53	539.9688 ± 0.0003
				537.9557 (-)	20.18 ± 0.16	20.18 ± 0.08	0.74	537.9561 ± 0.0001	0.68	537.9561 ± 0.0001
6	Diflubenzuron	$C_{14}H_9ClF_2N_2O_2$	Benzoylurea	311.0393 (+)	14.99 ± 0.11	14.93 ± 0.12	-1.98	311.0387 ± 0.0001	-2.63	311.0385 ± 0.0001
				309.0248 (-)	14.76 ± 0.13	14.73 ± 0.24	0.84	309.0251 ± 0.0001	0.86	309.0251 ± 0.0001
7	Carbaryl	$C_{12}H_{11}NO_2$	Carbamate	202.0862 (+)	9.47 ± 0.28	9.43 ± 0.10	-1.15	202.0860 ± 0.0001	-1.73	202.0859 ± 0.0001
8	Carbofuran	$C_{12}H_{15}NO_3$	Carbamate	222.1125 (+)	8.03 ± 0.18	7.99 ± 0.17	-1.20	222.1122 ± 0.0001	-2.25	222.1120 ± 0.0001
9	Methomyl	$C_5H_{10}N_2O_2S$	Carbamate	163.0536 (+)	4.58 ± 0.23	4.27 ± 0.25	-1.64	163.0533 ± 0.0001	-1.64	163.0533 ± 0.0001
10	Niclosamide	$C_{13}H_8Cl_2N_2O_4$	Chloronitrophenol	324.9788 (-)	18.67 ± 0.14	18.67 ± 0.13	0.92	324.9791 ± 0.0001	0.72	324.9790 ± 0.0001
11	Tebufenozide	$C_{22}H_{28}N_2O_2$	Diacylhydrazine	353.2224 (+)	14.68 ± 0.14	14.63 ± 0.18	-2.22	353.2216 ± 0.0001	-2.83	353.2214 ± 0.0002
				351.2078 (-)	14.48 ± 0.16	14.45 ± 0.25	0.63	351.2080 ± 0.0001	0.71	351.2081 ± 0.0001
12	Pendimethalin	$C_{13}H_{19}N_3O_4$	Dinitroaniline	282.1448 (+)	18.64 ± 0.21	18.58 ± 0.13	-1.54	282.1444 ± 0.0001	-2.48	282.1441 ± 0.0001
13	Prochloraz	$C_{15}H_{16}Cl_3N_3O_2$	Imidazole	376.0381 (+)	16.34 ± 0.14	16.28 ± 0.12	-1.82	376.0374 ± 0.0001	-2.53	376.0372 ± 0.0001
14	Clomazone	$C_{12}H_{14}ClNO_2$	Isoxazolidinone	240.0786 (+)	11.44 ± 0.20	11.40 ± 0.13	-1.25	240.0783 ± 0.0001	-2.29	240.0781 ± 0.0001
15	Dinotefuran	$C_7H_{14}N_4O_3$	Neonicotinoid	201.0993 (-)	3.87 ± 0.19	3.72 ± 0.43	-4.14	201.0985 ± 0.0001	-2.82	201.0987 ± 0.0001
16	Imidacloprid	$C_9H_{10}ClN_5O_2$	Neonicotinoid	256.0596 (+)	5.76 ± 0.26	5.74 ± 0.09	-1.56	256.0592 ± 0.0001	-2.41	256.0590 ± 0.0001
17	Pentachlorophenol	C_6HCl_5O	organochlorin- POPs (persistent Organic pollutants)	262.8397 (-)	17.50 ± 0.08	17.49 ± 0.16	1.29	262.8400 ± 0.0001	1.08	262.8400 ± 0.0001
18	Diazinon	$C_{12}H_{21}N_2O_3PS$	Organophosphate	305.1083 (+)	15.40 ± 0.15	15.35 ± 0.16	-1.80	305.1078 ± 0.0001	-2.57	305.1075 ± 0.0001
19	Dimethoate	$C_5H_{12}NO_3PS_2$	Organophosphate	230.0069 (+)	5.67 ± 0.30	5.65 ± 0.13	-1.23	230.0066 ±0.00004	-2.03	230.0064 ± 0.0001
20	Phenthoate	$C_{12}H_{17}O_4PS_2$	Organophosphate	321.0379 (+)	15.25 ± 0.14	15.20 ± 0.15	-1.82	321.0373 ± 0.0001	-2.54	321.0371 ± 0.0002
21	Profenofos	$C_{11}H_{15}BrClO_3PS$	Organophosphate	372.9424 (+)	16.93 ± 0.18	16.89 ± 0.07	-1.12	372.9420 ±0.00004	-2.06	372.9416 ± 0.0001
22	Quinalphos	$C_{12}H_{15}N_2O_3PS$	Organophosphate	299.0614 (+)	14.97 ± 0.12	14.92 ± 0.14	-1.67	299.0609 ± 0.0001	-2.51	299.0607 ± 0.0002

No	Compound name	Chemical formula	Category	Precursor theorical (polarity)	RT ± rsd, min (n=6)		[M+H] or [M-H] experiment			
					solvent	Match matrix	solvent		Match matrix	
							Mass accuracy	m/z ± SD (n=6)	Mass accuracy	m/z ± SD (n=6)
23	Triazophos	$C_{12}H_{16}N_3O_3PS$	Organophosphate	314.0723 (+)	13.93 ± 0.12	13.88 ± 0.20	-1.96	314.0717 ± 0.0001	-2.65	314.0715 ± 0.0001
24	Trichlorfon	$C_4H_8Cl_3O_4P$	Organophosphate	256.9298 (+)	5.20 ± 0.24	5.18 ± 0.14	-1.04	256.9295 ± 0.0001	-2.21	256.9292 ± 0.0001
25	Dichlorvos	$C_4H_7Cl_2O_4P$	Organophosphate	220.9532 (+)	8.19 ± 0.20	8.15 ± 0.14	-0.60	220.9531 ± 0.0001	-1.81	220.9528 ± 0.0001
26	Parathion-methyl	$C_8H_{10}NO_5PS$	Organophosphate	261.9944 (-)	6.56 ± 0.28	6.56 ± 0.20	1.15	261.9947 ± 0.0001	0.76	261.9946 ± 0.0001
27	Indoxacarb	$C_{22}H_{17}ClF_3N_3O_7$	Oxadiazine	528.078 (+)	18.15 ± 0.26	18.10 ± 0.14	-1.74	528.0771 ± 0.0002	-2.49	528.0767 ± 0.0002
28	Oxadiargyl	$C_{15}H_{14}Cl_2N_2O_3$	Oxidiazole	341.0454 (+)	15.97 ± 0.18	15.92 ± 0.16	-1.86	341.0448 ± 0.0002	-2.59	341.0445 ± 0.0001
29	Oxadiazon	$C_{15}H_{18}Cl_2N_2O_3$	Oxidiazole	345.0767 (+)	17.91 ± 0.18	17.86 ± 0.10	-1.69	345.0761 ± 0.0001	-2.56	345.0758 ± 0.0001
30	Linuron	$C_9H_{10}Cl_2N_2O_2$	Phenylurea	249.0192 (+)	12.77 ± 0.14	12.71 ± 0.11	-1.27	249.0189 ±0.00004	-1.81	249.0188 ± 0.0001
31	Diuron	$C_9H_{10}Cl_2N_2O$	Phenylurea	233.0243 (+)	11.28 ± 0.20	11.23 ± 0.13	-0.93	233.0241 ± 0.0001	-1.72	233.0239 ± 0.0001
				231.0097 (-)	11.03 ± 0.23	11.01 ± 0.28	-0.94	231.0095 ± 0.0001	-1.37	231.0094 ± 0.00004
32	Fipronil	$C_{12}H_4Cl_2F_6N_4OS$	Phenylpyrazole	434.9314 (-)	16.39 ± 0.16	16.38 ± 0.16	0.87	434.9318 ± 0.0001	0.61	434.9317 ± 0.0001
33	Fludioxonil	$C_{12}H_6F_2N_2O_2$	Phenylpyrazole	247.0324 (-)	13.98 ± 0.18	13.96 ± 0.20	-0.49	247.0323 ± 0.0001	-0.47	247.0323 ± 0.00004
34	Fenitrothion	$C_9H_{12}NO_5PS$	Phosphorothioate	278.0246 (+)	13.38 ± 0.07	13.32 ± 0.28	-1.74	278.0241 ± 0.0001	-1.80	278.0241 ± 0.0001
35	Pyridalyl	$C_{18}H_{14}Cl_4F_3NO_3$	Pyridalyl	489.9753 (+)	22.23 ± 0.04	22.26 ± 0.17	-1.94	489.9744 ± 0.0001	-2.93	489.9739 ± 0.0001
36	Pyribenzoxim	$C_{32}H_{27}N_5O_8$	Pyrimidinyloxybenzoic	610.1932 (+)	18.32 ± 0.28	18.24 ± 0.14	-2.08	610.1919 ± 0.0004	-2.81	610.1915 ± 0.0001
37	Pyridaben	$C_{19}H_{25}ClN_2OS$	Pyridazinone	365.1449 (+)	19.54 ± 0.09	19.51 ± 0.09	-1.64	365.1443 ± 0.0001	-2.24	365.1441 ± 0.0001
38	Pyriproxyfen	$C_{20}H_{19}NO_3$	pyridine-based	322.1438 (+)	17.79 ± 0.29	17.75 ± 0.10	-1.97	322.1432 ± 0.0001	-2.79	322.1429 ± 0.0001
39	Rotenone	$C_{23}H_{22}O_6$	Rotenoid	395.1489 (+)	15.10 ± 0.11	15.05 ± 0.16	-1.98	395.1481 ± 0.0002	-2.78	395.1478 ± 0.0002
40	Azoxystrobin	$C_{22}H_{17}N_3O_5$	Strobilurin	404.1241 (+)	13.49 ± 0.06	13.44 ± 0.25	-1.86	404.1234 ± 0.0001	-2.39	404.1231 ± 0.0001
41	Picoxystrobin	$C_{18}H_{16}F_3NO_4$	Strobilurin	368.1104 (+)	15.54 ± 0.16	15.49 ± 0.16	-1.99	368.1097 ± 0.0002	-2.81	368.1094 ± 0.0002
42	Trifloxystrobin	$C_{20}H_{19}F_3N_2O_4$	Strobilurin	409.137 (+)	17.56 ± 0.33	17.47 ± 0.08	-2.20	409.1361 ± 0.0002	-2.57	409.1360 ± 0.0001
43	Cinosulfuron	$C_{15}H_{19}N_5O_7S$	Sulfonylurea	414.1078 (+)	9.94 ± 0.29	9.91 ± 0.15	-1.93	414.1070 ± 0.0002	-2.54	414.1068 ± 0.0001
				412.0932 (-)	9.70 ± 0.21	9.68 ± 0.09	1.42	412.0938 ± 0.0001	1.29	412.0937 ± 0.0001
44	Metsulfuron methyl	$C_{14}H_{15}N_5O_6S$	Sulfonylurea	382.0816 (+)	11.02 ± 0.21	10.98 ± 0.18	-2.01	382.0808 ± 0.0001	-2.97	382.0805 ± 0.0001
				380.0670 (-)	10.77 ± 0.27	10.75 ± 0.20	0.96	380.0674 ± 0.0001	0.92	380.0674 ± 0.0001
45	Pyrazosulfuron Ethyl	$C_{14}H_{18}N_6O_7S$	Sulfonylurea	415.103 (+)	14.86 ± 0.29	14.77 ± 0.12	-3.49	415.1016 ± 0.0001	-2.41	415.1020 ± 0.0001
				413.0885 (-)	14.59 ± 0.21	14.56 ± 0.24	0.24	413.0886 ± 0.0011	0.93	413.0889 ± 0.0001
46	Fentrazamide	$C_{16}H_{20}ClN_5O_2$	Tetrazolinone	350.1378 (+)	15.77 ± 0.16	15.72 ± 0.18	-1.86	350.1372 ± 0.0001	-2.76	350.1368 ± 0.0001
47	Cyproconazole	$C_{15}H_{18}ClN_3O$	Triazole	292.1211 (+)	12.92 ± 0.09	12.87 ± 0.14	-1.65	292.1206 ± 0.0001	-2.34	292.1204 ± 0.0001
48	Difenoconazole	$C_{19}H_{17}Cl_2N_3O_3$	Triazole	406.0720 (+)	17.11 ± 0.26	17.06 ± 0.10	-1.52	406.0714 ± 0.0001	-2.34	406.0711 ± 0.0001

No	Compound name	Chemical formula	Category	Precursor theorical (polarity)	RT ± rsd, min (n=6)		[M+H] or [M-H] experiment			
					solvent	Match matrix	solvent		Match matrix	
							Mass accuracy	m/z ± SD (n=6)	Mass accuracy	m/z ± SD (n=6)
49	Propiconazole	$C_{15}H_{17}Cl_2N_3O_2$	Triazole	342.0770 (+)	15.49 ± 0.19	15.44 ± 0.15	-1.71	342.0764 ± 0.0001	-2.63	342.0761 ± 0.0002
50	Penoxsulam	$C_{16}H_{14}F_5N_5O_5S$	Triazopyrimidine	484.0708 (+)	11.55 ± 1.12	11.46 ± 0.14	-2.07	484.0698 ± 0.0001	-2.62	484.0695 ± 0.0002
51	Bromadiolone	$C_{30}H_{23}BrO_4$		525.0707 (-)	17.63 ± 0.09	17.63 ± 0.17	0.91	525.0712 ± 0.0001	0.60	525.0710 ± 0.0001
52	Gibberellic acid	$C_{19}H_{22}O_6$		345.1344 (-)	5.55 ± 0.25	5.53 ± 0.15	0.34	345.1345 ± 0.0001	0.34	345.1345 ± 0.00004
53	Spinosad A	$C_{41}H_{65}NO_{10}$		732.4681 (+)	19.27± 0.15	19.25 ± 0.12	-2.30	732.4664 ± 0.0001	-3.32	732.4657 ± 0.0002
	Spinosad D	$C_{42}H_{67}NO_{10}$		746.4838 (+)	19.91 ± 0.13	19.91 ± 0.14	-2.63	746.4818 ± 0.0003	-3.91	746.4809 ± 0.0004

Table S2: Calibration curves and correlation of analytes on the UPLC-Orbitrap Q Exactive MS

No	Compound name	RT ± RSD, min (n=6)		Polarity	Regression equation	Correlation coefficient (R^2)
		solvent	Matrix- match			
1	Fenoxaprop-P-Ethyl	17.70 ± 0.27	17.66 ± 0.09	+	Y = 5.71e6X + 6.487e7	0.9974
2	Fenoxanil	15.12 ± 0.09	15.06 ± 0.15	+	Y = 4.182e6X + 8.905e7	0.9924
3	Chlorantraniliprole	11.5 ± 0.18	11.47 ± 0.15	+	Y = 4.372e5X + 8.847e5	0.9996
4	Fluopicolide	13.40 ± 0.05	13.35 ± 0.21	+	Y = 1.116e7X + 9.527e7	0.9937
5	Chlorfluazuron	20.34 ± 0.09	20.34 ± 0.09	+	Y = 4.039e5X – 7.491e6	0.9996
		20.18 ± 0.16	20.18 ± 0.08	-	Y = 2.407e5X + 5.281e6	0.9966
6	Diflubenzuron	14.99 ± 0.11	14.93 ± 0.12	+	Y = 3.384e5X + 1.912e6	0.9969
		14.76 ± 0.13	14.73 ± 0.24	-	Y = 2.117e5X + 6.647e6	0.9974
7	Carbaryl	9.47 ± 0.28	9.43 ± 0.10	+	Y = 2.276e6X + 4.913e7	0.9976
8	Carbofuran	8.03 ± 0.18	7.99 ± 0.17	+	Y = 1.557e7X + 8.567e7	0.9989
9	Methomyl	4.58 ± 0.23	4.27 ± 0.25	+	Y = 8.082e5X + 3.096e6	0.9987
10	Niclosamide	18.67 ± 0.14	18.67 ± 0.13	-	Y = 1.872e6X + 1.266e7	0.9968
11	Tebufenozide	14.68 ± 0.14	14.63 ± 0.18	+	Y = 1.234e6X + 1.836e7	0.9943
		14.48 ± 0.16	14.45 ± 0.25	-	Y = 1.501e5X + 2.343e6	0.9913
12	Pendimethalin	18.64 ± 0.21	18.58 ± 0.13	+	Y = 6.565e5X – 6.168e6	0.9987
13	Prochloraz	16.34 ± 0.14	16.28 ± 0.12	+	Y = 1.816e6X + 2.296e7	0.9927
14	Clomazone	11.44 ± 0.20	11.40 ± 0.13	+	Y = 6.398e6X + 5.009e7	0.9974
15	Dinotefuran	3.87 ± 0.19	3.72 ± 0.43	-	Y = 3.015e5X + 3.434e6	0.9983
16	Imidacloprid	5.76 ± 0.26	5.74 ± 0.09	+	Y = 2.356e6X + 1.18e7	0.9972
17	Pentachlorophenol	17.50 ± 0.08	17.49 ± 0.16	-	Y = 1.402e5X + 6.331e7	0.9954
18	Diazinon	15.40 ± 0.15	15.35 ± 0.16	+	Y = 1.294e7X + 4.064e8	0.9951
19	Dimethoate	5.67 ± 0.30	5.65 ± 0.13	+	Y = 1.716e7X + 4.219e8	0.9955
20	Phenthoate	15.25 ± 0.14	15.20 ± 0.15	+	Y = 3.72e6X + 6.293e7	0.9943
21	Profenofos	16.93 ± 0.18	16.89 ± 0.07	+	Y = 1.938e6X + 3.319e7	0.9976
22	Quinalphos	14.97 ± 0.12	14.92 ± 0.14	+	Y = 5.374e6X + 6.676e7	0.9974
23	Triazophos	13.93 ± 0.12	13.88 ± 0.20	+	Y = 2.091e7X + 8.225e8	0.9904
24	Trichlorfon	5.20 ± 0.24	5.18 ± 0.14	+	Y = 1.288e6X + 2.828e6	0.9985
25	Dichlorvos	8.19 ± 0.20	8.15 ± 0.14	+	Y = 6.509e5X – 2.636e6	0.9999
26	Parathion-methyl	6.56 ± 0.28	6.56 ± 0.20	-	Y = 4.215e4X + 7.874e5	0.9977
27	Indoxacarb	18.15 ± 0.26	18.10 ± 0.14	+	Y = 7.002e5X – 2.547e6	1.0000
28	Oxadiargyl	15.97 ± 0.18	15.92 ± 0.16	+	Y = 2.326e5X – 4.047e5	0.9996
29	Oxadiazon	17.91 ± 0.18	17.86 ± 0.10	+	Y = 4.14e5X – 1.557e6	0.9999
30	Linuron	12.77 ± 0.14	12.71 ± 0.11	+	Y = 1.362e6X + 2.263e7	0.9981
31	Diuron	11.28 ± 0.20	11.23 ± 0.13	+	Y = -1.915e2e2X^2 + 4.166e6X + 5.552e8	0.9991
		11.03 ± 0.23	11.01 ± 0.28	-	Y = 6.225e4X + 8.543e6	0.9958
32	Fipronil	16.39 ± 0.16	16.38 ± 0.16	-	Y = 1.314e6X + 2.509e7	0.9937
33	Fludioxonil	13.98 ± 0.18	13.96 ± 0.20	-	Y = 2.602e6X + 8.479e7	0.9943
34	Fenitrothion	13.38 ± 0.07	13.32 ± 0.28	+	Y = 2.313e4X + 5.448e5	0.9945
35	Pyridalyl	22.23 ± 0.04	22.26 ± 0.17	+	Y = 1.019e6X + 1.496e6	0.9995
36	Pyribenzoxim	18.32 ± 0.28	18.24 ± 0.14	+	Y = 1.107e6X + 4.023e6	0.9992
37	Pyridaben	19.54 ± 0.09	19.51 ± 0.09	+	Y = 2.855e6X + 6.367e6	0.9993
38	Pyriproxyfen	17.79 ± 0.29	17.75 ± 0.10	+	Y = 8.016e6X + 3.136e7	0.9980
39	Rotenone	15.10 ± 0.11	15.05 ± 0.16	+	Y = 1.365e6X + 9.923e6	0.9969
40	Azoxystrobin	13.49 ± 0.06	13.44 ± 0.25	+	Y = 1.503e7X + 8.688e7	0.9980
41	Picoxystrobin	15.54 ± 0.16	15.49 ± 0.16	+	Y = 3.452e6X + 3.184e7	0.9987
42	Trifloxystrobin	17.56 ± 0.33	17.47 ± 0.08	+	Y = 7.643e6X + 9.536e7	0.9968
43	Cinosulfuron	9.94 ± 0.29	9.91 ± 0.15	+	Y = 2.929e6X – 4.774e6	0.9902
		9.70 ± 0.21	9.68 ± 0.09	-	Y = 4.787e4X + 3.746e5	0.9971
44	Metsulfuron methyl	11.02 ± 0.21	10.98 ± 0.18	+	Y = 1.003e6X – 1.197e6	0.9997
		10.77 ± 0.27	10.75 ± 0.20	-	Y = 3.095e4X + 2.316e7	0.9986
45	Pyrazosulfuron Ethyl	14.86 ± 0.29	14.77 ± 0.12	+	Y = 7.7e5X – 2.245e7	0.9984
		14.59 ± 0.21	14.56 ± 0.24	-	Y = 9.211e0X^2 - 6.743e3X + 5.705e5	0.9960
46	Fentrazamide	15.77 ± 0.16	15.72 ± 0.18	+	Y = 3.233e6X + 1.416e7	0.9960
47	Cyproconazole	12.92 ± 0.09	12.87 ± 0.14	+	Y = 3.601e6X + 1.177e7	0.9997
48	Difenoconazole	17.11 ± 0.26	17.06 ± 0.10	+	Y = 1.038e6X + 2.965e8	0.9957
49	Propiconazole	15.49 ± 0.19	15.44 ± 0.15	+	Y = 7.542e5X + 1.068e7	0.9905

No	Compound name	RT ± RSD, min (n=6)		Polarity	Regression equation	Correlation coefficient (R^2)
		solvent	Matrix- match			
50	Penoxsulam	11.55 ± 1.12	11.46 ± 0.14	+	Y = 1.773e6X + 8.768e5	0.9996
51	Bromadiolone	17.63 ± 0.09	17.63 ± 0.17	-	Y = 3.446e5X + 5.128e7	0.9926
52	Gibberellic acid	5.55 ± 0.25	5.53 ± 0.15	-	Y = 5.568e4X + 1.06e6	0.9994
53	Spinosad A	19.27± 0.15	19.25 ± 0.12	+	Y = 2.227e6X + 1.639e7	0.9967
	Spinosad D	19.91 ± 0.13	19.91 ± 0.14	+	Y = 7.59e5X – 9.484e6	0.9993

Table S3. Analytical figures of merit of the UPLC- Q Exactive Orbitrap MS method for pesticide analysis.

No.	Compound name	Precursor theorical (polarity)	RT ± RSD, min (n=6)		ng/g		pg	
			sovent	Match matrix	LOD	LOQ	MDL*	MQL*
1	Fenoxaprop-P-Ethyl	362.0790 (+)	17.70 ± 0.27	17.66 ± 0.09	0.04	0.13	0.2	0.7
2	Fenoxanil	329.0818 (+)	15.12 ± 0.09	15.06 ± 0.15	0.08	0.27	0.4	1.3
3	Chlorantraniliprole	481.9781 (+)	11.5 ± 0.18	11.47 ± 0.15	0.21	0.70	1.1	3.5
4	Fluopicolide	382.9727 (+)	13.40 ± 0.05	13.35 ± 0.21	0.02	0.07	0.1	0.3
5	Chlorfluazuron	539.9702 (+)	20.34 ± 0.09	20.34 ± 0.09	0.19	0.63	1.0	3.2
		537.9557 (-)	20.18 ± 0.16	20.18 ± 0.08	0.07	0.23	0.4	1.2
6	Diflubenzuron	311.0393 (+)	14.99 ± 0.11	14.93 ± 0.12	0.17	0.57	0.9	2.8
		309.0248 (-)	14.76 ± 0.13	14.73 ± 0.24	0.36	1.20	1.8	6.0
7	Carbaryl	202.0862 (+)	9.47 ± 0.28	9.43 ± 0.10	0.26	0.87	1.3	4.3
8	Carbofuran	222.1125 (+)	8.03 ± 0.18	7.99 ± 0.17	0.09	0.30	0.5	1.5
9	Methomyl	163.0536 (+)	4.58 ± 0.23	4.27 ± 0.25	0.43	1.43	2.2	7.2
10	Niclosamide	324.9788 (-)	18.67 ± 0.14	18.67 ± 0.13	0.04	0.13	0.2	0.7
11	Tebufenozide	353.2224 (+)	14.68 ± 0.14	14.63 ± 0.18	0.11	0.37	0.6	1.8
		351.2078 (-)	14.48 ± 0.16	14.45 ± 0.25	0.13	0.43	0.7	2.2
12	Pendimethalin	282.1448 (+)	18.64 ± 0.21	18.58 ± 0.13	0.36	1.20	1.8	6.0
13	Prochloraz	376.0381 (+)	16.34 ± 0.14	16.28 ± 0.12	0.25	0.83	1.3	4.2
14	Clomazone	240.0786 (+)	11.44 ± 0.20	11.40 ± 0.13	0.10	0.33	0.5	1.7
15	Dinotefuran	201.0993 (-)	3.87 ± 0.19	3.72 ± 0.43	0.24	0.80	1.2	4.0
16	Imidacloprid	256.0596 (+)	5.76 ± 0.26	5.74 ± 0.09	0.86	2.87	4.3	14.3
17	Pentachlorophenol	262.8397 (-)	17.50 ± 0.08	17.49 ± 0.16	0.34	1.13	1.7	5.7
18	Diazinon	305.1083 (+)	15.40 ± 0.15	15.35 ± 0.16	0.05	0.17	0.3	0.8
19	Dimethoate	230.0069 (+)	5.67 ± 0.30	5.65 ± 0.13	0.17	0.57	0.9	2.8
20	Phenthoate	321.0379 (+)	15.25 ± 0.14	15.20 ± 0.15	0.05	0.17	0.3	0.8
21	Profenofos	372.9424 (+)	16.93 ± 0.18	16.89 ± 0.07	0.15	0.50	0.8	2.5
22	Quinalphos	299.0614 (+)	14.97 ± 0.12	14.92 ± 0.14	0.05	0.17	0.3	0.8
23	Triazophos	314.0723 (+)	13.93 ± 0.12	13.88 ± 0.20	0.01	0.03	0.1	0.2
24	Trichlorfon	256.9298 (+)	5.20 ± 0.24	5.18 ± 0.14	0.21	0.70	1.1	3.5
25	Dichlorvos	220.9532 (+)	8.19 ± 0.20	8.15 ± 0.14	0.25	0.83	1.3	4.2
26	Parathion-methyl	261.9944 (-)	6.56 ± 0.28	6.56 ± 0.20	0.28	0.93	1.4	4.7
27	Indoxacarb	528.078 (+)	18.15 ± 0.26	18.10 ± 0.14	0.10	0.33	0.5	1.7
28	Oxadiargyl	341.0454 (+)	15.97 ± 0.18	15.92 ± 0.16	0.23	0.77	1.2	3.8
29	Oxadiazon	345.0767 (+)	17.91 ± 0.18	17.86 ± 0.10	0.13	0.43	0.7	2.2
30	Linuron	249.0192 (+)	12.77 ± 0.14	12.71 ± 0.11	0.18	0.60	0.9	3.0
31	Diuron	233.0243 (+)	11.28 ± 0.20	11.23 ± 0.13	0.15	0.50	0.8	2.5
		231.0097 (-)	11.03 ± 0.23	11.01 ± 0.28	0.15	0.50	0.8	2.5
32	Fipronil	434.9314 (-)	16.39 ± 0.16	16.38 ± 0.16	0.03	0.10	0.2	0.5
33	Fludioxonil	247.0324 (-)	13.98 ± 0.18	13.96 ± 0.20	0.03	0.10	0.2	0.5
34	Fenitrothion	278.0246 (+)	13.38 ± 0.07	13.32 ± 0.28	0.81	2.70	4.1	13.5
35	Pyridalyl	489.9753 (+)	22.23 ± 0.04	22.26 ± 0.17	0.21	0.70	1.1	3.5
36	Pyribenzoxim	610.1932 (+)	18.32 ± 0.28	18.24 ± 0.14	0.12	0.40	0.6	2.0
37	Pyridaben	365.1449 (+)	19.54 ± 0.09	19.51 ± 0.09	0.09	0.30	0.5	1.5
38	Pyriproxyfen	322.1438 (+)	17.79 ± 0.29	17.75 ± 0.10	0.05	0.17	0.3	0.8
39	Rotenone	395.1489 (+)	15.10 ± 0.11	15.05 ± 0.16	0.46	1.53	2.3	7.7
40	Azoxystrobin	404.1241 (+)	13.49 ± 0.06	13.44 ± 0.25	0.02	0.07	0.1	0.3
41	Picoxystrobin	368.1104 (+)	15.54 ± 0.16	15.49 ± 0.16	0.09	0.30	0.5	1.5
42	Trifloxystrobin	409.137 (+)	17.56 ± 0.33	17.47 ± 0.08	0.03	0.10	0.2	0.5
43	Cinosulfuron	414.1078 (+)	9.94 ± 0.29	9.91 ± 0.15	0.43	1.43	2.2	7.2
		412.0932 (-)	9.70 ± 0.21	9.68 ± 0.09	0.25	0.83	1.3	4.2
44	Metsulfuron methyl	382.0816 (+)	11.02 ± 0.21	10.98 ± 0.18	0.13	0.43	0.7	2.2
		380.067 (-)	10.77 ± 0.27	10.75 ± 0.20	0.85	2.83	4.3	14.2
45	Pyrazosulfuron Ethyl	415.103 (+)	14.86 ± 0.29	14.77 ± 0.12	0.21	0.70	1.1	3.5
		413.0885 (-)	14.59 ± 0.21	14.56 ± 0.24	0.08	0.27	0.4	1.3
46	Fentrazamide	350.1378 (+)	15.77 ± 0.16	15.72 ± 0.18	0.10	0.33	0.5	1.7
47	Cyproconazole	292.1211 (+)	12.92 ± 0.09	12.87 ± 0.14	0.48	1.60	2.4	8.0
48	Difenoconazole	406.0720 (+)	17.11 ± 0.26	17.06 ± 0.10	0.26	0.87	1.3	4.3
49	Propiconazole	342.0770 (+)	15.49 ± 0.19	15.44 ± 0.15	0.35	1.17	1.8	5.8

No.	Compound name	Precursor theorical (polarity)	RT ± RSD, min (n=6)		ng/g		pg	
			sovent	Match matrix	LOD	LOQ	MDL*	MQL*
50	Penoxsulam	484.0708 (+)	11.55 ± 1.12	11.46 ± 0.14	0.03	0.10	0.2	0.5
51	Bromadiolone	525.0707 (-)	17.63 ± 0.09	17.63 ± 0.17	0.10	0.33	0.5	1.7
52	Gibberellic acid	345.1344 (-)	5.55 ± 0.25	5.53 ± 0.15	0.69	2.30	3.5	11.5
53	Spinosad A	732.4681 (+)	19.27± 0.15	19.25 ± 0.12	0.25	0.83	1.3	4.2
	Spinosad D	746.4838 (+)	19.91 ± 0.13	19.91 ± 0.14	0.24	0.80	1.2	4.0

** absolute MDL and MQL are defined as amount of analyte that injected into LC column*

Table S4a: Matrix effect, recovery of extraction and overall recovery of pesticides in pooled samples

No	Compound name	Category	Precursor theorical (polarity)	RT ± RSD, min (n=6)		ME ± RSD, (n=6)	RE ± RSD, (n=6)	R ± RSD, (n=6)
				solvent	Matched matrix		%	
1	Fenoxaprop-P-Ethyl	aryloxyphenoxypropionate	362.0790 (+)	17.70 ± 0.27	17.66 ± 0.09	168.9 ± 7.3	67.4 ± 16.3	113.9 ± 17.9
2	Fenoxanil	amide	329.0818 (+)	15.12 ± 0.09	15.06 ± 0.15	136.3 ± 9.3	67.1 ± 13.5	91.5 ± 16.5
3	Chlorantraniliprole	anthranilic diamide	481.9781 (+)	11.5 ± 0.18	11.47 ± 0.15	114.7 ± 6.6	87.3 ± 4.4	99.9 ± 7.8
4	Fluopicolide	benzamide pyridine	382.9727 (+)	13.40 ± 0.05	13.35 ± 0.21	136.2 ± 12.8	63.5 ± 16.2	86.5 ± 20.6
5	Chlorfluazuron	benzoylurea	539.9702 (+)	20.34 ± 0.09	20.34 ± 0.09	179.9 ± 6.2	76.7 ± 8.3	137.8 ± 10.4
			537.9557 (-)	20.18 ± 0.16	20.18 ± 0.08	89.9 ± 8.1	78.8 ± 9.8	70.8 ± 12.7
6	Diflubenzuron	benzoylurea	311.0393 (+)	14.99 ± 0.11	14.93 ± 0.12	163.4 ± 10.2	66.7 ± 7	109± 12.3
			309.0248 (-)	14.76 ± 0.13	14.73 ± 0.24	93.7 ± 6.5	82.9 ± 7.8	77.7 ± 10.2
7	Carbaryl	carbamate	202.0862 (+)	9.47 ± 0.28	9.43 ± 0.10	118.8 ± 4.6	57.8 ± 22.1	78.5 ± 22.6
8	Carbofuran	carbamate	222.1125 (+)	8.03 ± 0.18	7.99 ± 0.17	97.1 ± 1.6	89.9 ± 7.4	87.3 ± 7.5
9	Methomyl	carbamate	163.0536 (+)	4.58 ± 0.23	4.27 ± 0.25	73.6 ± 7.2	69.8 ± 20.2	57.8 ± 21.5
10	Niclosamide	chloronitrophenol	324.9788 (-)	18.67 ± 0.14	18.67 ± 0.13	104.9 ± 7.3	78.2 ± 5.7	82 ± 9.1
11	Tebufenozide	diacylhydrazine	353.2224 (+)	14.68 ± 0.14	14.63 ± 0.18	121.1 ± 5.3	74.8 ± 15.9	90.6 ± 16.7
			351.2078 (-)	14.48 ± 0.16	14.45 ± 0.25	109.4 ± 7.3	83.1 ± 6.8	90.9 ± 10.0
12	Pendimethalin	dinitroaniline	282.1448 (+)	18.64 ± 0.21	18.58 ± 0.13	92.9 ± 9.6	74.5 ± 18.2	69.2 ± 20.6
13	Prochloraz	imidazole	376.0381 (+)	16.34 ± 0.14	16.28 ± 0.12	123.7 ± 2.9	70.8 ± 7.4	87.6 ± 8.0
14	Clomazone	isoxazolidinone	240.0786 (+)	11.44 ± 0.20	11.40 ± 0.13	120.4 ± 6.5	62.3 ± 20.5	84.1 ± 21.5
15	Dinotefuran	neonicotinoid	201.0993 (-)	3.87 ± 0.19	3.72 ± 0.43	54.9 ± 5.5	73.5 ± 16.1	40.4 ± 17.1
16	Imidacloprid	neonicotinoid	256.0596 (+)	5.76 ± 0.26	5.74 ± 0.09	100.4 ± 1.8	76.8 ± 10.1	77.1 ± 10.3
17	Pentachlorophenol	organochlorin- POPs (persistent Organic pollutants)	262.8397 (-)	17.50 ± 0.08	17.49 ± 0.16	98.3 ± 6.1	76.4 ± 7.3	75.1 ± 9.5
18	Diazinon	organophosphate	305.1083 (+)	15.40 ± 0.15	15.35 ± 0.16	149.9 ± 12.9	73.8 ± 17.8	105.6 ± 22.0
19	Dimethoate	organophosphate	230.0069 (+)	5.67 ± 0.30	5.65 ± 0.13	58.9 ± 6.4	64.4 ± 15.6	37.9 ± 16.8
20	Phenthoate	organophosphate	321.0379 (+)	15.25 ± 0.14	15.20 ± 0.15	149.1 ± 4.4	64.6 ± 2.7	96.3 ± 5.0
21	Profenofos	organophosphate	372.9424 (+)	16.93 ± 0.18	16.89 ± 0.07	182.8 ± 6.5	92.1 ± 2.6	168.3 ± 7.0
22	Quinalphos	organophosphate	299.0614 (+)	14.97 ± 0.12	14.92 ± 0.14	129.5 ± 9.5	69.7 ± 9.3	90.3 ± 13.3
23	Triazophos	organophosphate	314.0723 (+)	13.93 ± 0.12	13.88 ± 0.20	140.8 ± 4.5	51.0 ± 12.1	73 ± 12.9
24	Trichlorfon	organophosphate	256.9298 (+)	5.20 ± 0.24	5.18 ± 0.14	147.9 ± 2.9	90.6 ± 2.5	134.1 ± 3.9
25	Dichlorvos	organophosphate	220.9532 (+)	8.19 ± 0.20	8.15 ± 0.14	122.9 ± 2.5	73.4 ± 6.5	90.2 ± 7.0
26	Parathion-methyl	organophosphate	261.9944 (-)	6.56 ± 0.28	6.56 ± 0.20	82.4 ± 4.5	81.6 ± 9.4	67.2 ± 10.4
27	Indoxacarb	oxadiazine	528.0780 (+)	18.15 ± 0.26	18.10 ± 0.14	123.4 ± 5.6	84.9 ± 4.5	104.8 ± 7.1
28	Oxadiargyl	oxidiazole	341.0454 (+)	15.97 ± 0.18	15.92 ± 0.16	177.4 ± 7.9	77.7 ± 10.3	137.8 ± 13.0
29	Oxadiazon	oxidiazole	345.0767 (+)	17.91 ± 0.18	17.86 ± 0.10	151.5 ± 4.7	84.5 ± 2.6	128.3 ± 5.4
30	Linuron	phenylurea	249.0192 (+)	12.77 ± 0.14	12.71 ± 0.11	151.9 ± 11.7	66.9 ± 11.6	100.9 ± 16.4
31	Diuron	phenylurea	233.0243 (+)	11.28 ± 0.20	11.23 ± 0.13	88.5 ± 22.6	93.6 ± 16.5	86.9 ± 28.0
			231.0097 (-)	11.03 ± 0.23	11.01 ± 0.28	119 ± 9.1	76.1 ± 5.9	90.6 ± 10.8
32	Fipronil	phenylpyrazole	434.9314 (-)	16.39 ± 0.16	16.38 ± 0.16	105.5 ± 9.2	82.7 ± 8.2	87.1 ± 12.3
33	Fludioxonil	phenylpyrazole	247.0324 (-)	13.98 ± 0.18	13.96 ± 0.20	104.5 ± 7.8	78.1 ± 6	81.5 ± 9.8
34	Fenitrothion	phosphorothioate	278.0246 (+)	13.38 ± 0.07	13.32 ± 0.28	164.8 ± 14.7	60.4 ± 8.6	99.6 ± 16.9

No	Compound name	Category	Precursor theorical (polarity)	RT ± RSD, min (n=6)		ME ± RSD, (n=6)	RE ± RSD, (n=6)	R ± RSD, (n=6)
				solvent	Matched matrix	%		
35	Pyridalyl	pyridalyl	489.9753 (+)	22.23 ± 0.04	22.26 ± 0.17	123.9 ± 4.5	73.8 ± 11.4	91.5 ± 12.3
36	Pyribenzoxim	pyrimidinyloxybenzoic	610.1932 (+)	18.32 ± 0.28	18.24 ± 0.14	88.5 ± 2.7	78.9 ± 3.7	69.8 ± 4.6
37	Pyridaben	pyridazinone	365.1449 (+)	19.54 ± 0.09	19.51 ± 0.09	121 ± 8.7	72.4 ± 10.8	87.5 ± 13.8
38	Pyriproxyfen	pyridine-based	322.1438 (+)	17.79 ± 0.29	17.75 ± 0.10	137.2 ± 12.2	66.4 ± 13.8	91 ± 18.3
39	Rotenone	rotenoid	395.1489 (+)	15.10 ± 0.11	15.05 ± 0.16	152.8 ± 4.4	82.6 ± 7.2	126.5 ± 8.5
40	Azoxystrobin	strobilurin	404.1241 (+)	13.49 ± 0.06	13.44 ± 0.25	140.2 ± 13.8	60.8 ± 11.4	85 ± 17.9
41	Picoxystrobin	strobilurin	368.1104 (+)	15.54 ± 0.16	15.49 ± 0.16	130.1 ± 3.3	73.2 ± 8.9	95.1 ± 9.5
42	Trifloxystrobin	strobilurin	409.1370 (+)	17.56 ± 0.33	17.47 ± 0.08	127.9 ± 14.8	76.5 ± 9.7	97.7 ± 17.7
43	Cinosulfuron	sulfonylurea	414.1078 (+)	9.94 ± 0.29	9.91 ± 0.15	292.8 ± 4.3	68.1 ± 7.1	199.2 ± 8.3
			412.0932 (-)	9.70 ± 0.21	9.68 ± 0.09	196.4 ± 9.4	68.2 ± 8.6	134.1 ± 12.7
44	Metsulfuron methyl	sulfonylurea	382.0816 (+)	11.02 ± 0.21	10.98 ± 0.18	157.3 ± 10.4	79.1 ± 7.7	124.5 ± 12.9
			380.0670 (-)	10.77 ± 0.27	10.75 ± 0.20	122.7 ± 8.2	76.5 ± 7.3	93.8 ± 11
45	Pyrazosulfuron Ethyl	sulfonylurea	415.1030 (+)	14.86 ± 0.29	14.77 ± 0.12	218.4 ± 1.1	93 ± 1.9	203.1 ± 2.2
			413.0885 (-)	14.59 ± 0.21	14.56 ± 0.24	190.1 ± 2.3	91.8 ± 1.5	174.6 ± 2.7
46	Fentrazamide	tetrazolinone	350.1378 (+)	15.77 ± 0.16	15.72 ± 0.18	155.5 ± 11.7	66.2 ± 17.3	102.8 ± 20.8
47	Cyproconazole	triazole	292.1211 (+)	12.92 ± 0.09	12.87 ± 0.14	142.5 ± 10.1	66.6 ± 18.7	94.9 ± 21.2
48	Difenoconazole	triazole	406.0720 (+)	17.11 ± 0.26	17.06 ± 0.10	208 ± 8.3	73.1 ± 11.6	152.2 ± 14.2
49	Propiconazole	triazole	342.0770 (+)	15.49 ± 0.19	15.44 ± 0.15	114.8 ± 14	72.5 ± 12.5	83.2 ± 18.8
50	Penoxsulam	triazopyrimidine	484.0708 (+)	11.55 ± 1.12	11.46 ± 0.14	175.9 ± 2.5	75.9 ± 13.5	131.5 ± 13.7
51	Bromadiolone		525.0707 (-)	17.63 ± 0.09	17.63 ± 0.17	196.5 ± 6.1	79.3 ± 7	155.8 ± 9.3
52	Gibberellic acid		345.1344 (-)	5.55 ± 0.25	5.53 ± 0.15	50 ± 9.1	78.9 ± 14.4	39.4 ± 17.0
53	Spinosad A		732.4681 (+)	19.27± 0.15	19.25 ±0.12	113.3 ± 6.3	76.1 ± 11	86.2 ± 12.7
	Spinosad D		746.4838 (+)	19.91 ± 0.13	19.91 ± 0.14	104.7 ± 8.5	84.6 ± 8.8	88.5 ± 12.2

Table S4b: Matrix effect, recovery of extraction and overall recovery of pesticides in three vegetable matrices (cabbage, white mustard and edible chrysanthemum)

No	Compound name	Category	[M ± H] theorical (Polarity)	Cabbage			Edible chrysanthemum			White mustard		
				ME ± RSD	RE ± RSD	R ± RSD	ME ± RSD	RE ± RSD	R ± RSD	ME ± RSD	RE ± RSD	R ± RSD
				(n=3), %								
1	Fenoxaprop-P-Ethyl	aryloxyphesnoxypropionate	362.0790 (+)	288.9 ± 4.06	52.5 ± 29.1	151.6 ± 29.1	256.7 ± 25.3	79.7 ± 7.84	204.6 ± 7.84	227.1 ± 4.00	82.2 ± 8.15	186.6 ± 8.15
2	Fenoxanil	amide	329.0818 (+)	165.3 ± 6.01	83.3 ± 0.13	137.6 ± 0.13	155.3 ± 13.8	78.8 ± 5.97	122.4 ± 5.97	137.9 ± 5.70	86.7 ± 2.60	119.5 ± 2.60
3	Chlorantraniliprole	anthranilic diamide	481.9781 (+)	189.0 ± 0.98	80.1 ± 0.64	151.3 ± 0.64	177.8 ± 10.8	83.9 ± 3.71	149.1 ± 3.71	97.2 ± 5.52	108 ± 8.92	105.4 ± 8.92
4	Fluopicolide	benzamide pyridine	382.9727 (+)	226.0 ± 6.64	69.0 ± 2.64	155.9 ± 2.64	184.3 ± 1.31	92.5 ± 4.76	170.5 ± 4.76	132.1 ± 15.9	97.5 ± 1.03	128.8 ± 1.03
5	Chlorfluazuron	benzoylurea	539.9702 (+)	206.9 ±19.6	49.2 ± 7.94	101.7 ± 7.94	126.7 ± 14.1	86.2 ± 0.26	109.3 ± 0.26	161.8 ± 4.09	84.0 ± 2.46	135.9 ± 2.46
6	Diflubenzuron	benzoylurea	311.0393 (+)	164.1 ± 8.65	70.1 ± 9.12	115.1 ± 9.12	191.9 ± 3.99	72.9 ± 7.95	140.0 ± 7.95	97.1 ± 4.56	111 ± 4.28	108.1 ± 4.28
7	Carbaryl	carbamate	202.0862 (+)	152.3 ± 3.60	81.1 ± 12.7	123.5 ± 12.7	123.6 ± 0.93	88.0 ± 3.85	108.7 ± 3.85	75.8 ± 18.5	112 ± 12.1	85.1 ± 12.1
8	Carbofuran	carbamate	222.1125 (+)	131.0 ± 0.94	75.9 ± 10.2	99.4 ± 10.2	166.3 ± 13.9	81.4 ± 1.85	135.4 ± 1.85	82.1 ± 4.20	104 ± 0.71	85.4 ± 0.71
9	Methomyl	carbamate	163.0536 (+)	149.2 ± 5.38	65.5 ± 16.4	97.7 ± 16.4	105.2 ± 10.9	97.6 ± 3.51	102.6 ± 3.51	94.2 ± 21.1	98.8 ± 4.02	93.1 ± 4.02
11	Tebufenozide	diacylhydrazine	353.2224 (+)	264.6 ± 11.2	67.6 ± 24.5	179.0 24.5	209.2 ± 7.50	100 ± 6.01	210.0 ± 6.01	187.4 ± 7.11	83.7 ± 6.01	156.9 ± 6.01
12	Pendimethalin	dinitroaniline	282.1448 (+)	263.4 ± 6.60	58.1 ± 19.5	153.1 ± 19.5	258.0 ± 0.19	78.5 ± 9.69	202.5 ± 9.69	251.8 ± 23.4	79.4 ± 0.70	199.9 ± 0.70
13	Prochloraz	imidazole	376.0381 (+)	263.6 ± 2.87	54.4 ± 25.1	143.4 ± 25.1	203.4 ± 1.05	77.8 ± 0.96	158.3 ± 0.96	202.2 ± 11.4	88.7 ± 7.29	179.2 ± 7.29
14	Clomazone	isoxazolidinone	240.0786 (+)	192.3 ± 4.95	67.2 ± 1.25	129.3 ± 11.3	145.2 ± 5.44	92.9 ± 11.3	134.9 ± 11.3	92.5 ± 1.08	117 ± 2.24	108.3 ± 2.24
16	Imidacloprid	neonicotinoid	256.0596 (+)	115.4 ± 13.2	82.2 ± 14.8	94.9 ± 14.8	99.8 ± 15.2	102 ± 10.0	102.3 ± 10.0	53.11 ± 21.2	111 ± 1.12	59.4 ± 1.12
18	Diazinon	organophosphate	305.1083 (+)	322.6 ± 0.65	54.4 ± 14.8	175.4 ± 14.8	254.2 ± 17.4	89.6 ± 4.41	227.7 ± 4.41	189.3 ± 2.90	86.3 ± 7.96	163.5 ± 7.96
19	Dimethoate	organophosphate	230.0069 (+)	115.2 ± 0.15	60.0 ± 1.66	69.1 ± 1.66	84.1 ± 0.30	96.9 ± 1.90	81.5 ± 1.90	53.95 ± 26.3	103 ± 1.42	55.43 ± 1.42
20	Phenthoate	organophosphate	321.0379 (+)	271.1 ± 7.80	58.5 ± 15.2	158.7 ± 15.2	227.5 ± 10.4	95.6 ± 9.83	217.5 ± 9.83	191.8 ± 15.2	100 ± 3.48	192.3 ± 3.48
21	Profenofos	organophosphate	372.9424 (+)	581.2 ± 8.54	55.2 ± 21.7	321.1 ± 21.7	465.1 ± 20.4	95.0 ± 6.46	441.8 ± 6.46	341.2 ± 38.6	125 ± 1.08	425.9 ± 1.08
22	Quinalphos	organophosphate	299.0614 (+)	265.5 ± 4.85	64.7 ± 22.7	171.7 ± 22.7	245.7 ± 20.2	87.7 ± 9.08	215.5 ± 9.08	157.6 ± 9.58	111 ± 5.30	175.1 ± 5.30
23	Triazophos	organophosphate	314.0723 (+)	356.5 ± 2.56	52.1 ± 16.3	185.6 ± 16.3	305.1 ± 27.7	90.5 ± 15.7	276.1 ± 15.7	163.0 ± 11.3	78.8 ± 15.6	128.4 ± 15.6
24	Trichlorfon	organophosphate	256.9298 (+)	168.8 ± 1.39	86.7 ± 0.04	146.3 ± 0.04	168.9 ± 0.16	101 ± 2.75	170.4 ± 2.75	157.8 ± 6.11	91.9 ± 0.77	145.0 ± 0.77
25	Dichlorvos	organophosphate	220.9532 (+)	120.8 ± 4.85	78.7 ± 0.82	95.0 ± 0.82	99.7 ± 0.64	90.0 ± 0.64	89.7 ± 0.64	109.8 ± 3.06	82.2 ± 2.29	90.3 ± 2.29
27	Indoxacarb	oxadiazine	528.078 (+)	288.6 ± 5.46	58.7 ± 23.3	169.5 ± 23.3	225.5 ± 18.5	92.8 ± 5.86	209.3 ± 5.86	208.8 ± 0.45	92.6 ± 6.78	193.3 ± 6.78
28	Oxadiargyl	oxidiazole	341.0454 (+)	257.6 ± 5.26	67.6 ± 23.7	174.1 ± 23.7	230.4 ± 19.9	92.2 ± 11.2	212.5 ± 11.2	202.5 ± 32.8	94.8 ± 4.34	192.1 ± 4.34
29	Oxadiazon	oxidiazole	345.0767 (+)	320.7 ± 20.6	51.6 ± 23.9	165.6 ± 23.9	220.6 ± 19.6	92.0 ± 12.1	202.9 ± 12.1	214.8 ± 4.50	82.7 ± 10.1	177.6 ± 10.1
30	Linuron	phenylurea	249.0192 (+)	214.1 ± 11.5	72.2 ± 9.5	154.6 ± 9.5	199.1 ± 8.21	81.0 ± 3.94	161.2 ± 3.94	130.7 ± 18.1	89.4 ± 0.10	116.9 ± 0.10

No	Compound name	Category	[M ± H] theorical (Polarity)	Cabbage			Edible chrysanthemum			White mustard		
				ME ± RSD	RE ± RSD	R ± RSD	ME ± RSD	RE ± RSD	R ± RSD	ME ± RSD	RE ± RSD	R ± RSD
								(n=3), %				
31	Diuron	phenylurea	233.0243 (+)	169.9 ± 10.1	78.5 ± 14.9	133.3 ± 14.9	90.9 ± 4.74	93.3 ± 0.07	84.8 ± 0.07	65.25 ± 23.1	113 ± 10.9	74.2 ± 10.9
34	Fenitrothion	phosphorothioate	278.0246 (+)	202.7 ± 8.02	79.8 ± 3.46	161.8 ± 3.46	92.8 54.7	115 ± 17.4	107.2 ± 17.4	134.1 ± 1.92	86.5 ± 7.65	116.0 ± 7.65
35	Pyridalyl	pyridalyl	489.9753 (+)	284.3 ± 23.0	55.8 ± 23.2	158.6 ± 23.2	175.6 ± 19.3	90.9 13.5	159.6 13.5	182.2 ± 29.2	85.6 ± 8.24	156.0 ± 8.24
36	Pyribenzoxim	pyrimidinyloxybenzoic	610.1932 (+)	359.5 ± 27.2	56.6 ± 27.2	203.5 ± 27.2	255.2 ± 25.6	85.7 ± 2.10	218.8 ± 2.10	301.0 ± 1.00	75.5 ± 8.06	227.2 ± 8.06
37	Pyridaben	pyridazinone	365.1449 (+)	336.3 ±28.4	54.3 ± 28.8	182.7 ± 28.8	222.9 ± 29.1	86.8 ± 9.16	193.4 ± 9.16	296.6 ± 10.5	64.9 ± 7.17	192.4 ± 7.17
38	Pymetrozine	pyridine	218.1036 (+)	85.4 ± 44.6	76.5 ± 1.50	65.3 ± 1.50	126.3 ± 23.7	65.4 ± 17.4	82.6 ± 17.4	77.03 ± 14.1	68.1 ± 11.7	52.5 ± 11.7
39	Pyriproxyfen	pyridine-based	322.1438 (+)	302.7 ± 9.04	57.3 ± 23.3	173.5 ± 23.3	238.4 ± 25.6	92.0 ± 13.2	219.3 ± 13.2	236.4 ± 6.09	84.6 ± 8.80	200.0 ± 8.80
40	Rotenone	rotenoid	395.1489 (+)	276.8 ± 10.7	66.2 ± 20.3	183.2 ± 20.3	268.4 ± 17.0	84.4 ± 11.0	226.6 ± 11.0	204.1 ± 7.40	107 ± 2.20	218.6 ± 2.20
41	Azoxystrobin	strobilurin	404.1241 (+)	160.2 ± 18.8	100.2 ± 6.30	160.6 ± 6.24	209.6 ± 1.73	91.3 ± 10.3	191.4 ± 10.3	113.0 ± 0.93	94.3 ± 1.70	106.5 ± 1.70
42	Picoxystrobin	strobilurin	368.1104 (+)	279.0 ± 3.64	60.4 ± 13.4	168.5 ± 13.4	217.6 ± 7.13	82.7 ± 1.18	180.0 ± 1.18	218.7 ± 9.38	86.4 ± 2.53	188.9 ± 2.53
43	Trifloxystrobin	strobilurin	409.137 (+)	286.3 ± 2.83	58.4 ± 28.5	167.2 ± 28.5	230.8 ± 25.8	87.5 ± 6.90	202.0 ± 6.90	284.8 ± 17.5	74.2 ± 5.85	211.2 ± 5.85
44	Cinosulfuron	sulfonylurea	414.1078 (+)	191.1 ± 6.07	83.6 ± 41.4	159.8 ± 41.4	200 ± 36.1	73.7 ± 31.9	147.6 ± 31.9	183.2 ± 0.00	80.0 ± 4.70	146.5 ± 4.70
45	Metsulfuron methyl	sulfonylurea	382.0816 (+)	460.9 ± 2.12	63.8 ± 19.7	294.2 ± 19.7	260.0 ± 7.06	74.8 ± 6.38	194.6 ± 6.38	458.4 ± 17.4	104 ± 9.27	477.2 ± 9.27
46	Pyrazosulfuron Ethyl	sulfonylurea	415.103 (+)	2682 ± 3.73	85.1 ± 0.11	2282 ± 0.11	2953 ± 2.25	93.9 ± 0.16	2771 ± 0.16	2918 ± 2.29	99.6 ± 1.22	2907 ± 1.22
47	Fentrazamide	tetrazolinone	350.1378 (+)	306.4 ± 2.59	60.0 ± 11.7	183.7 ± 11.7	265.1 ± 16.9	82.6 ± 3.41	219.0 ± 3.41	243.2 ± 2.62	73.9 ± 6.37	179.8 ± 6.37
48	Cyproconazole	triazole	292.1211 (+)	193.7 ± 2.81	58.5 ± 7.16	113.3 ± 7.16	173.7 ± 15.3	90.6 ± 9.17	157.4 ± 9.17	107.0 ± 11.2	95.4 ± 3.65	102.1 ± 3.65
49	Difenoconazole	triazole	406.0720 (+)	313.9 ± 3.68	47.4 ± 1.16	148.8 ± 1.16	205.5 ± 15.4	87.2 ± 13.4	179.1 ± 13.4	245.4 ± 4.25	71.9 ± 0.96	176.5 ± 0.96
50	Propiconazole	triazole	342.0770 (+)	194.4 ± 4.69	60.2 ± 10.4	117.1 ± 10.4	161.4 ± 4.01	96.1 ± 4.30	155.1 ± 4.30	125.4 ± 34.3	119 ± 5.98	149.8 ± 5.98
53	Spinosad A		732.4681 (+)	337.9 ± 28.8	47.9 ± 23.4	161.9 ± 23.4	227.3 ± 22.3	91.0 ± 12.2	206.8 ± 12.2	384.1 ± 32.4	46.1 ± 7.66	176.9 ± 7.66
	Spinosad D		746.4838 (+)	319.4 ± 28.2	52.7 ± 26.4	168.3 ± 26.4	224.5 ± 26.2	86.3 ± 13.6	193.7 ± 13.6	391.5 ± 34.9	44.4 ± 8.50	173.8 ± 8.50

Table S5: Mass and mass accuracy of analytical product ions

No	Compound name	Chemical formula	RT ± rsd, min (n=6)	Precursor (Polarity)	Fragment 1				Fragment 2			
			solvent		Formula	m/z theorical	m/z exp	Mass accuracy	Formula	m/z theorical	m/z exp	Mass accuracy
1	Fenoxaprop-P-Ethyl	$C_{18}H_{16}ClNO_5$	17.70 ± 0.27	362.0790 (+)	$C_{15}H_{11}ClNO_3$	288.0427	288.0417	-3.47				
2	Fenoxanil	$C_{15}H_{18}Cl_2N_2O_2$	15.12 ± 0.09	329.0818 (+)	$C_5H_{12}N$	86.0970	86.0968	-2.32	$C_8H_7Cl_2O$	188.9874	188.9866	-4.23
3	Chlorantraniliprole	$C_{18}H_{14}BrCl_2N_5O_2$	11.5 ± 0.18	481.9781 (+)	$C_9H_4BrClN_3O$	283.9226	283.9215	-3.87	$C_{17}H_{10}O_2N_4BrCl_2$	450.9364	450.9351	-2.88
4	Fluopicolide	$C_{14}H_8Cl_3F_3N_2O$	13.40 ± 0.05	382.9727 (+)	$C_7H_3OCl_2$	172.9561	172.9554	-4.05				
5	Chlorfluazuron	$C_{20}H_9Cl_3F_5N_3O_3$	20.34 ± 0.09	539.9702 (+)	$C_{13}H_5Cl_3F_3N_2O_2$	382.9369	382.9355	-3.66	$C_7H_6F_2NO$	158.0417	158.0410	-4.43
			20.18 ± 0.16	537.9557 (-)	$C_9H_6O_2N_2Cl_3F_4$	354.9431	354.9431	0.00	$CO_3N_3ClF_2$	174.9596	174.9591	-2.86
6	Diflubenzuron	$C_{14}H_9ClF_2N_2O_2$	14.99 ± 0.11	311.0393 (+)	$C_7H_3F_2O$	141.0152	141.0144	-5.67	$C_7H_6F_2NO$	158.0417	158.0410	-4.43
			14.76 ± 0.13	309.0248 (-)	$C_6H_3F_2$	113.0203	113.0198	-4.42	$C_7H_4ClN_2$	151.0063	151.0059	-2.65
7	Carbaryl	$C_{12}H_{11}NO_2$	9.47 ± 0.28	202.0862 (+)	$C_{10}H_9O$	145.0653	145.0647	-4.14	C_9H_9	117.0704	117.0700	-3.42
8	Carbofuran	$C_{12}H_{15}NO_3$	8.03 ± 0.18	222.1125 (+)	$C_7H_7O_2$	123.0446	123.0441	-4.06				
9	Methomyl	$C_5H_{10}N_2O_2S$	4.58 ± 0.23	163.0536 (+)	C_3H_6NS	88.0221	88.0219	-2.27	C_3H_8NOS	106.0326	106.0321	-4.72
10	Niclosamide	$C_{13}H_8Cl_2N_2O_4$	18.67 ± 0.14	324.9788 (-)	$C_6H_4ClN_2O_2$	170.9961	170.9961	0.00				
11	Tebufenozide	$C_{22}H_{28}N_2O_2$	14.68 ± 0.14	353.2224 (+)	C_9H_9O	133.0653	133.0647	-4.51				
			14.48 ± 0.16	351.2078 (-)	$C_9H_9O_2$	149.0602	149.0598	-2.68				
12	Pendimethalin	$C_{13}H_{19}N_3O_4$	18.64 ± 0.21	282.1448 (+)	C_6H_5N	91.0422	91.0419	-3.30	$C_7H_7N_2$	119.0609	119.0604	-4.20
13	Prochloraz	$C_{15}H_{16}Cl_3N_3O_2$	16.34 ± 0.14	376.0381 (+)	C_3H_4ON	70.0293	70.0292	-1.43	$C_{12}H_{13}O_2NCl_3$	308.0012	308.0002	-3.25
14	Clomazone	$C_{12}H_{14}ClNO_2$	11.44 ± 0.20	240.0786 (+)	C_7H_6Cl	125.0158	125.0153	-4.00				
15	Dinotefuran	$C_7H_{14}N_4O_2$	3.87 ± 0.19	201.0993 (-)	HN_2O_2	61.0038	61.0031	-11.47				
16	Imidacloprid	$C_9H_{10}ClN_5O_2$	5.76 ± 0.26	256.0596 (+)	$C_9H_{11}N_4$	175.0984	175.0976	-4.57	$C_9H_{10}ClN_4$	209.0594	209.0585	-4.30
17	Pentachlorophenol	C_6HCl_5O	17.50 ± 0.08	262.8397 (-)	C_6OCl_5	262.8392	262.8400	3.04				
18	Diazinon	$C_{12}H_{21}N_2O_3PS$	15.40 ± 0.15	305.1083 (+)	$C_8H_{13}N_2S$	169.0799	169.0792	-4.14	$C_8H_{13}N_2O$	153.1028	153.1021	-4.57
19	Dimethoate	$C_5H_{12}NO_3PS_2$	5.67 ± 0.30	230.0069 (+)	$C_2H_6O_2PS$	124.9826	124.9820	-4.80				
20	Phenthoate	$C_{12}H_{17}O_4PS_2$	15.25 ± 0.14	321.0379 (+)	C_7H_7O	107.0497	107.0493	-3.74	$C_8H_7O_2$	135.0446	135.0439	-5.18
21	Profenofos	$C_{11}H_{15}BrClO_3PS$	16.93 ± 0.18	372.9424 (+)	$C_6H_6O_3BrClPS$	302.8647	302.8635	-3.96				
22	Quinalphos	$C_{12}H_{15}N_2O_3PS$	14.97 ± 0.12	299.0614 (+)	$C_8H_7ON_2$	147.0558	147.0551	-4.76	$C_8H_7N_2S$	163.0330	163.0322	-4.91
23	Triazophos	$C_{12}H_{16}N_3O_3PS$	13.93 ± 0.12	314.0723 (+)	$C_8H_8ON_3$	162.0667	162.0660	-4.32				
24	Trichlorfon	$C_4H_8Cl_3O_4P$	5.20 ± 0.24	256.9298 (+)	$C_2H_8O_4P$	127.0160	127.0154	-4.72				
25	Dichlorvos	$C_4H_7Cl_2O_4P$	8.19 ± 0.20	220.9532 (+)	$C_2H_8O_4P$	127.0160	127.0154	-4.72	CH_4O_2P	78.9949	78.9948	-1.27
26	Parathion-methyl	$C_8H_{10}NO_5PS$	6.56 ± 0.28	261.9944 (-)	$C_7H_6O_3N$	152.0348	152.0343	-3.29	$C_6H_4O_3N$	138.0191	138.0187	-2.90
27	Indoxacarb	$C_{22}H_{17}ClF_3N_3O_7$	18.15 ± 0.26	528.0780 (+)	$C_{15}H_7O_3N$	249.0426	249.0421	-2.01	$C_{13}H_{10}ClN_2O_4$	293.0329	293.0324	-1.71
28	Oxadiargyl	$C_{15}H_{14}Cl_2N_2O_3$	15.97 ± 0.18	341.0454 (+)	C_8H_6NCl	151.0189	151.0181	-5.30	$C_{10}H_5O_3NCl$	221.9958	221.9950	-3.60
29	Oxadiazon	$C_{15}H_{18}Cl_2N_2O_3$	17.91 ± 0.18	345.0767 (+)	$C_6H_3Cl_2O_2$	176.9510	176.9505	-2.83	$C_7H_4Cl_2NO_3$	219.9568	219.9563	-2.27
30	Linuron	$C_9H_{10}Cl_2N_2O_2$	12.77 ± 0.14	249.0192 (+)	$C_6H_4Cl_2N$	159.9721	159.9713	-5.00	$C_8H_7ClN_2O$	182.0247	182.0239	-4.40
31	Diuron	$C_9H_{10}Cl_2N_2O$	11.28 ± 0.20	233.0243 (+)	C_3H_6NO	72.0449	72.0449	0.00				
			11.03 ± 0.23	231.0097 (-)	$C_7H_2Cl_2NO$	185.9513	185.9510	-1.61	C_7HClNO	149.9747	149.9741	-4.00
32	Fipronil	$C_{12}H_4Cl_2F_6N_4OS$	16.39 ± 0.16	434.9314 (-)	$C_8H_2ClF_3N_2S$	249.9579	249.9586	2.80	$C_9H_2ClF_3N_2OS$	277.9528	277.9538	3.60
33	Fludioxonil	$C_{12}H_6F_2N_2O_2$	13.98 ± 0.18	247.0324 (-)	$C_{11}H_4N_2O$	180.0324	180.0324	0.00				
34	Fenitrothion	$C_9H_{12}NO_5PS$	13.38 ± 0.07	278.0246 (+)	$C_2H_8O_3PS$	142.9932	142.9926	-4.20				
35	Pyridalyl	$C_{18}H_{14}Cl_4F_3NO_3$	22.23 ± 0.04	489.9753 (+)	$C_3H_3Cl_2$	108.9612	108.9608	-3.67	$C_6H_5F_3NO$	164.0323	164.0315	-4.88

No	Compound name	Chemical formula	RT ± rsd, min (n=6) solvent	Precursor (Polarity)	Fragment 1 Formula	Fragment 1 m/z theorical	Fragment 1 m/z exp	Fragment 1 Mass accuracy	Fragment 2 Formula	Fragment 2 m/z theorical	Fragment 2 m/z exp	Fragment 2 Mass accuracy
36	Pyribenzoxim	$C_{32}H_{27}N_5O_8$	18.32 ± 0.28	610.1932 (+)	$C_{19}H_{17}N_4O_7$	413.1097	413.1082	-3.63				
37	Pyridaben	$C_{19}H_{25}ClN_2OS$	19.54 ± 0.09	365.1449 (+)	C11H15	147.1174	147.1166	-5.44	$C_{15}H_{18}ClN_2OS$	309.0828	309.0814	-4.53
38	Pyriproxyfen	$C_{20}H_{19}NO_3$	17.79 ± 0.29	322.1438 (+)	C_5H_6NO	96.0449	96.0447	-2.08	$C_{15}H_{15}O_2$	227.1072	227.1065	-3.08
39	Rotenone	$C_{23}H_{22}O_6$	15.10 ± 0.11	395.1489 (+)	$C_{14}H_{13}O_2$	213.0916	213.0906	-4.69	$C_{11}H_{12}O_3$	192.0786	192.0778	-4.16
40	Azoxystrobin	$C_{22}H_{17}N_3O_5$	13.49 ± 0.06	404.1241 (+)	$C_{21}H_{14}N_3O_4$	372.0984	372.0972	-3.22	$C_{20}H_{14}N_3O_3$	344.1035	344.1024	-3.20
41	Picoxystrobin	$C_{18}H_{16}F_3NO_4$	15.54 ± 0.16	368.1104 (+)	$C_{10}H_9O$	145.0653	145.0646	-4.83				
42	Trifloxystrobin	$C_{20}H_{19}F_3N_2O_4$	17.56 ± 0.33	409.137 (+)	$C_9H_7F_3N$	186.0530	186.0522	-4.30				
43	Cinosulfuron	$C_{15}H_{19}N_5O_7S$	9.94 ± 0.29	414.1078 (+)	$C_6H_7N_4O_3$	183.0518	183.0510	-4.37	$C_5H_9N_4O_2$	157.0726	157.0718	-5.09
			9.70 ± 0.21	412.0932 (-)	$C_5H_7O_2N_4$	155.0569	155.0565	-2.58				
44	Metsulfuron methyl	$C_{14}H_{15}N_5O_6S$	11.02 ± 0.21	382.0816 (+)	$C_6H_7N_4O_2$	167.0569	167.0562	-4.19	$C_8H_7O_2$	135.0446	135.0439	-5.18
			10.77 ± 0.27	380.0670 (-)	$C_5H_7O_4$	139.0620	139.0614	-4.31				
45	Pyrazosulfuron Ethyl	$C_{14}H_{18}N_6O_7S$	14.86 ± 0.29	415.1030 (+)	$C_7H_8N_3O_3$	182.0566	182.0558	-4.39				
			14.59 ± 0.21	413.0885 (-)	$C_6H_8N_3O_2$	154.0616	154.0613	-1.95				
46	Fentrazamide	$C_{16}H_{20}ClN_5O_2$	15.77 ± 0.16	350.1378 (+)	C_6H_{11}	83.0861	83.0859	-2.41				
47	Cyproconazole	$C_{15}H_{18}ClN_3O$	12.92 ± 0.09	292.1211 (+)	$C_2H_4N_3$	70.0405	70.0405	0.00	C_7H_6Cl	125.0158	125.0153	-4.00
48	Difenoconazole	$C_{19}H_{17}Cl_2N_3O_3$	17.11 ± 0.26	406.0720 (+)	$C_{13}H_9Cl_2O$	251.0030	251.0020	-3.98				
49	Propiconazole	$C_{15}H_{17}Cl_2N_3O_2$	15.49 ± 0.19	342.0770 (+)	$C_7H_5Cl_2$	158.9768	158.9760	-5.03	C_5H_9	69.0704	69.0704	0.00
50	Penoxsulam	$C_{16}H_{14}F_5N_5O_5S$	11.55 ± 1.12	484.0708 (+)	$C_7H_9O_2N_5$	195.0756	195.0749	-3.59	$C_{16}H_{13}O_5N_5F_3S$	444.0589	444.0572	-3.83
51	Bromadiolone	$C_{30}H_{23}BrO_4$	17.63 ± 0.09	525.0707 (-)	$C_{30}H_{22}BrO_4$	525.0701	525.0712	2.09	$C_{16}H_{10}O_3$	250.0630	250.0637	2.80
52	Gibberellic acid	$C_{19}H_{22}O_6$	5.55 ± 0.25	345.1344 (-)	$C_{11}H_{11}$	143.0861	143.0855	-4.19				
53	Spinosad A	$C_{41}H_{65}NO_{10}$	19.27± 0.15	732.4681 (+)	$C_8H_{16}ON$	142.1232	142.1225	-4.93	$C_{41}H_{65}NO_{10}$	732.4681	732.4667	-1.91
	Spinosad D	$C_{42}H_{67}NO_{10}$	19.91 ± 0.13	746.4838 (+)	$C_8H_{16}ON$	142.1232	142.1225	-4.93	$C_{42}H_{67}NO_{10}$	746.4838	746.4819	-2.55

Table S6. Short- and long-term stability of the analytical signal in standard solution and in matrix-match solution

No.	Name	Category	Polarity	RSD of analytical signal in standard solution		RSD of analytical signal in matrix match solution	
				Short term	Long term	Short term	Long term
1	Fenoxaprop-P-Ethyl	aryloxyphenoxypropionate	362.0790 (+)	1.18	4.41	1.55	4.15
2	Fenoxanil	amide	329.0818 (+)	0.55	6.38	1.22	4.58
3	Chlorantraniliprole	anthranilic diamide	481.9781 (+)	2.78	7.40	3.74	4.70
4	Fluopicolide	benzamide pyridine	382.9727 (+)	0.78	4.94	1.21	4.84
5	Chlorfluazuron	benzoylurea	539.9702 (+)	2.23	7.27	5.66	5.33
			537.9557 (-)	3.16	3.54	1.35	4.70
6	Diflubenzuron	benzoylurea	311.0393 (+)	1.97	5.16	1.88	5.17
			309.0248 (-)	2.04	3.28	1.57	4.54
7	Carbaryl	carbamate	202.0862 (+)	0.38	5.28	1.00	2.02
8	Carbofuran	carbamate	222.1125 (+)	0.70	4.22	0.77	0.99
9	Methomyl	carbamate	163.0536 (+)	0.20	4.18	2.42	4.15
10	Niclosamide	chloronitrophenol	324.9788 (-)	2.37	4.09	3.07	5.22
11	Tebufenozide	diacylhydrazine	353.2224 (+)	1.19	5.37	2.79	2.48
			351.2078 (-)	2.70	4.25	1.60	5.88
12	Pendimethalin	dinitroaniline	282.1448 (+)	9.00	10.39	3.91	4.89
13	Prochloraz	imidazole	376.0381 (+)	0.72	5.30	0.74	1.91
14	Clomazone	isoxazolidinone	240.0786 (+)	2.90	5.60	0.27	2.89
15	Dinotefuran	neonicotinoid	201.0993 (-)	2.98	4.44	2.09	2.97
16	Imidacloprid	neonicotinoid	256.0596 (+)	1.80	9.81	0.56	0.71
17	Pentachlorophenol	organochlorin- POPs (persistent Organic pollutants)	262.8397 (-)	2.19	2.65	0.93	4.32
18	Diazinon	organophosphate	305.1083 (+)	1.53	5.64	3.01	8.84
19	Dimethoate	organophosphate	230.0069 (+)	2.75	5.50	1.50	2.93
20	Phenthoate	organophosphate	321.0379 (+)	1.12	5.13	2.45	2.09
21	Profenofos	organophosphate	372.9424 (+)	0.71	5.42	2.70	6.47
22	Quinalphos	organophosphate	299.0614 (+)	0.66	5.50	5.15	4.26
23	Triazophos	organophosphate	314.0723 (+)	1.26	4.84	1.07	1.65
24	Trichlorfon	organophosphate	256.9298 (+)	1.98	2.08	9.15	8.48
25	Dichlorvos	organophosphate	220.9532 (+)	4.84	5.94	1.55	2.05
26	Parathion-methyl	organophosphate	261.9944 (-)	5.64	5.83	1.35	3.39
27	Indoxacarb	oxadiazine	528.078 (+)	1.37	6.26	6.48	5.46
28	Oxadiargyl	oxidiazole	341.0454 (+)	1.85	6.05	1.81	5.96
29	Oxadiazon	oxidiazole	345.0767 (+)	0.60	5.41	3.91	3.49
30	Linuron	phenylurea	249.0192 (+)	3.14	5.45	4.14	6.84
31	Diuron	phenylurea	233.0243 (+)	0.48	3.71	4.22	8.35
			231.0097 (-)	3.72	5.49	5.63	6.36
32	Fipronil	phenylpyrazole	434.9314 (-)	4.03	5.17	0.48	7.15
33	Fludioxonil	phenylpyrazole	247.0324 (-)	3.36	4.92	1.39	6.12
34	Fenitrothion	phosphorothioate	278.0246 (+)	1.02	5.65	1.99	7.03
35	Pyridalyl	pyridalyl	489.9753 (+)	2.11	6.25	1.01	2.69
36	Pyribenzoxim	pyrimidinyloxybenzoic	610.1932 (+)	0.42	5.47	0.89	1.63
37	Pyridaben	pyridazinone	365.1449 (+)	1.71	5.54	3.68	4.40

38	Pyriproxyfen	pyridine-based	322.1438 (+)	0.80	4.60	3.07	6.11
39	Rotenone	rotenoid	395.1489 (+)	0.53	6.25	0.76	4.42
40	Azoxystrobin	strobilurin	404.1241 (+)	0.96	5.78	1.17	6.31
41	Picoxystrobin	strobilurin	368.1104 (+)	1.80	4.70	1.28	1.67
42	Trifloxystrobin	strobilurin	409.137 (+)	6.61	9.22	2.72	6.92
43	Cinosulfuron	sulfonylurea	414.1078 (+)	0.88	7.63	1.35	3.38
			412.0932 (-)	2.10	2.99	4.22	7.74
44	Metsulfuron methyl	sulfonylurea	382.0816 (+)	1.80	8.30	2.68	4.57
			380.067 (-)	2.15	1.84	1.31	3.55
45	Pyrazosulfuron Ethyl	sulfonylurea	415.103 (+)	1.56	2.89	2.65	2.65
			413.0885 (-)	9.15	13.38	6.36	5.79
46	Fentrazamide	tetrazolinone	350.1378 (+)	1.87	4.87	1.85	6.21
47	Cyproconazole	triazole	292.1211 (+)	0.96	4.65	1.08	5.02
48	Difenoconazole	triazole	406.0720 (+)	2.19	4.72	2.30	5.00
49	Propiconazole	triazole	342.0770 (+)	1.77	5.48	2.96	5.37
50	Penoxsulam	triazopyrimidine	484.0708 (+)	2.34	9.58	1.9	2.04
51	Bromadiolone		525.0707 (-)	8.16	10.21	1.92	3.16
52	Gibberellic acid		345.1344 (-)	0.44	3.57	0.45	5.77
53	Spinosad A		732.4681 (+)	1.53	5.08	3.89	4.39
	Spinosad D		746.4838 (+)	2.90	6.54	3.97	8.47

Fig. S1. The total ion chromatogram of the target analytes in the standard solution (positive mode)

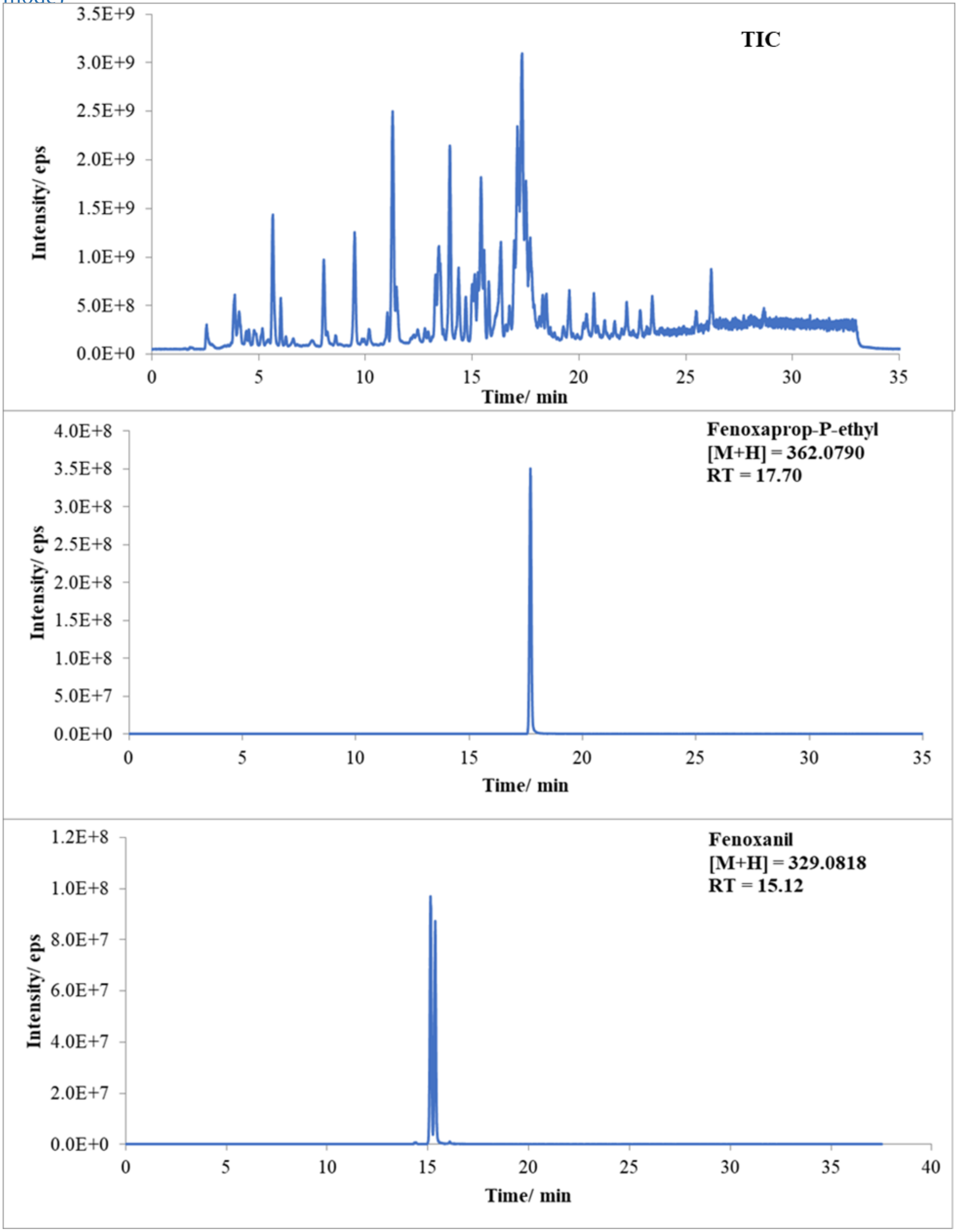

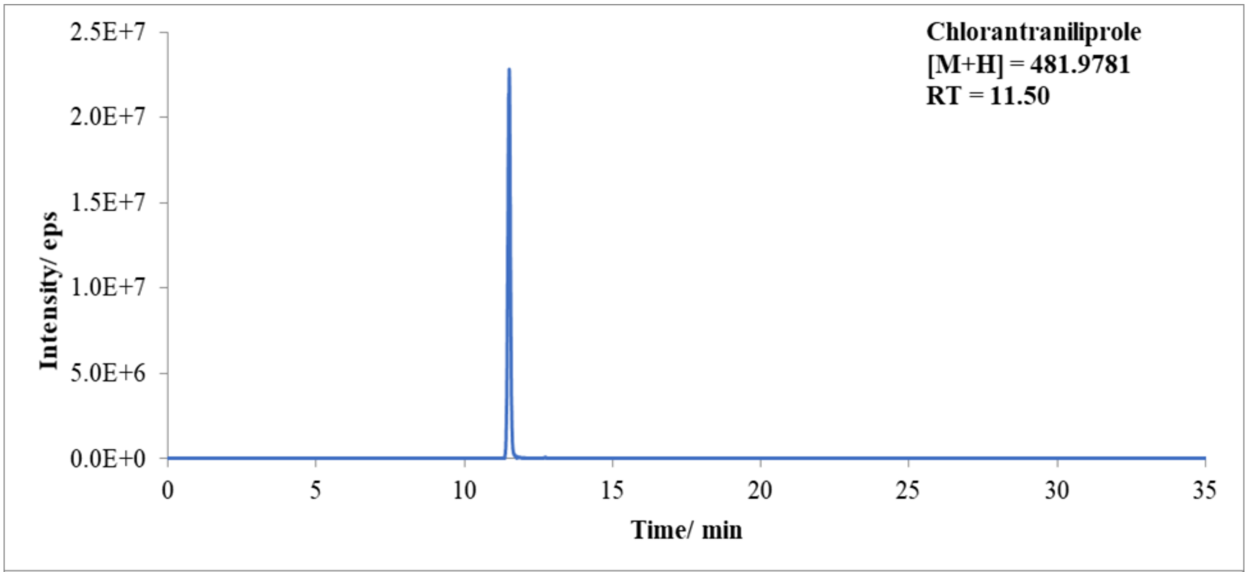
Chlorantraniliprole
[M+H] = 481.9781
RT = 11.50
Intensity/ eps
2.5E+7
2.0E+7
1.5E+7
1.0E+7
5.0E+6
0.0E+0
0
5
10
15
20
25
30
35
Time/ min

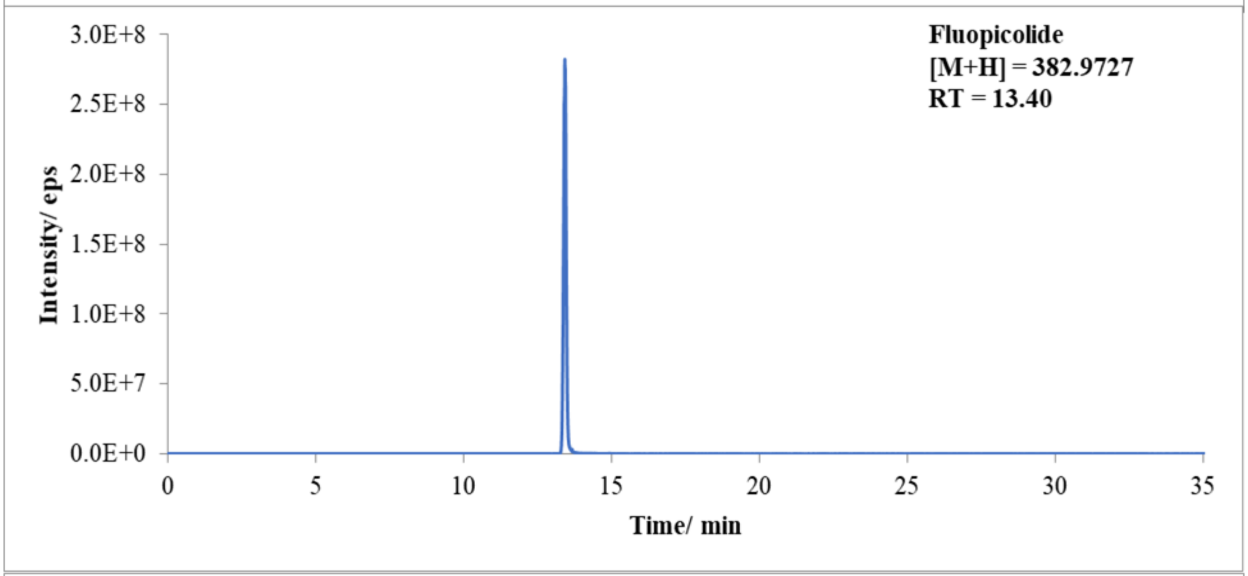
Fluopicolide
[M+H] = 382.9727
RT = 13.40
Intensity/ eps
3.0E+8
2.5E+8
2.0E+8
1.5E+8
1.0E+8
5.0E+7
0.0E+0
0
5
10
15
20
25
30
35
Time/ min

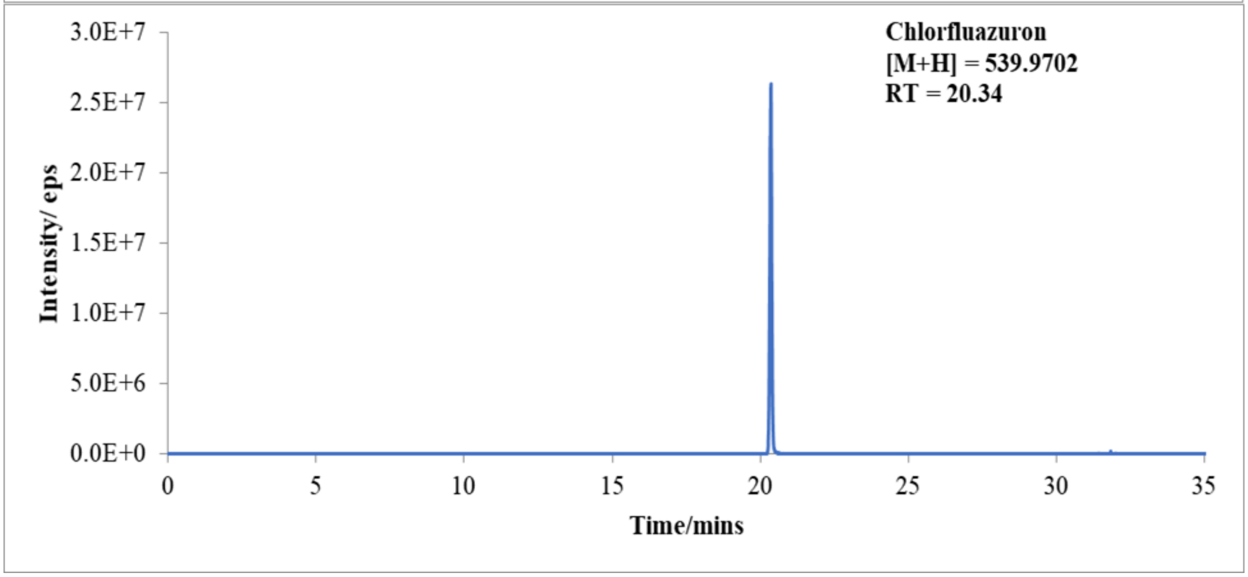
Chlorfluazuron
[M+H] = 539.9702
RT = 20.34
Intensity/ eps
3.0E+7
2.5E+7
2.0E+7
1.5E+7
1.0E+7
5.0E+6
0.0E+0
0
5
10
15
20
25
30
35
Time/mins

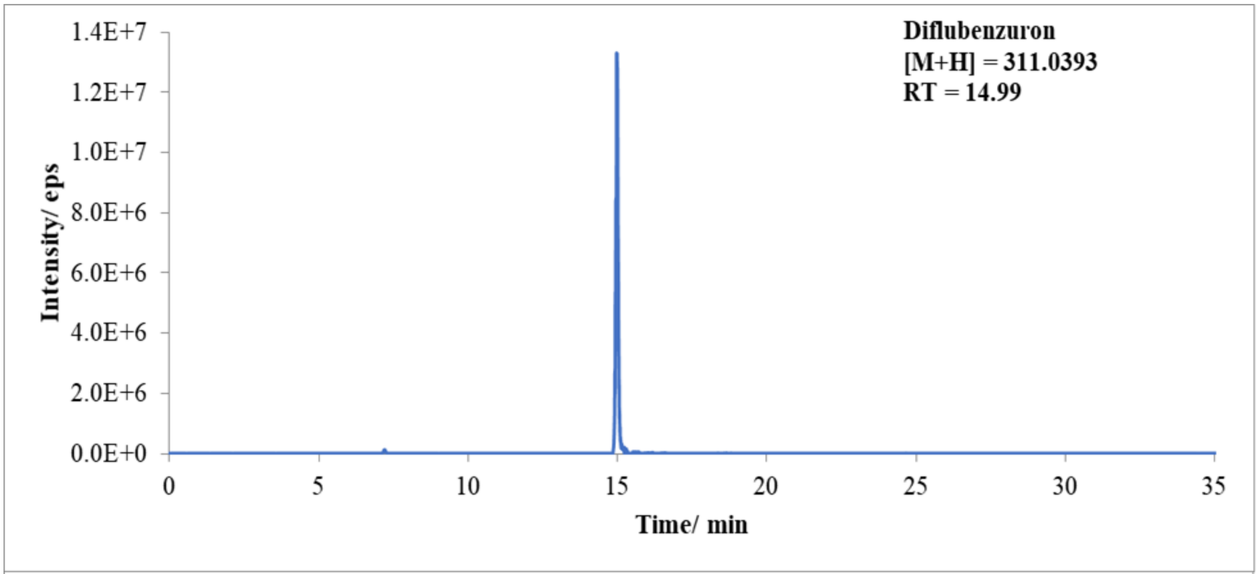
Diflubenzuron
[M+H] = 311.0393
RT = 14.99
Intensity/ eps
1.4E+7
1.2E+7
1.0E+7
8.0E+6
6.0E+6
4.0E+6
2.0E+6
0.0E+0
0
5
10
15
20
25
30
35
Time/ min

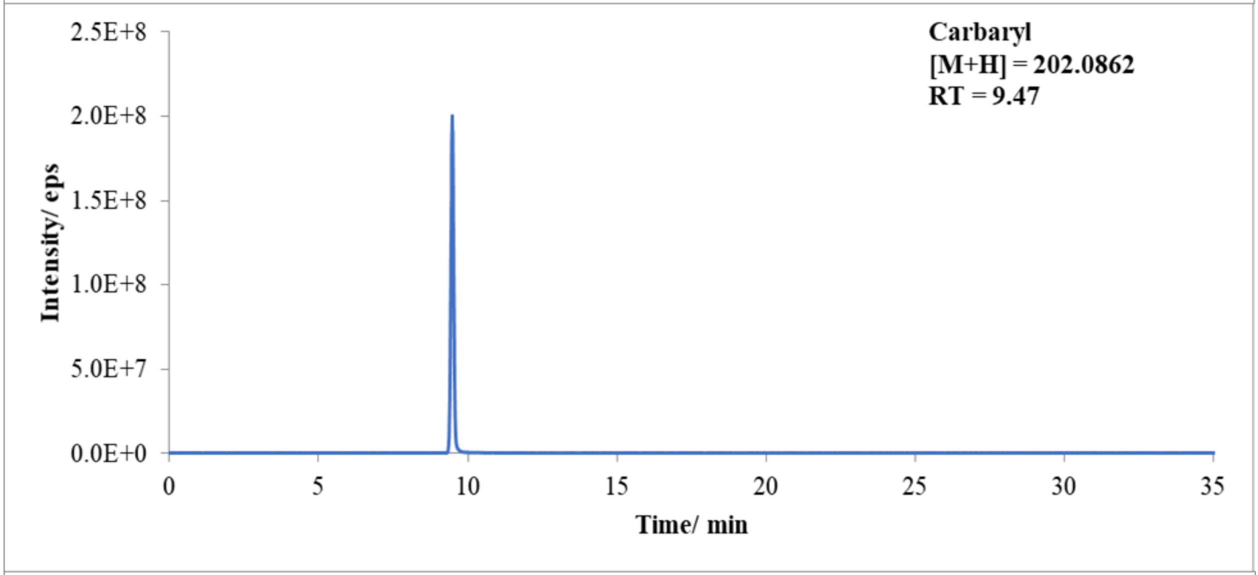
Carbaryl
[M+H] = 202.0862
RT = 9.47
Intensity/ eps
2.5E+8
2.0E+8
1.5E+8
1.0E+8
5.0E+7
0.0E+0
0
5
10
15
20
25
30
35
Time/ min

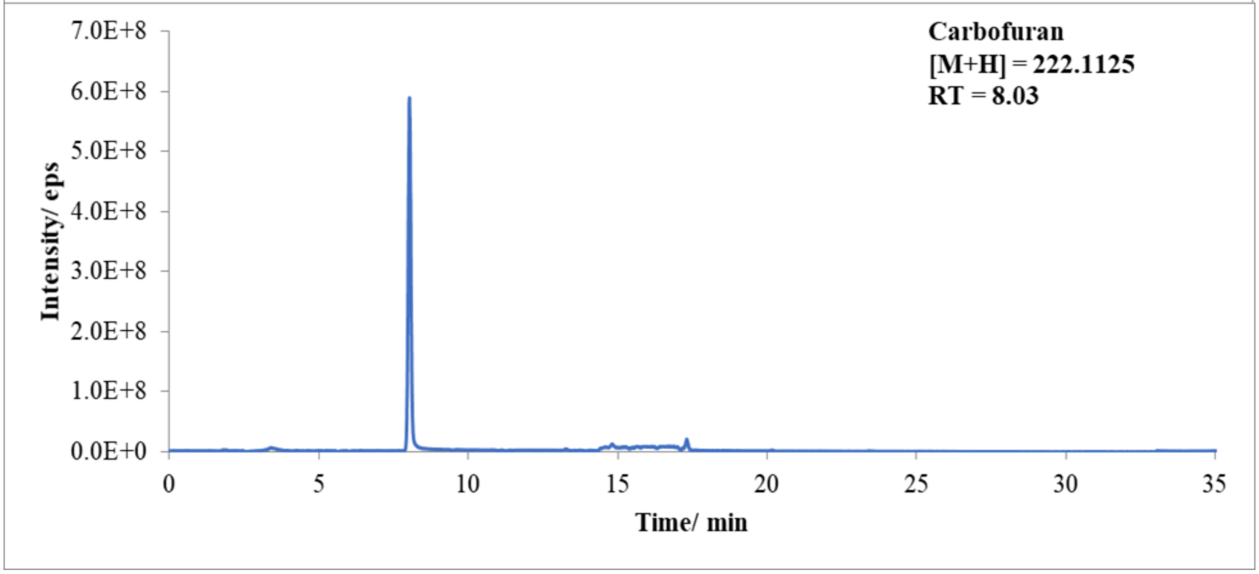
Carbofuran
[M+H] = 222.1125
RT = 8.03
Intensity/ eps
7.0E+8
6.0E+8
5.0E+8
4.0E+8
3.0E+8
2.0E+8
1.0E+8
0.0E+0
0
5
10
15
20
25
30
35
Time/ min

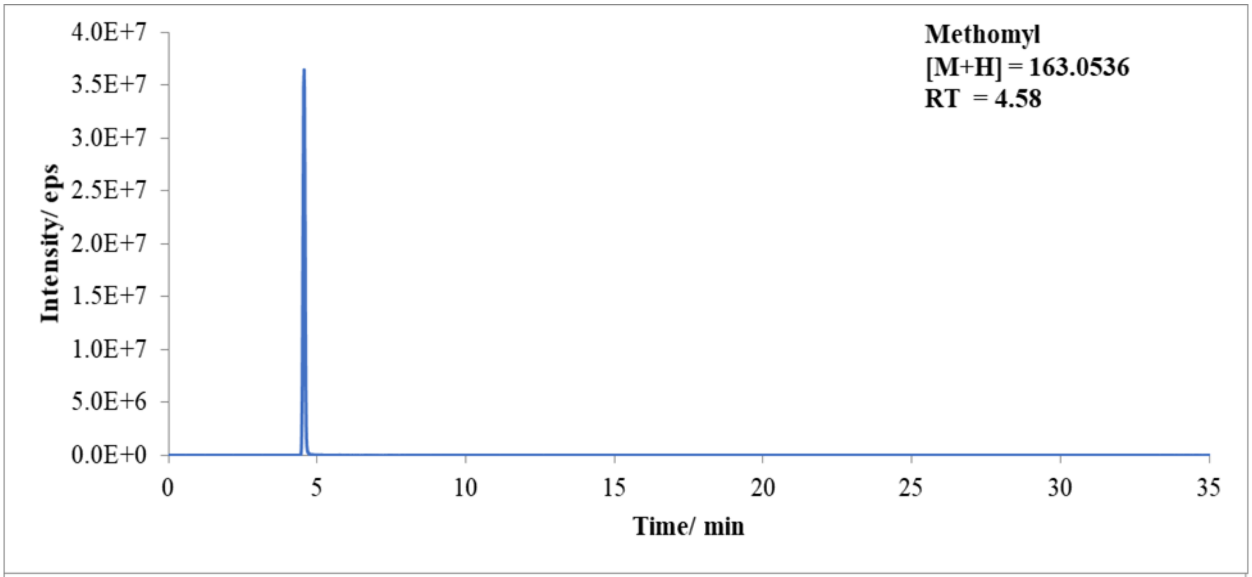
Methomyl
[M+H] = 163.0536
RT = 4.58
Intensity/ eps
4.0E+7
3.5E+7
3.0E+7
2.5E+7
2.0E+7
1.5E+7
1.0E+7
5.0E+6
0.0E+0
0
5
10
15
20
25
30
35
Time/ min

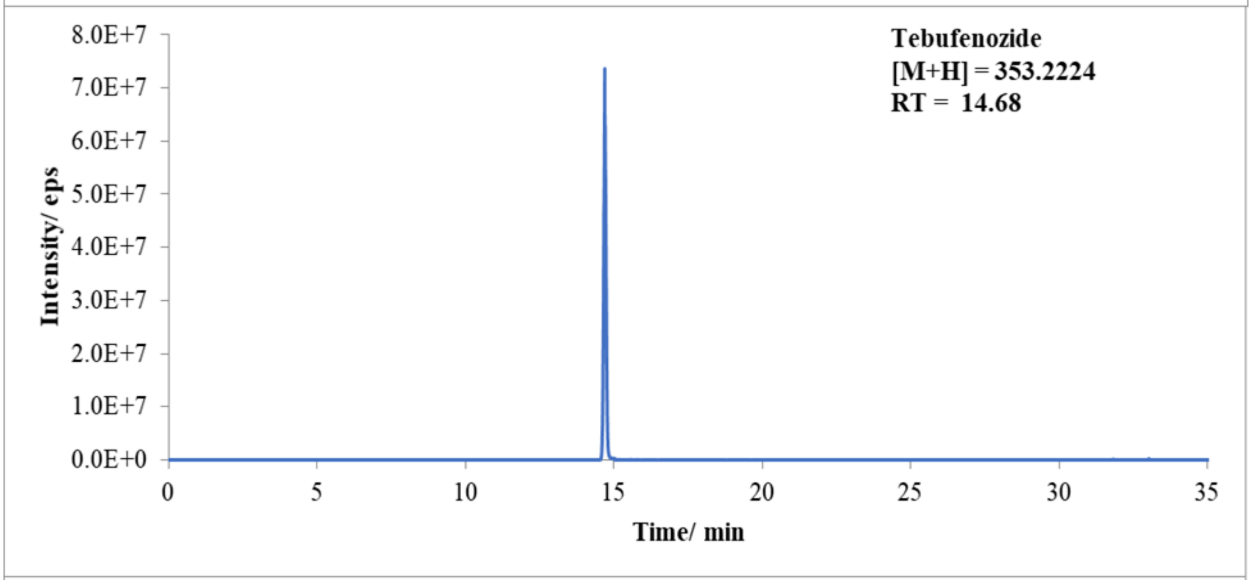
Tebufenozide
[M+H] = 353.2224
RT = 14.68
Intensity/ eps
8.0E+7
7.0E+7
6.0E+7
5.0E+7
4.0E+7
3.0E+7
2.0E+7
1.0E+7
0.0E+0
0
5
10
15
20
25
30
35
Time/ min

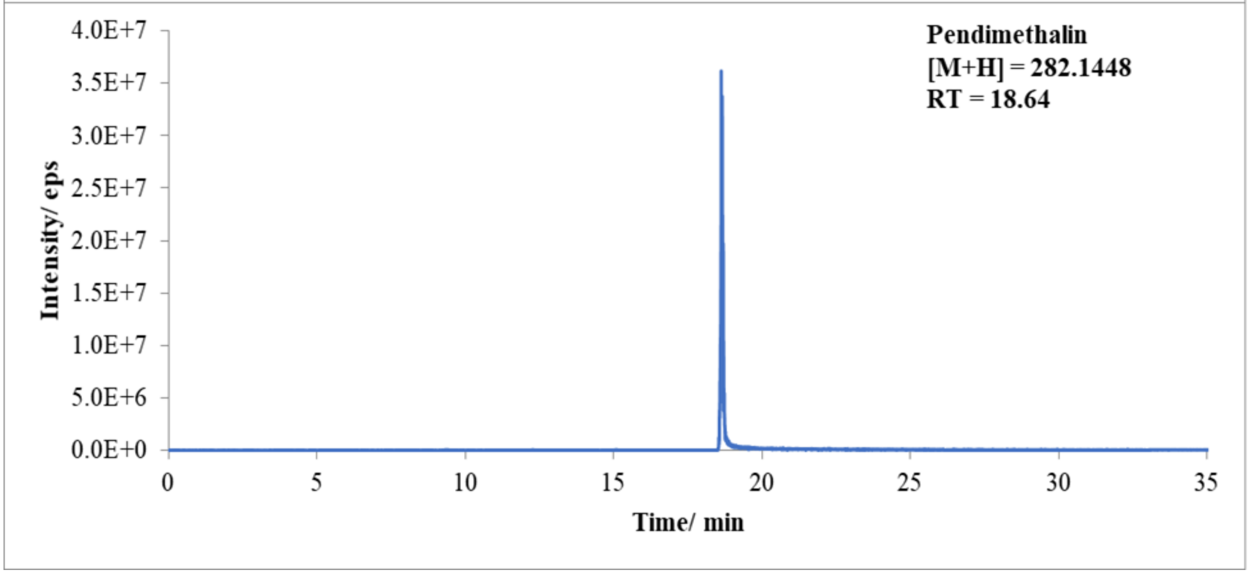
Pendimethalin
[M+H] = 282.1448
RT = 18.64
Intensity/ eps
4.0E+7
3.5E+7
3.0E+7
2.5E+7
2.0E+7
1.5E+7
1.0E+7
5.0E+6
0.0E+0
0
5
10
15
20
25
30
35
Time/ min

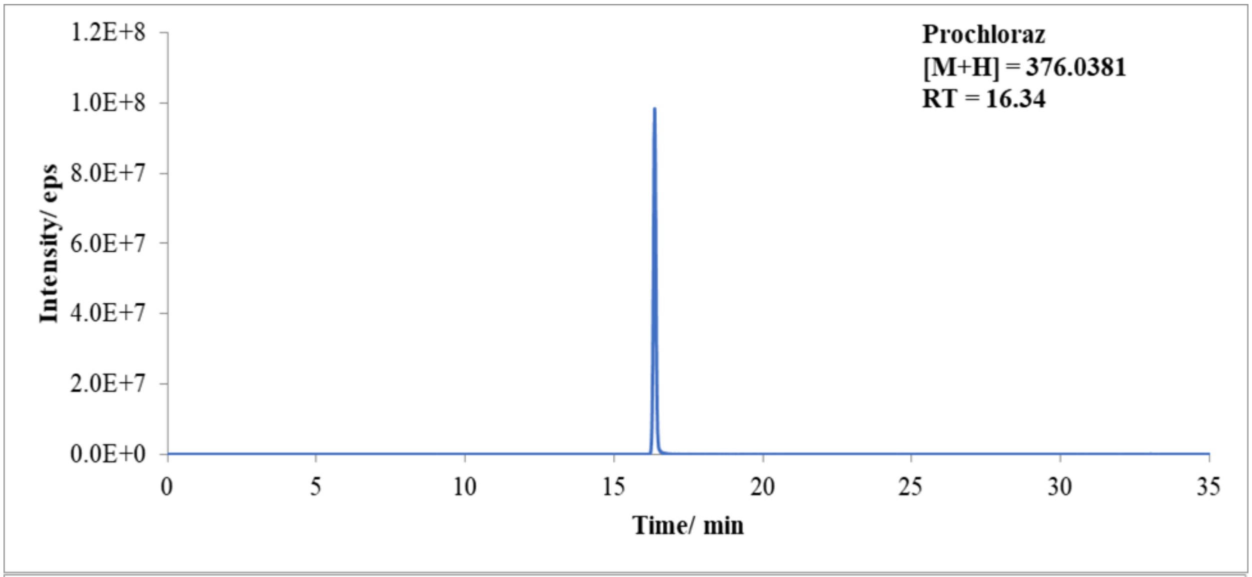
Prochloraz
[M+H] = 376.0381
RT = 16.34
Intensity/ eps
Time/ min

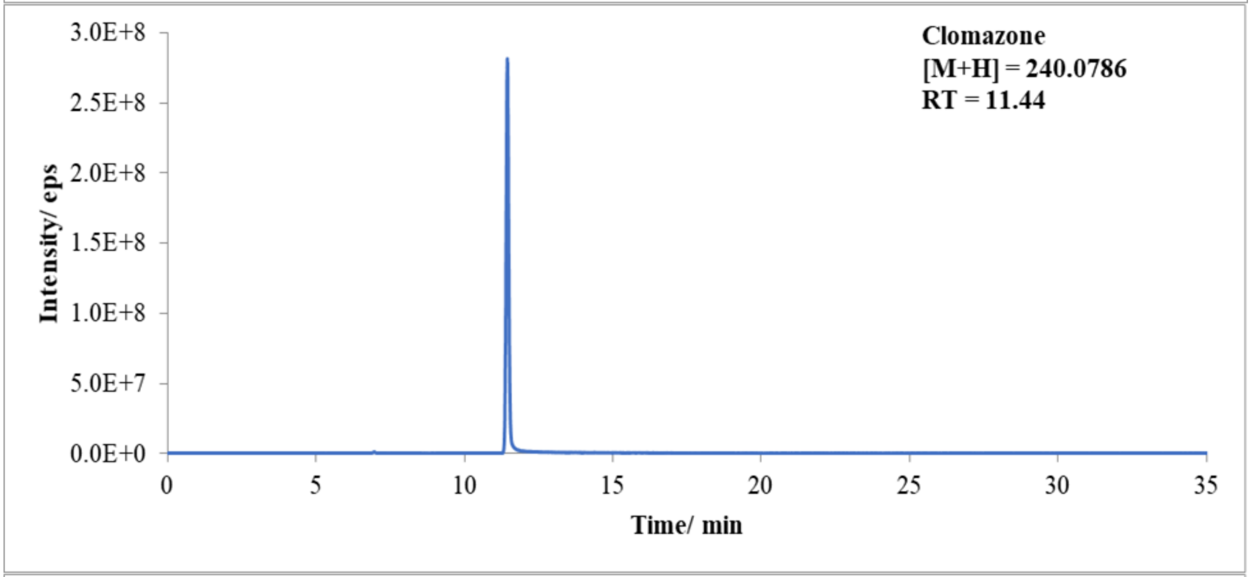
Clomazone
[M+H] = 240.0786
RT = 11.44
Intensity/ eps
Time/ min

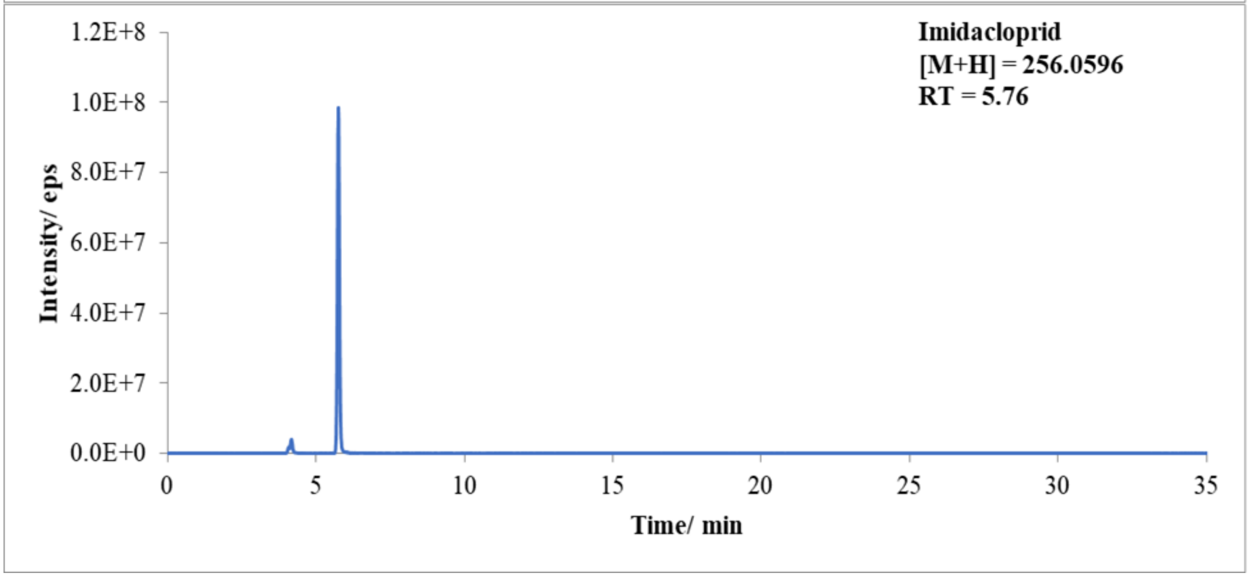
Imidacloprid
[M+H] = 256.0596
RT = 5.76
Intensity/ eps
Time/ min

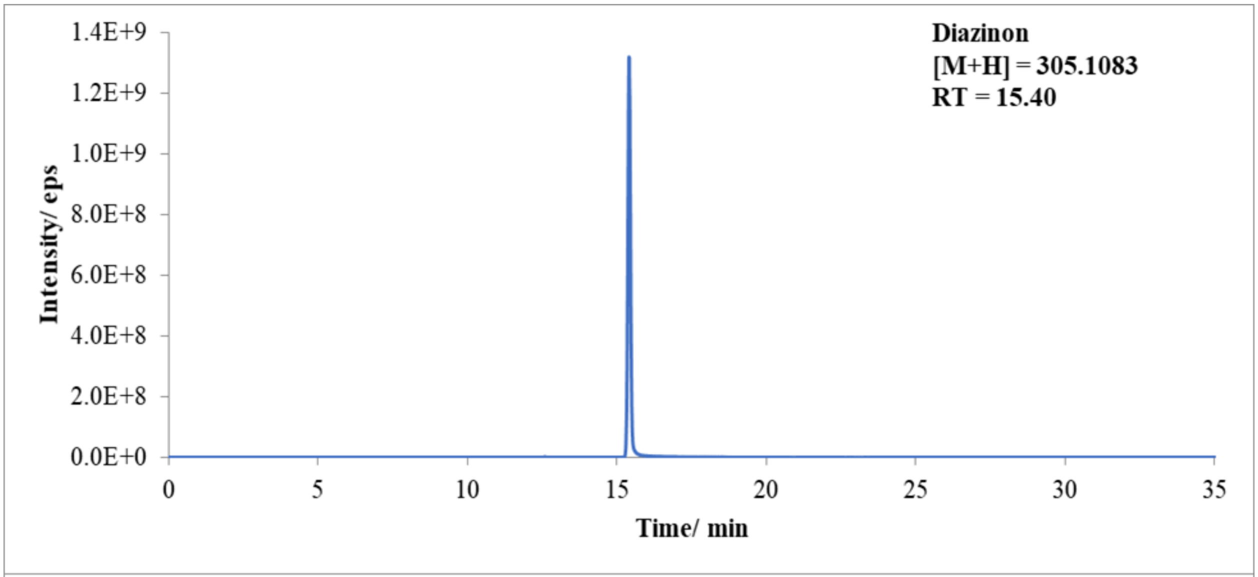
Diazinon
[M+H] = 305.1083
RT = 15.40
Intensity/ eps
Time/ min
1.4E+9
1.2E+9
1.0E+9
8.0E+8
6.0E+8
4.0E+8
2.0E+8
0.0E+0
0
5
10
15
20
25
30
35

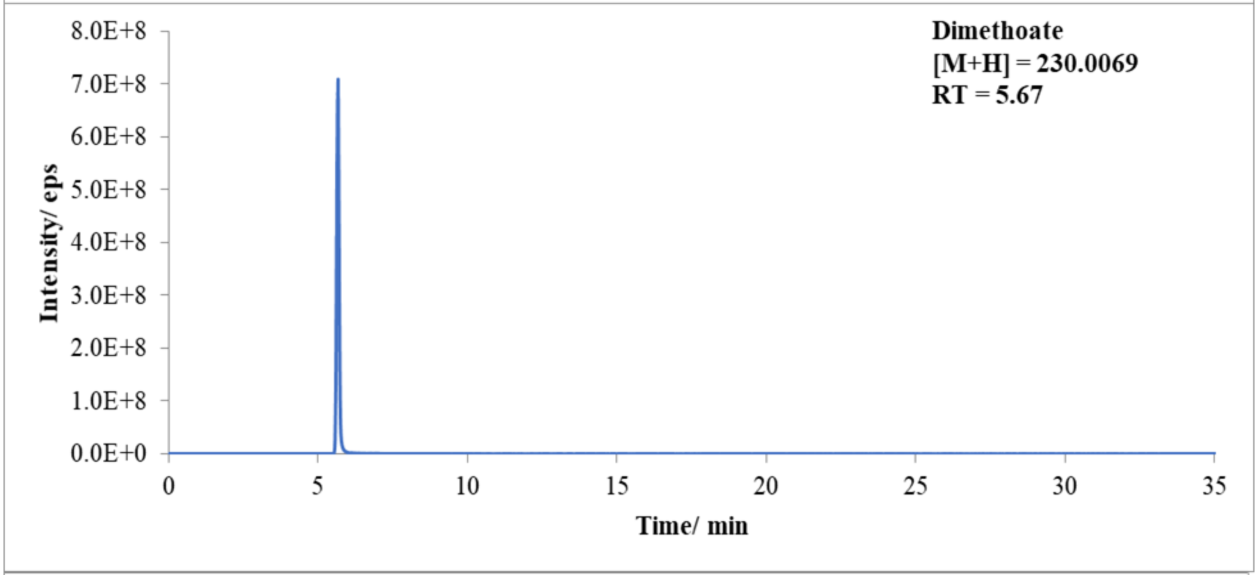
Dimethoate
[M+H] = 230.0069
RT = 5.67
Intensity/ eps
Time/ min
8.0E+8
7.0E+8
6.0E+8
5.0E+8
4.0E+8
3.0E+8
2.0E+8
1.0E+8
0.0E+0
0
5
10
15
20
25
30
35

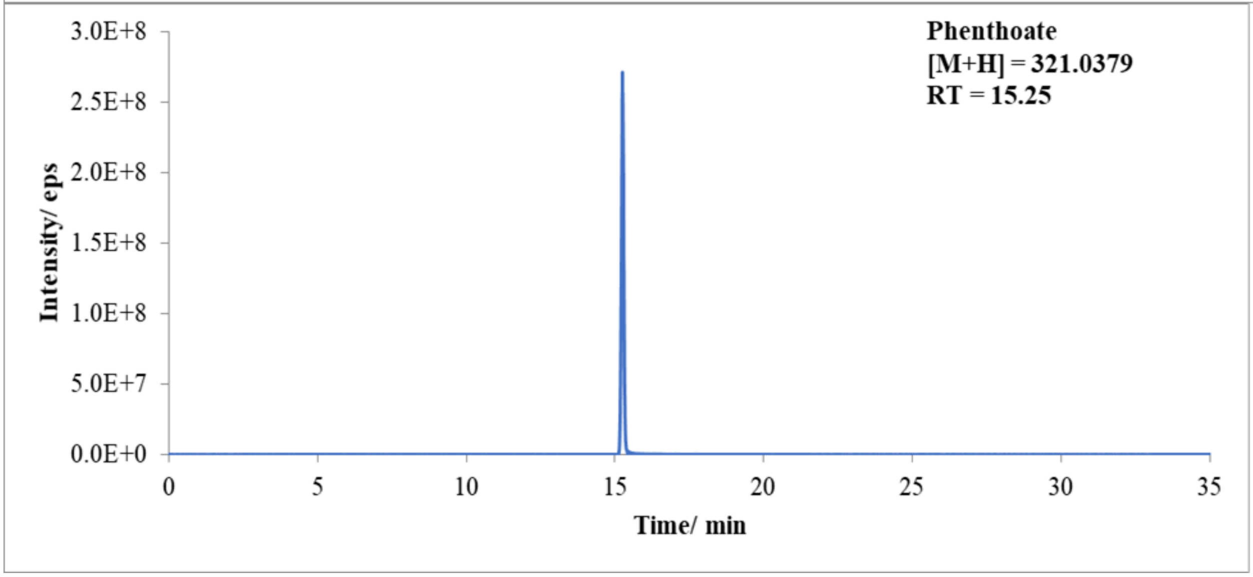
Phenthoate
[M+H] = 321.0379
RT = 15.25
Intensity/ eps
Time/ min
3.0E+8
2.5E+8
2.0E+8
1.5E+8
1.0E+8
5.0E+7
0.0E+0
0
5
10
15
20
25
30
35

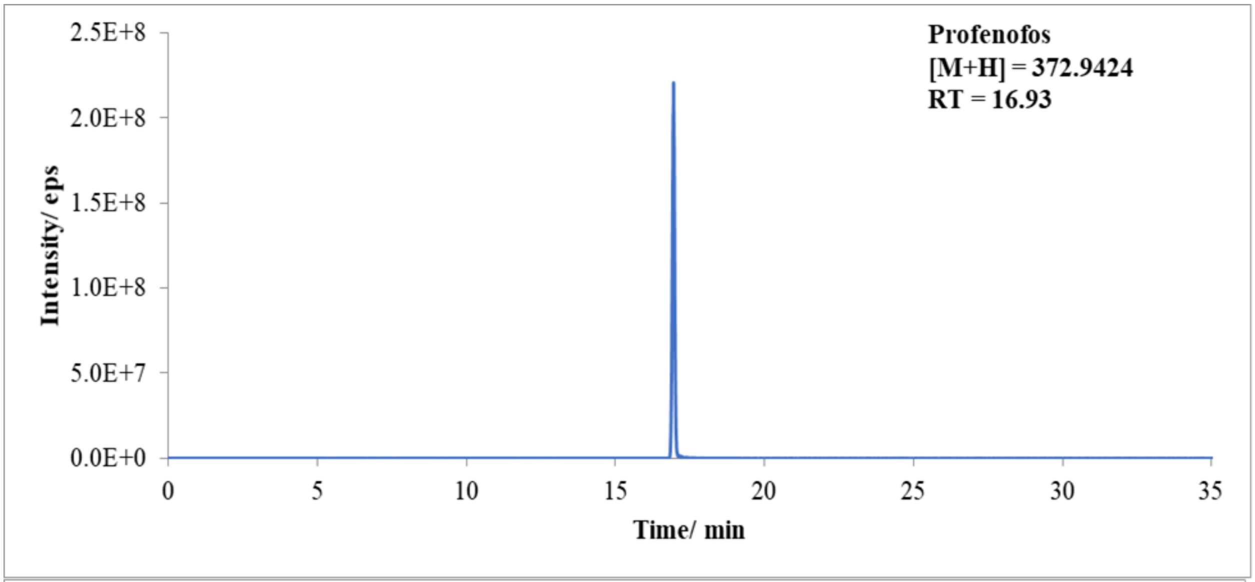
Profenofos
[M+H] = 372.9424
RT = 16.93
Intensity/ eps
Time/ min

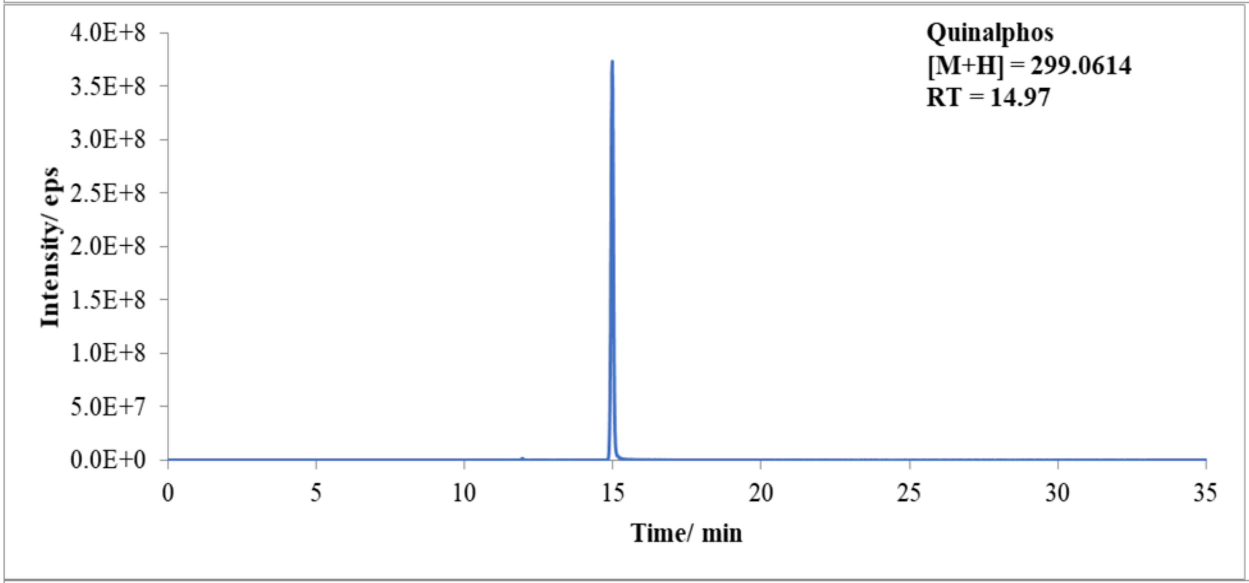
Quinalphos
[M+H] = 299.0614
RT = 14.97
Intensity/ eps
Time/ min

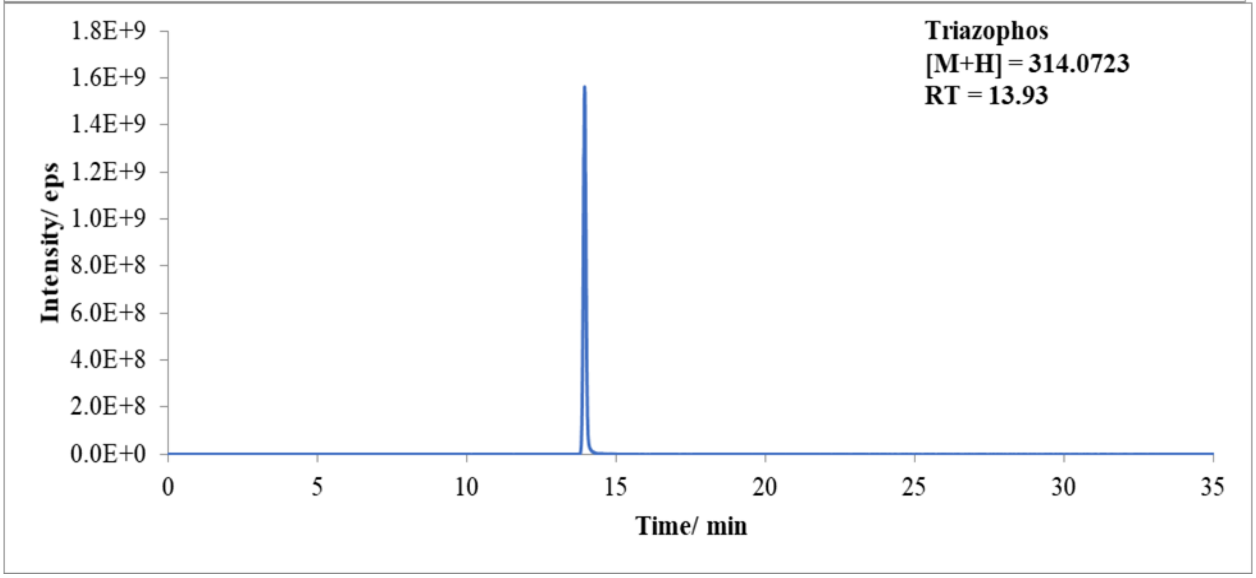
Triazophos
[M+H] = 314.0723
RT = 13.93
Intensity/ eps
Time/ min

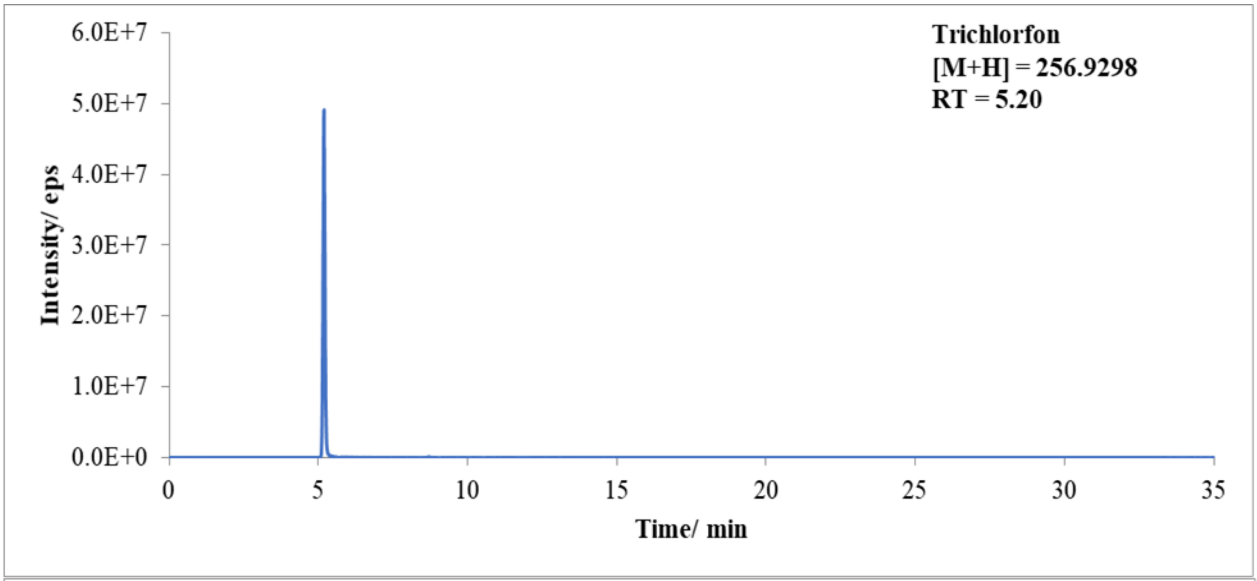
Trichlorfon
[M+H] = 256.9298
RT = 5.20
Intensity/ eps
Time/ min
6.0E+7
5.0E+7
4.0E+7
3.0E+7
2.0E+7
1.0E+7
0.0E+0
0
5
10
15
20
25
30
35

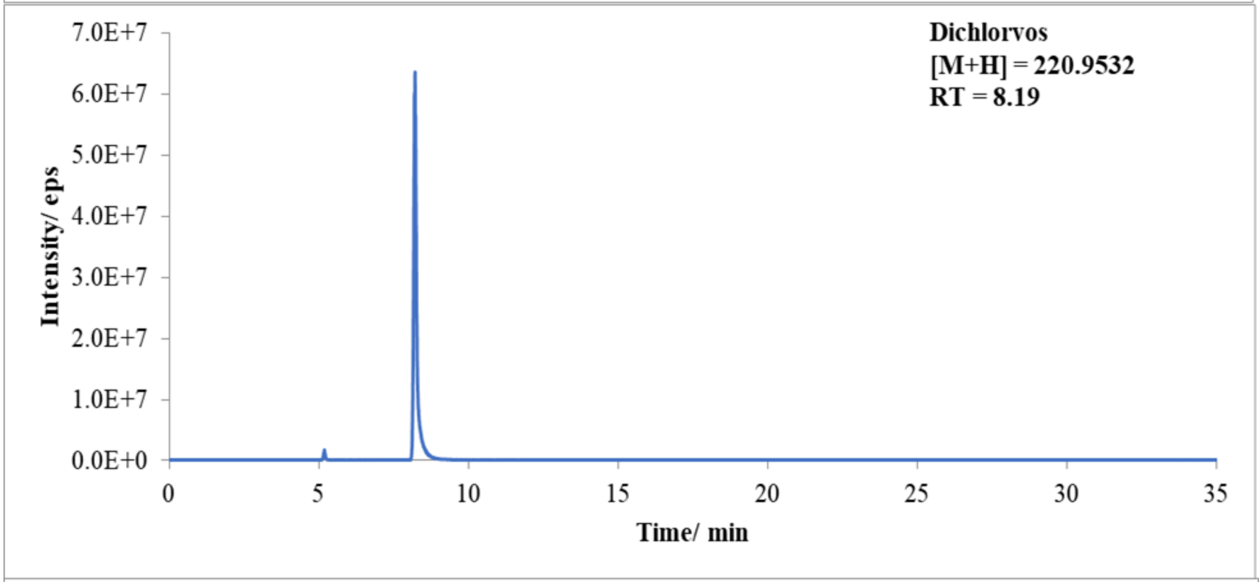
Dichlorvos
[M+H] = 220.9532
RT = 8.19
Intensity/ eps
Time/ min
7.0E+7
6.0E+7
5.0E+7
4.0E+7
3.0E+7
2.0E+7
1.0E+7
0.0E+0
0
5
10
15
20
25
30
35

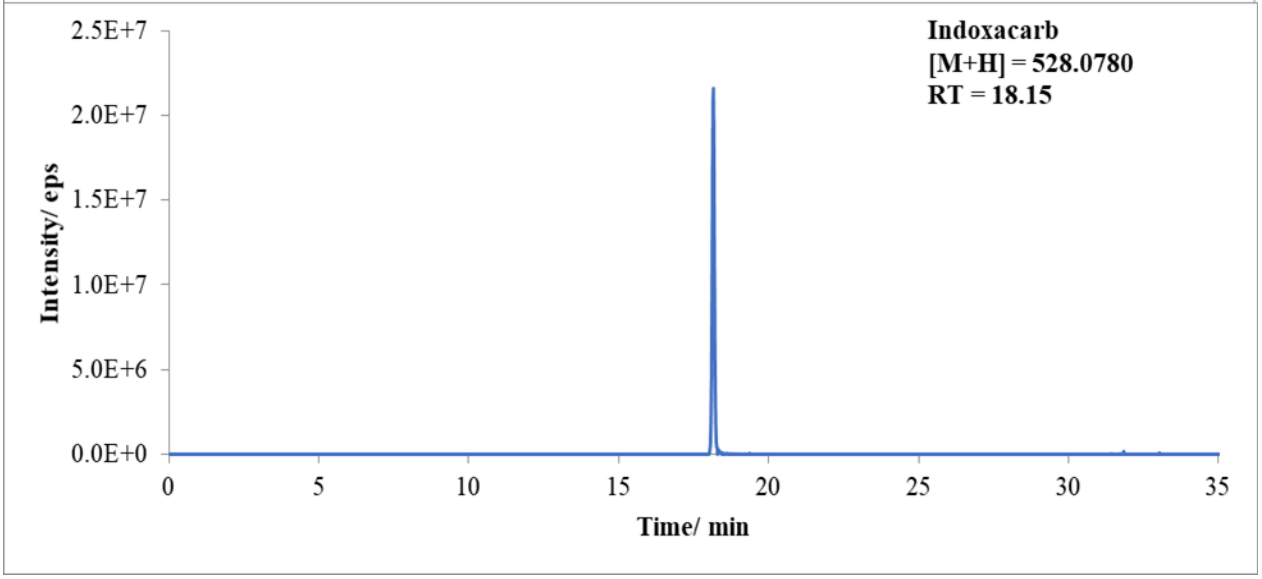
Indoxacarb
[M+H] = 528.0780
RT = 18.15
Intensity/ eps
Time/ min
2.5E+7
2.0E+7
1.5E+7
1.0E+7
5.0E+6
0.0E+0
0
5
10
15
20
25
30
35

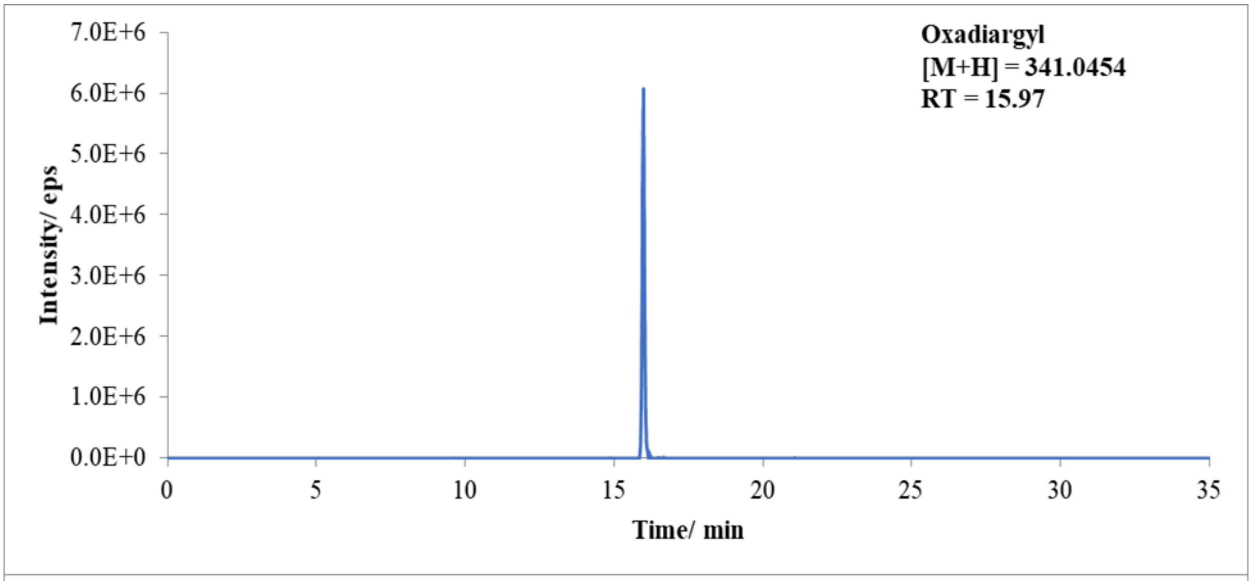
Oxadiargyl
[M+H] = 341.0454
RT = 15.97
Intensity/ eps
Time/ min
7.0E+6
6.0E+6
5.0E+6
4.0E+6
3.0E+6
2.0E+6
1.0E+6
0.0E+0
0
5
10
15
20
25
30
35

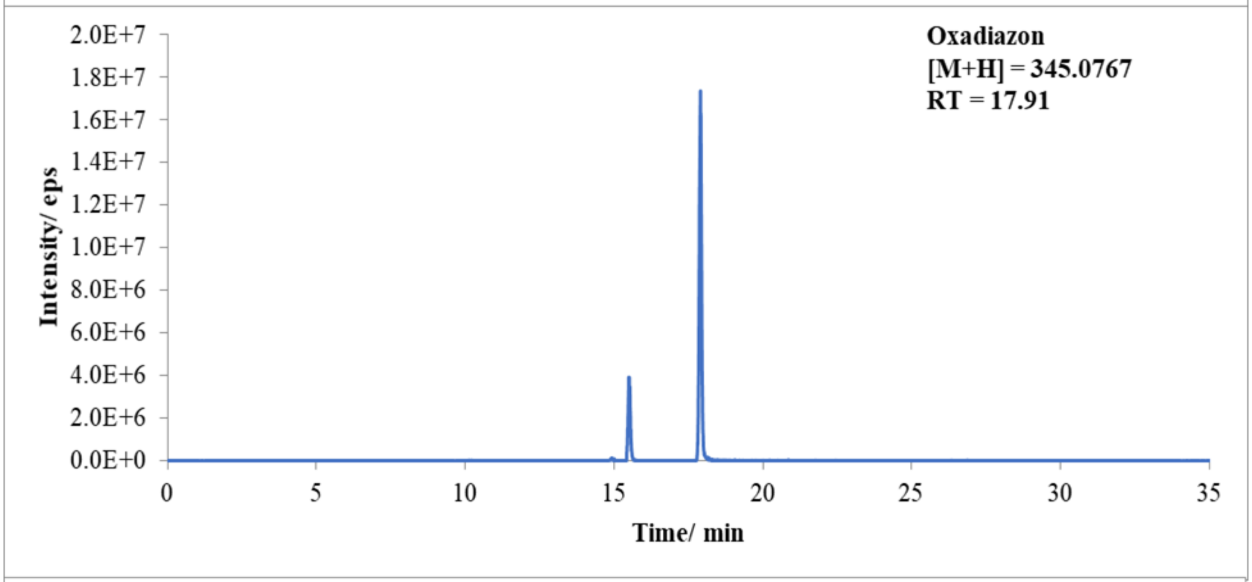
Oxadiazon
[M+H] = 345.0767
RT = 17.91
Intensity/ eps
Time/ min
2.0E+7
1.8E+7
1.6E+7
1.4E+7
1.2E+7
1.0E+7
8.0E+6
6.0E+6
4.0E+6
2.0E+6
0.0E+0
0
5
10
15
20
25
30
35

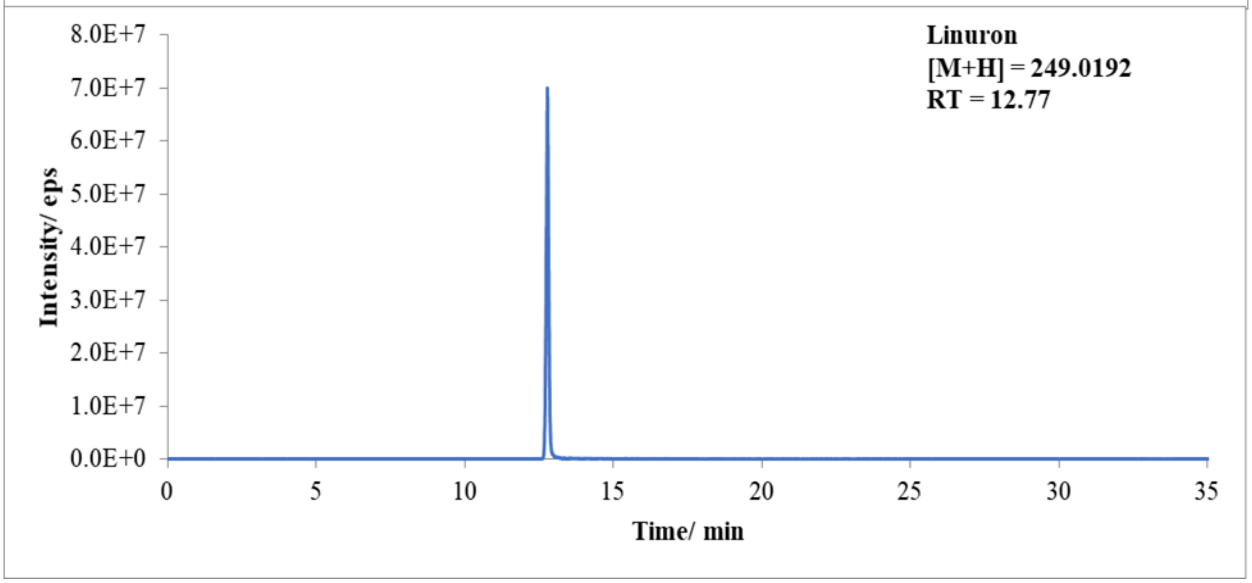
Linuron
[M+H] = 249.0192
RT = 12.77
Intensity/ eps
Time/ min
8.0E+7
7.0E+7
6.0E+7
5.0E+7
4.0E+7
3.0E+7
2.0E+7
1.0E+7
0.0E+0
0
5
10
15
20
25
30
35

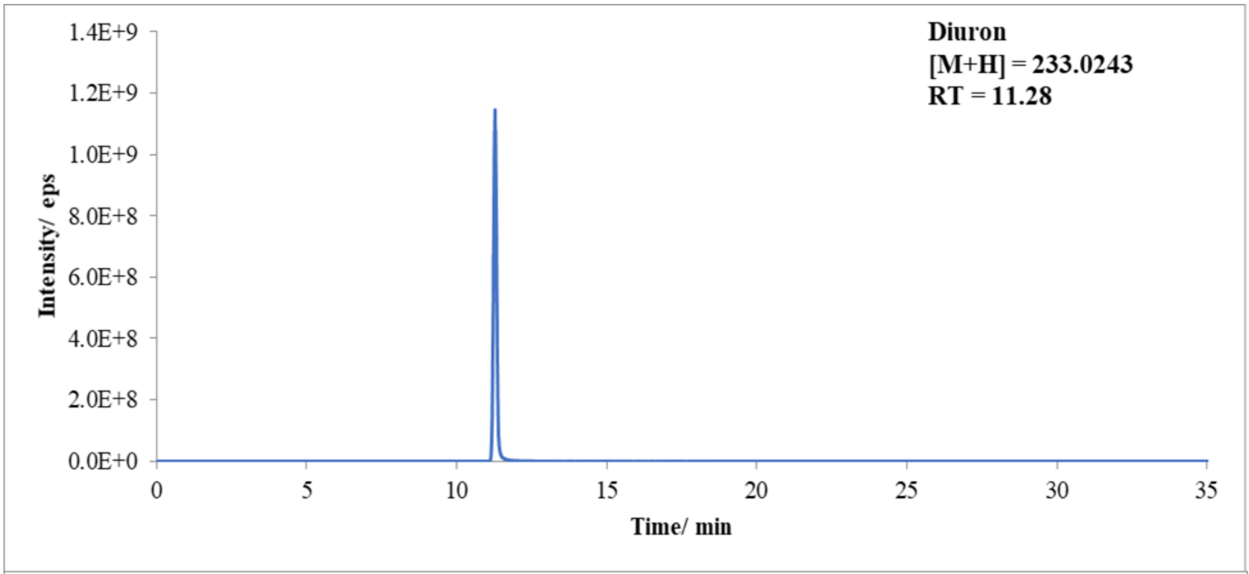
Diuron
[M+H] = 233.0243
RT = 11.28
Intensity/ eps
1.4E+9
1.2E+9
1.0E+9
8.0E+8
6.0E+8
4.0E+8
2.0E+8
0.0E+0
0
5
10
15
20
25
30
35
Time/ min

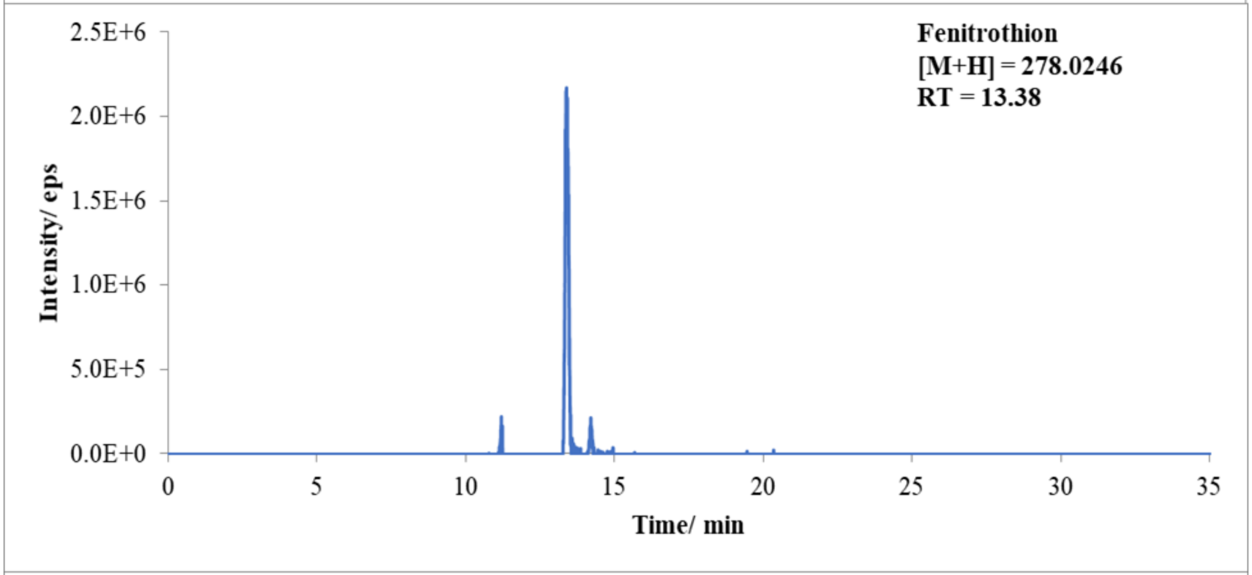
Fenitrothion
[M+H] = 278.0246
RT = 13.38
Intensity/ eps
2.5E+6
2.0E+6
1.5E+6
1.0E+6
5.0E+5
0.0E+0
0
5
10
15
20
25
30
35
Time/ min

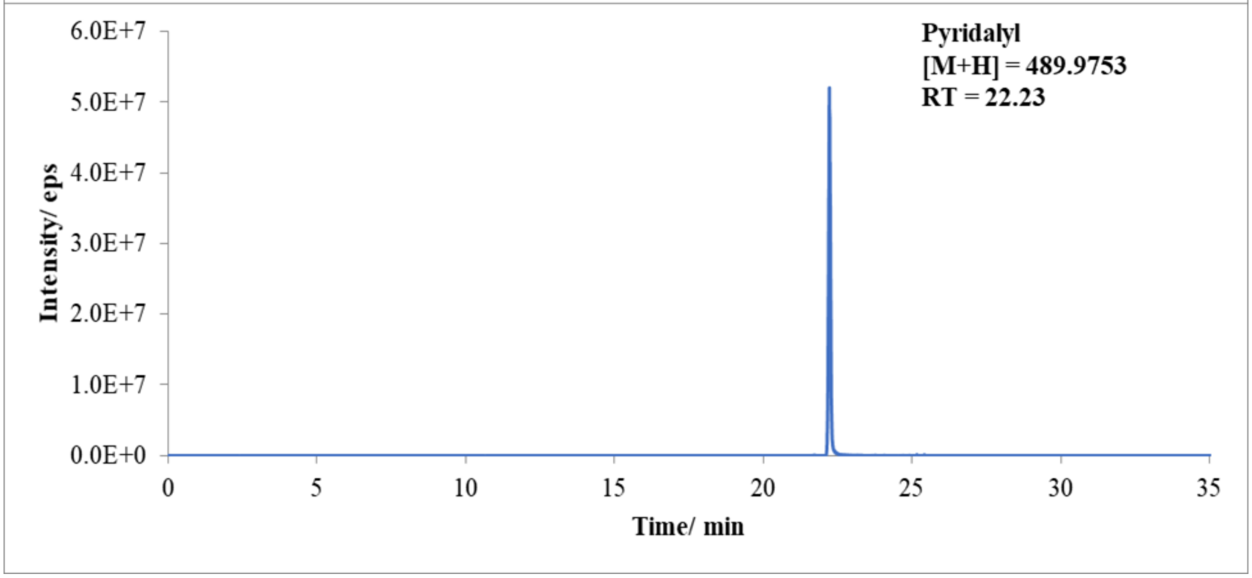
Pyridalyl
[M+H] = 489.9753
RT = 22.23
Intensity/ eps
6.0E+7
5.0E+7
4.0E+7
3.0E+7
2.0E+7
1.0E+7
0.0E+0
0
5
10
15
20
25
30
35
Time/ min

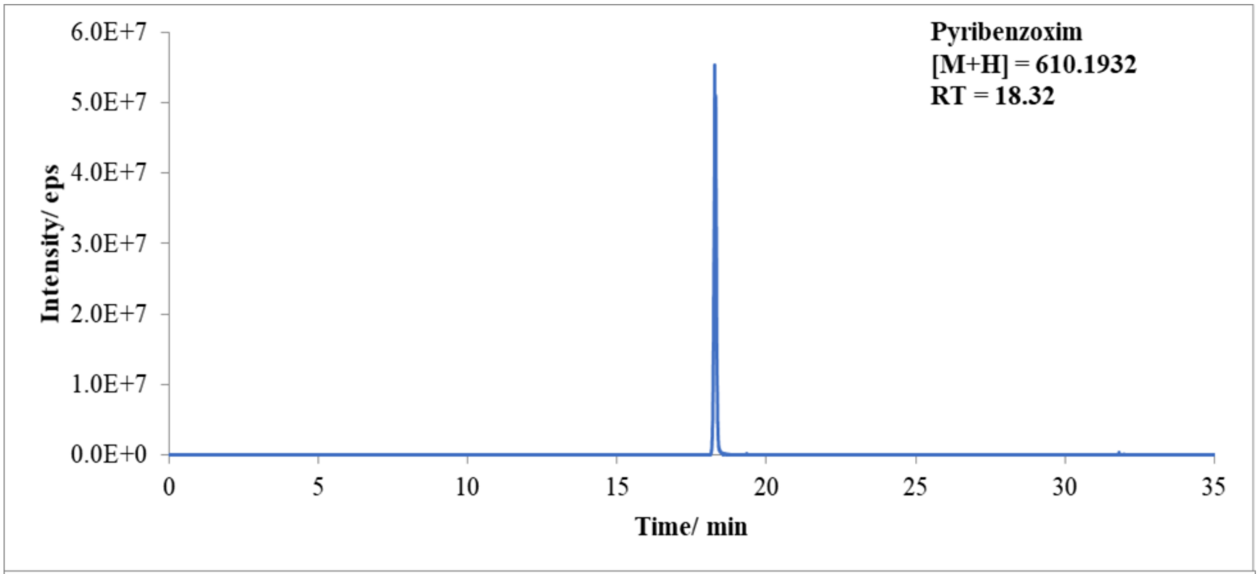
Pyribenzoxim
[M+H] = 610.1932
RT = 18.32
Intensity/ eps
6.0E+7
5.0E+7
4.0E+7
3.0E+7
2.0E+7
1.0E+7
0.0E+0
0
5
10
15
20
25
30
35
Time/ min
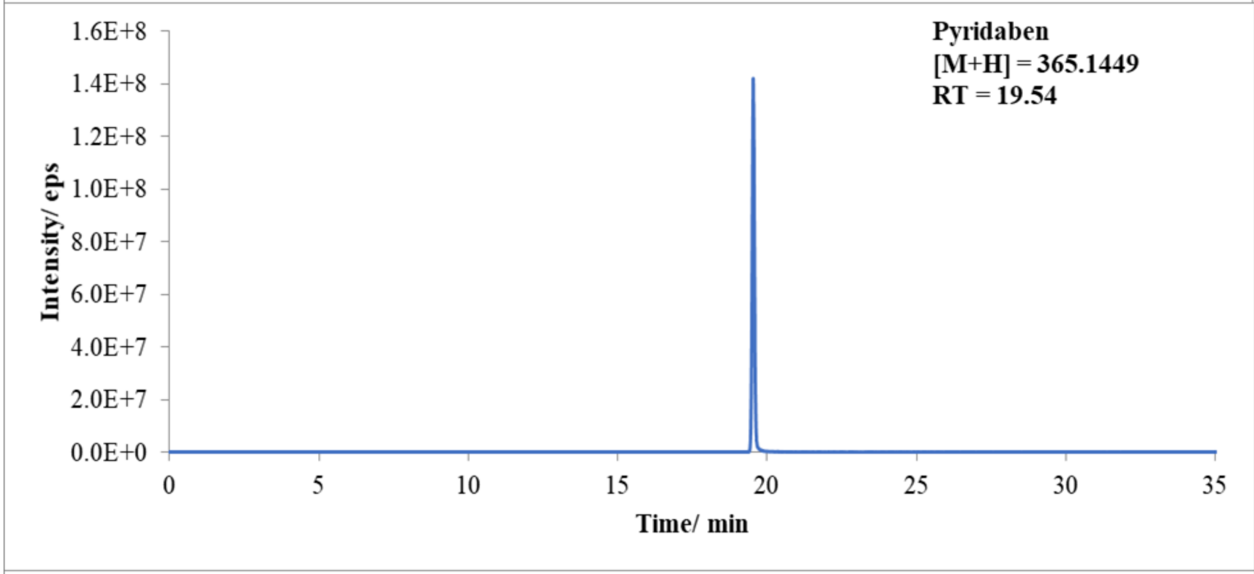
Pyridaben
[M+H] = 365.1449
RT = 19.54
Intensity/ eps
1.6E+8
1.4E+8
1.2E+8
1.0E+8
8.0E+7
6.0E+7
4.0E+7
2.0E+7
0.0E+0
0
5
10
15
20
25
30
35
Time/ min
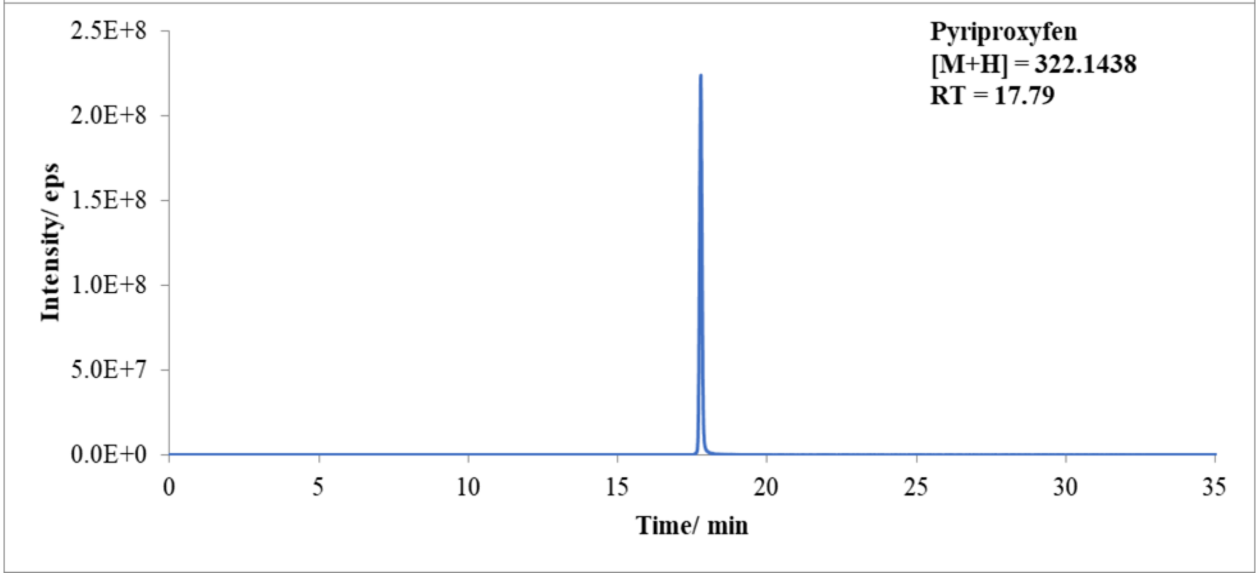
Pyriproxyfen
[M+H] = 322.1438
RT = 17.79
Intensity/ eps
2.5E+8
2.0E+8
1.5E+8
1.0E+8
5.0E+7
0.0E+0
0
5
10
15
20
25
30
35
Time/ min

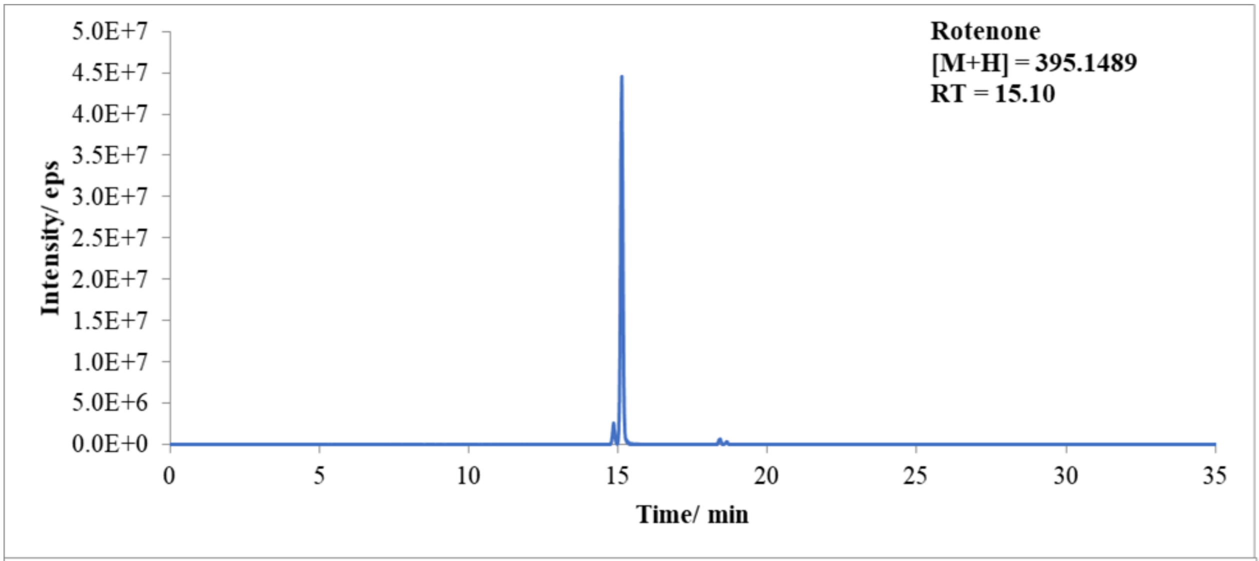
Rotenone
[M+H] = 395.1489
RT = 15.10
Intensity/ eps
5.0E+7
4.5E+7
4.0E+7
3.5E+7
3.0E+7
2.5E+7
2.0E+7
1.5E+7
1.0E+7
5.0E+6
0.0E+0
0
5
10
15
20
25
30
35
Time/ min

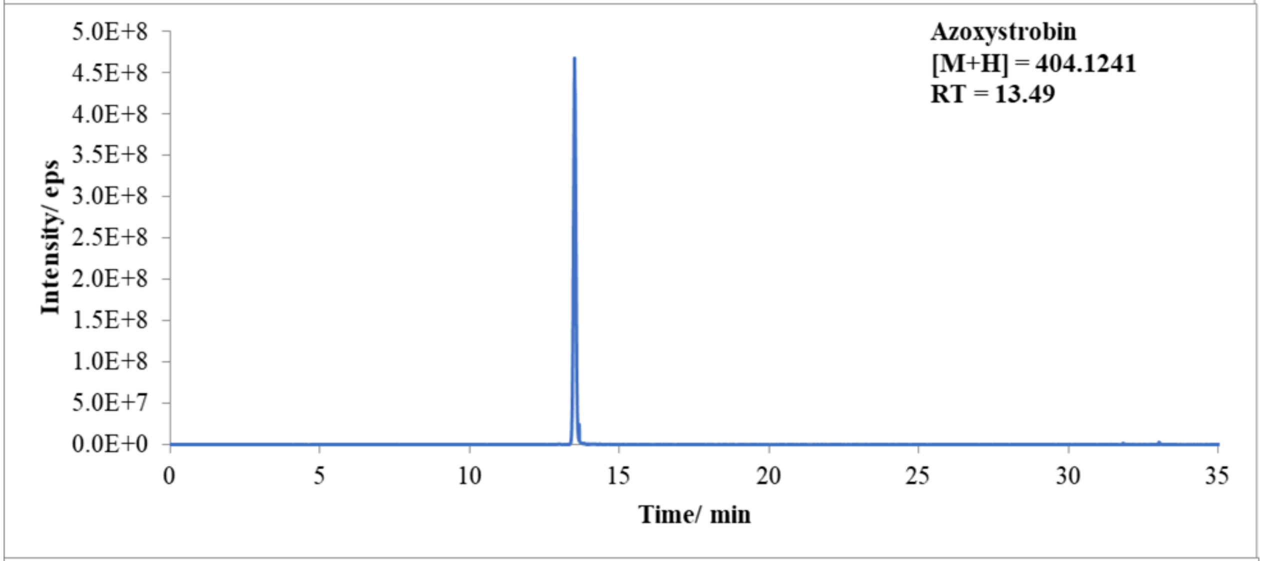
Azoxystrobin
[M+H] = 404.1241
RT = 13.49
Intensity/ eps
5.0E+8
4.5E+8
4.0E+8
3.5E+8
3.0E+8
2.5E+8
2.0E+8
1.5E+8
1.0E+8
5.0E+7
0.0E+0
0
5
10
15
20
25
30
35
Time/ min

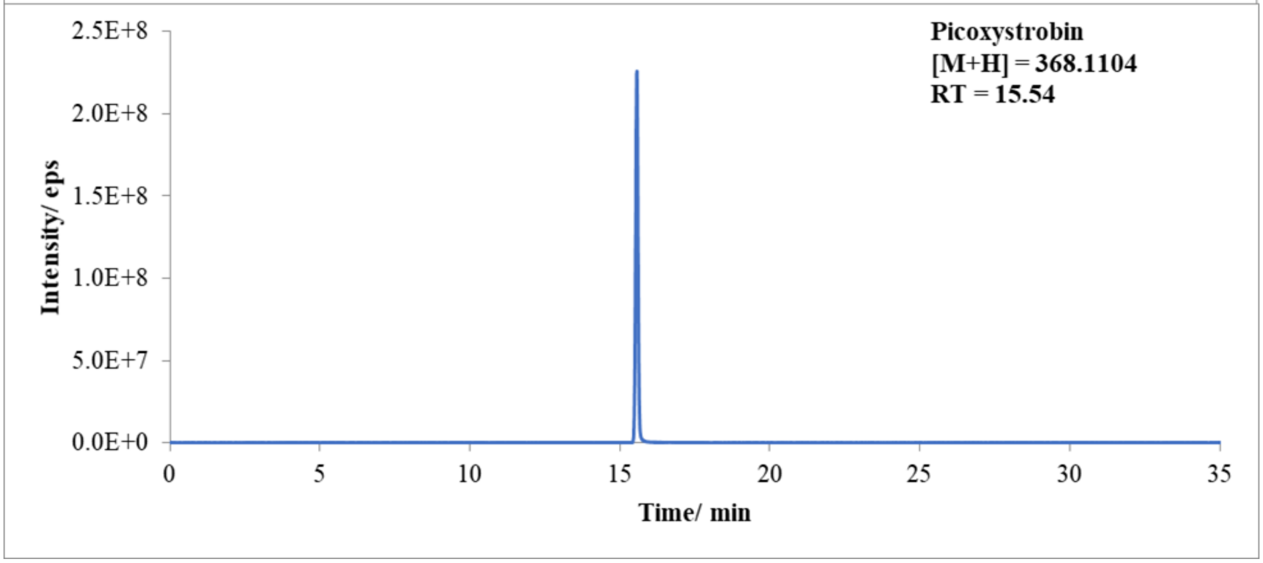
Picoxystrobin
[M+H] = 368.1104
RT = 15.54
Intensity/ eps
2.5E+8
2.0E+8
1.5E+8
1.0E+8
5.0E+7
0.0E+0
0
5
10
15
20
25
30
35
Time/ min

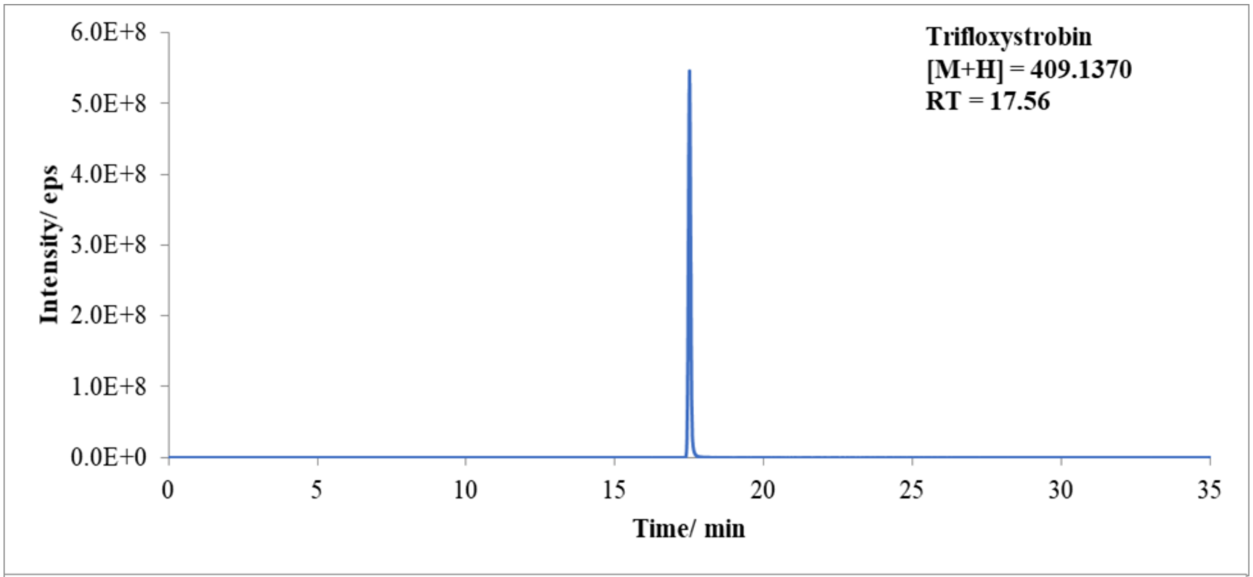
Trifloxystrobin
[M+H] = 409.1370
RT = 17.56
Intensity/ eps
Time/ min
6.0E+8
5.0E+8
4.0E+8
3.0E+8
2.0E+8
1.0E+8
0.0E+0
0
5
10
15
20
25
30
35

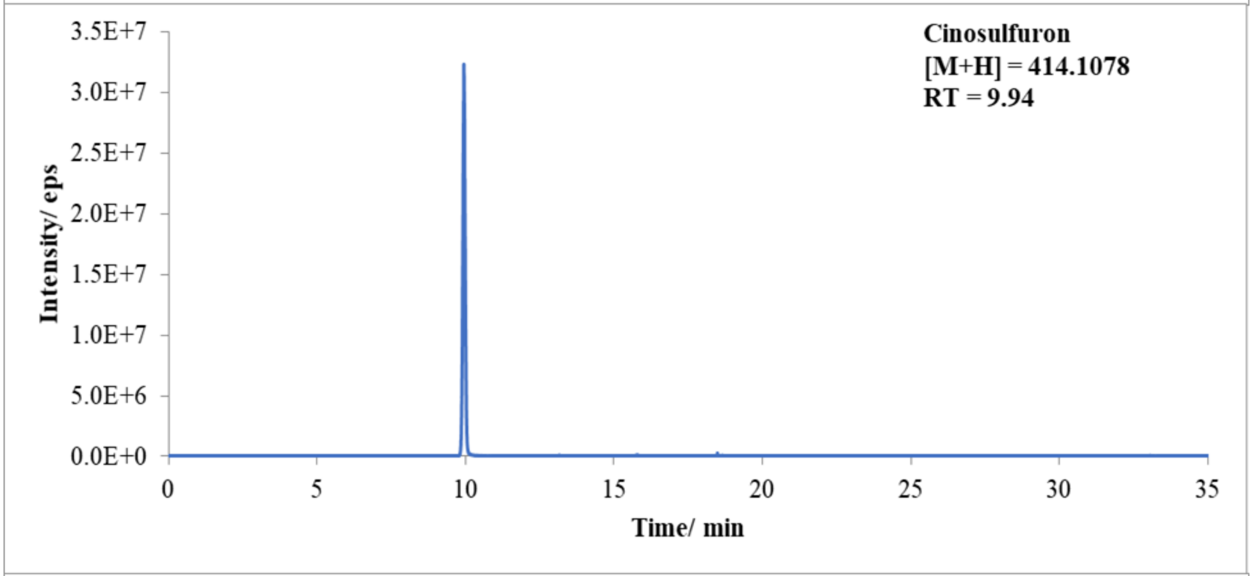
Cinosulfuron
[M+H] = 414.1078
RT = 9.94
Intensity/ eps
Time/ min
3.5E+7
3.0E+7
2.5E+7
2.0E+7
1.5E+7
1.0E+7
5.0E+6
0.0E+0
0
5
10
15
20
25
30
35

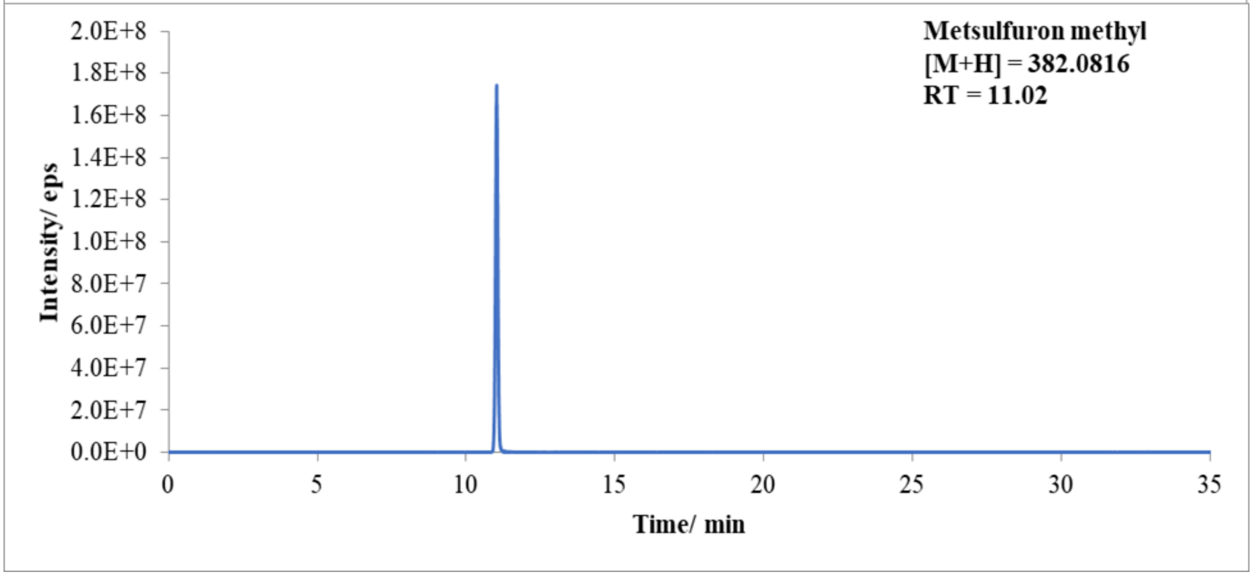
Metsulfuron methyl
[M+H] = 382.0816
RT = 11.02
Intensity/ eps
Time/ min
2.0E+8
1.8E+8
1.6E+8
1.4E+8
1.2E+8
1.0E+8
8.0E+7
6.0E+7
4.0E+7
2.0E+7
0.0E+0
0
5
10
15
20
25
30
35

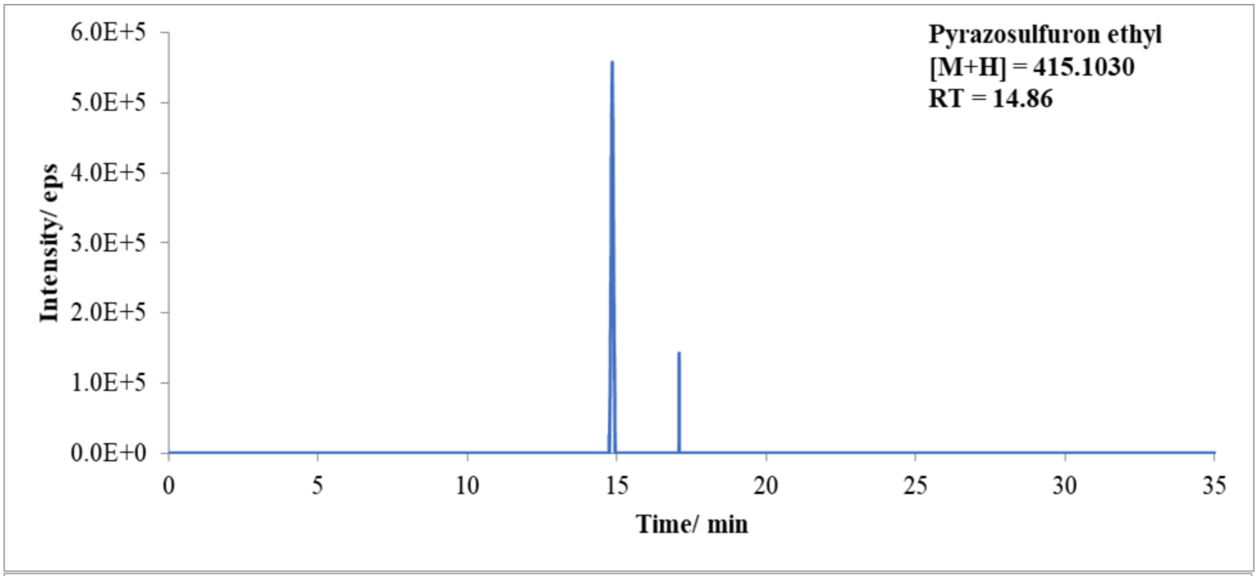
Pyrazosulfuron ethyl
[M+H] = 415.1030
RT = 14.86
Intensity/ eps
Time/ min

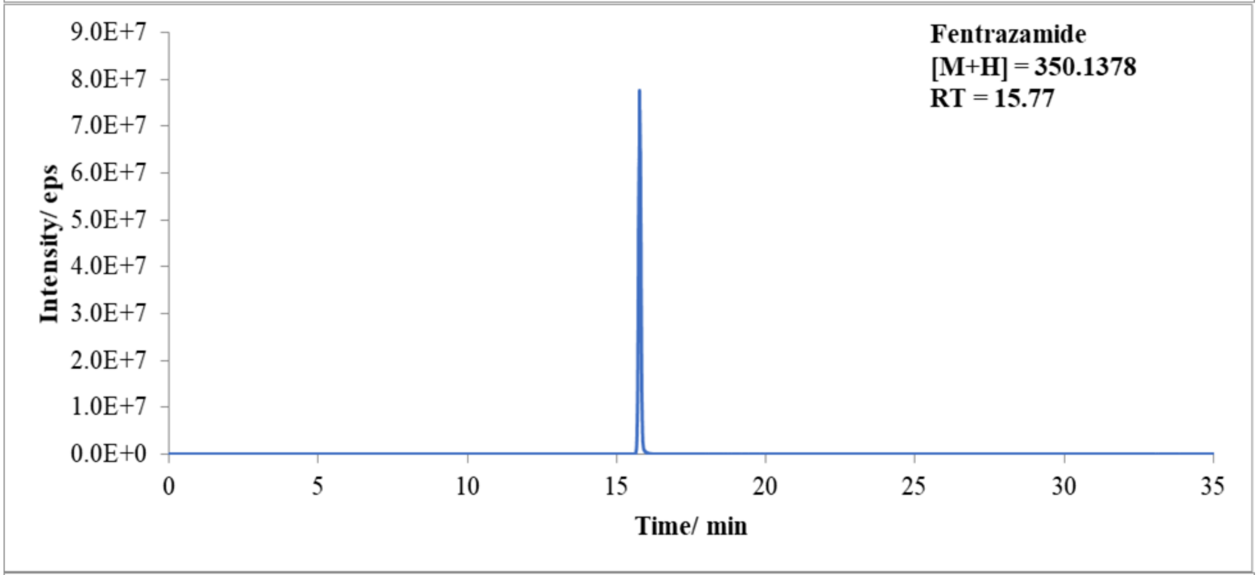
Fentrazamide
[M+H] = 350.1378
RT = 15.77
Intensity/ eps
Time/ min

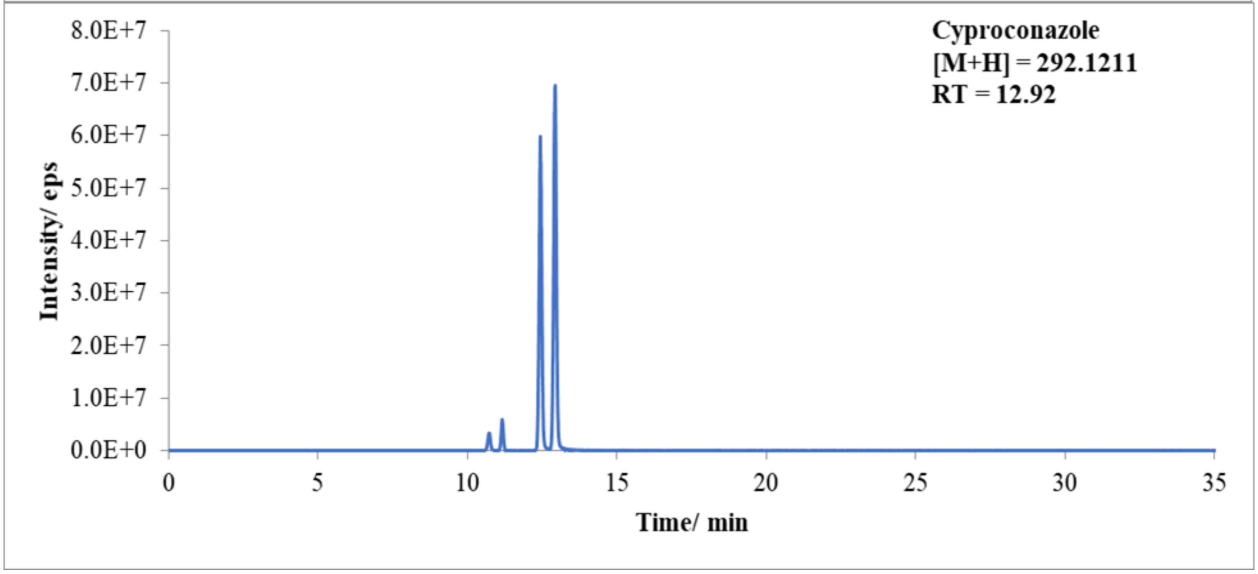
Cyproconazole
[M+H] = 292.1211
RT = 12.92
Intensity/ eps
Time/ min

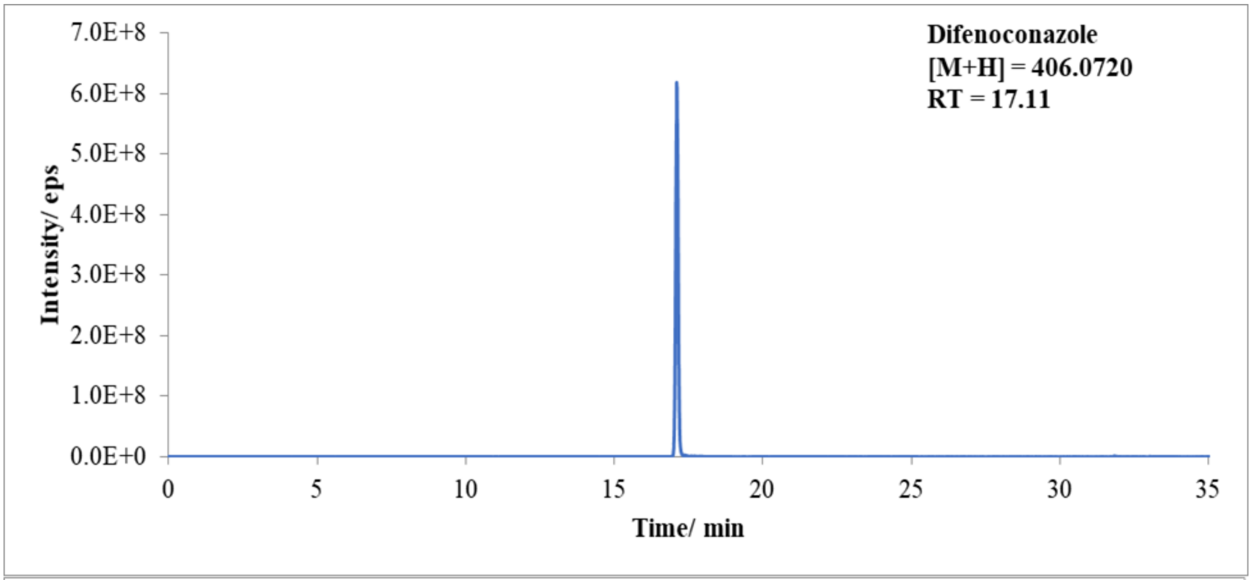
Difenoconazole
[M+H] = 406.0720
RT = 17.11
Intensity/ eps
Time/ min
7.0E+8
6.0E+8
5.0E+8
4.0E+8
3.0E+8
2.0E+8
1.0E+8
0.0E+0
0
5
10
15
20
25
30
35

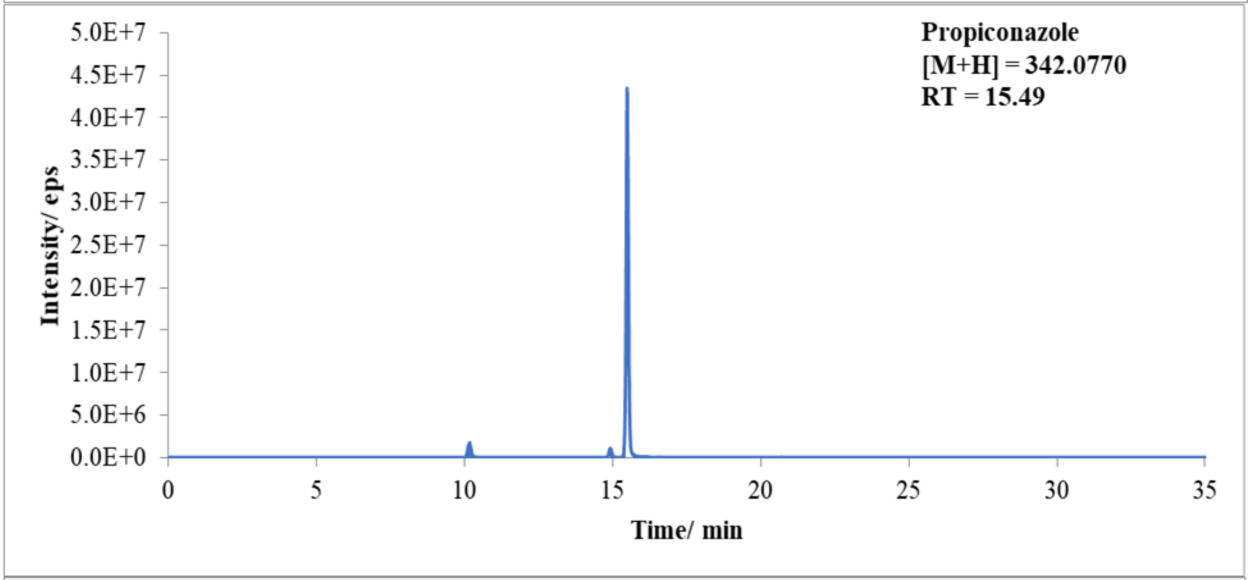
Propiconazole
[M+H] = 342.0770
RT = 15.49
Intensity/ eps
Time/ min
5.0E+7
4.5E+7
4.0E+7
3.5E+7
3.0E+7
2.5E+7
2.0E+7
1.5E+7
1.0E+7
5.0E+6
0.0E+0
0
5
10
15
20
25
30
35

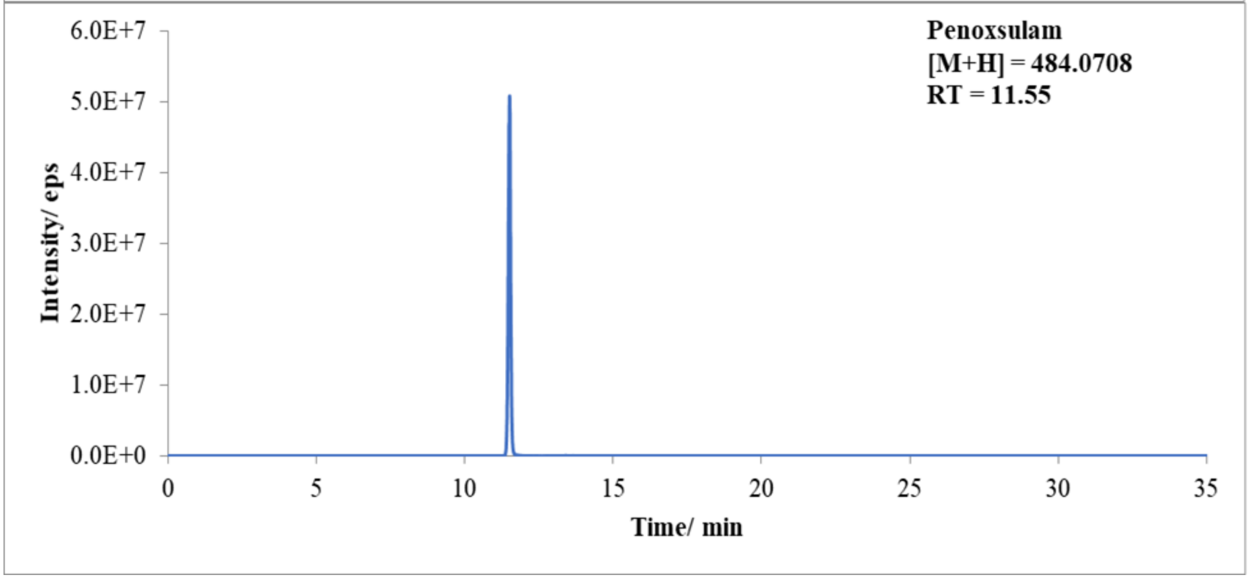
Penoxsulam
[M+H] = 484.0708
RT = 11.55
Intensity/ eps
Time/ min
6.0E+7
5.0E+7
4.0E+7
3.0E+7
2.0E+7
1.0E+7
0.0E+0
0
5
10
15
20
25
30
35

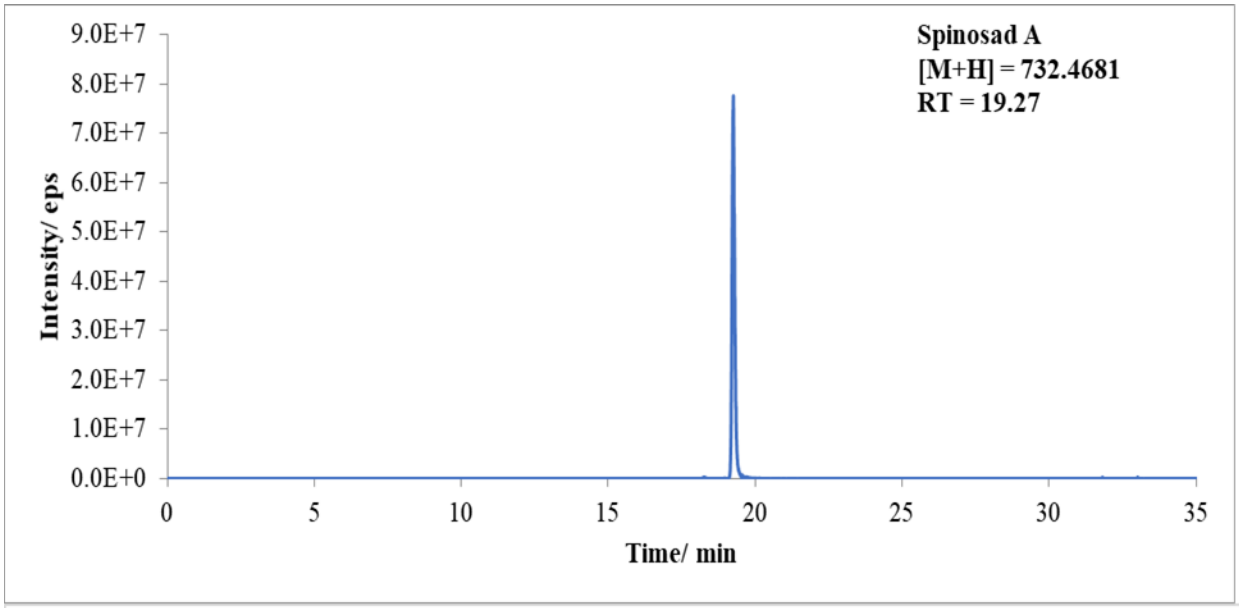
Spinosad A
[M+H] = 732.4681
RT = 19.27
Intensity/ eps
Time/ min
9.0E+7
8.0E+7
7.0E+7
6.0E+7
5.0E+7
4.0E+7
3.0E+7
2.0E+7
1.0E+7
0.0E+0
0
5
10
15
20
25
30
35

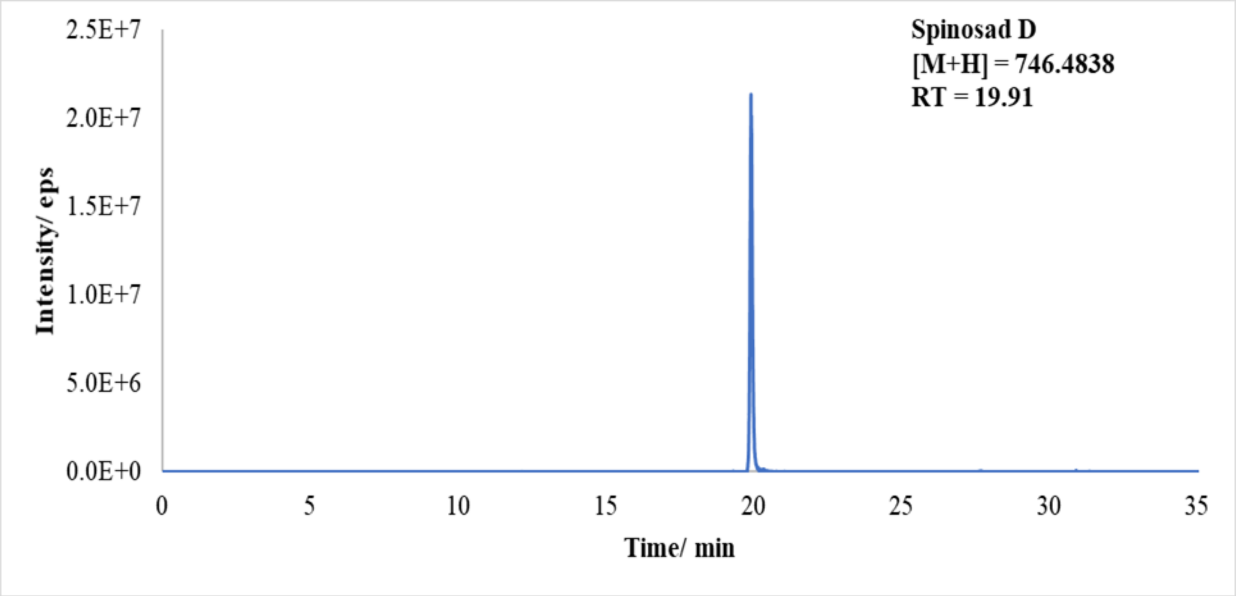
Spinosad D
[M+H] = 746.4838
RT = 19.91
Intensity/ eps
Time/ min
2.5E+7
2.0E+7
1.5E+7
1.0E+7
5.0E+6
0.0E+0
0
5
10
15
20
25
30
35

Fig. S2. The total ion chromatogram of the target analytes in the standard solution (negative mode)

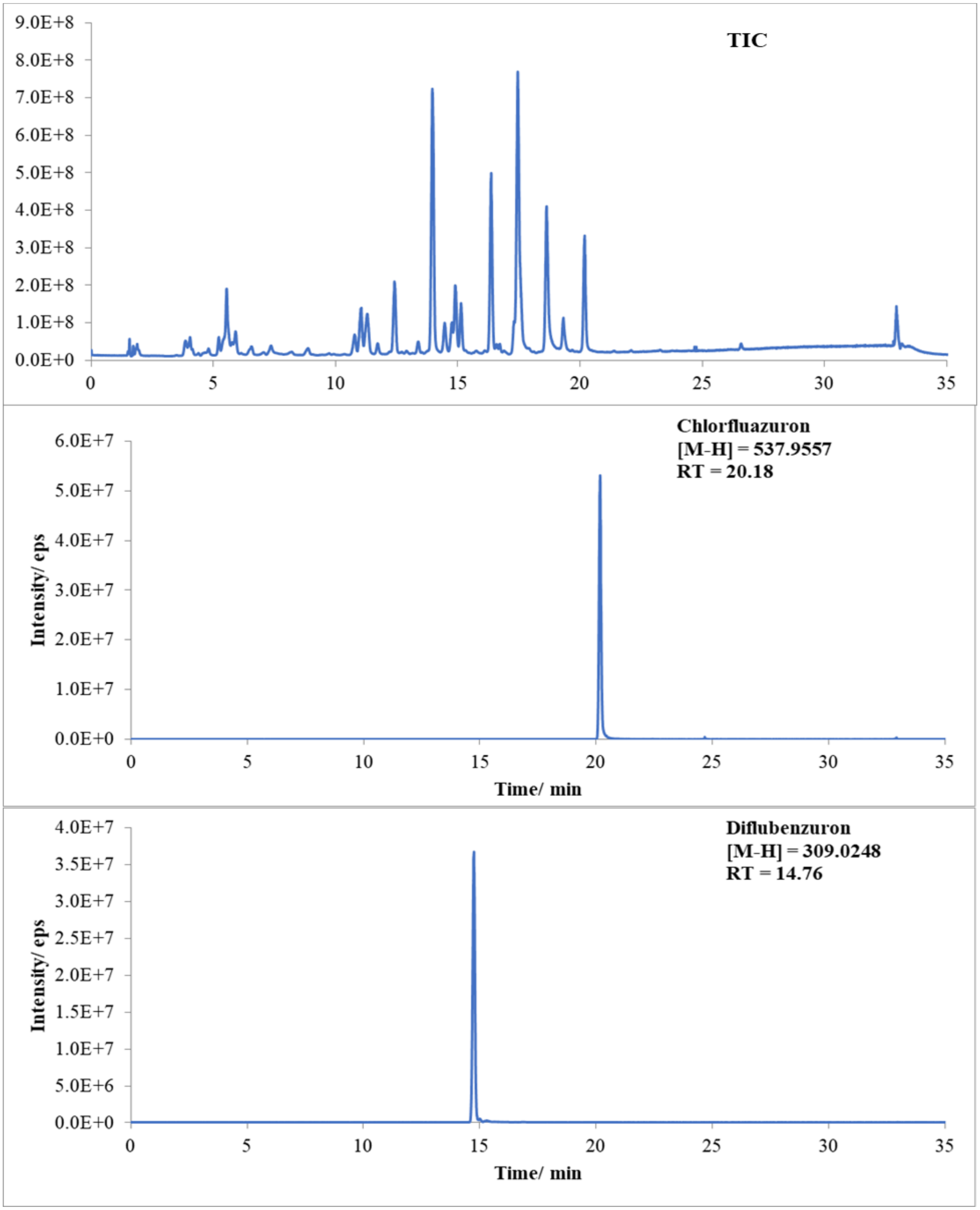

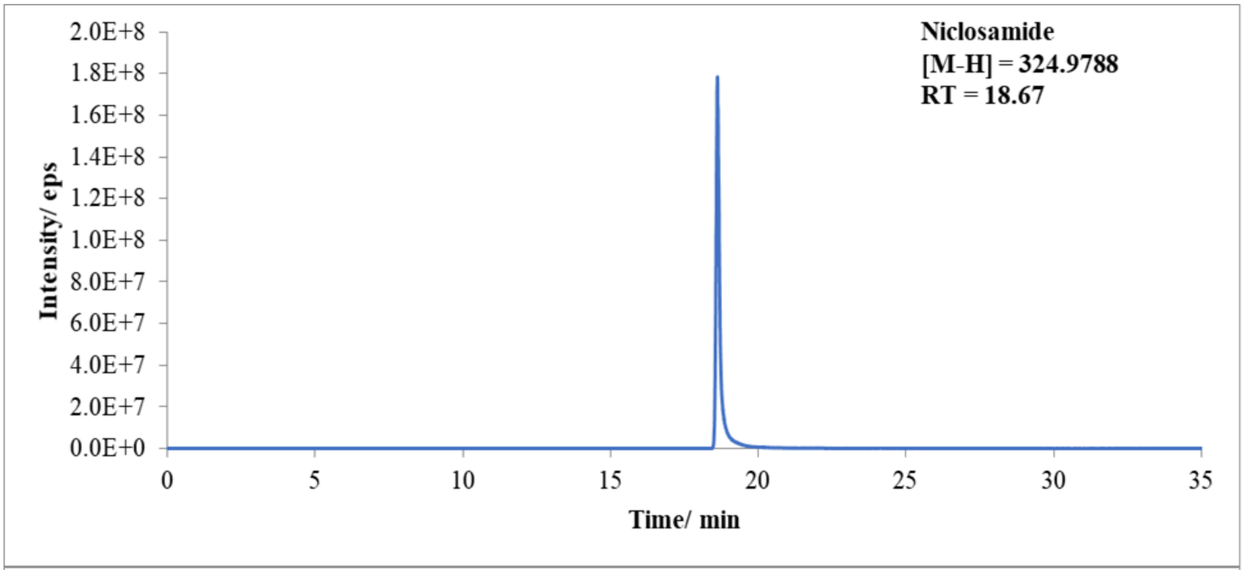
Niclosamide
[M-H] = 324.9788
RT = 18.67
Intensity/ eps
2.0E+8
1.8E+8
1.6E+8
1.4E+8
1.2E+8
1.0E+8
8.0E+7
6.0E+7
4.0E+7
2.0E+7
0.0E+0
0
5
10
15
20
25
30
35
Time/ min

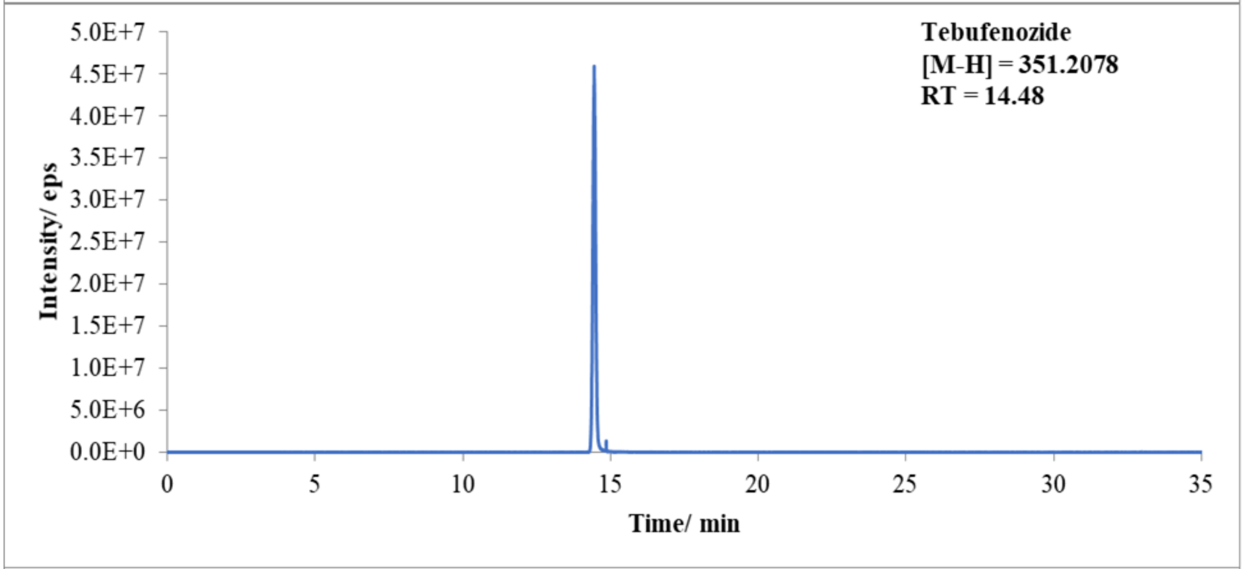
Tebufenozide
[M-H] = 351.2078
RT = 14.48
Intensity/ eps
5.0E+7
4.5E+7
4.0E+7
3.5E+7
3.0E+7
2.5E+7
2.0E+7
1.5E+7
1.0E+7
5.0E+6
0.0E+0
0
5
10
15
20
25
30
35
Time/ min

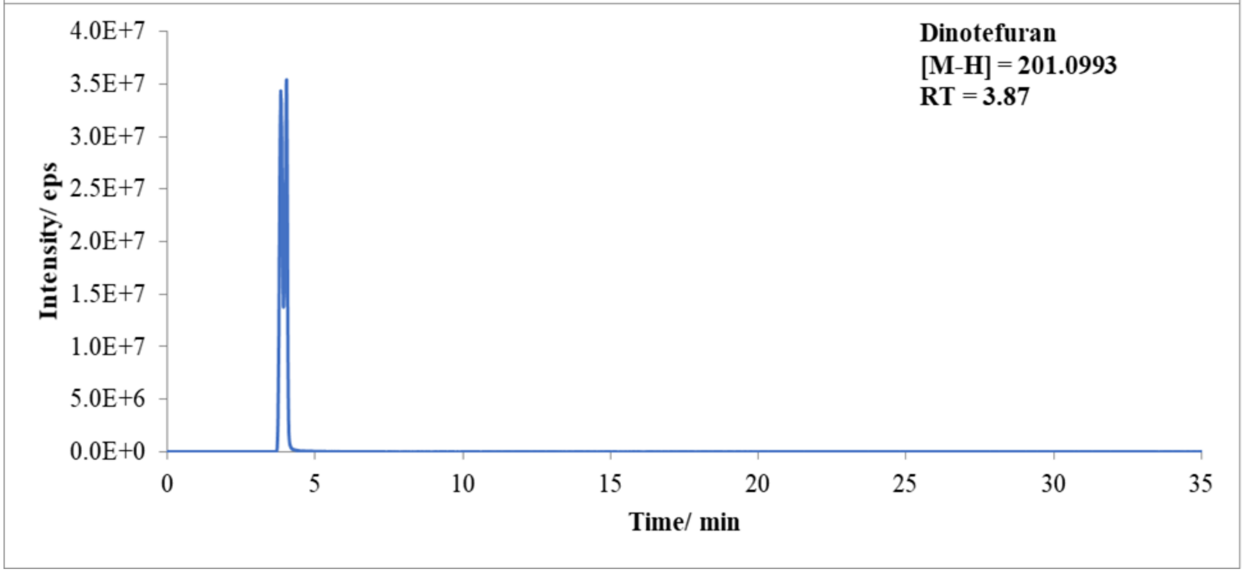
Dinotefuran
[M-H] = 201.0993
RT = 3.87
Intensity/ eps
4.0E+7
3.5E+7
3.0E+7
2.5E+7
2.0E+7
1.5E+7
1.0E+7
5.0E+6
0.0E+0
0
5
10
15
20
25
30
35
Time/ min

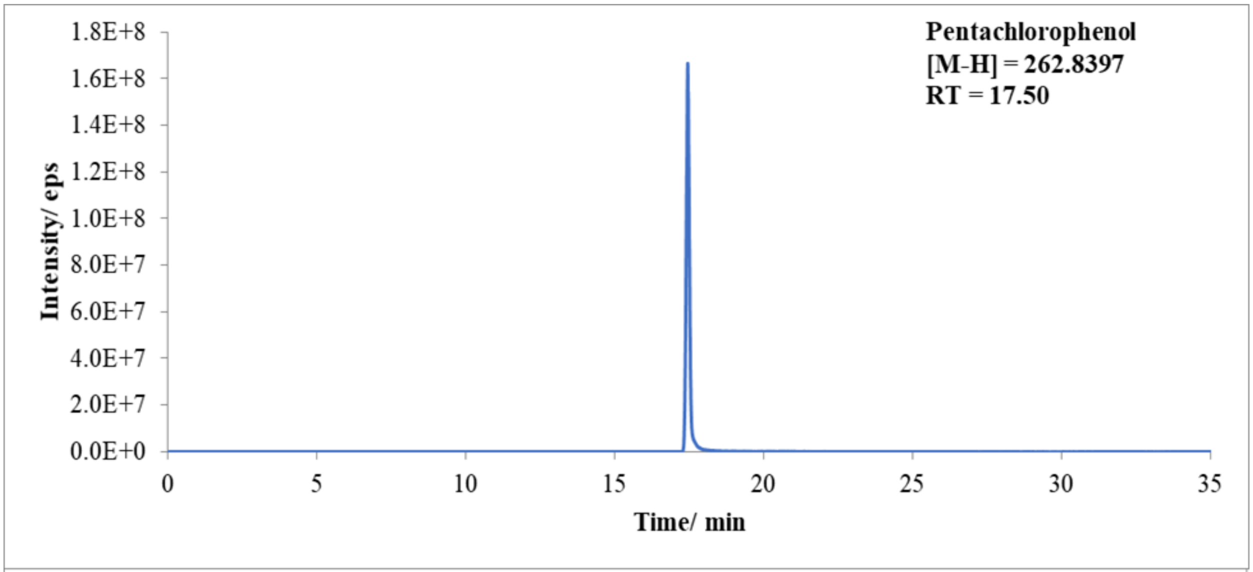
Pentachlorophenol
[M-H] = 262.8397
RT = 17.50
Intensity/ eps
Time/ min

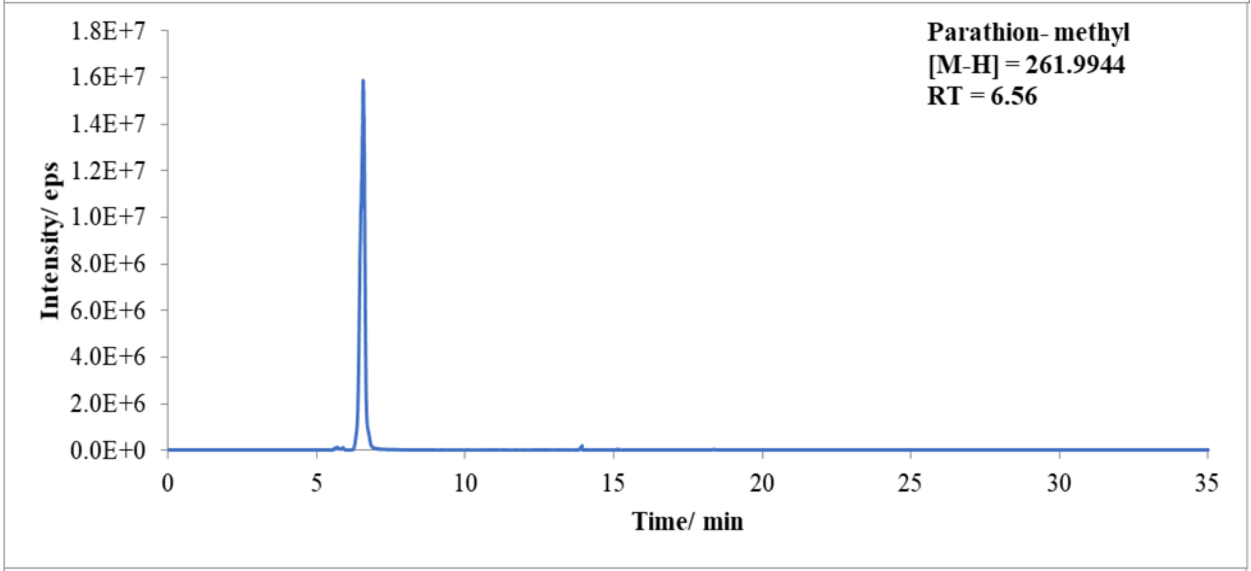
Parathion- methyl
[M-H] = 261.9944
RT = 6.56
Intensity/ eps
Time/ min

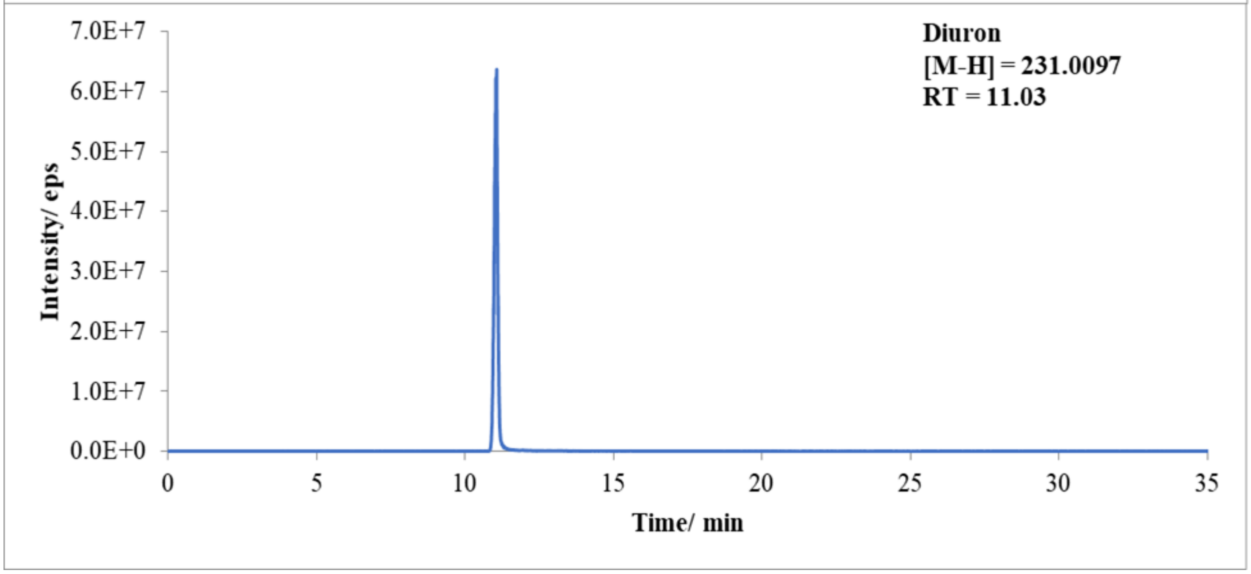
Diuron
[M-H] = 231.0097
RT = 11.03
Intensity/ eps
Time/ min

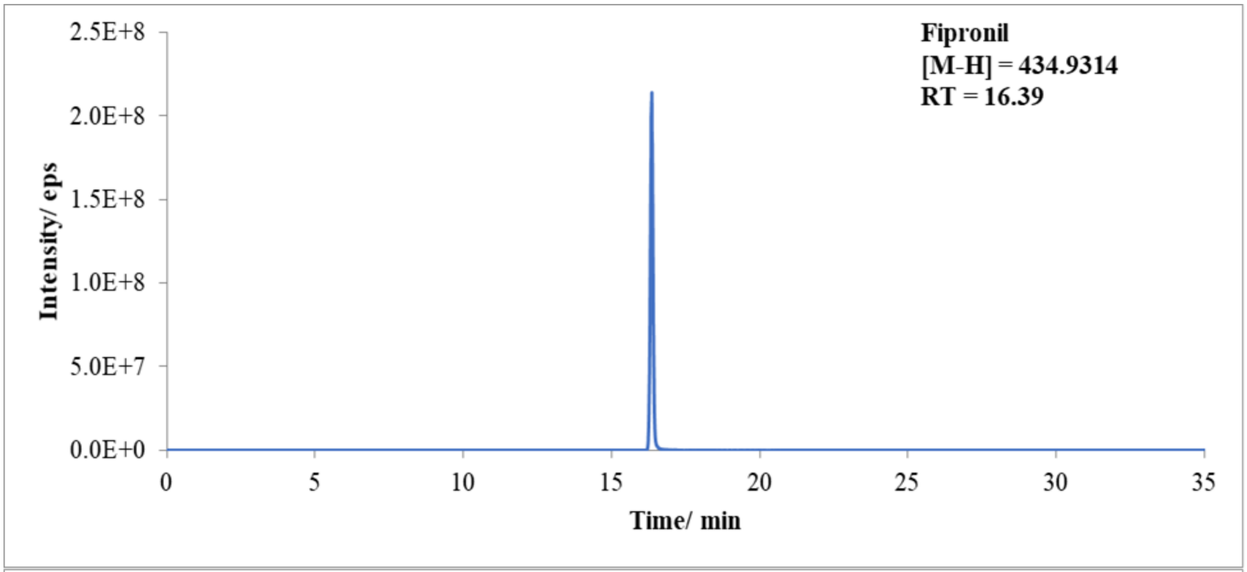
Fipronil
[M-H] = 434.9314
RT = 16.39
Intensity/ eps
2.5E+8
2.0E+8
1.5E+8
1.0E+8
5.0E+7
0.0E+0
0
5
10
15
20
25
30
35
Time/ min

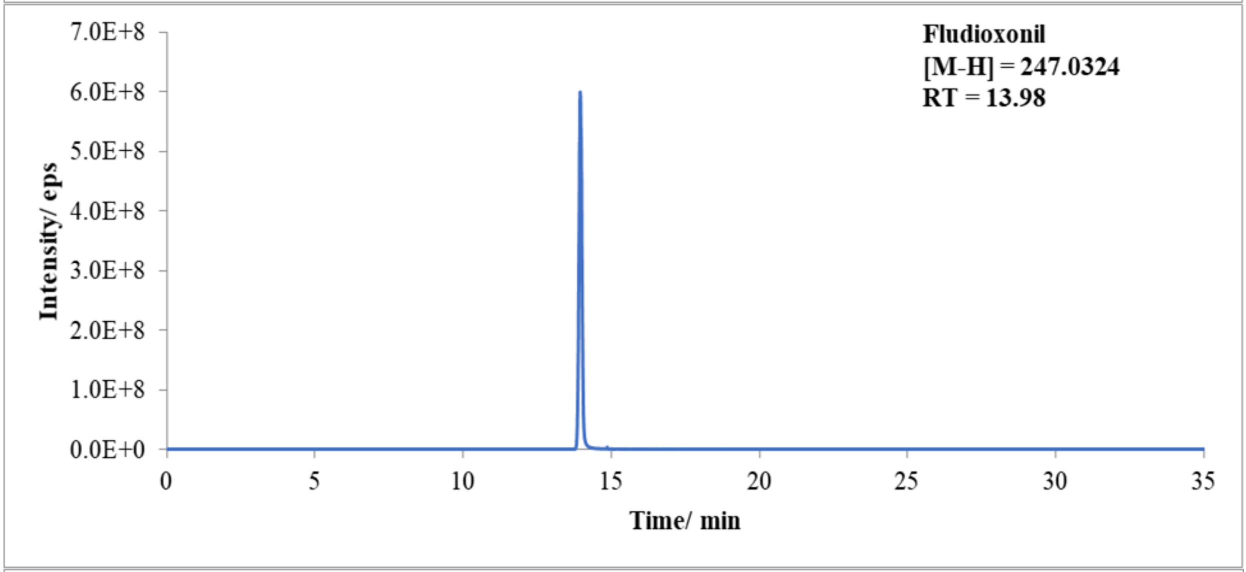
Fludioxonil
[M-H] = 247.0324
RT = 13.98
Intensity/ eps
7.0E+8
6.0E+8
5.0E+8
4.0E+8
3.0E+8
2.0E+8
1.0E+8
0.0E+0
0
5
10
15
20
25
30
35
Time/ min

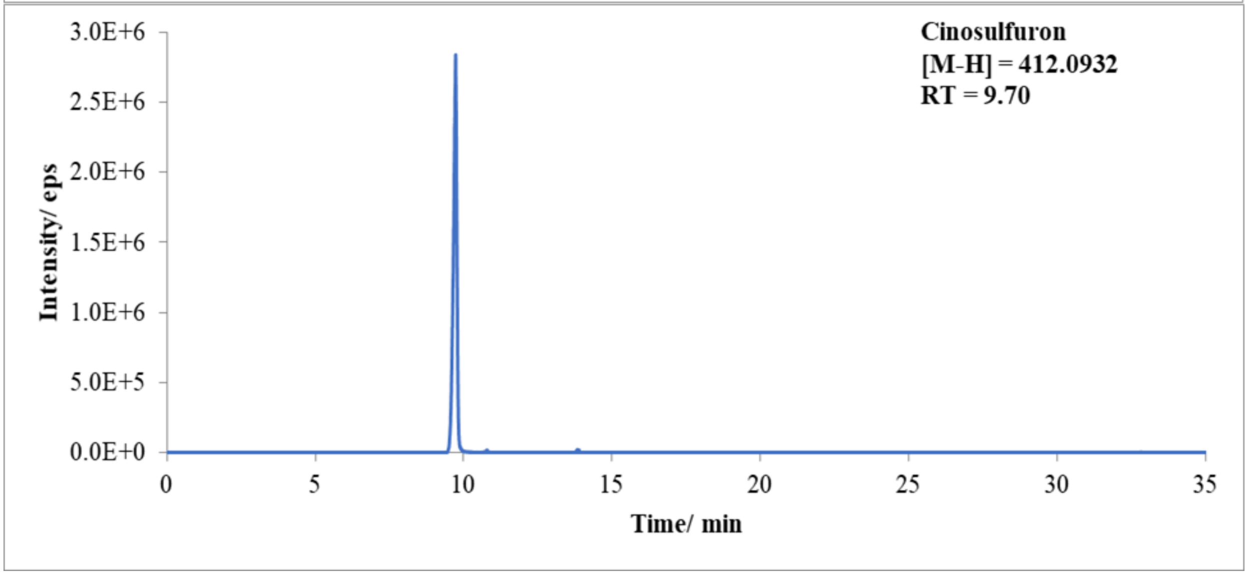
Cinosulfuron
[M-H] = 412.0932
RT = 9.70
Intensity/ eps
3.0E+6
2.5E+6
2.0E+6
1.5E+6
1.0E+6
5.0E+5
0.0E+0
0
5
10
15
20
25
30
35
Time/ min

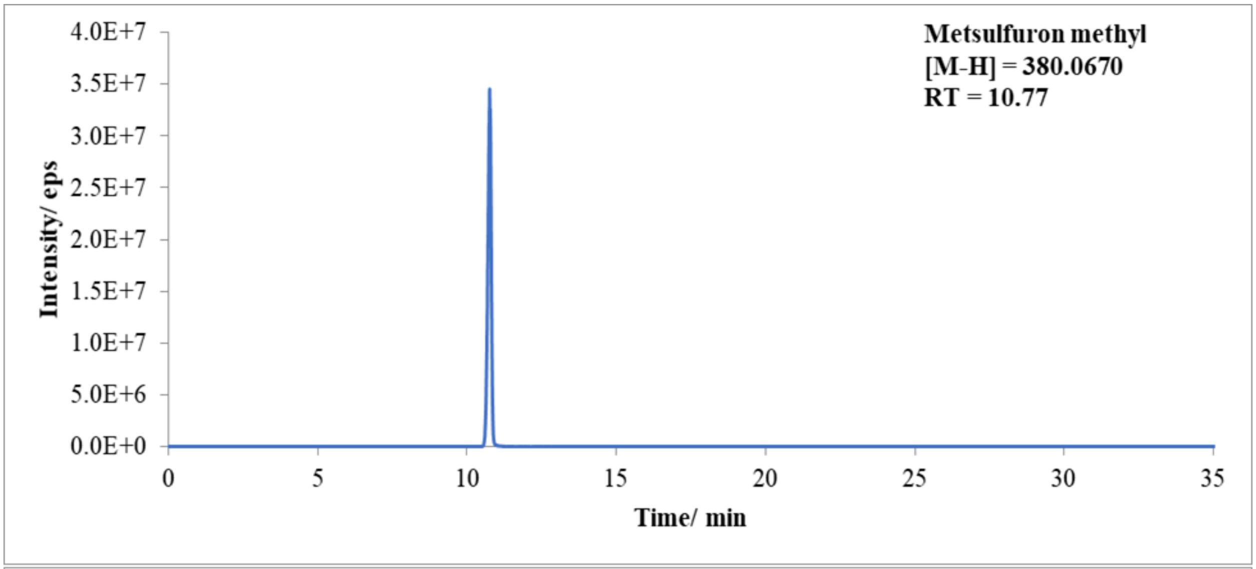

Metsulfuron methyl
[M-H] = 380.0670
RT = 10.77
Intensity/ eps
Time/ min

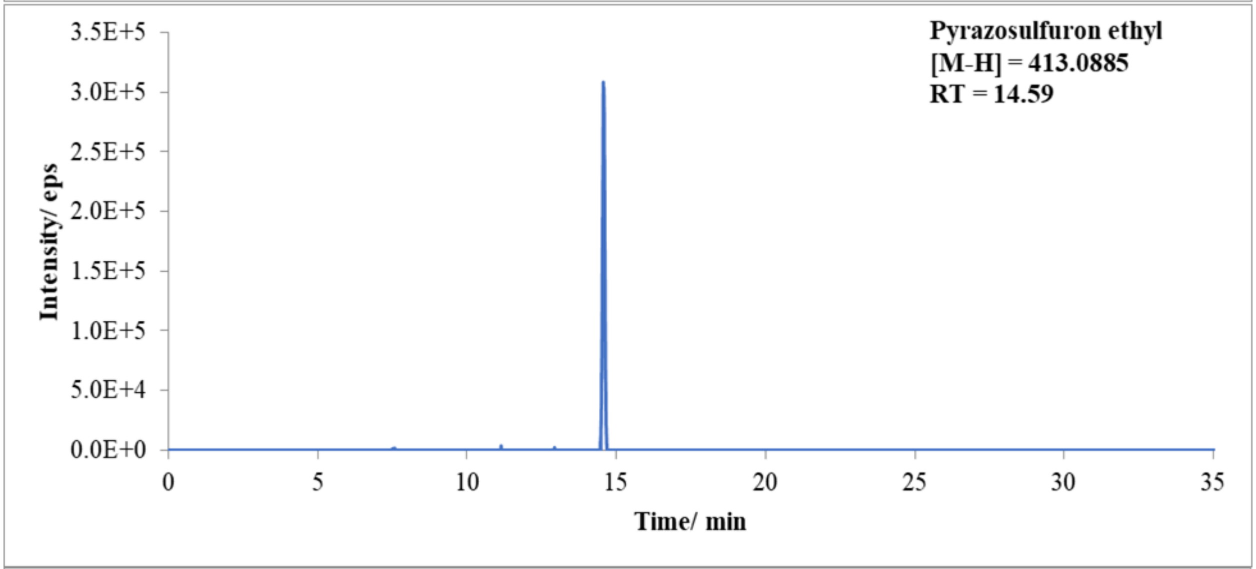

Pyrazosulfuron ethyl
[M-H] = 413.0885
RT = 14.59
Intensity/ eps
Time/ min

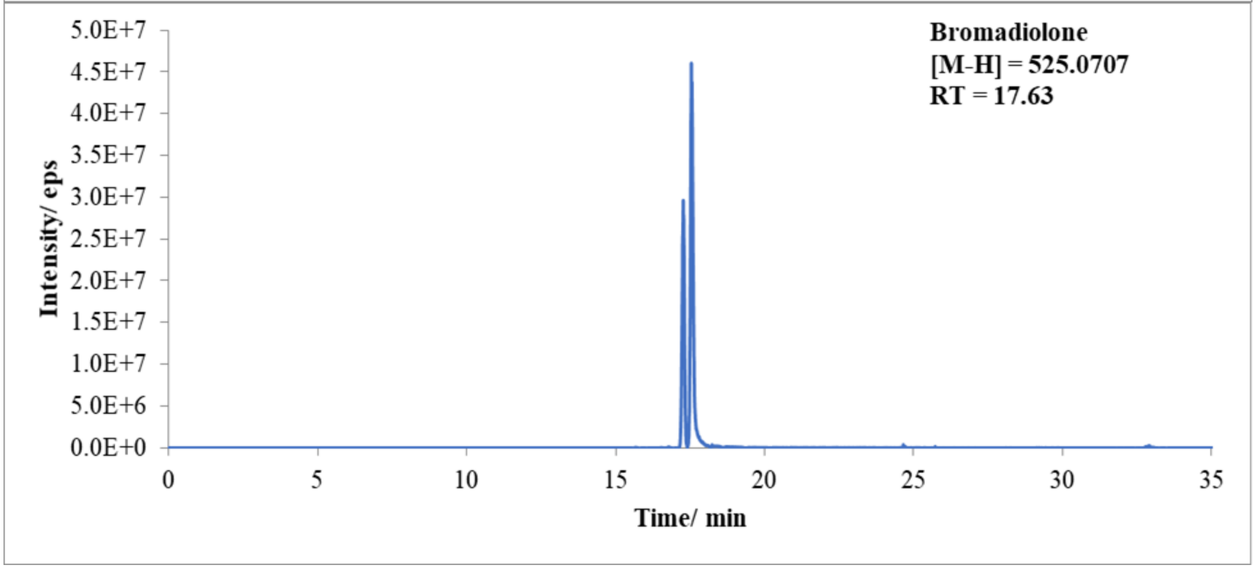

Bromadiolone
[M-H] = 525.0707
RT = 17.63
Intensity/ eps
Time/ min

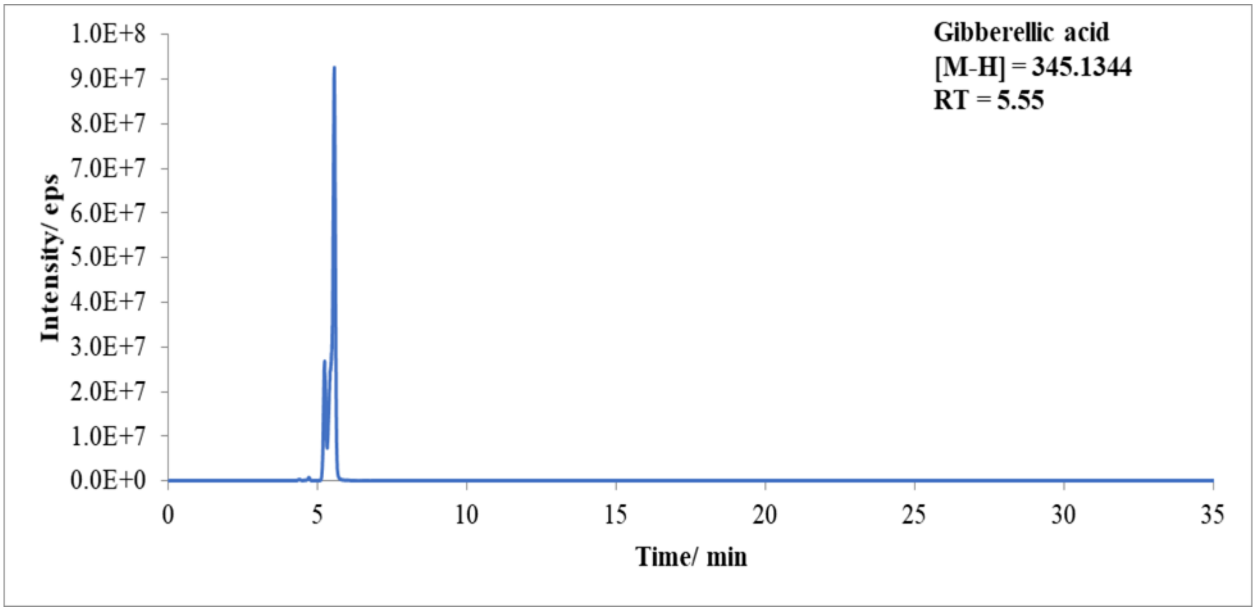
Gibberellic acid
[M-H] = 345.1344
RT = 5.55
Intensity/ eps
1.0E+8
9.0E+7
8.0E+7
7.0E+7
6.0E+7
5.0E+7
4.0E+7
3.0E+7
2.0E+7
1.0E+7
0.0E+0
0
5
10
15
20
25
30
35
Time/ min

Fig. S3. The total ion chromatogram and extracted ion chromatogram of the target analytes in the matrix match solution (positive mode)

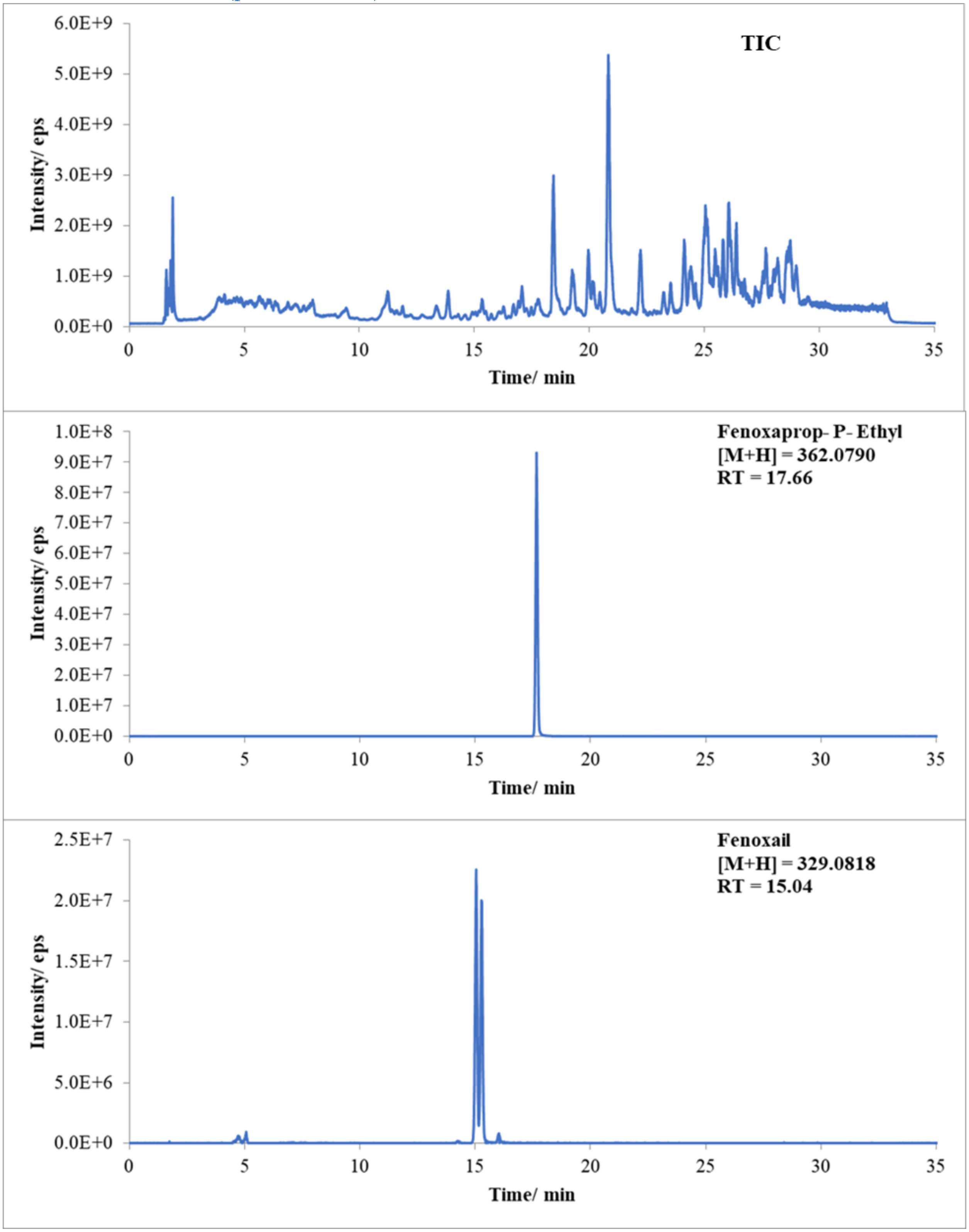

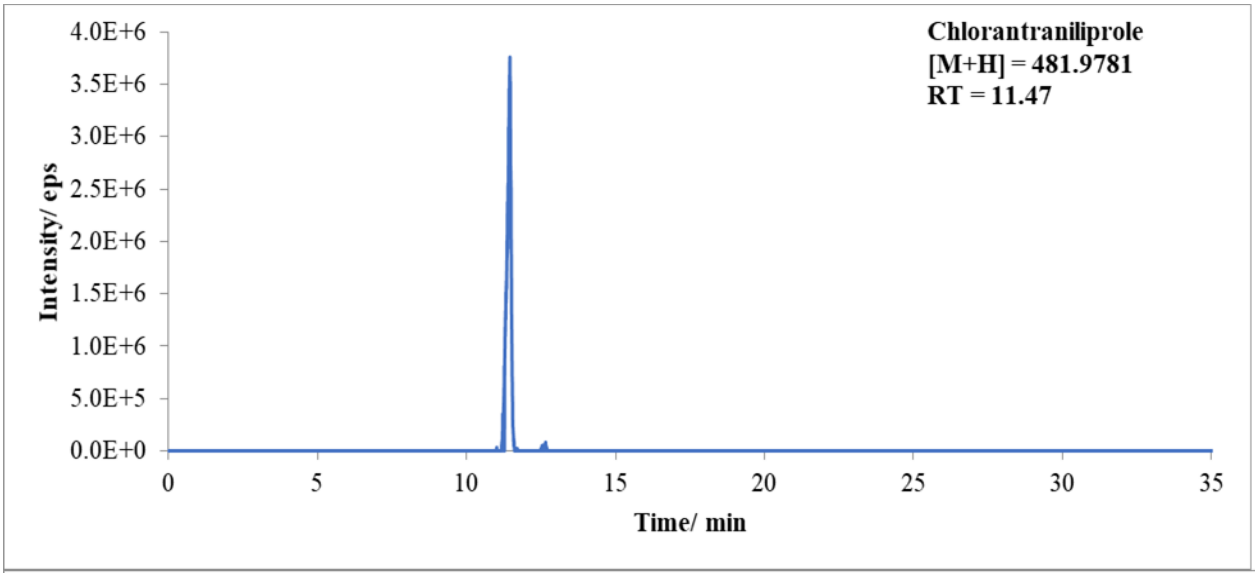
Chlorantraniliprole
[M+H] = 481.9781
RT = 11.47
Intensity/ eps
Time/ min
4.0E+6
3.5E+6
3.0E+6
2.5E+6
2.0E+6
1.5E+6
1.0E+6
5.0E+5
0.0E+0
0
5
10
15
20
25
30
35

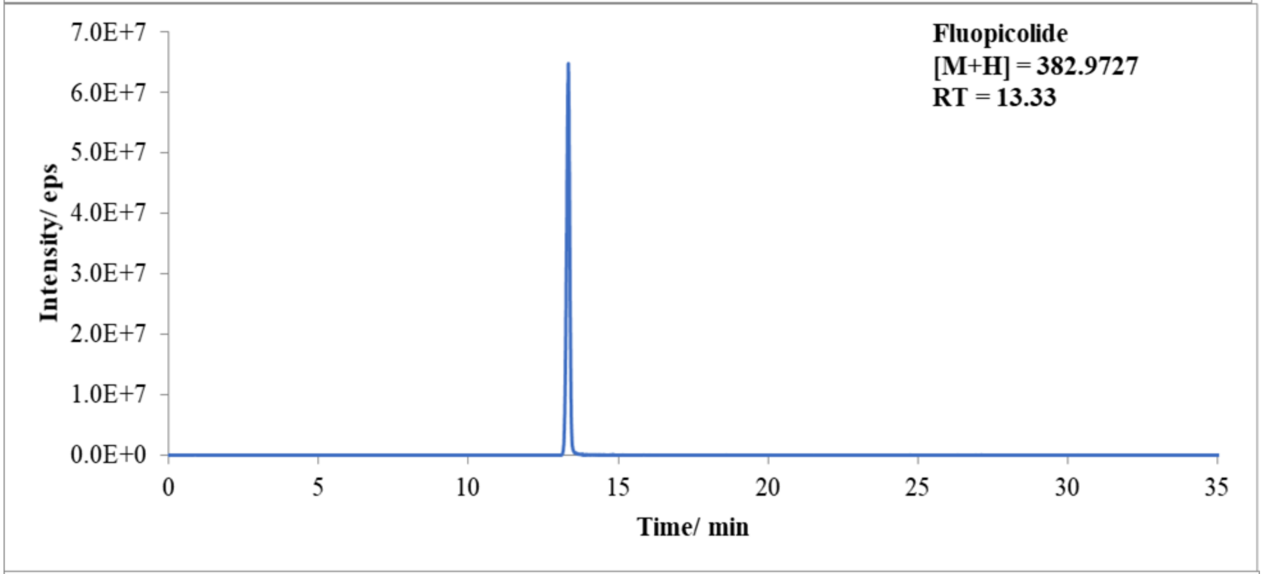
Fluopicolide
[M+H] = 382.9727
RT = 13.33
Intensity/ eps
Time/ min
7.0E+7
6.0E+7
5.0E+7
4.0E+7
3.0E+7
2.0E+7
1.0E+7
0.0E+0
0
5
10
15
20
25
30
35

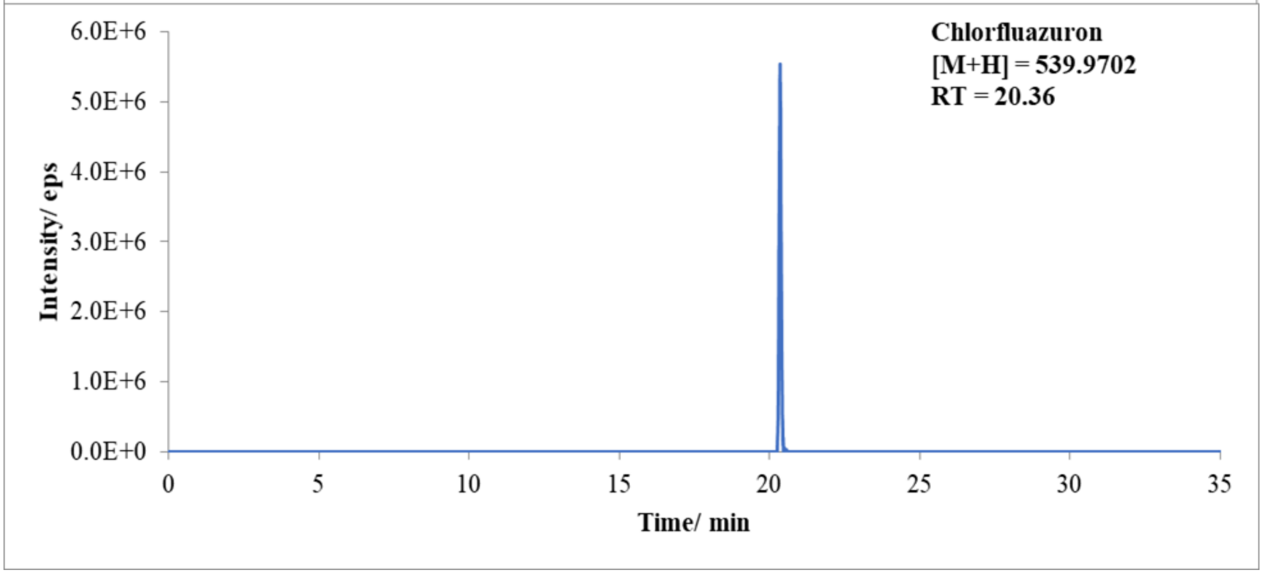
Chlorfluazuron
[M+H] = 539.9702
RT = 20.36
Intensity/ eps
Time/ min
6.0E+6
5.0E+6
4.0E+6
3.0E+6
2.0E+6
1.0E+6
0.0E+0
0
5
10
15
20
25
30
35

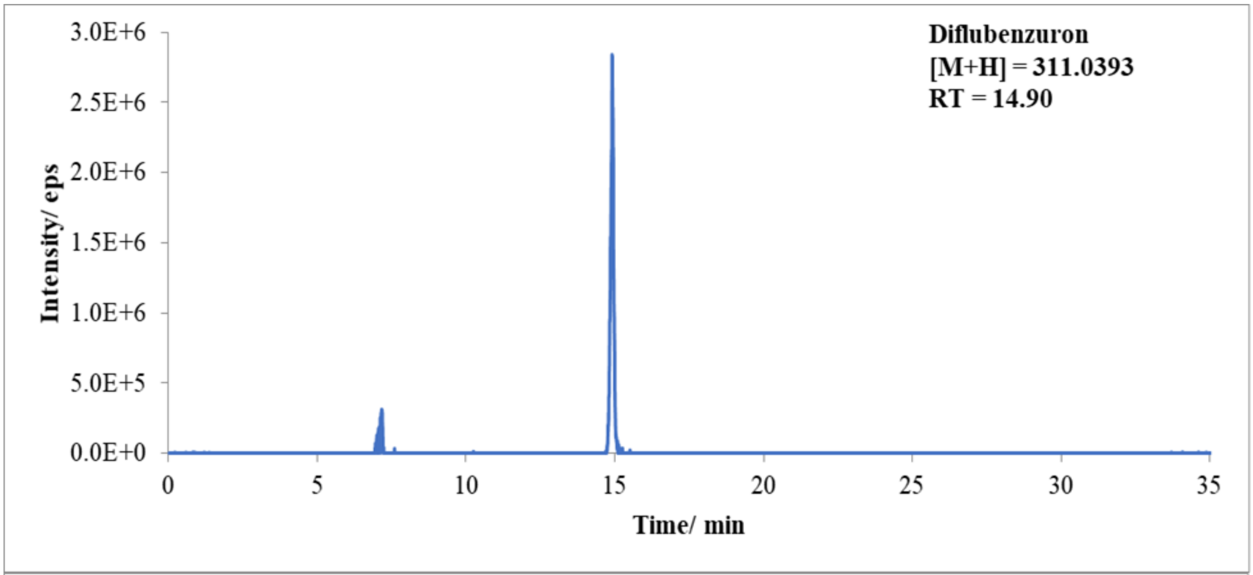
Diflubenzuron
[M+H] = 311.0393
RT = 14.90
Intensity/ eps
Time/ min
3.0E+6
2.5E+6
2.0E+6
1.5E+6
1.0E+6
5.0E+5
0.0E+0
0
5
10
15
20
25
30
35

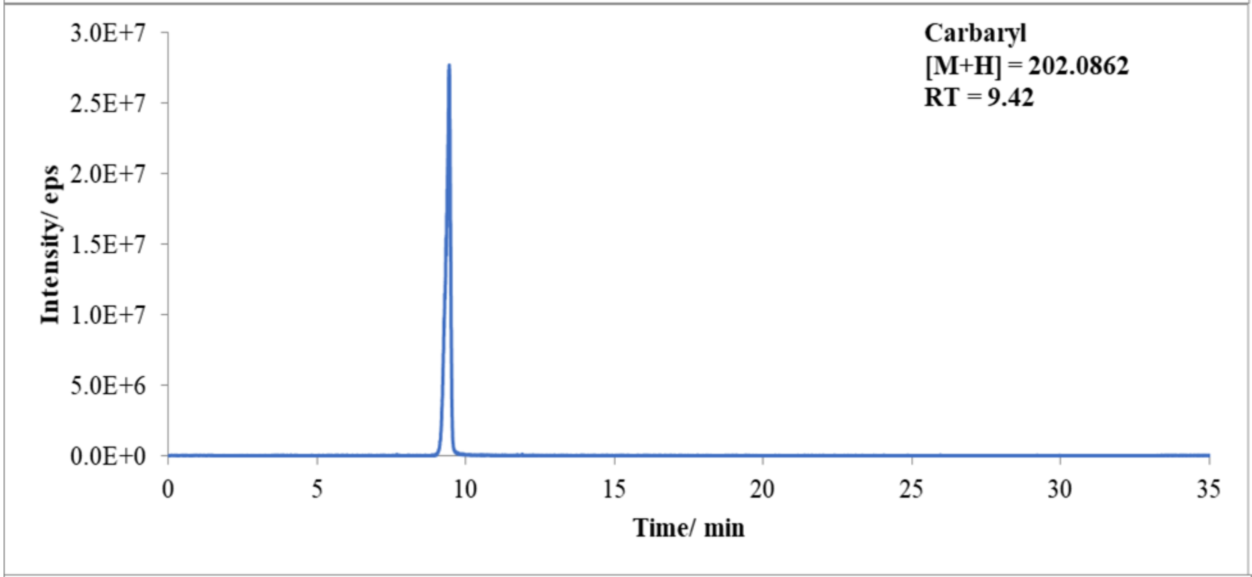
Carbaryl
[M+H] = 202.0862
RT = 9.42
Intensity/ eps
Time/ min
3.0E+7
2.5E+7
2.0E+7
1.5E+7
1.0E+7
5.0E+6
0.0E+0
0
5
10
15
20
25
30
35

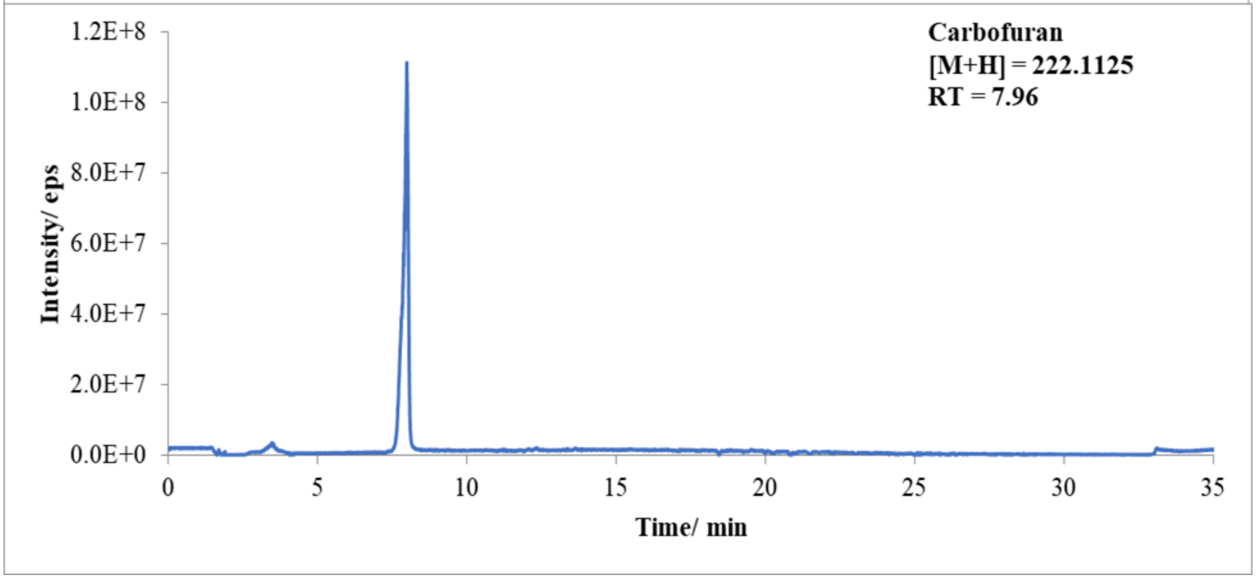
Carbofuran
[M+H] = 222.1125
RT = 7.96
Intensity/ eps
Time/ min
1.2E+8
1.0E+8
8.0E+7
6.0E+7
4.0E+7
2.0E+7
0.0E+0
0
5
10
15
20
25
30
35

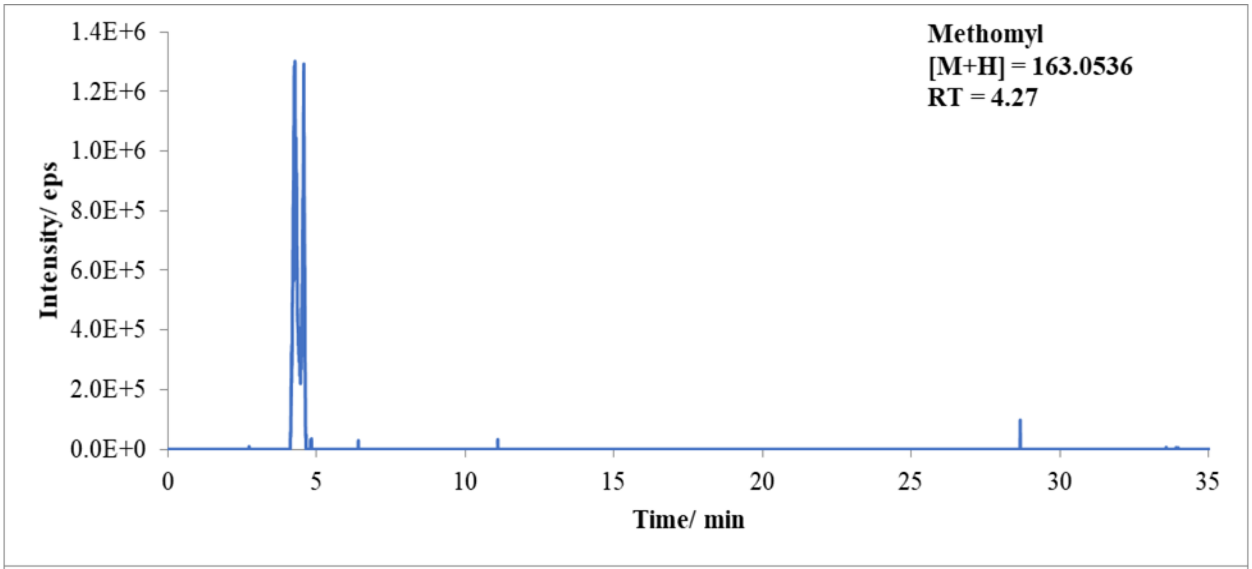
Methomyl
[M+H] = 163.0536
RT = 4.27
Intensity/ eps
Time/ min

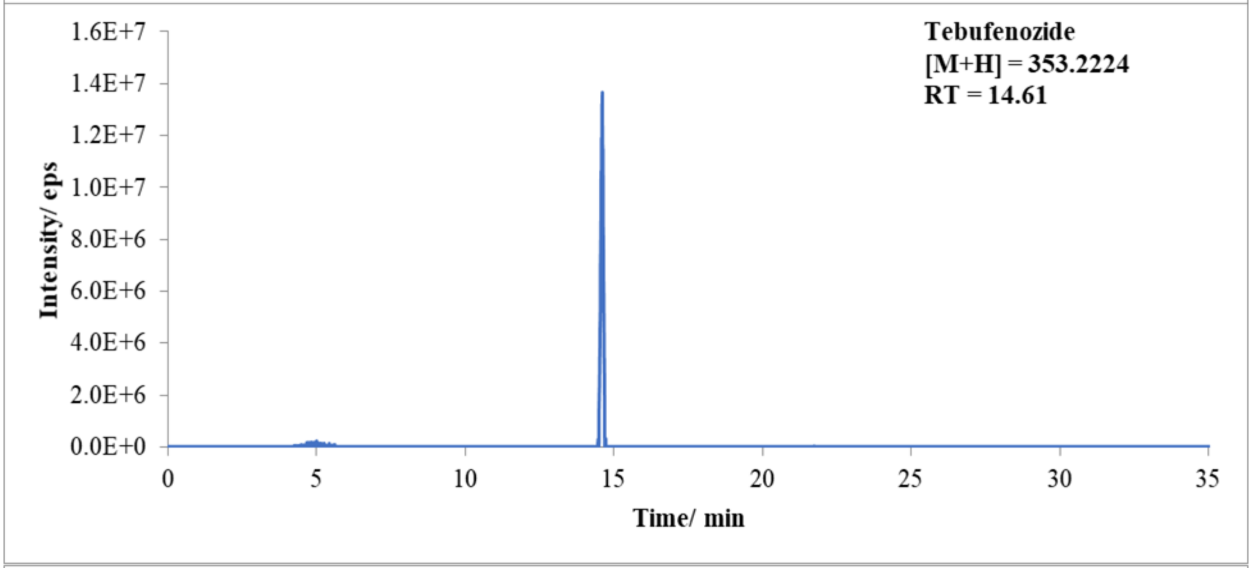
Tebufenozide
[M+H] = 353.2224
RT = 14.61
Intensity/ eps
Time/ min

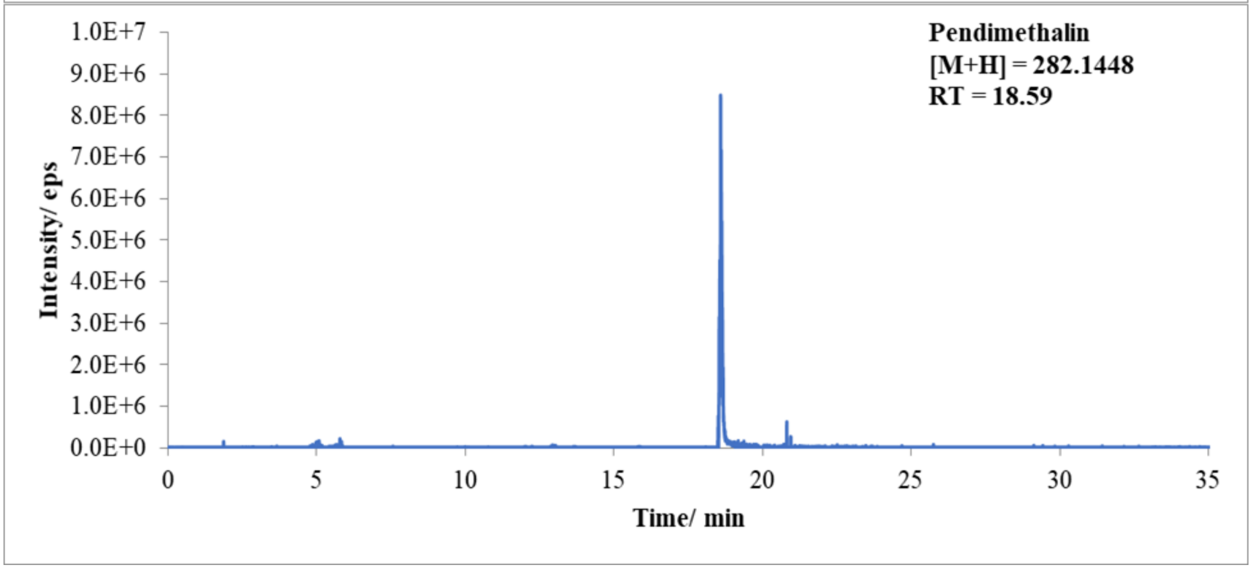
Pendimethalin
[M+H] = 282.1448
RT = 18.59
Intensity/ eps
Time/ min

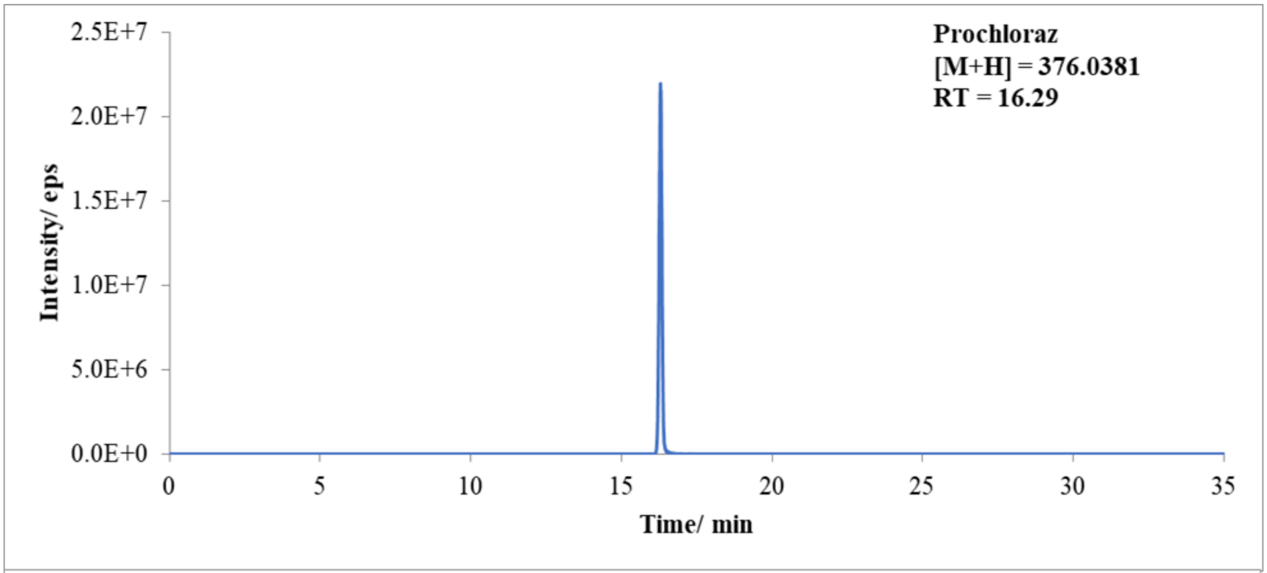
Prochloraz
[M+H] = 376.0381
RT = 16.29
Intensity/ eps
2.5E+7
2.0E+7
1.5E+7
1.0E+7
5.0E+6
0.0E+0
0
5
10
15
20
25
30
35
Time/ min

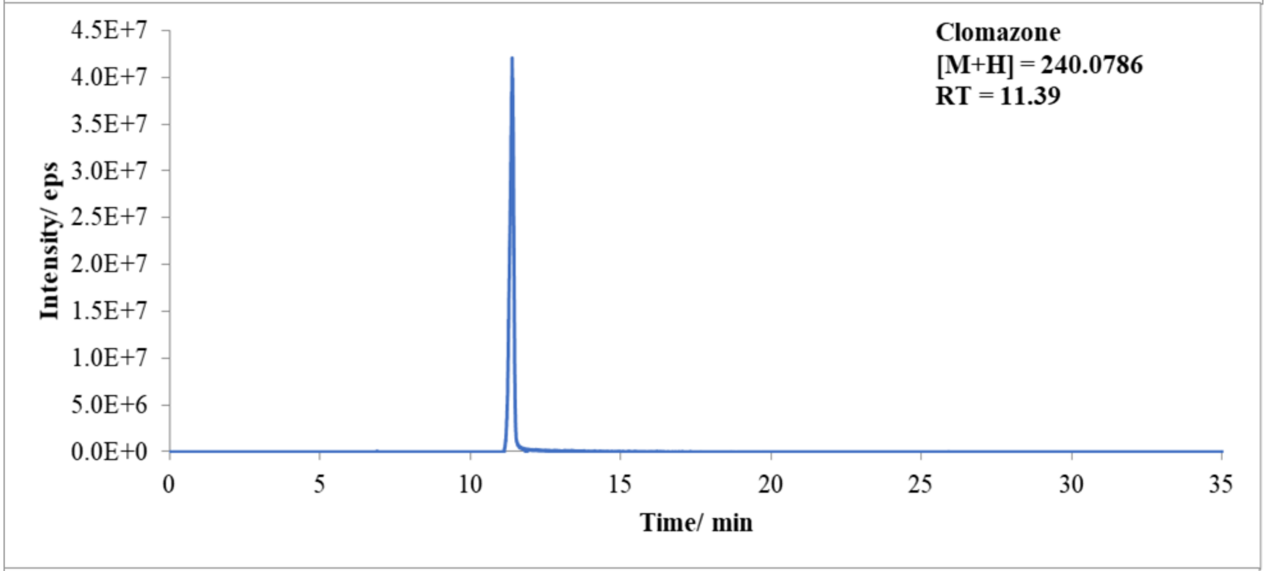
Clomazone
[M+H] = 240.0786
RT = 11.39
Intensity/ eps
4.5E+7
4.0E+7
3.5E+7
3.0E+7
2.5E+7
2.0E+7
1.5E+7
1.0E+7
5.0E+6
0.0E+0
0
5
10
15
20
25
30
35
Time/ min

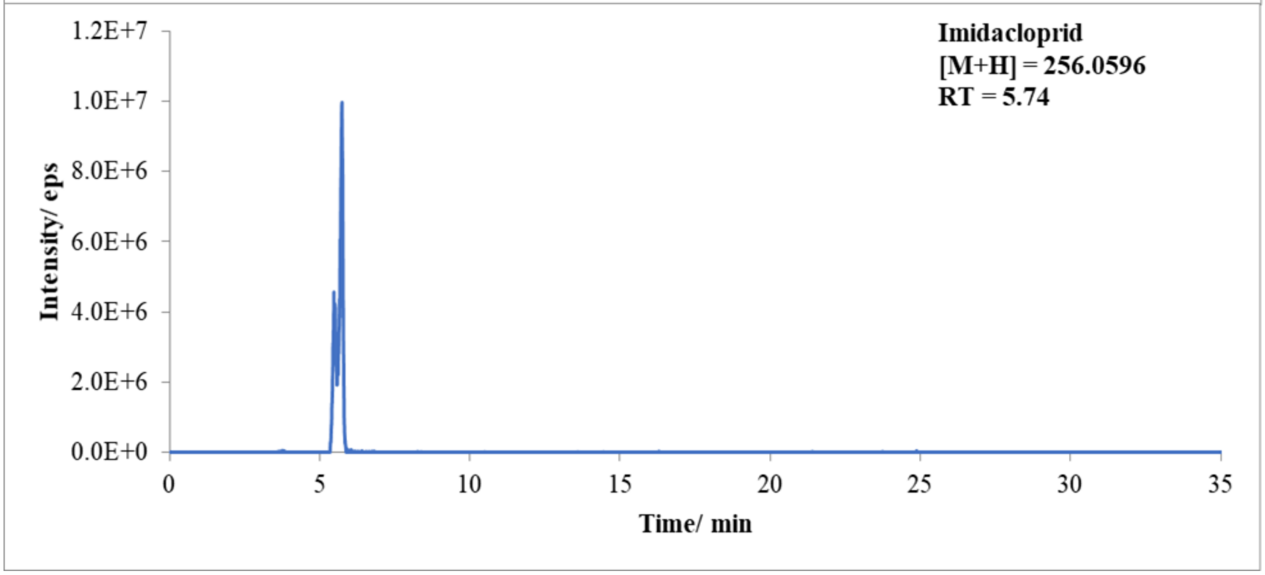
Imidacloprid
[M+H] = 256.0596
RT = 5.74
Intensity/ eps
1.2E+7
1.0E+7
8.0E+6
6.0E+6
4.0E+6
2.0E+6
0.0E+0
0
5
10
15
20
25
30
35
Time/ min

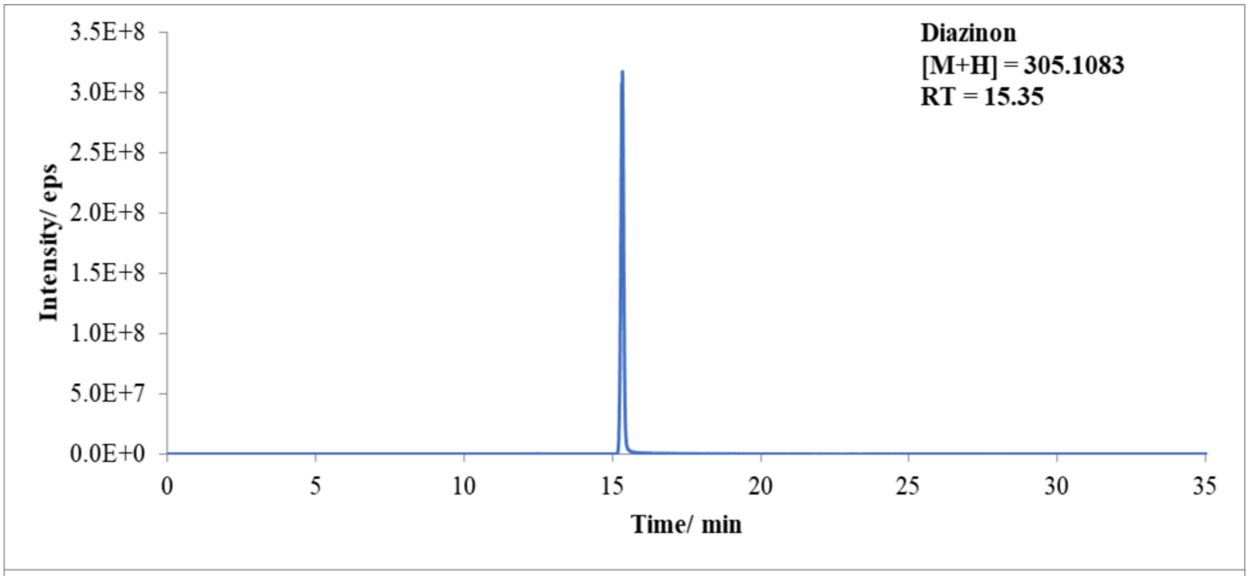
Diazinon
[M+H] = 305.1083
RT = 15.35
Intensity/ eps
Time/ min
3.5E+8
3.0E+8
2.5E+8
2.0E+8
1.5E+8
1.0E+8
5.0E+7
0.0E+0
0
5
10
15
20
25
30
35

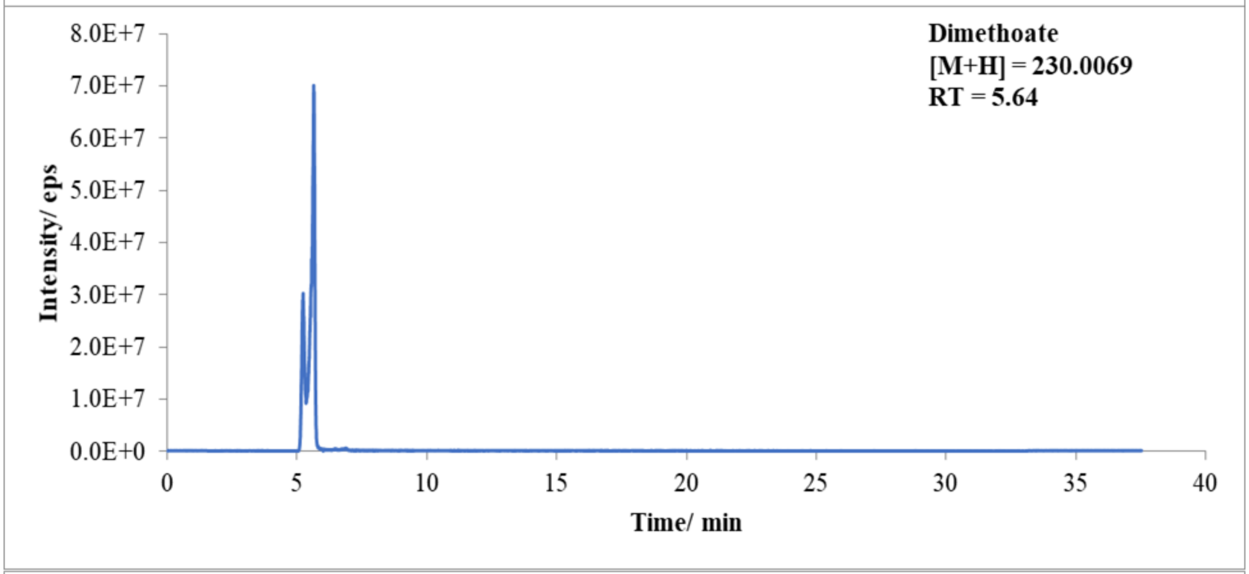
Dimethoate
[M+H] = 230.0069
RT = 5.64
Intensity/ eps
Time/ min
8.0E+7
7.0E+7
6.0E+7
5.0E+7
4.0E+7
3.0E+7
2.0E+7
1.0E+7
0.0E+0
0
5
10
15
20
25
30
35
40

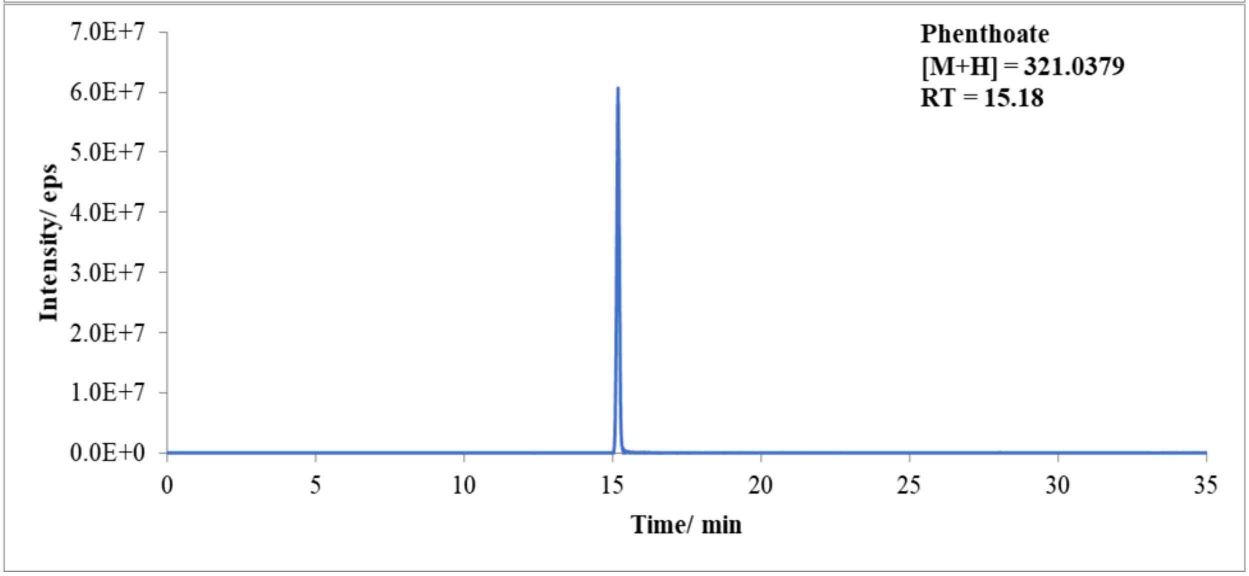
Phenthoate
[M+H] = 321.0379
RT = 15.18
Intensity/ eps
Time/ min
7.0E+7
6.0E+7
5.0E+7
4.0E+7
3.0E+7
2.0E+7
1.0E+7
0.0E+0
0
5
10
15
20
25
30
35

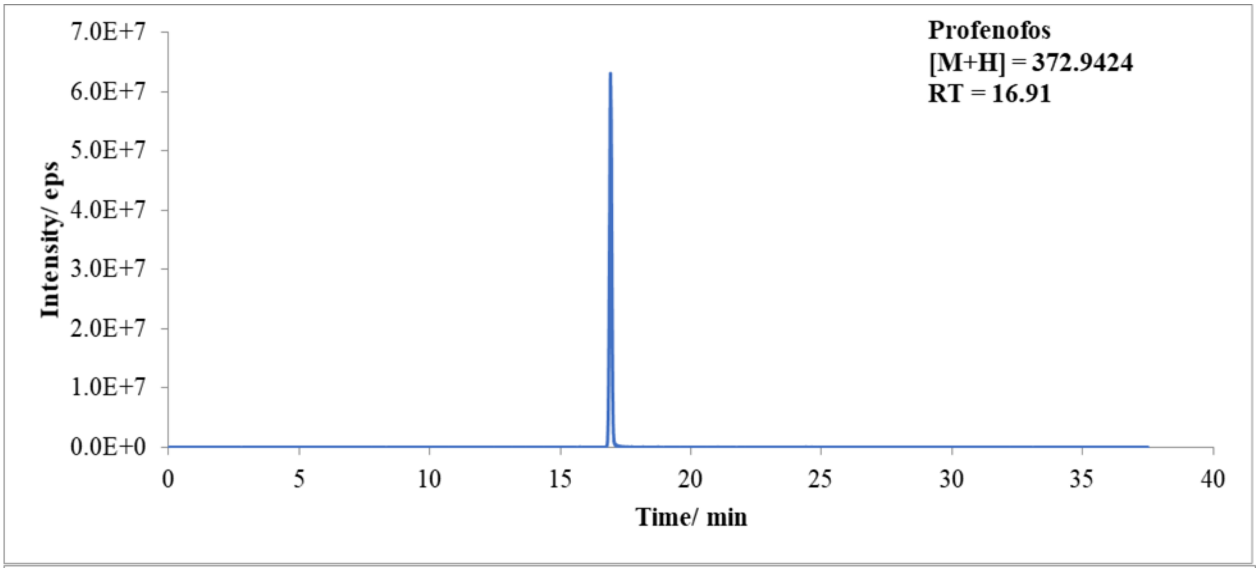
Profenofos
[M+H] = 372.9424
RT = 16.91
Intensity/ eps
Time/ min

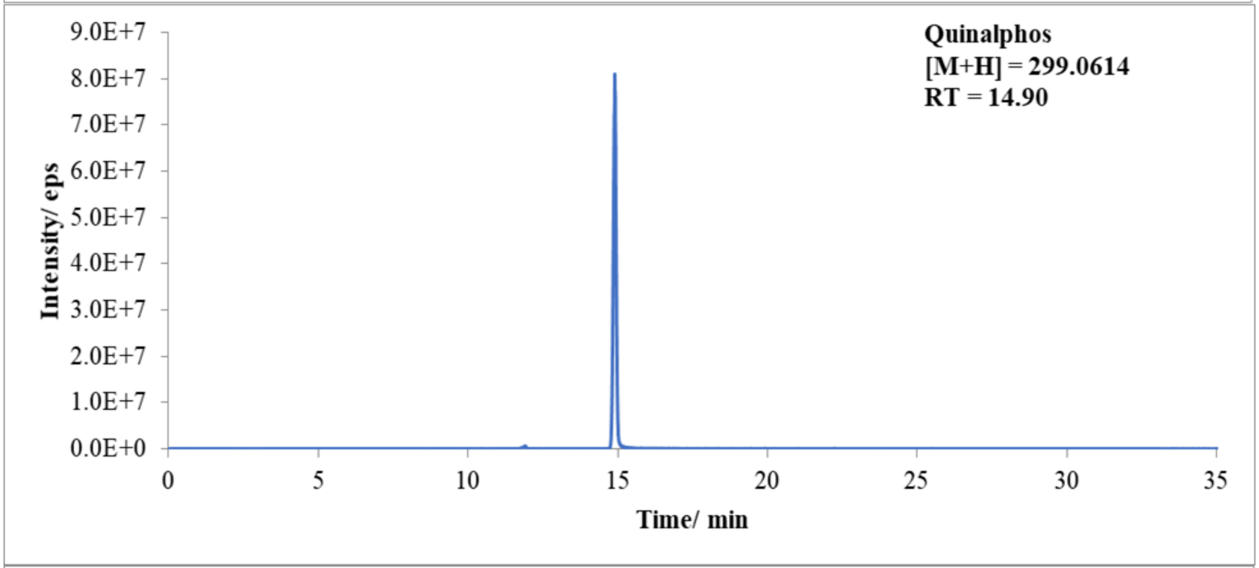
Quinalphos
[M+H] = 299.0614
RT = 14.90
Intensity/ eps
Time/ min

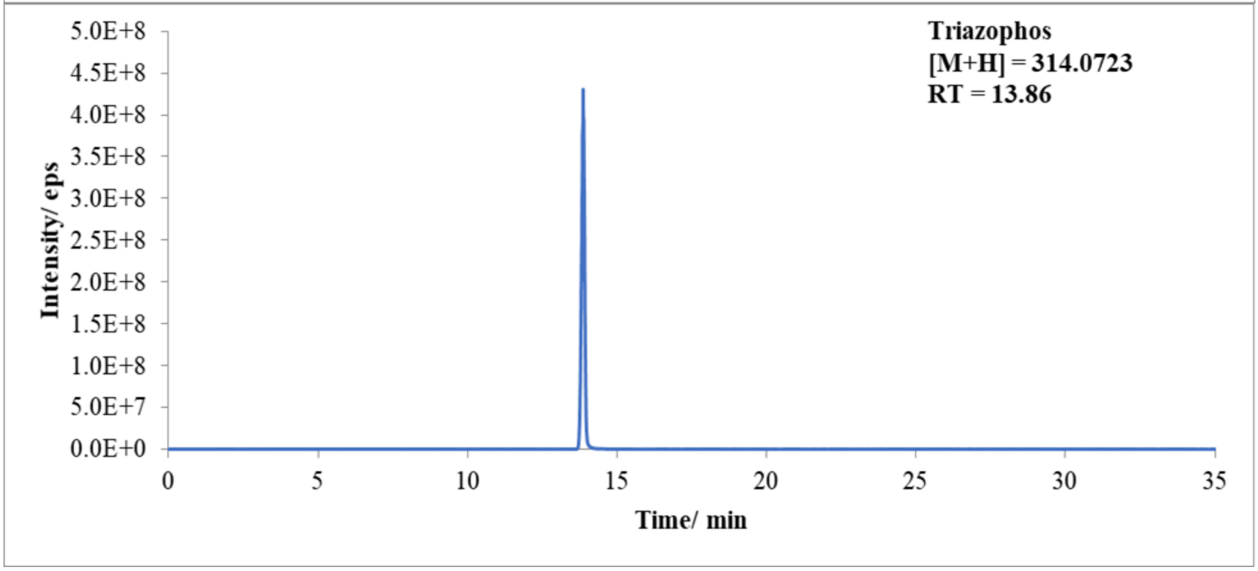
Triazophos
[M+H] = 314.0723
RT = 13.86
Intensity/ eps
Time/ min

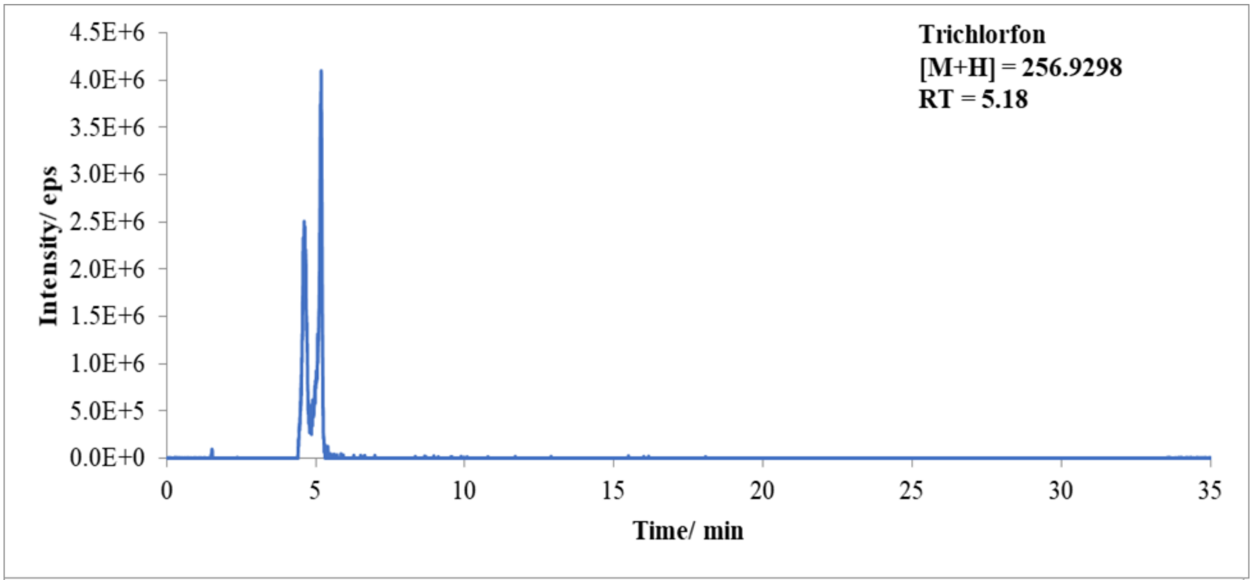

Trichlorfon
[M+H] = 256.9298
RT = 5.18
Intensity/ eps
4.5E+6
4.0E+6
3.5E+6
3.0E+6
2.5E+6
2.0E+6
1.5E+6
1.0E+6
5.0E+5
0.0E+0
0
5
10
15
20
25
30
35
Time/ min

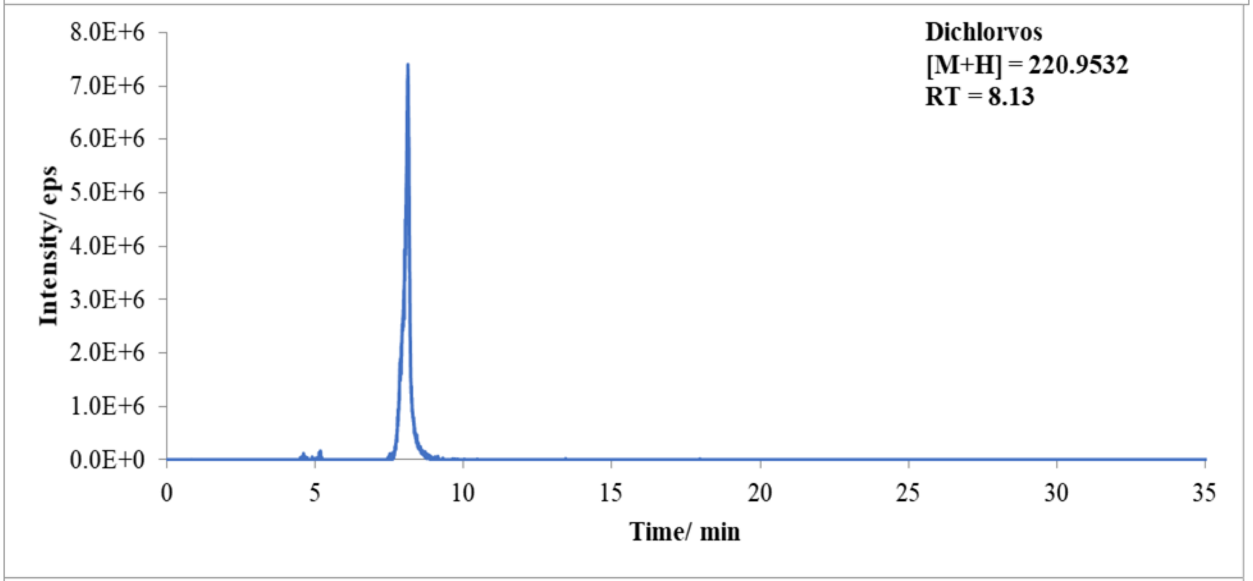

Dichlorvos
[M+H] = 220.9532
RT = 8.13
Intensity/ eps
8.0E+6
7.0E+6
6.0E+6
5.0E+6
4.0E+6
3.0E+6
2.0E+6
1.0E+6
0.0E+0
0
5
10
15
20
25
30
35
Time/ min

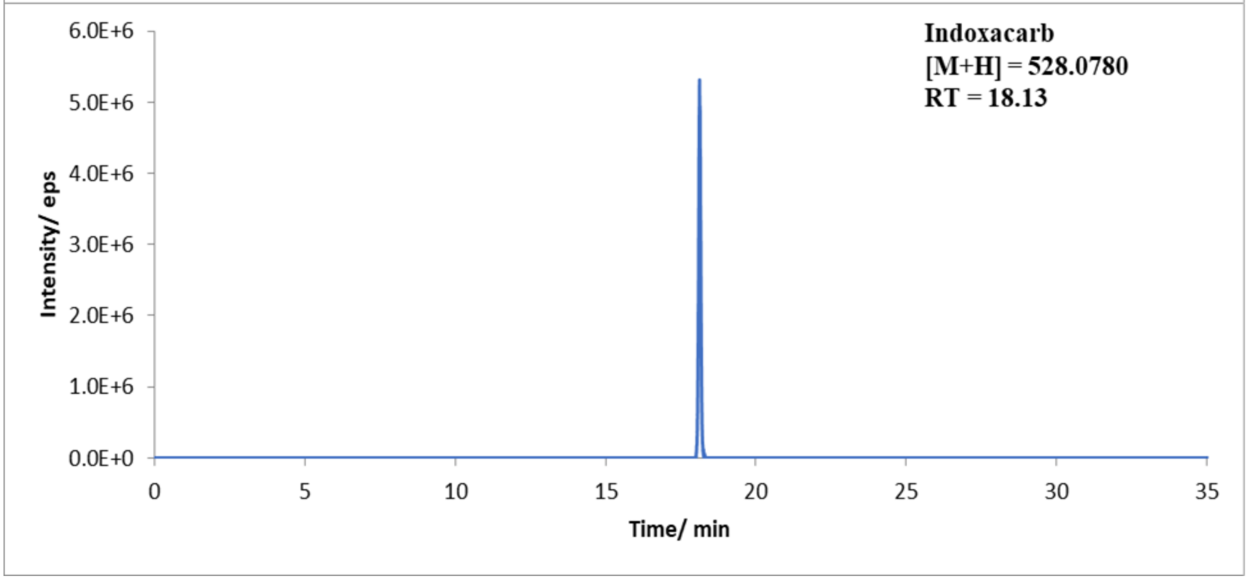

Indoxacarb
[M+H] = 528.0780
RT = 18.13
Intensity/ eps
6.0E+6
5.0E+6
4.0E+6
3.0E+6
2.0E+6
1.0E+6
0.0E+0
0
5
10
15
20
25
30
35
Time/ min

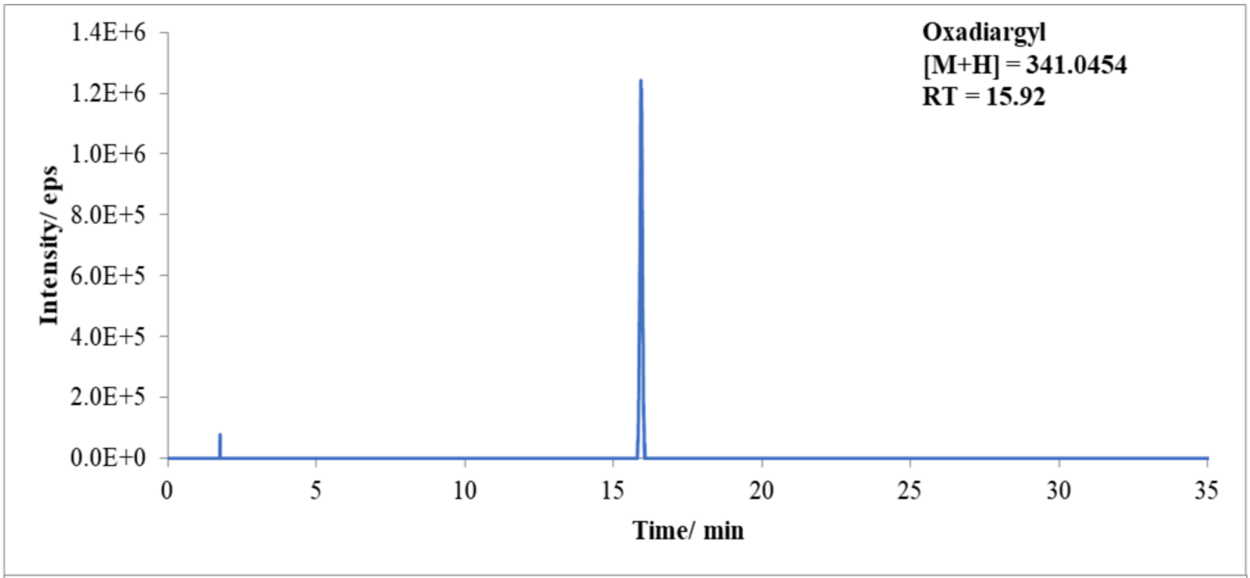

Oxadiargyl
[M+H] = 341.0454
RT = 15.92
Intensity/ eps
1.4E+6
1.2E+6
1.0E+6
8.0E+5
6.0E+5
4.0E+5
2.0E+5
0.0E+0
0
5
10
15
20
25
30
35
Time/ min

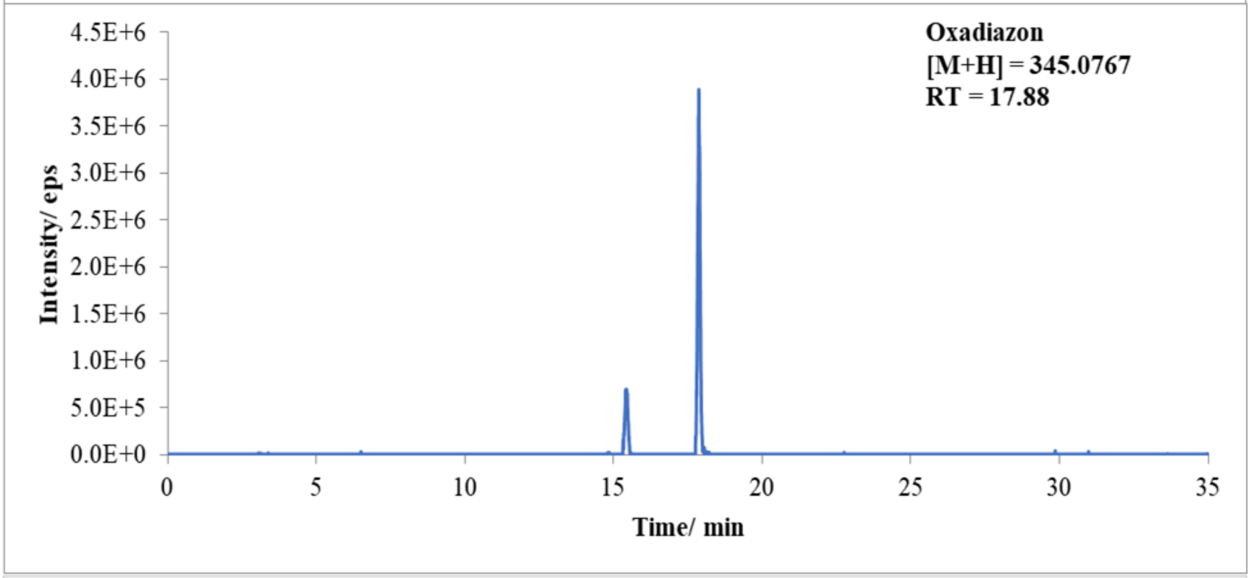

Oxadiazon
[M+H] = 345.0767
RT = 17.88
Intensity/ eps
4.5E+6
4.0E+6
3.5E+6
3.0E+6
2.5E+6
2.0E+6
1.5E+6
1.0E+6
5.0E+5
0.0E+0
0
5
10
15
20
25
30
35
Time/ min

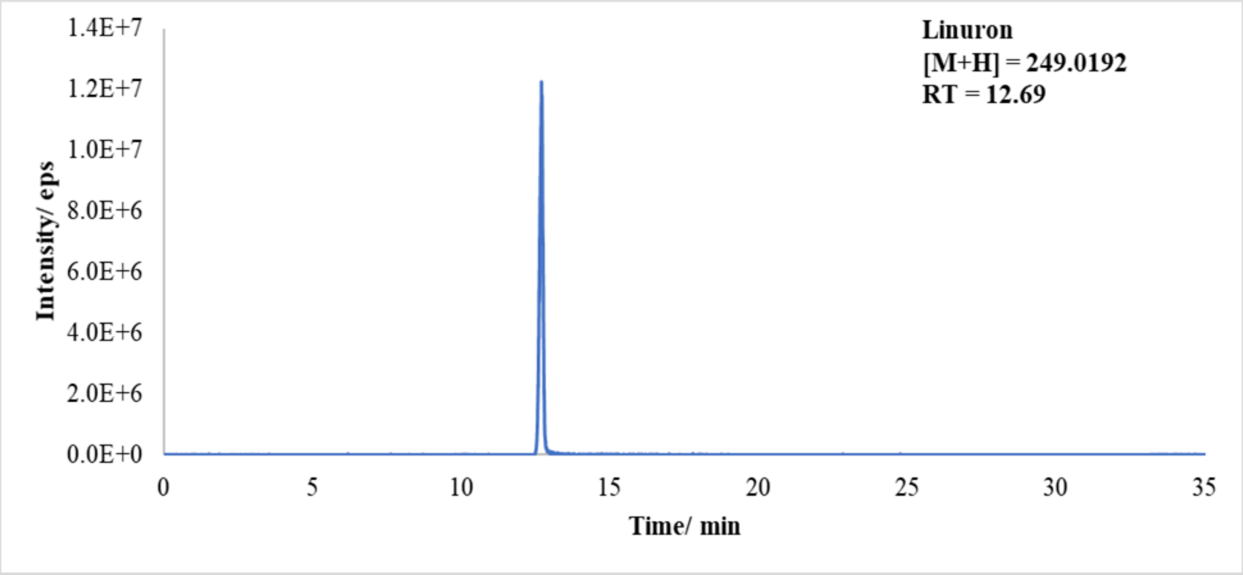

Linuron
[M+H] = 249.0192
RT = 12.69
Intensity/ eps
1.4E+7
1.2E+7
1.0E+7
8.0E+6
6.0E+6
4.0E+6
2.0E+6
0.0E+0
0
5
10
15
20
25
30
35
Time/ min

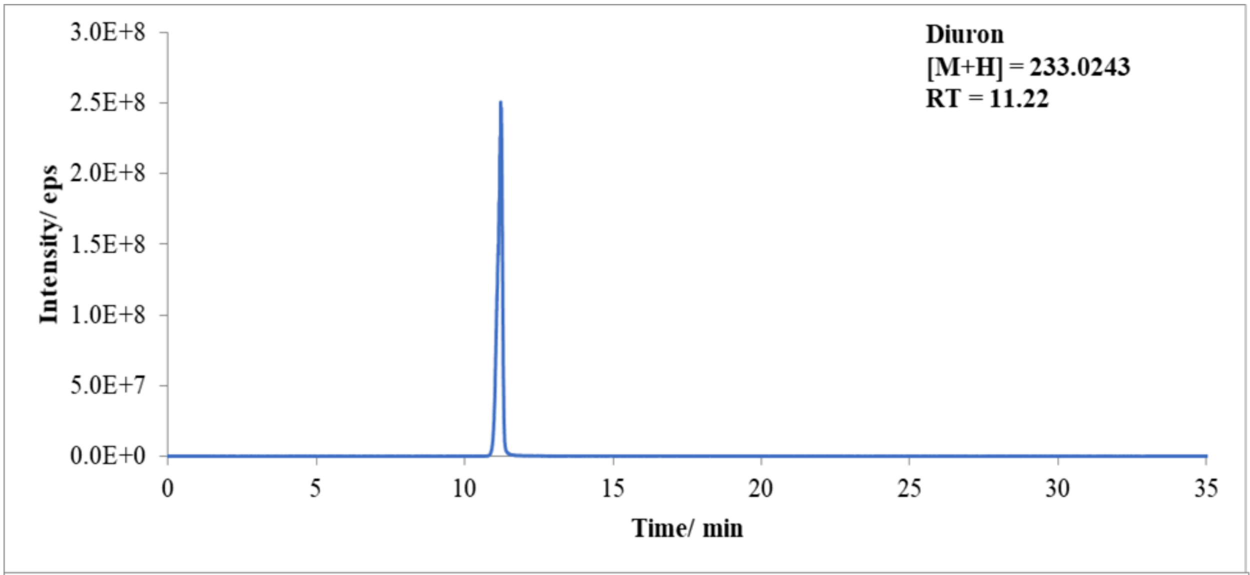
Diuron
[M+H] = 233.0243
RT = 11.22
Intensity/ eps
3.0E+8
2.5E+8
2.0E+8
1.5E+8
1.0E+8
5.0E+7
0.0E+0
0
5
10
15
20
25
30
35
Time/ min

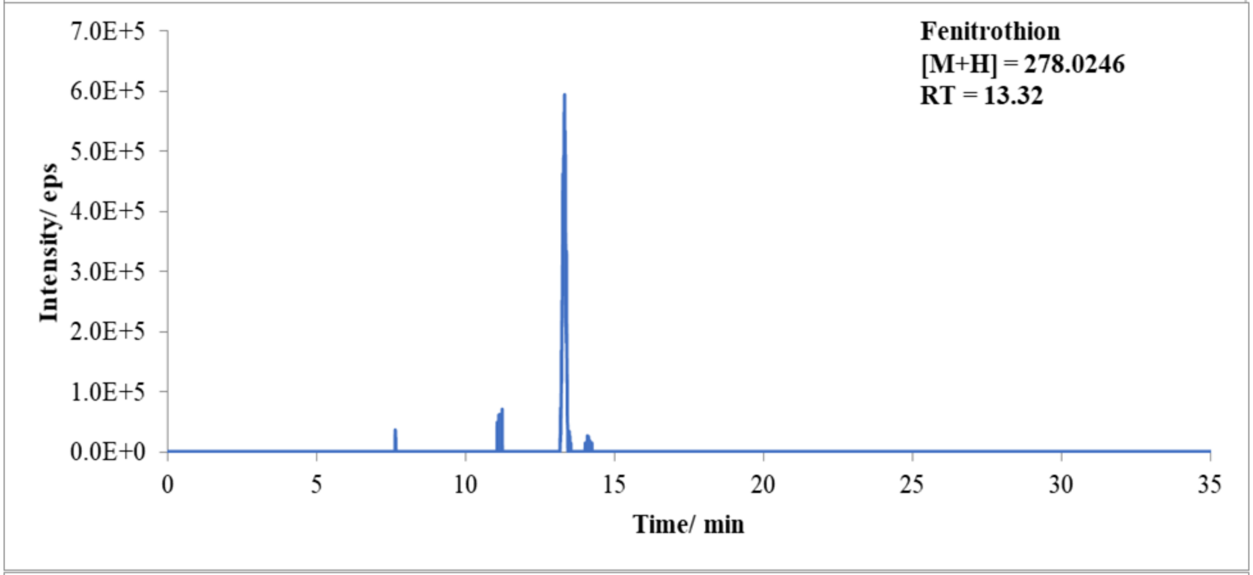
Fenitrothion
[M+H] = 278.0246
RT = 13.32
Intensity/ eps
7.0E+5
6.0E+5
5.0E+5
4.0E+5
3.0E+5
2.0E+5
1.0E+5
0.0E+0
0
5
10
15
20
25
30
35
Time/ min

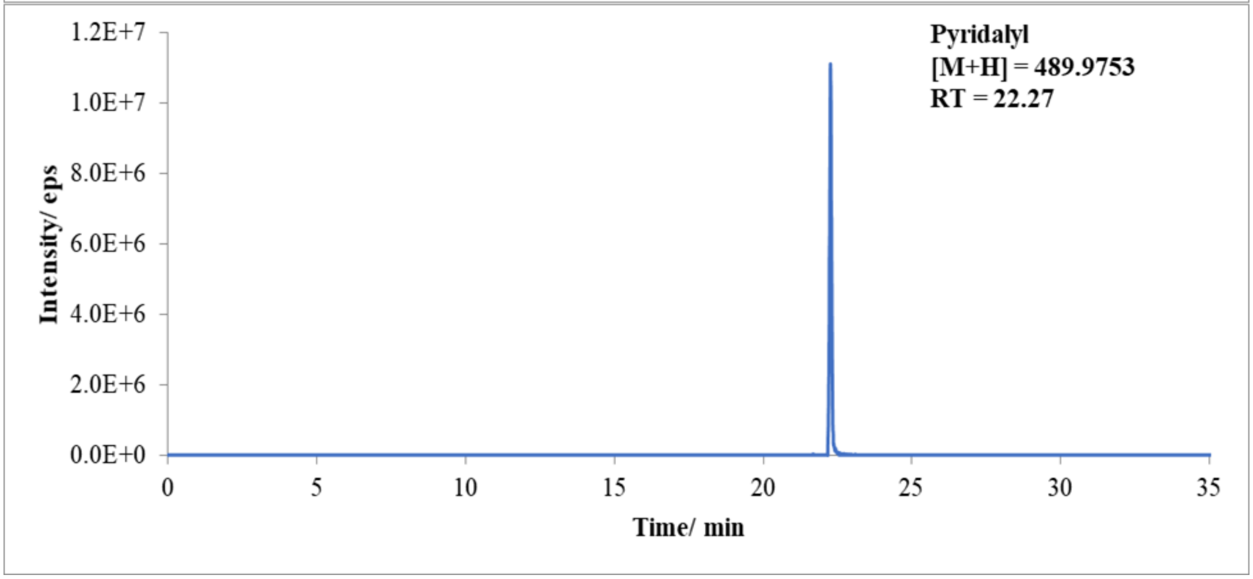
Pyridalyl
[M+H] = 489.9753
RT = 22.27
Intensity/ eps
1.2E+7
1.0E+7
8.0E+6
6.0E+6
4.0E+6
2.0E+6
0.0E+0
0
5
10
15
20
25
30
35
Time/ min

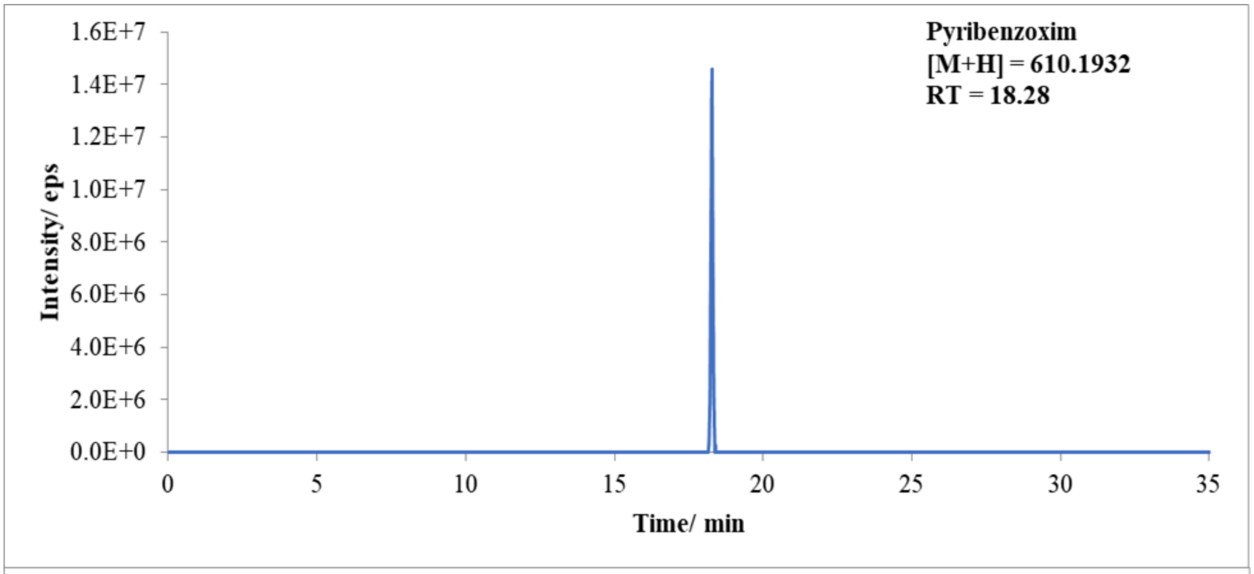
Pyribenzoxim
[M+H] = 610.1932
RT = 18.28
Intensity/ eps
1.6E+7
1.4E+7
1.2E+7
1.0E+7
8.0E+6
6.0E+6
4.0E+6
2.0E+6
0.0E+0
0
5
10
15
20
25
30
35
Time/ min

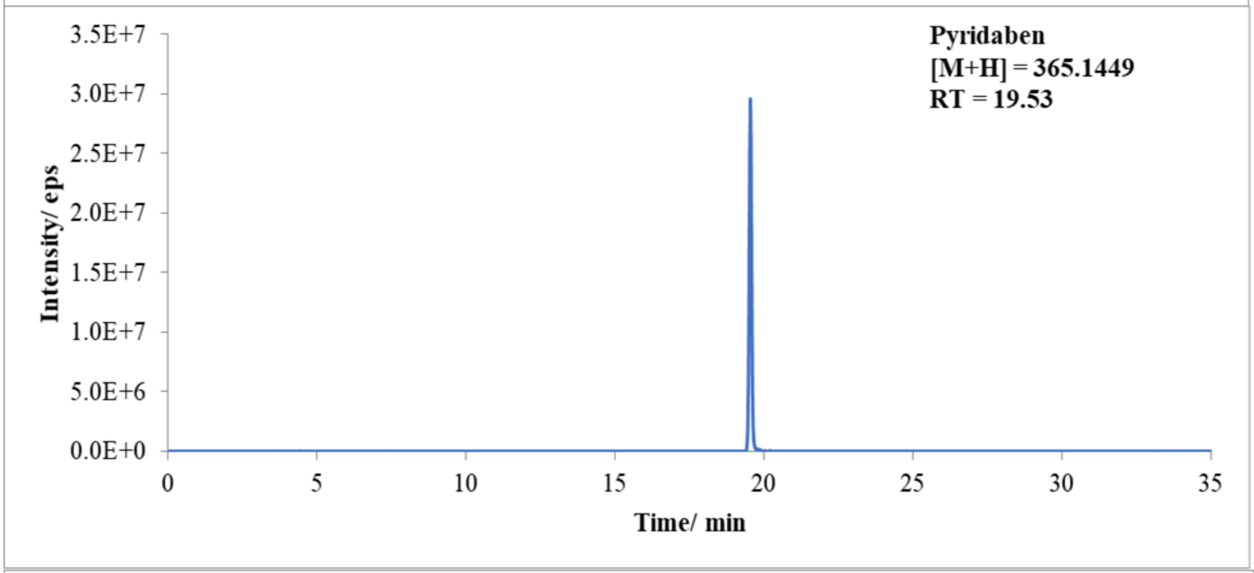
Pyridaben
[M+H] = 365.1449
RT = 19.53
Intensity/ eps
3.5E+7
3.0E+7
2.5E+7
2.0E+7
1.5E+7
1.0E+7
5.0E+6
0.0E+0
0
5
10
15
20
25
30
35
Time/ min

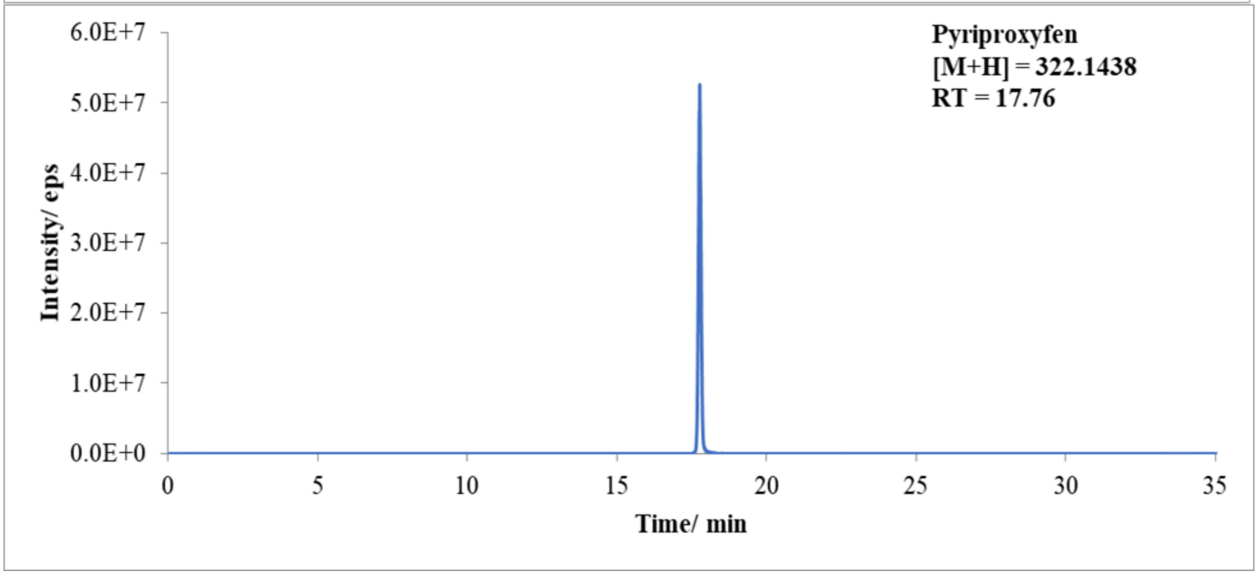
Pyriproxyfen
[M+H] = 322.1438
RT = 17.76
Intensity/ eps
6.0E+7
5.0E+7
4.0E+7
3.0E+7
2.0E+7
1.0E+7
0.0E+0
0
5
10
15
20
25
30
35
Time/ min

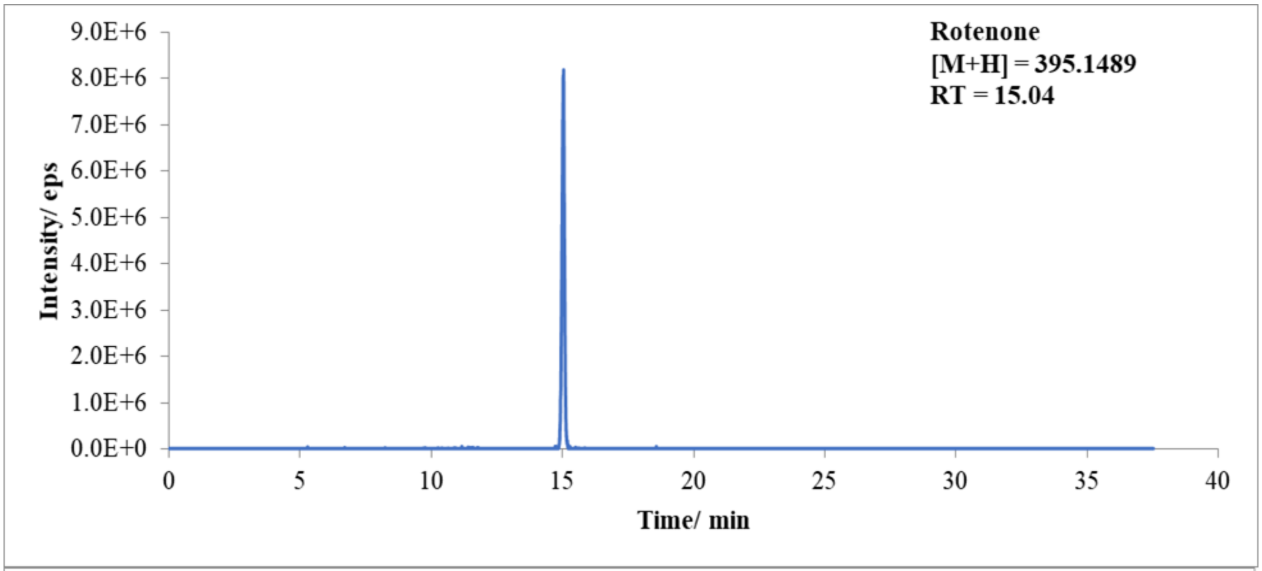
Rotenone
[M+H] = 395.1489
RT = 15.04
Intensity/ eps
9.0E+6
8.0E+6
7.0E+6
6.0E+6
5.0E+6
4.0E+6
3.0E+6
2.0E+6
1.0E+6
0.0E+0
0
5
10
15
20
25
30
35
40
Time/ min

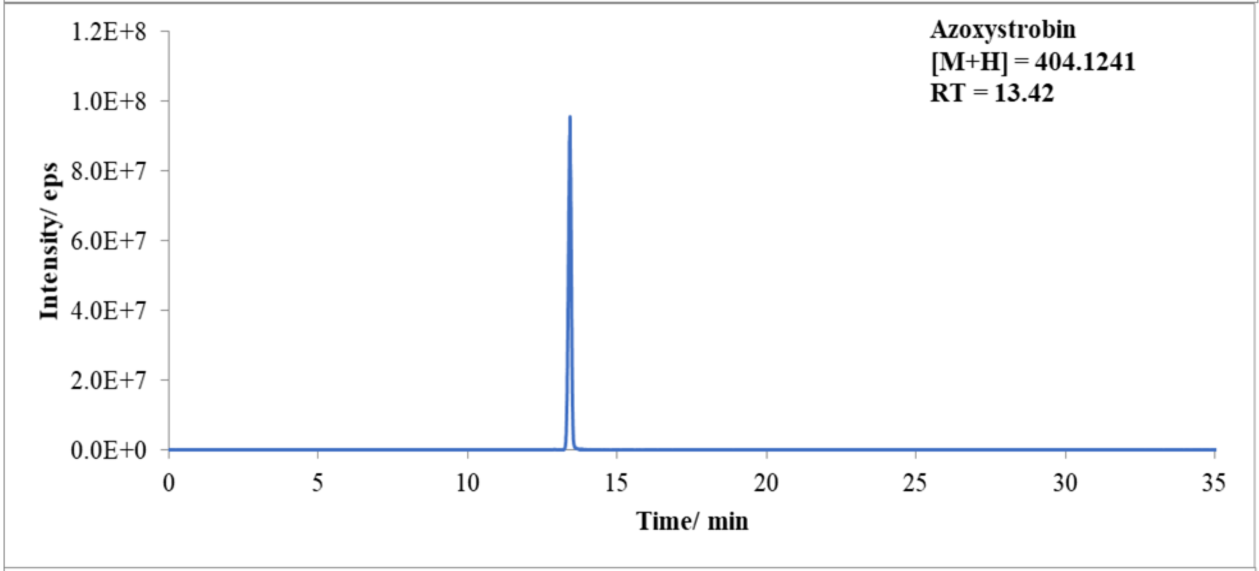
Azoxystrobin
[M+H] = 404.1241
RT = 13.42
Intensity/ eps
1.2E+8
1.0E+8
8.0E+7
6.0E+7
4.0E+7
2.0E+7
0.0E+0
0
5
10
15
20
25
30
35
Time/ min

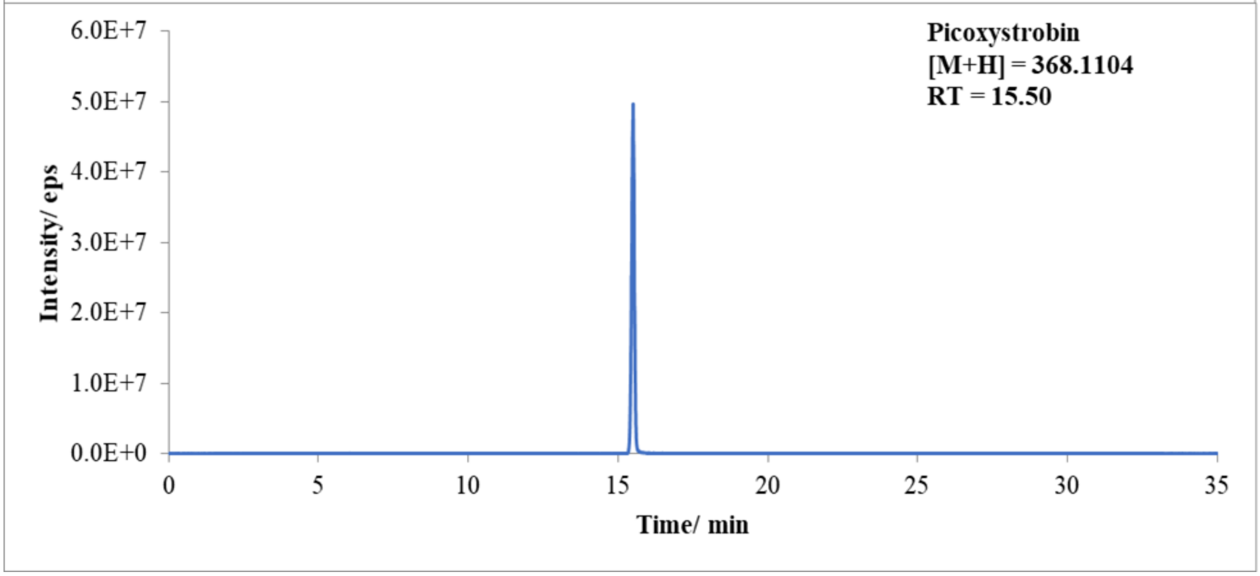
Picoxystrobin
[M+H] = 368.1104
RT = 15.50
Intensity/ eps
6.0E+7
5.0E+7
4.0E+7
3.0E+7
2.0E+7
1.0E+7
0.0E+0
0
5
10
15
20
25
30
35
Time/ min

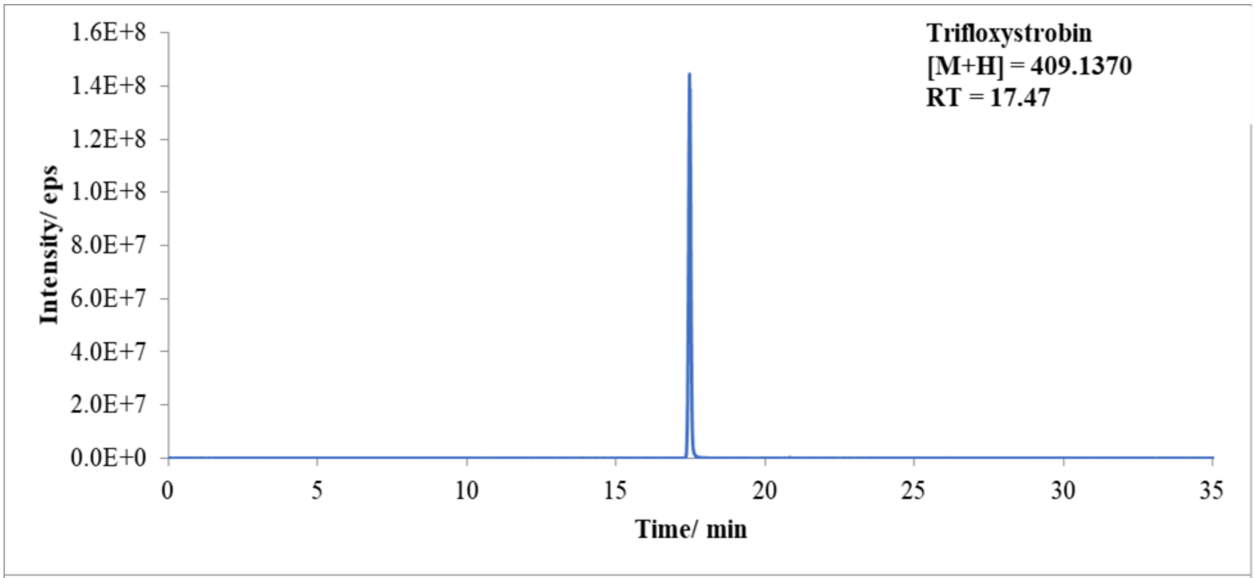
Trifloxystrobin
[M+H] = 409.1370
RT = 17.47
Intensity/ eps
Time/ min
1.6E+8
1.4E+8
1.2E+8
1.0E+8
8.0E+7
6.0E+7
4.0E+7
2.0E+7
0.0E+0
0
5
10
15
20
25
30
35

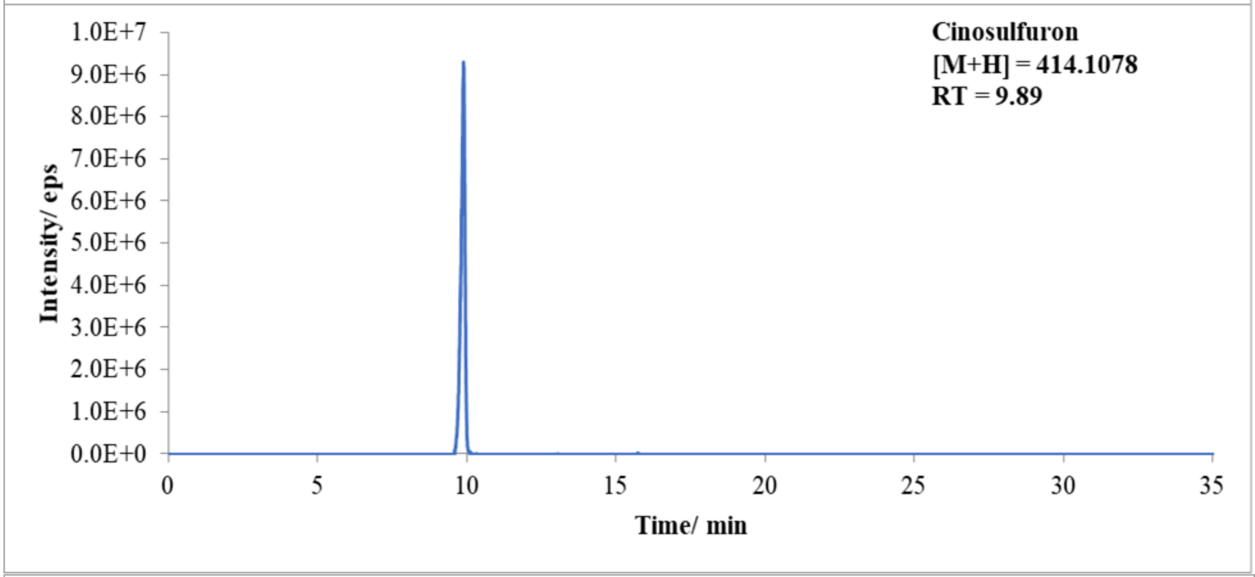
Cinosulfuron
[M+H] = 414.1078
RT = 9.89
Intensity/ eps
Time/ min
1.0E+7
9.0E+6
8.0E+6
7.0E+6
6.0E+6
5.0E+6
4.0E+6
3.0E+6
2.0E+6
1.0E+6
0.0E+0
0
5
10
15
20
25
30
35

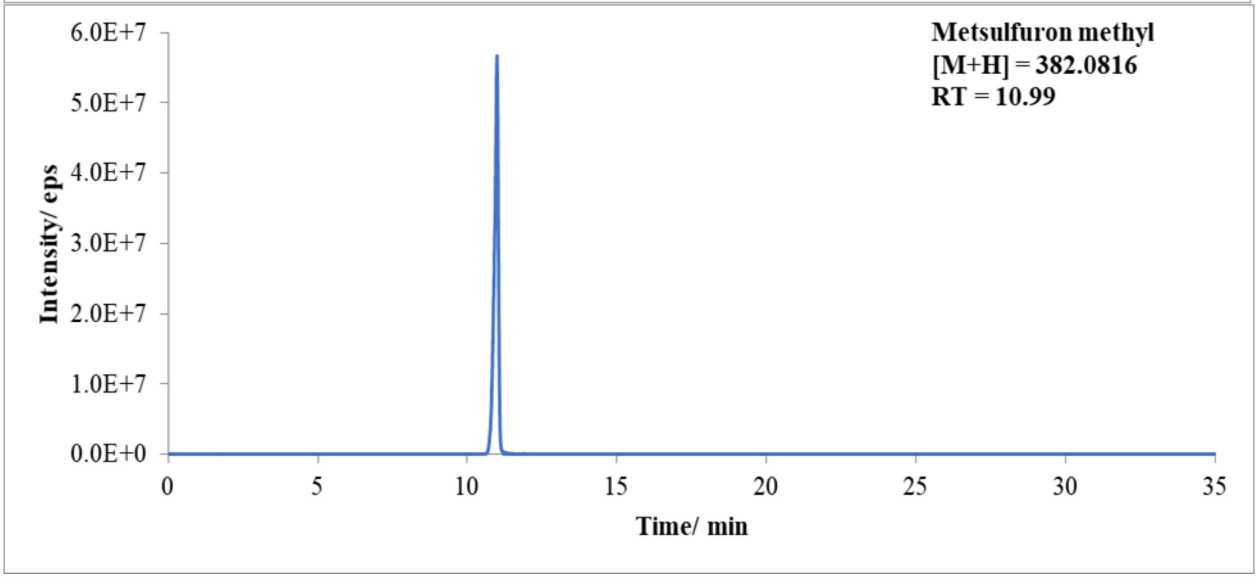
Metsulfuron methyl
[M+H] = 382.0816
RT = 10.99
Intensity/ eps
Time/ min
6.0E+7
5.0E+7
4.0E+7
3.0E+7
2.0E+7
1.0E+7
0.0E+0
0
5
10
15
20
25
30
35

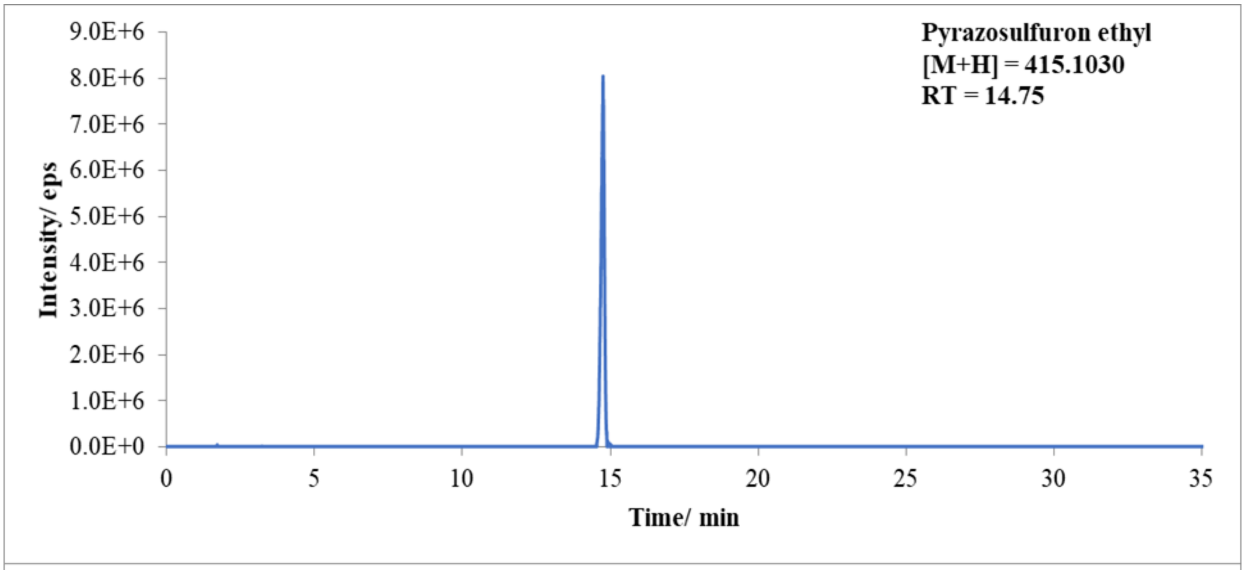
Pyrazosulfuron ethyl
[M+H] = 415.1030
RT = 14.75
Intensity/ eps
Time/ min

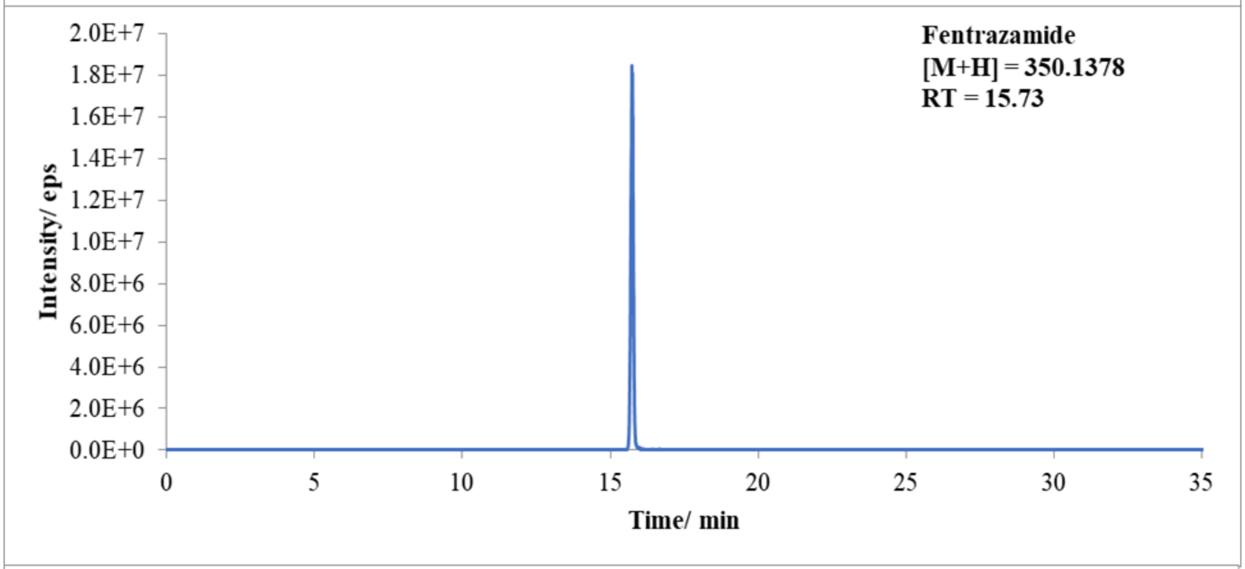
Fentrazamide
[M+H] = 350.1378
RT = 15.73
Intensity/ eps
Time/ min

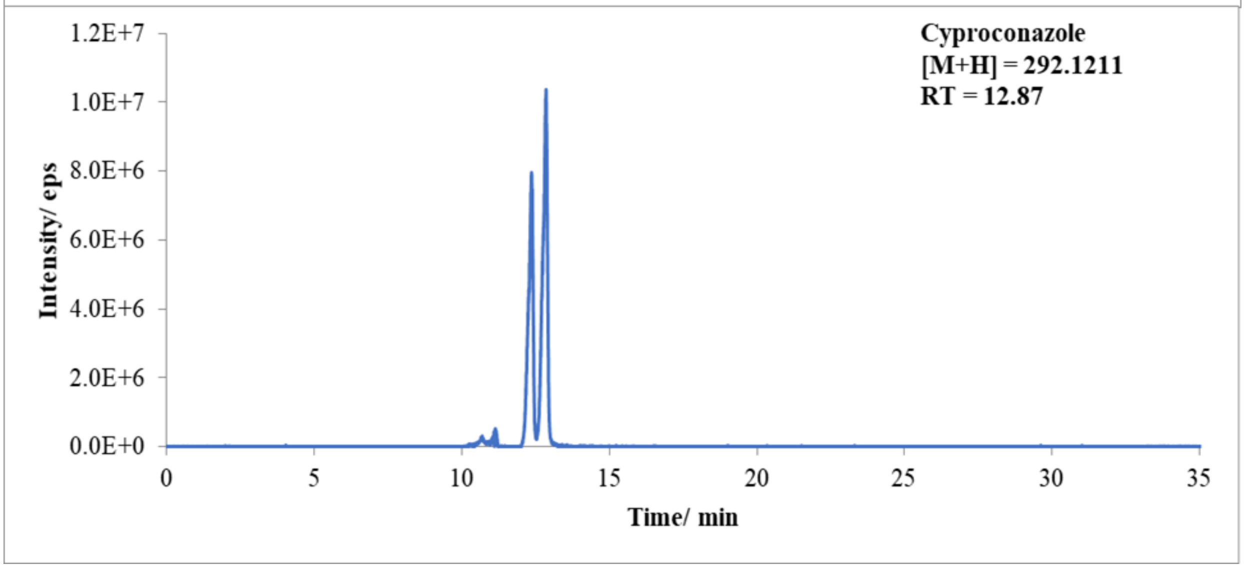
Cyproconazole
[M+H] = 292.1211
RT = 12.87
Intensity/ eps
Time/ min

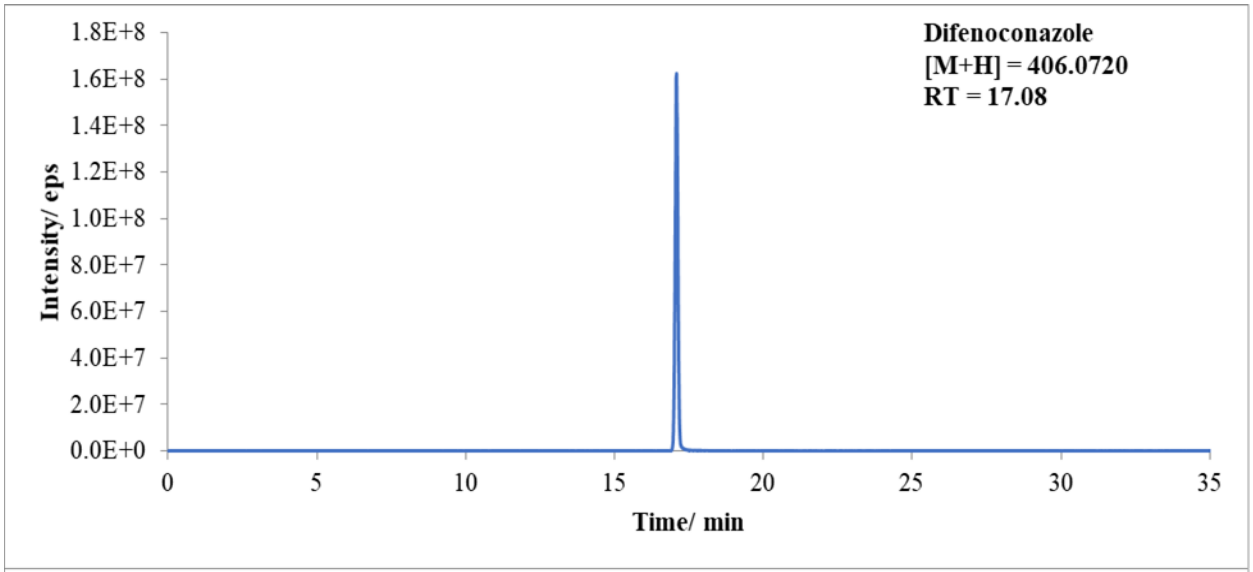
Difenoconazole
[M+H] = 406.0720
RT = 17.08
Intensity/ eps
1.8E+8
1.6E+8
1.4E+8
1.2E+8
1.0E+8
8.0E+7
6.0E+7
4.0E+7
2.0E+7
0.0E+0
0
5
10
15
20
25
30
35
Time/ min

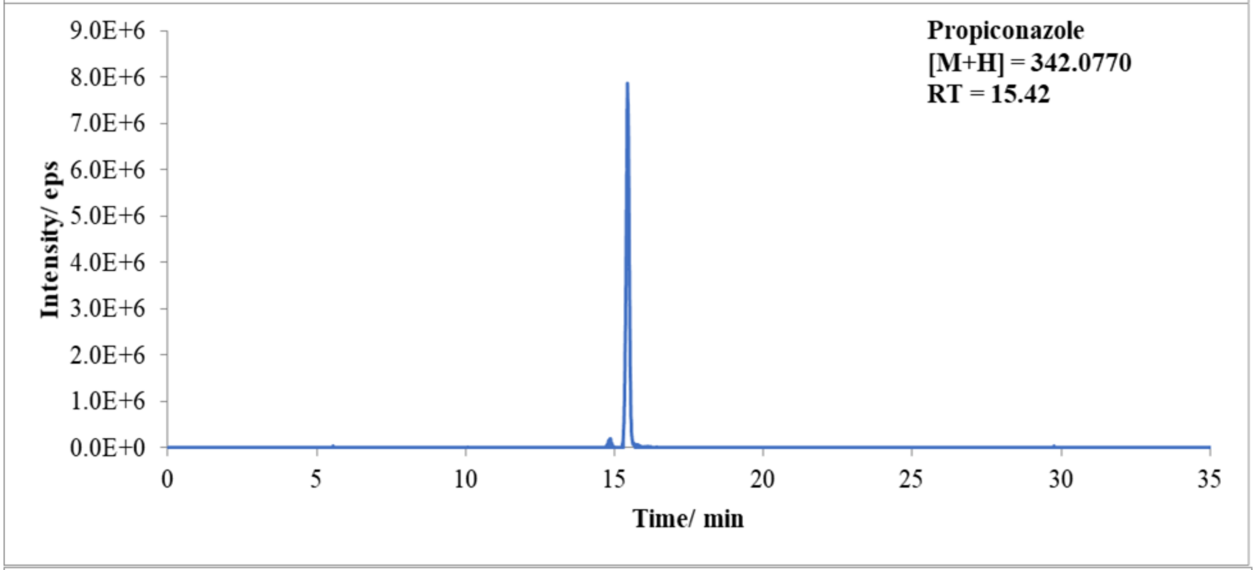
Propiconazole
[M+H] = 342.0770
RT = 15.42
Intensity/ eps
9.0E+6
8.0E+6
7.0E+6
6.0E+6
5.0E+6
4.0E+6
3.0E+6
2.0E+6
1.0E+6
0.0E+0
0
5
10
15
20
25
30
35
Time/ min

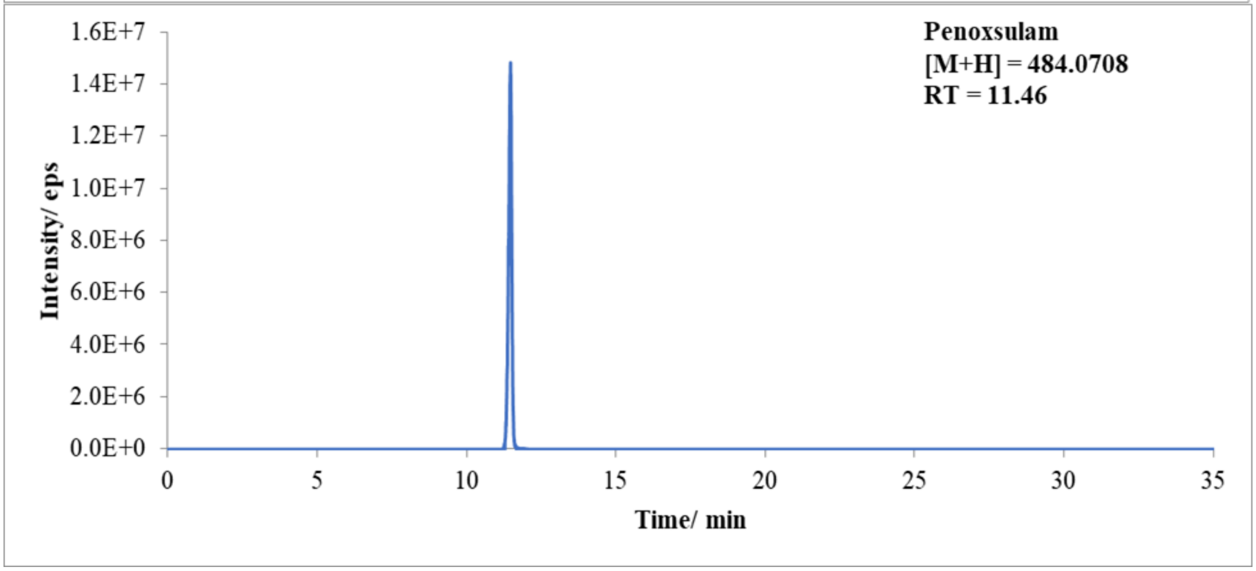
Penoxsulam
[M+H] = 484.0708
RT = 11.46
Intensity/ eps
1.6E+7
1.4E+7
1.2E+7
1.0E+7
8.0E+6
6.0E+6
4.0E+6
2.0E+6
0.0E+0
0
5
10
15
20
25
30
35
Time/ min

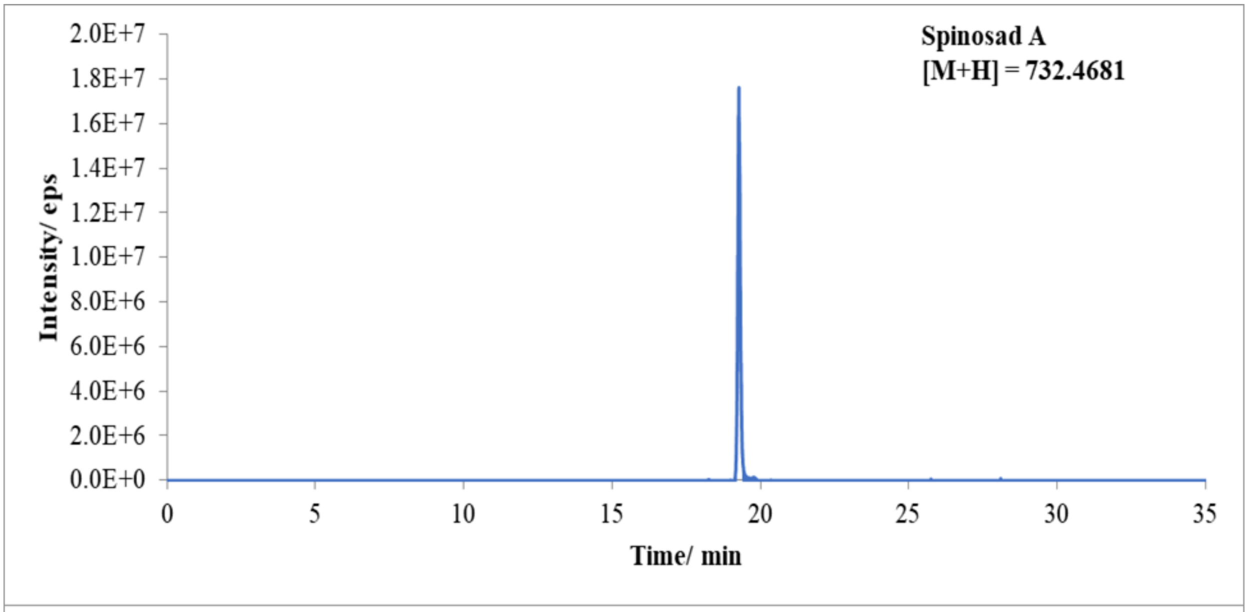
Spinosad A
[M+H] = 732.4681
Intensity/ eps
Time/ min
2.0E+7
1.8E+7
1.6E+7
1.4E+7
1.2E+7
1.0E+7
8.0E+6
6.0E+6
4.0E+6
2.0E+6
0.0E+0
0
5
10
15
20
25
30
35

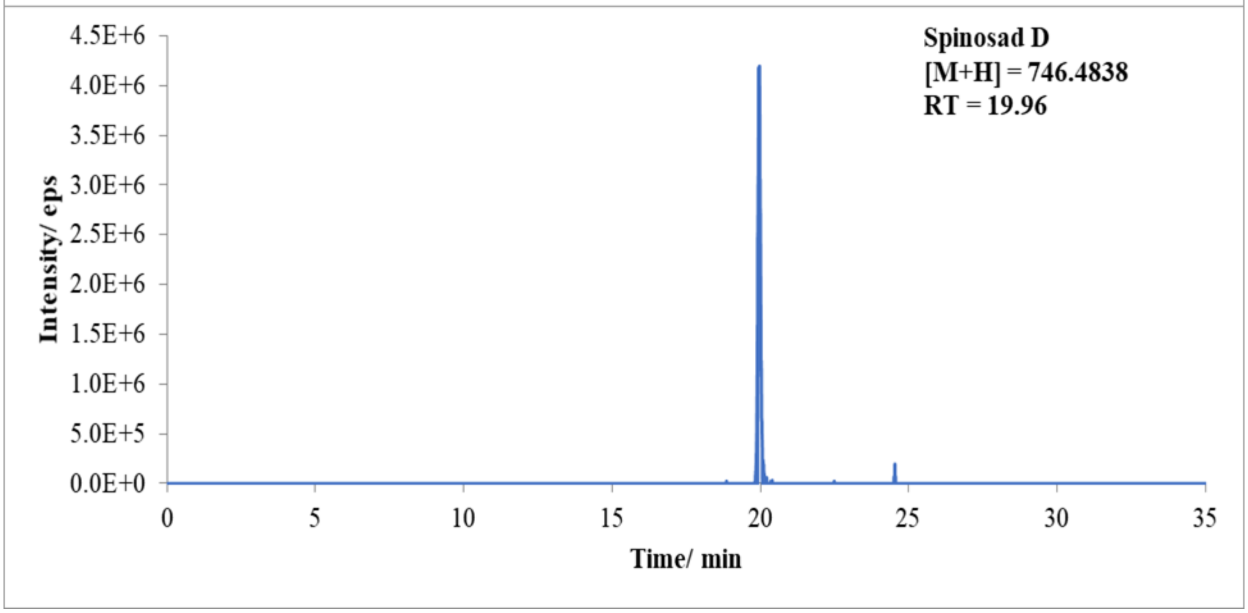
Spinosad D
[M+H] = 746.4838
RT = 19.96
Intensity/ eps
Time/ min
4.5E+6
4.0E+6
3.5E+6
3.0E+6
2.5E+6
2.0E+6
1.5E+6
1.0E+6
5.0E+5
0.0E+0
0
5
10
15
20
25
30
35

Fig. S4. The total ion chromatogram and extracted ion chromatogram of the target analytes in the matrix match solution (negative mode)

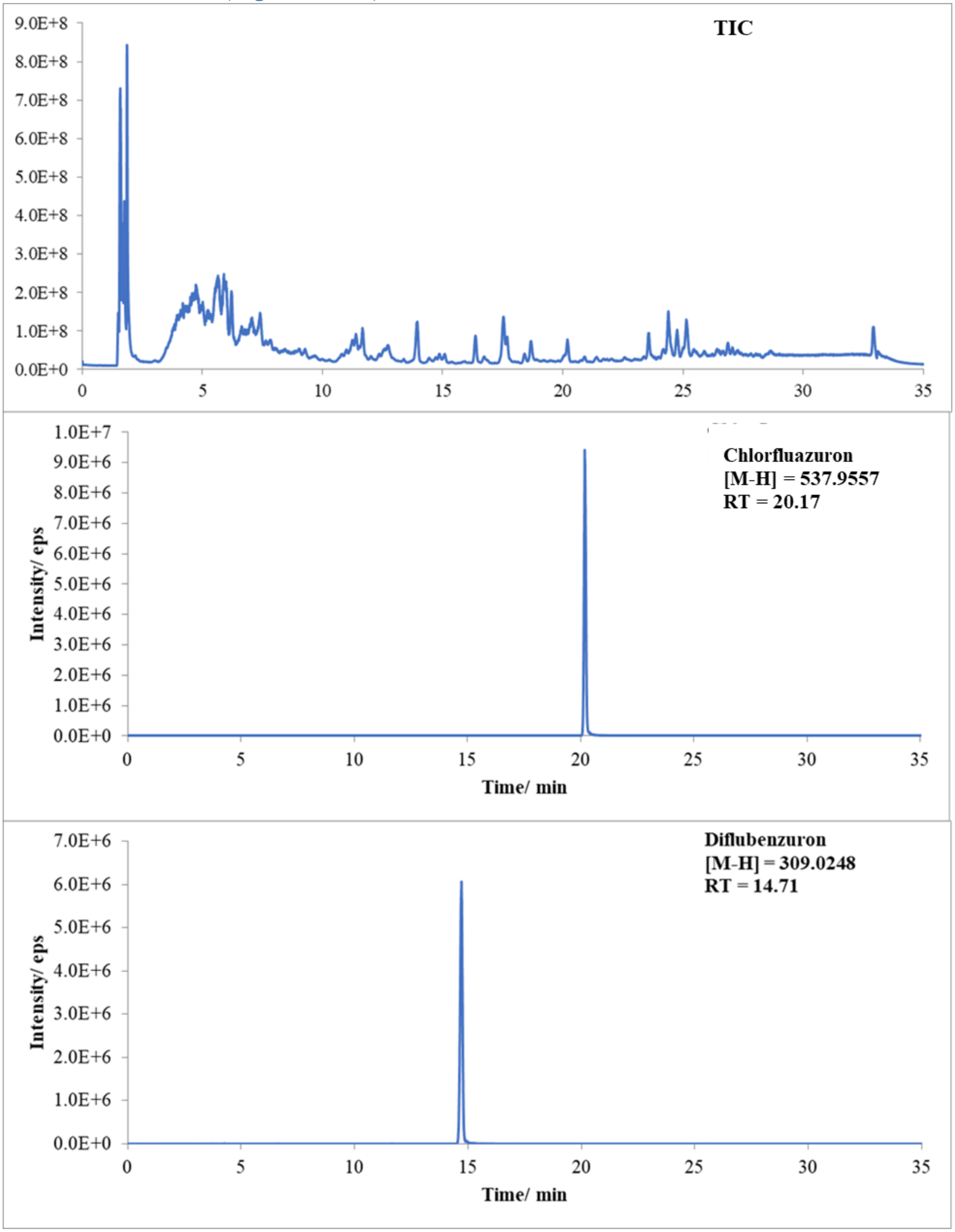

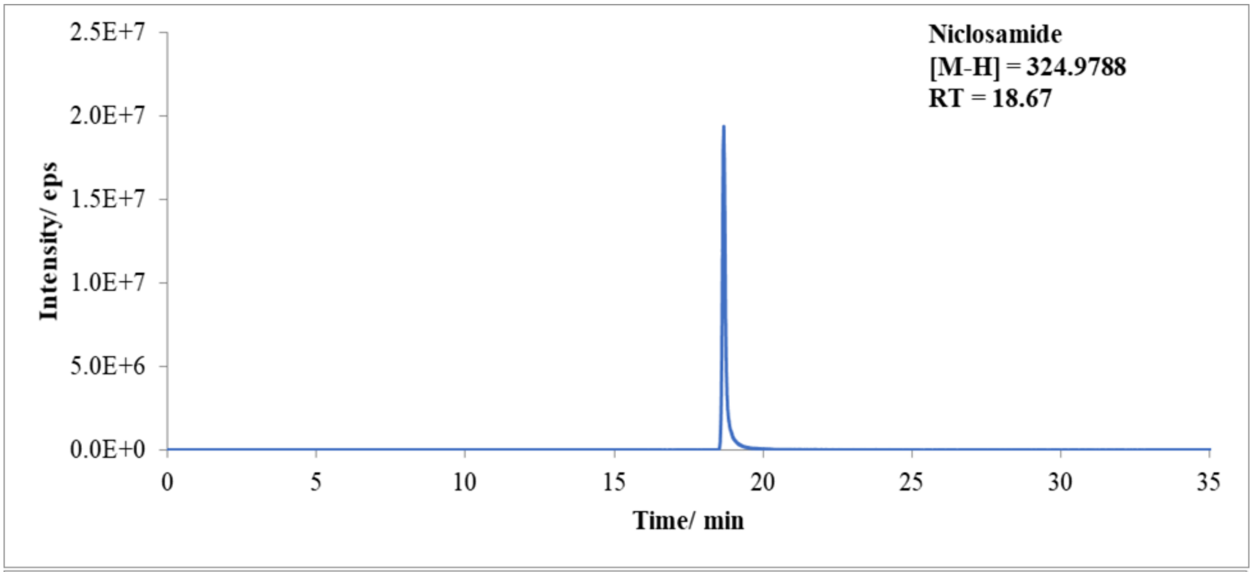
Niclosamide
[M-H] = 324.9788
RT = 18.67
Intensity/ eps
2.5E+7
2.0E+7
1.5E+7
1.0E+7
5.0E+6
0.0E+0
0
5
10
15
20
25
30
35
Time/ min

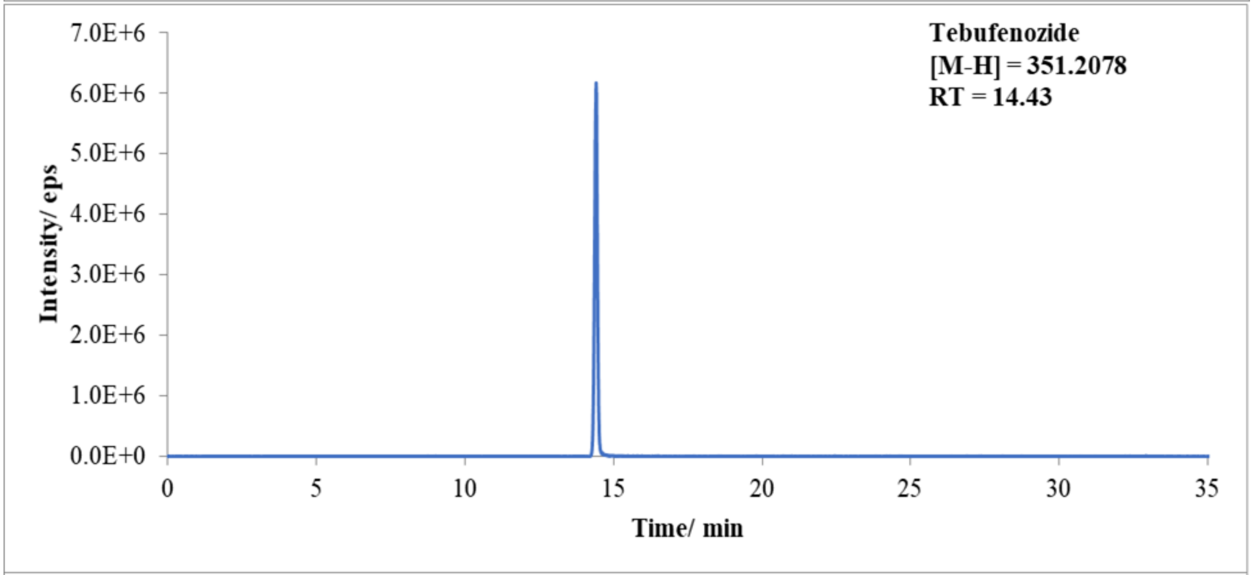
Tebufenozide
[M-H] = 351.2078
RT = 14.43
Intensity/ eps
7.0E+6
6.0E+6
5.0E+6
4.0E+6
3.0E+6
2.0E+6
1.0E+6
0.0E+0
0
5
10
15
20
25
30
35
Time/ min

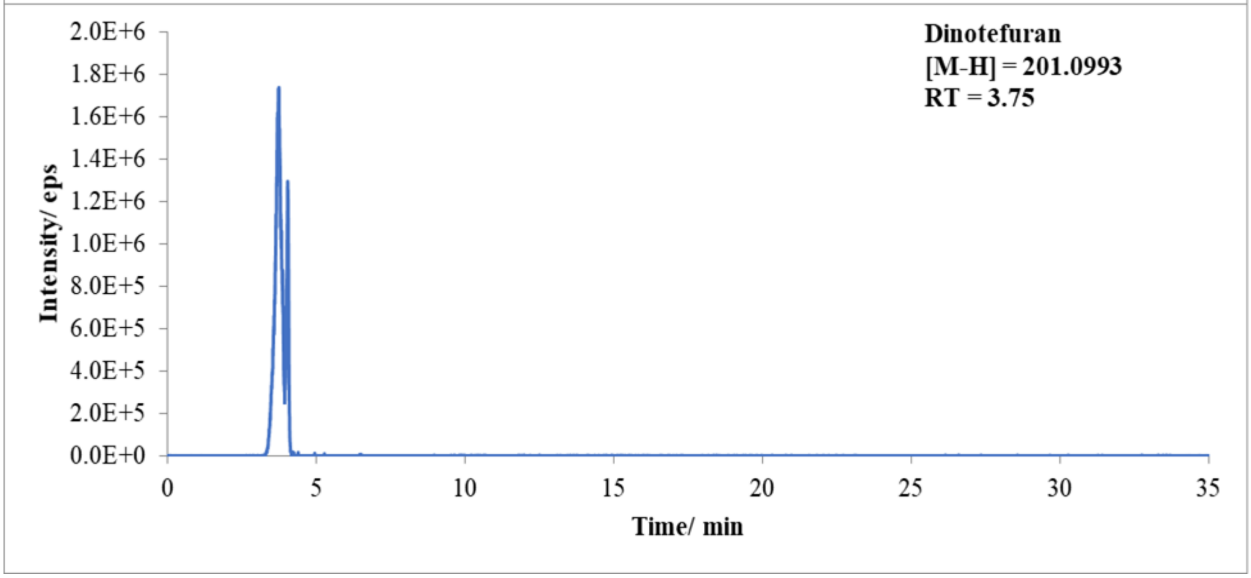
Dinotefuran
[M-H] = 201.0993
RT = 3.75
Intensity/ eps
2.0E+6
1.8E+6
1.6E+6
1.4E+6
1.2E+6
1.0E+6
8.0E+5
6.0E+5
4.0E+5
2.0E+5
0.0E+0
0
5
10
15
20
25
30
35
Time/ min

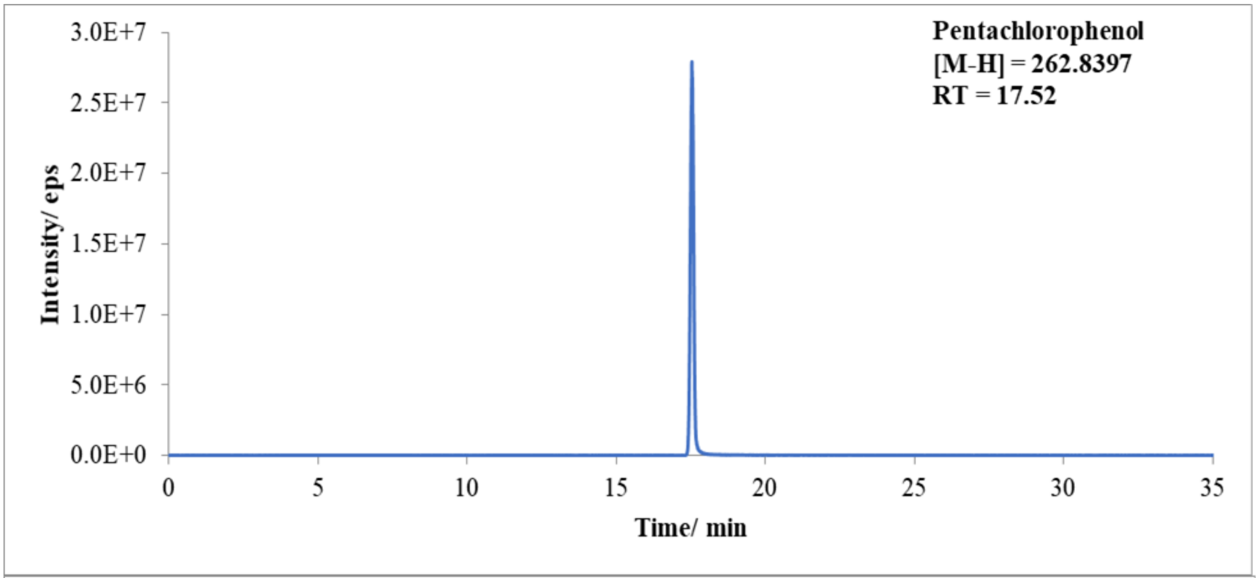
Pentachlorophenol
[M-H] = 262.8397
RT = 17.52
Intensity/ eps
Time/ min

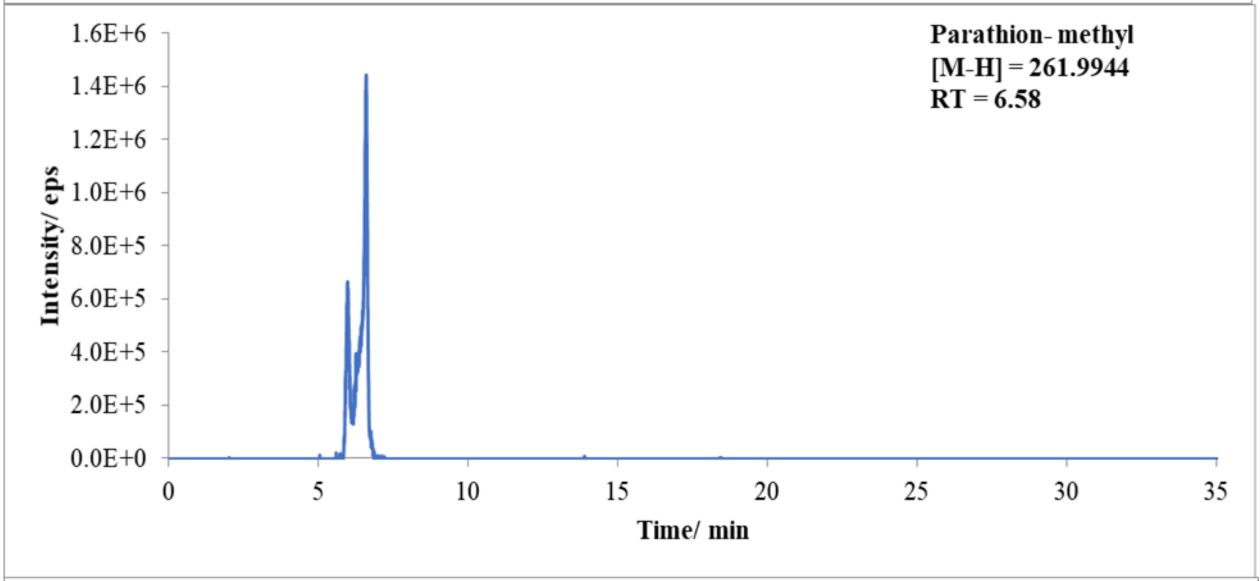
Parathion- methyl
[M-H] = 261.9944
RT = 6.58
Intensity/ eps
Time/ min

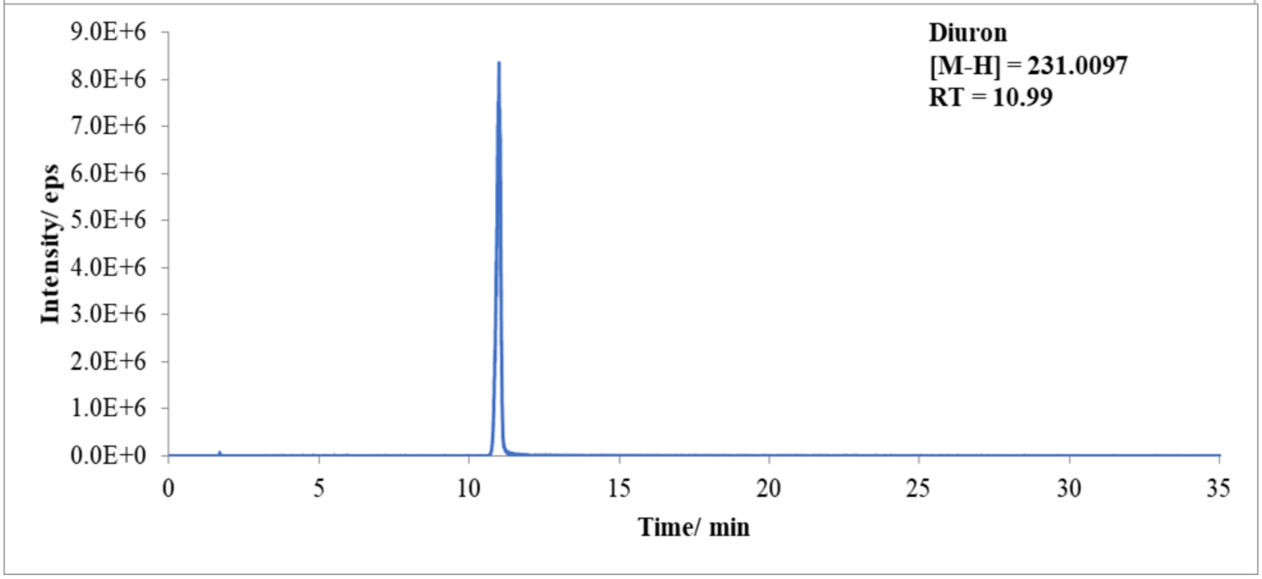
Diuron
[M-H] = 231.0097
RT = 10.99
Intensity/ eps
Time/ min

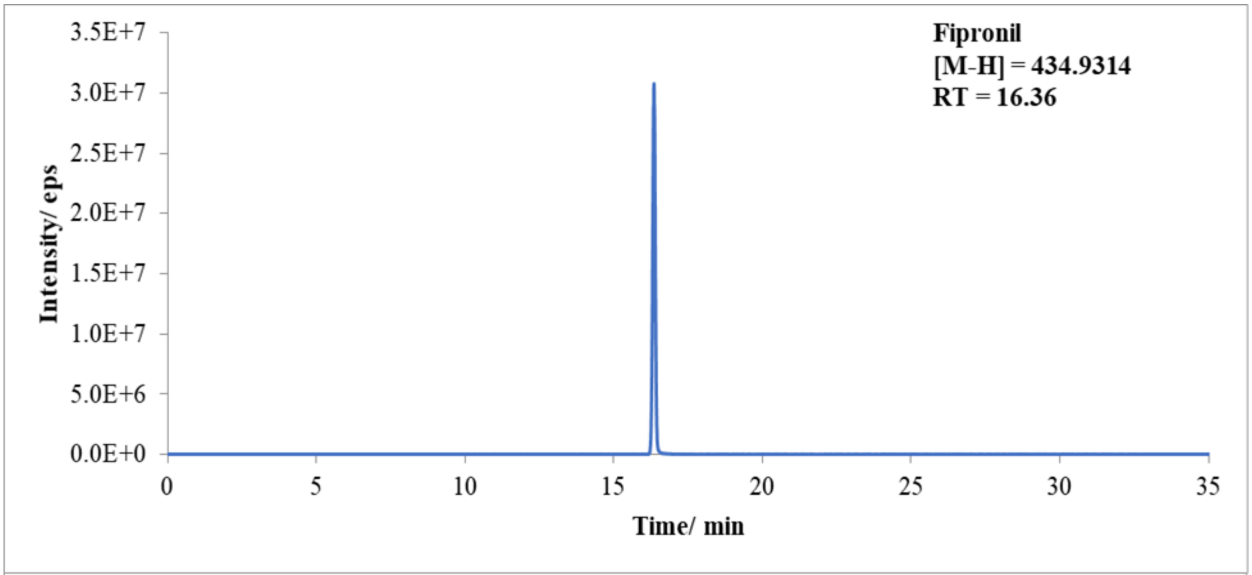
Fipronil
[M-H] = 434.9314
RT = 16.36
Intensity/ eps
3.5E+7
3.0E+7
2.5E+7
2.0E+7
1.5E+7
1.0E+7
5.0E+6
0.0E+0
0
5
10
15
20
25
30
35
Time/ min

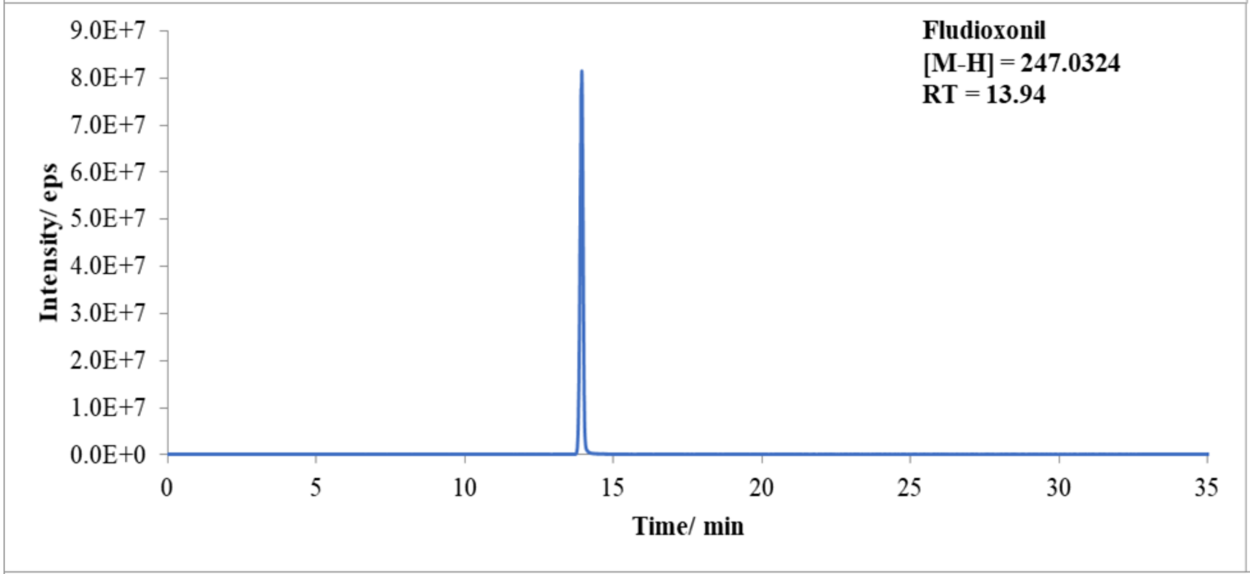
Fludioxonil
[M-H] = 247.0324
RT = 13.94
Intensity/ eps
9.0E+7
8.0E+7
7.0E+7
6.0E+7
5.0E+7
4.0E+7
3.0E+7
2.0E+7
1.0E+7
0.0E+0
0
5
10
15
20
25
30
35
Time/ min

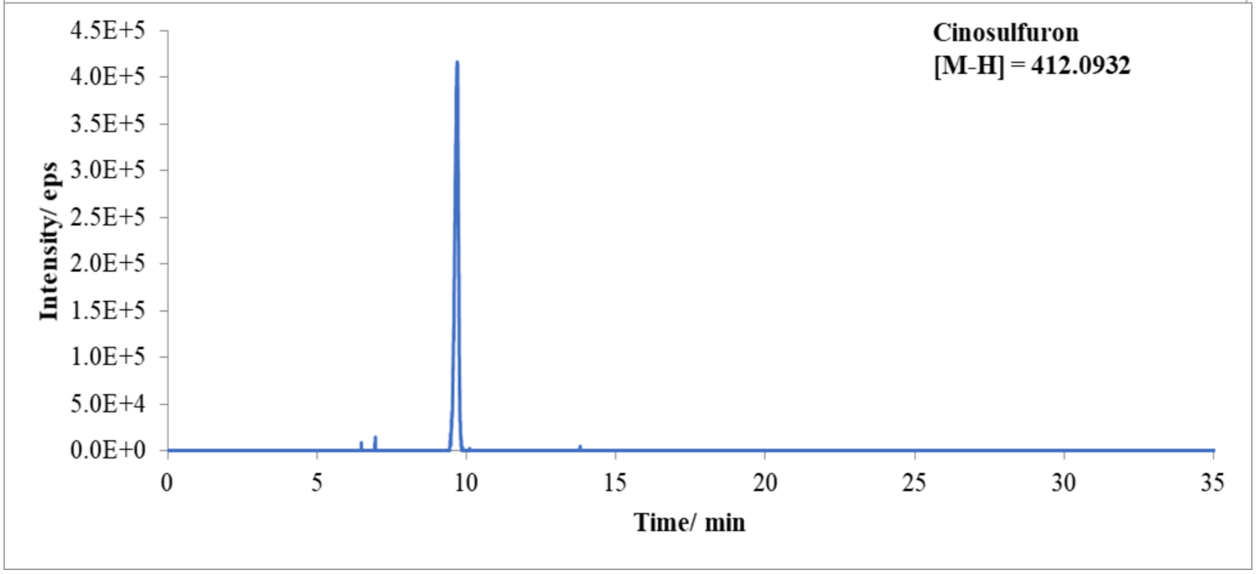
Cinosulfuron
[M-H] = 412.0932
Intensity/ eps
4.5E+5
4.0E+5
3.5E+5
3.0E+5
2.5E+5
2.0E+5
1.5E+5
1.0E+5
5.0E+4
0.0E+0
0
5
10
15
20
25
30
35
Time/ min

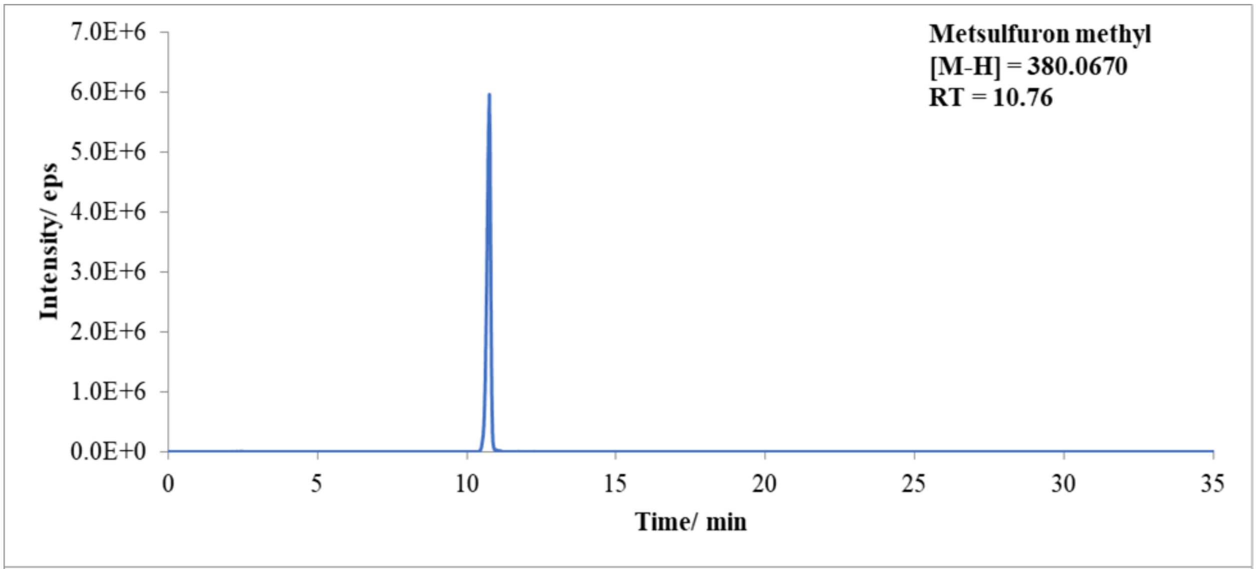
Metsulfuron methyl
[M-H] = 380.0670
RT = 10.76
Intensity/ eps
7.0E+6
6.0E+6
5.0E+6
4.0E+6
3.0E+6
2.0E+6
1.0E+6
0.0E+0
0
5
10
15
20
25
30
35
Time/ min

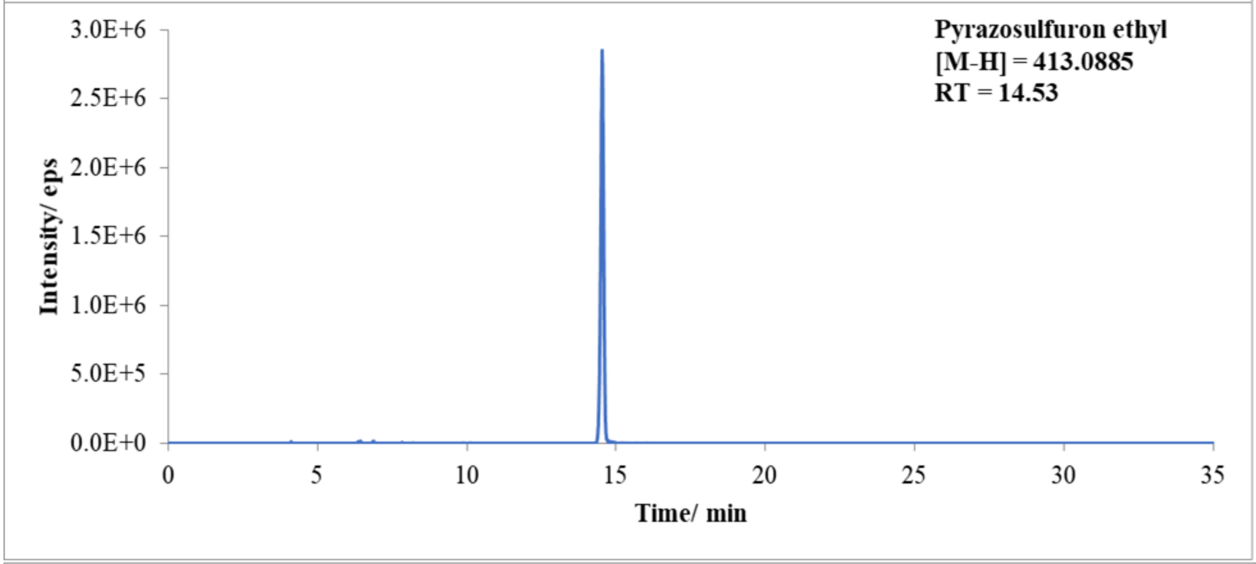
Pyrazosulfuron ethyl
[M-H] = 413.0885
RT = 14.53
Intensity/ eps
3.0E+6
2.5E+6
2.0E+6
1.5E+6
1.0E+6
5.0E+5
0.0E+0
0
5
10
15
20
25
30
35
Time/ min

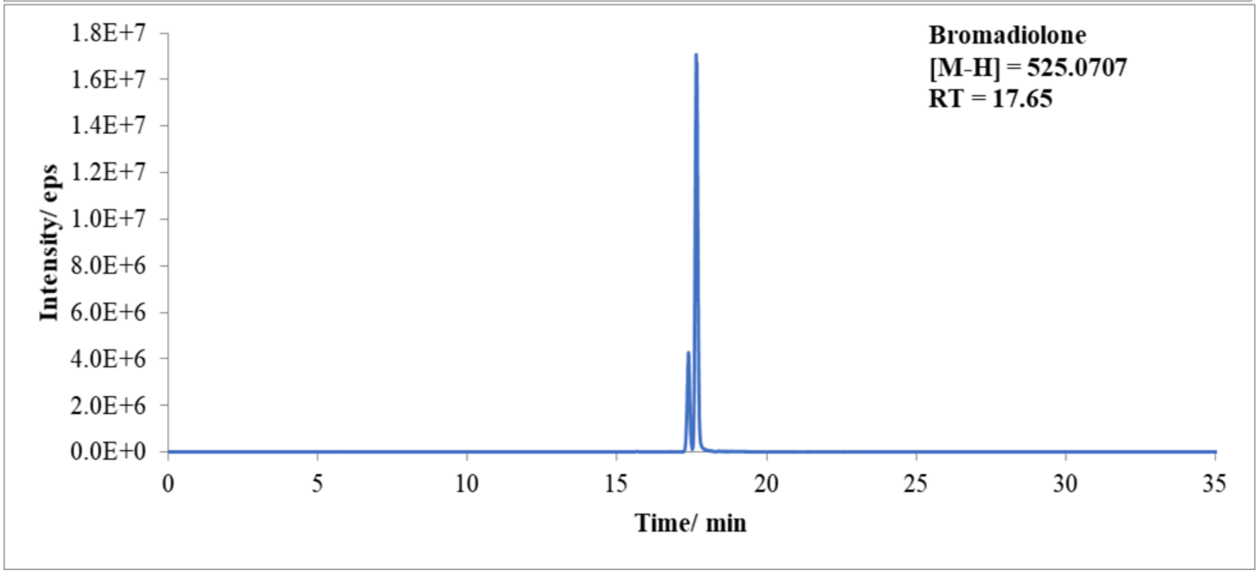
Bromadiolone
[M-H] = 525.0707
RT = 17.65
Intensity/ eps
1.8E+7
1.6E+7
1.4E+7
1.2E+7
1.0E+7
8.0E+6
6.0E+6
4.0E+6
2.0E+6
0.0E+0
0
5
10
15
20
25
30
35
Time/ min

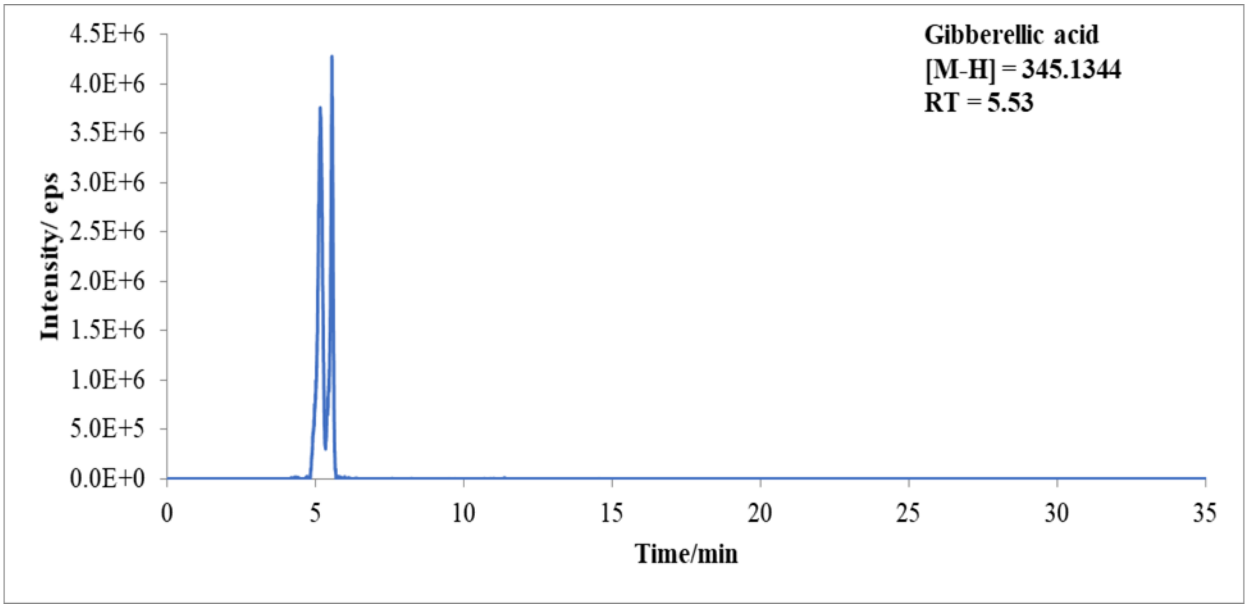
Gibberellic acid
[M-H] = 345.1344
RT = 5.53
Intensity/ eps
4.5E+6
4.0E+6
3.5E+6
3.0E+6
2.5E+6
2.0E+6
1.5E+6
1.0E+6
5.0E+5
0.0E+0
0
5
10
15
20
25
30
35
Time/min

Fig. S5: The total ion chromatogram and extract ion chromatogram of the target analytes in several real samples (positive mode)

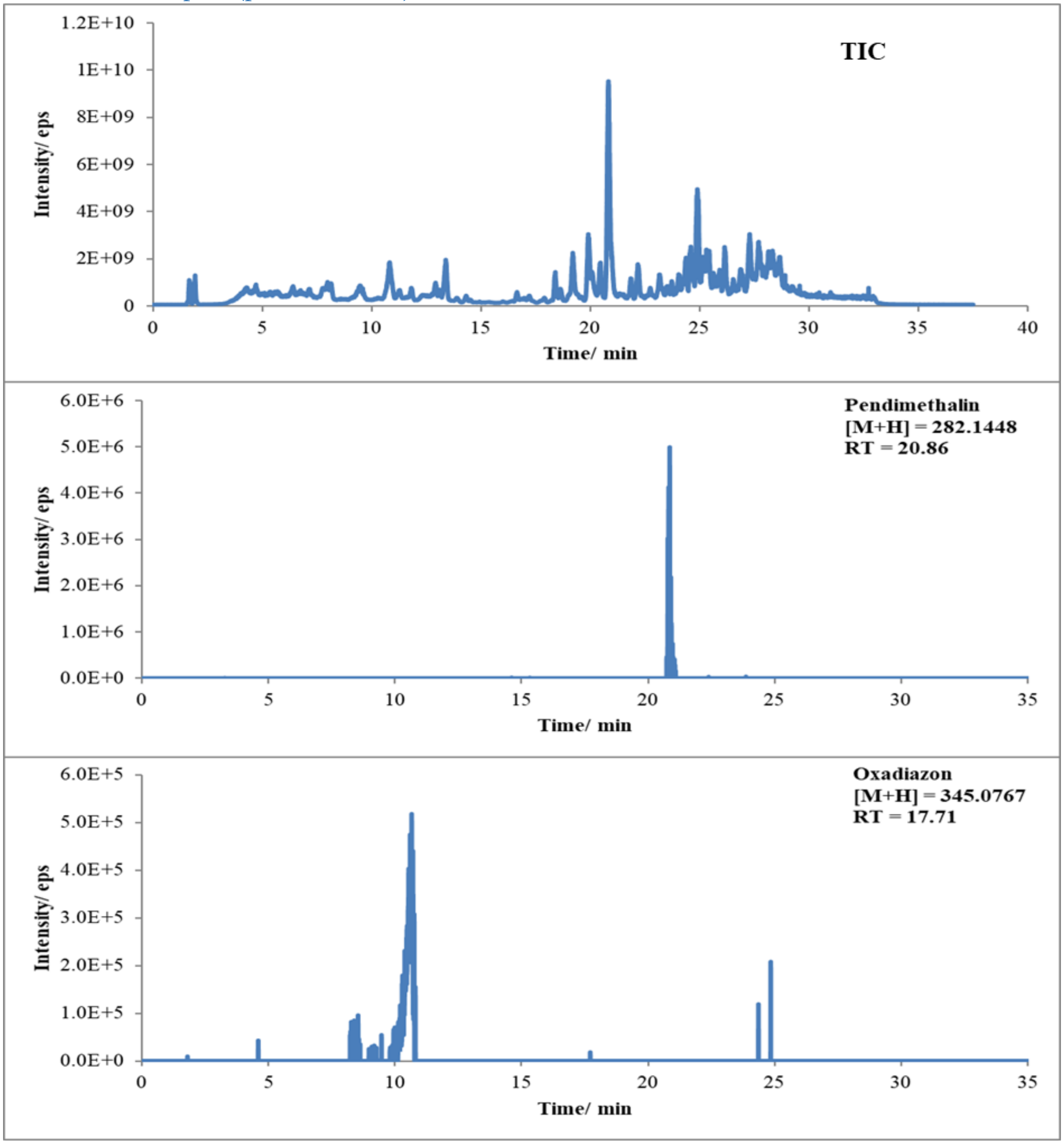

Edible chrysanthemum

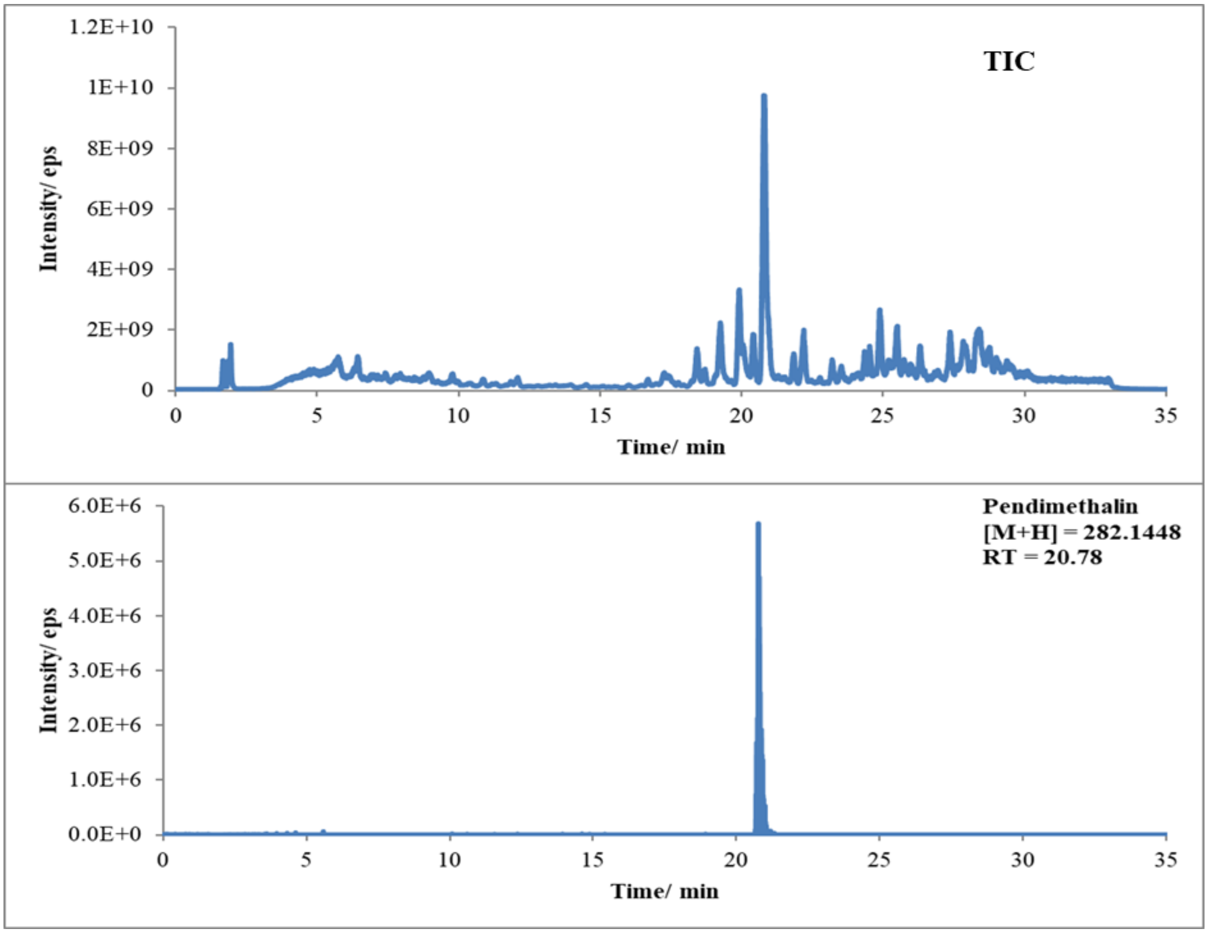

Green salad

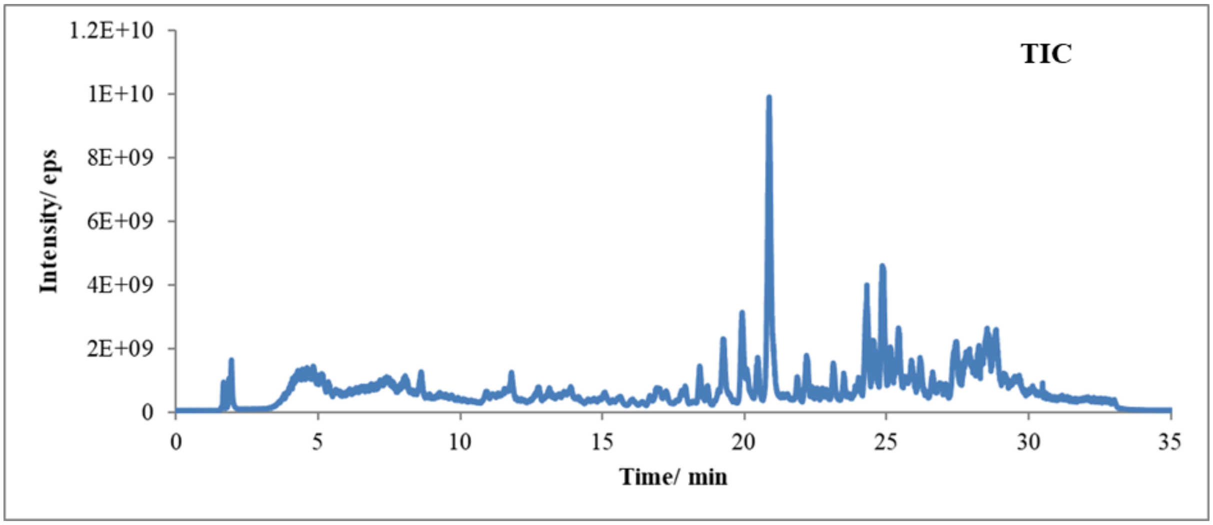

Perilla vegetable

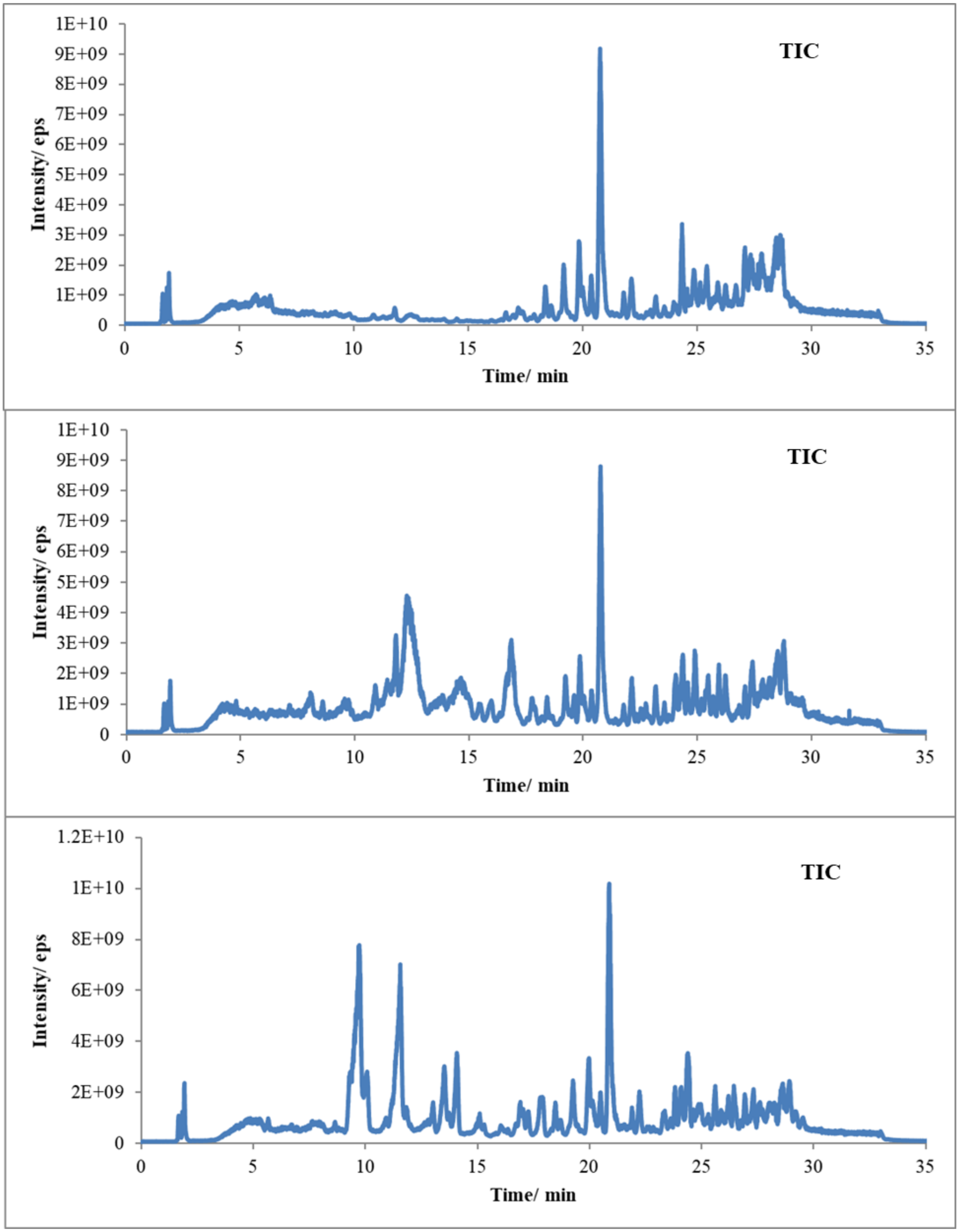

TIC of kales, oregano and coriander

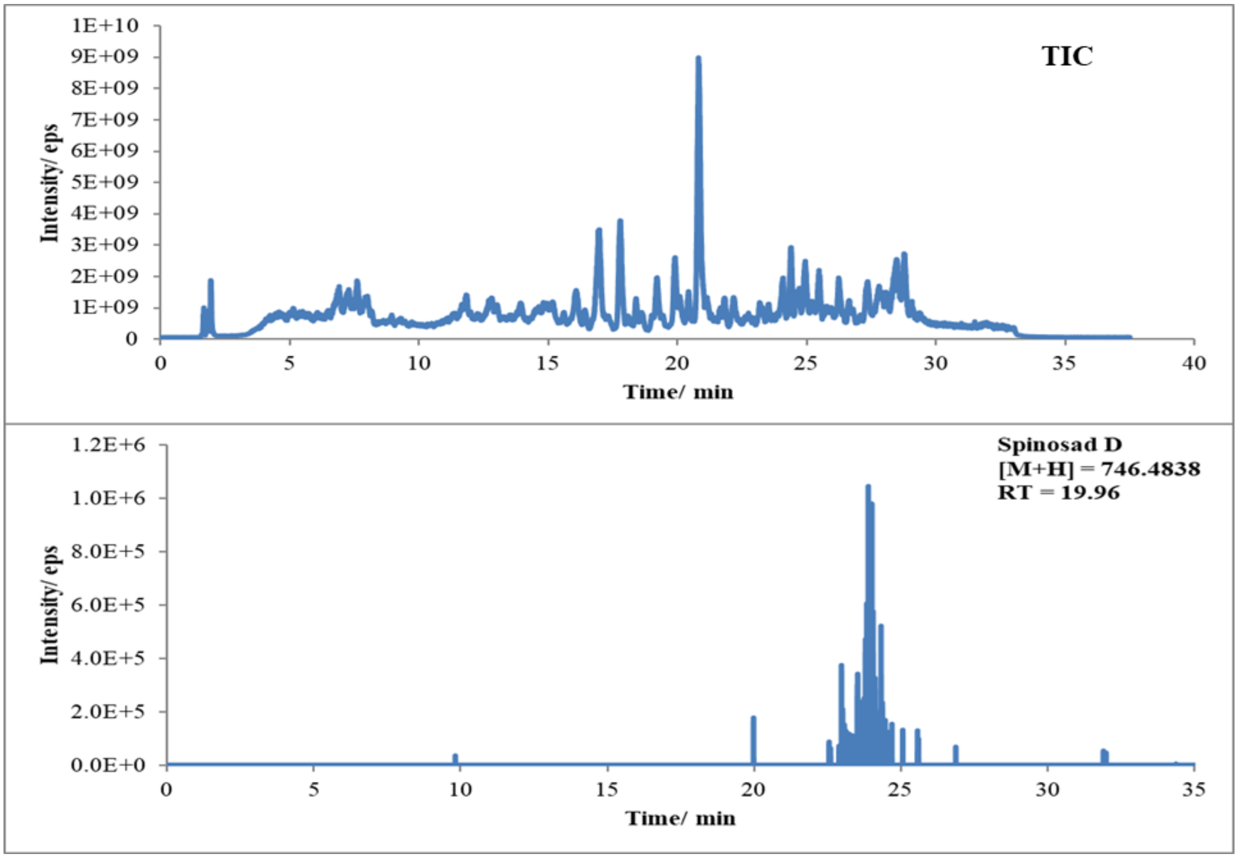

Morning spinach

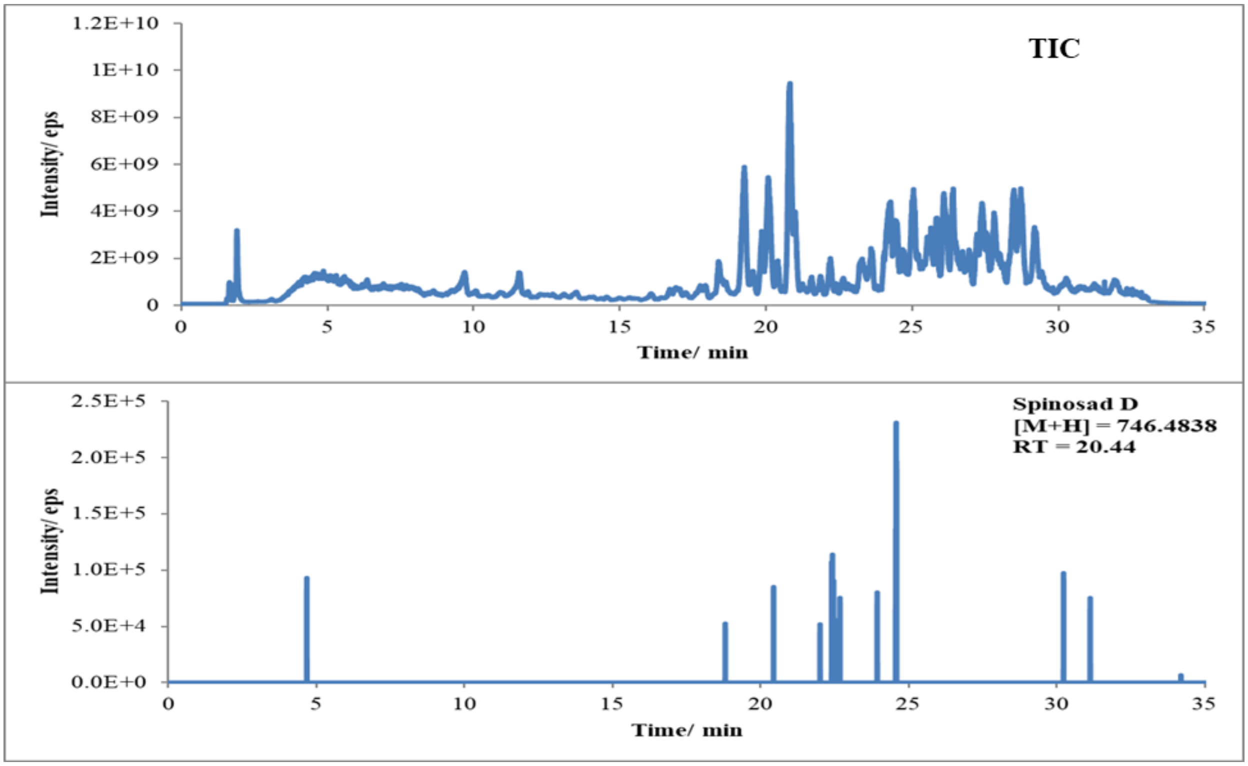

Shrinkage vegetable

Fig. S6. The total ion chromatogram and extracted ion chromatogram of the target analytes in several real samples (negative mode)

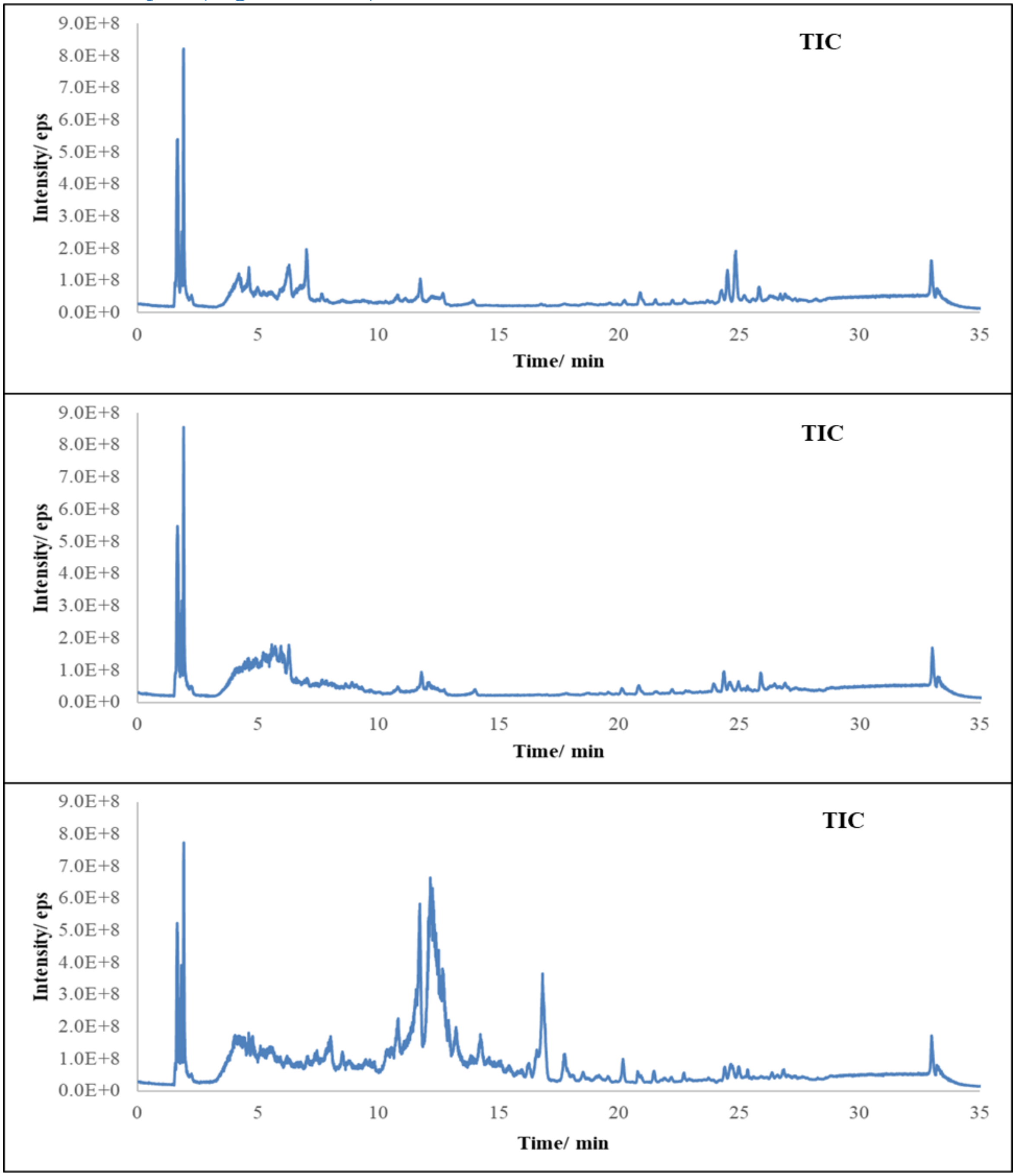

Edible chrysanthemum - kales- oregano

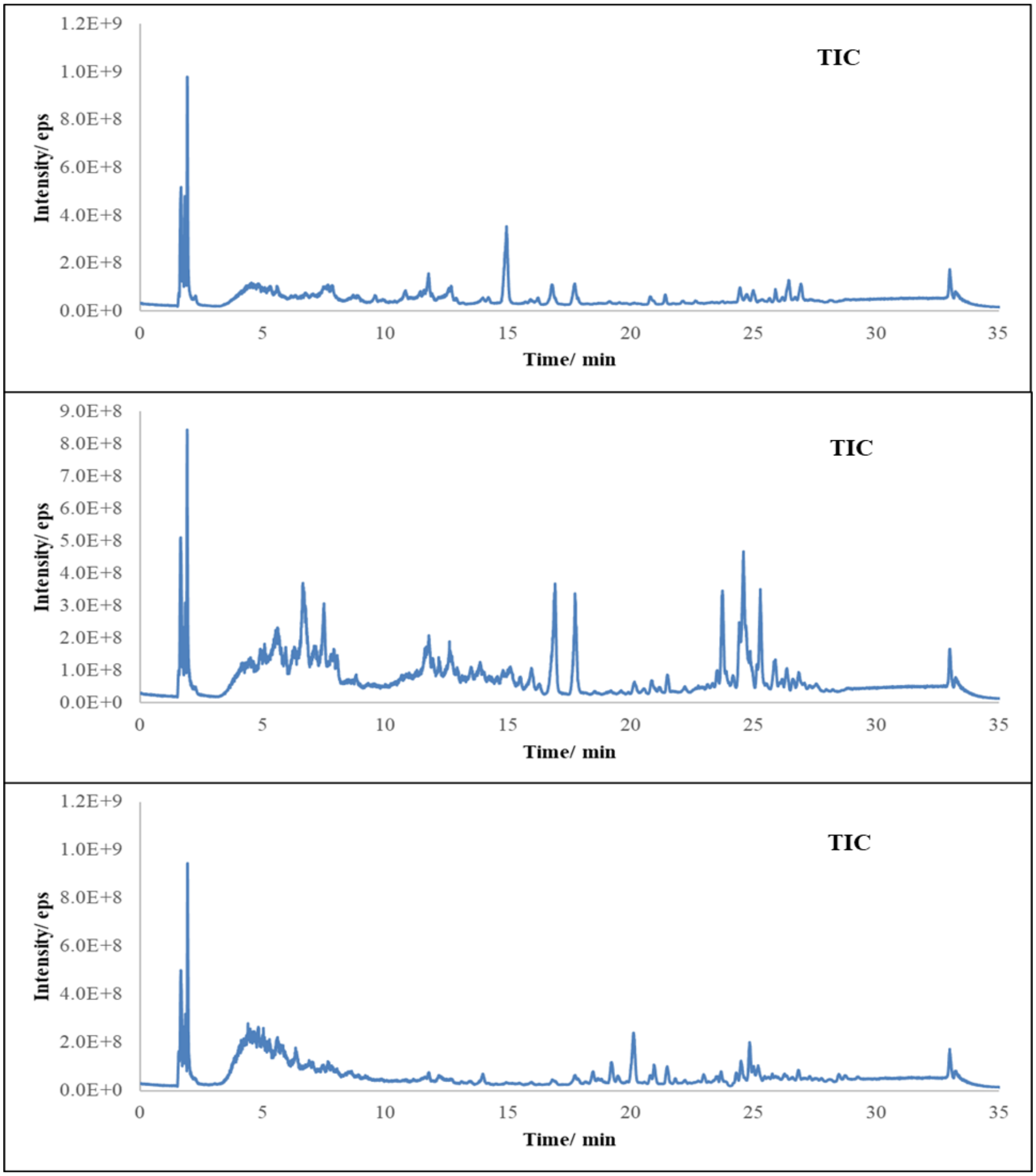

coriander- spinach- shrinkage vegetable
